# An Introductory Course on Geriatric Oncology

**DOI:** 10.15766/mep_2374-8265.11471

**Published:** 2024-11-14

**Authors:** David J. Gregorio, Kana Lucero, Sukeshi Patel Arora, Kate Lathrop, Justin Horowitz, Becky Powers

**Affiliations:** 1 Hematology/Medical Oncology Fellow, Department of Internal Medicine, University of Texas Health Science Center at San Antonio Joe R. & Teresa Lozano Long School of Medicine, and Department of Veterans Affairs (VA), South Texas Veterans Health Care System, Audie L. Murphy Veterans Memorial Medical Center; 2 Geriatrics/Palliative Medicine Fellow, Department of Internal Medicine, University of Texas Health Science Center at San Antonio Joe R. & Teresa Lozano Long School of Medicine, and Department of Veterans Affairs (VA), South Texas Veterans Health Care System, Audie L. Murphy Veterans Memorial Medical Center; 3 Associate Professor of Medicine, Division of Hematology/Oncology, Mays Cancer Center, University of Texas Health Science Center at San Antonio; 4 Associate Professor of Medicine, Division of Hematology/Oncology, Mays Cancer Center, University of Texas Health Science Center at San Antonio; Associate Professor and Hematology and Medical Oncology Fellowship Director, Department of Internal Medicine, University of Texas Health Science Center at San Antonio Joe R. & Teresa Lozano Long School of Medicine; 5 Hematology and Oncology Clinical Pharmacist, Division of Hematology/Oncology, Mays Cancer Center, University of Texas Health Science Center at San Antonio; 6 Associate Professor, Department of Veterans Affairs (VA), South Texas Veterans Health Care System, Audie L. Murphy Veterans Memorial Hospital Geriatric Research Education and Clinical Center; Associate Professor and Program Director of Geriatric Medicine Fellowship, Division of Geriatrics, Gerontology & Palliative Medicine, University of Texas Health Science Center at San Antonio Joe R. & Teresa Lozano Long School of Medicine

**Keywords:** Geriatric Medicine, Geriatric Oncology, Case-Based Learning, Geriatrics, Hematology, Oncology

## Abstract

**Introduction:**

The need to train oncologists to address the complexities of the aging population has been a focus of educational initiatives and strategies since the 1980s. However, large gaps in the dissemination and implementation of geriatric oncology curricula are still present. Currently, few resources exist for oncology training programs to implement a formal geriatric oncology curriculum. We aimed to create a formalized introductory course to teach oncology and geriatrics trainees the principles of geriatric oncology.

**Methods:**

Curriculum presentations were delivered to both hematology/oncology and geriatrics fellows during five 1-hour didactic/workshop sessions over a 2-month period. In addition to didactic presentations, sessions included interactive learning components and a case-based workshop. Evaluation of the curriculum was conducted through pre- and postcourse knowledge and competency assessments, as well as individual session satisfaction surveys.

**Results:**

Fifteen (11 hematology/oncology and four geriatric medicine) clinical fellows participated in the first presentation of this curriculum during the 2022–2023 academic year. The mean score on the precourse knowledge assessment was 7.1 (*SD* = 2.5) out of a maximum score of 15 compared with a mean score on the postcourse knowledge assessment of 9.8 (*SD* = 3.0; CI: 8.0–11.6; *t* = −2.5; *p* = .02).

**Discussion:**

Course content was successfully implemented into the hematology/oncology and geriatric medicine fellowship core curriculum using the above methods. Future directions include presentation of course material to incoming trainees, content refinement based on satisfaction surveys, and interdisciplinary adaptation for trainees in other health care disciplines (e.g., nursing, advanced practice providers, etc.).

## Educational Objectives

By the end of this activity, learners will be able to:

1. Define and distinguish between geriatric syndromes, comorbidity, and frailty.
2. Explain the domains of the Comprehensive Geriatric Assessment (CGA) and practice integrating the CGA into clinical scenarios.
3. Describe the existing biologic (age-related physiologic changes) and psychosocial (e.g., treatment goals and preferences) differences between older and younger patients.
4. Integrate abbreviated screening tools into the clinical evaluation of geriatric patients.
5. Predict chemotherapy toxicity in older adult patients.

## Introduction

In the United States, the proportion of adults over age 65 is increasing compared to other age-range cohorts, resulting in an increase in cancer diagnoses and need for oncology services. A 67% increase in cancer incidence is anticipated for older adults by 2030, and current estimates show that approximately 60% of all new cancer diagnoses occur in patients over age 65.^1^ Additionally, the geriatric population is typically considered to be more medically heterogenous, with unique syndromes, making dedicated curricula on this patient population necessary for trainees in hematology and medical oncology.^2^

Recommendations for competencies of medical oncology trainees in geriatric oncology have been published as part of the American Society of Clinical Oncology (ASCO) and the European Society of Medical Oncology (ESMO) Global Curriculum since the second edition of that document in 2010.^3^ Its recommendations were expanded in the 2016 (third) edition, highlighting the importance of incorporation of curricula into modern oncology training programs. Furthermore, a modified Delphi consensus of experts in medical oncology education and geriatric oncology was published in 2020 outlining competencies medical oncology trainees should obtain in caring for older adult patients with cancer.^4^ More recently, in 2024, the ASCO Geriatric Oncology Community of Practice published a framework detailing a 5-year strategic plan for the advancement of geriatric oncology that includes education of trainees and practicing clinicians as a top priority, in addition to research and implementation strategies.^5^

The need to train oncologists to address the complexities of the aging population in order to provide high-quality cancer care has been a focus of educational initiatives and strategies since the 1980s.^6^ However, despite previous efforts, large gaps in the dissemination and implementation of geriatric oncology curricula are still present. In 2017, a national survey of hematology/oncology trainees in the US found that 53% of respondents reported receiving no formal lectures in geriatric oncology and, when assessed on competencies, only 41% correctly identified the predictors of chemotherapy toxicity in older adults with cancer.^7^ Furthermore, a survey of hematology/oncology fellowship directors in 2008 found that only one-third of respondents incorporated a formal geriatric oncology curriculum into their program.^8^ Lastly, the global need for geriatric training in oncology cannot be overstated. Especially among low- and middle-income countries, there is a paucity of educational initiatives in geriatric oncology to train the oncology workforce regarding the complexities of older adult patients with cancer.^9^

Currently, few resources exist to aid oncology training programs in implementation of a formal geriatric oncology curriculum. Published geriatric oncology curricula are also limited. Much of the work in this area has been directed toward continuing professional development in the creation of dedicated 1- to 2-year fellowship training programs in geriatric oncology.^10^ An interprofessional geriatric oncology curriculum was successfully implemented within a tertiary health care system and outlined in the *Journal of Geriatric Oncology.*^11^ A geriatric oncology OSCE has been published in *MedEdPORTAL* for instruction in geriatric assessment tools to guide patient treatment decision-making.^12^ The International Society of Geriatric Oncology offers a 4-day advanced course in geriatric oncology held annually in person and virtually online.^13^ E-learning modules with content in geriatric oncology are currently available through ESMO's and ASCO's education platforms,^14,15^ but membership is required to access this content, limiting these resources to active members.

We aimed to create and implement a formalized introductory course to teach hematology/medical oncology and geriatrics fellows the principles of geriatric oncology to apply in caring for older adults with cancer. The approach from Kern's model for curriculum development^16^ was utilized. Prior to the curriculum's development, an educational needs assessment was performed and published separately.^17^ Next, educational objectives for the curriculum were created. Objectives were based on section XIV of the ASCO core curriculum^18^ (considered the educational framework around which a training program for medical oncologists should be developed) outlining core competencies in geriatric oncology. Curriculum objectives were reviewed and edited for content accuracy and readability by experts in hematology/oncology and geriatric medicine.

Five 1-hour lectures were created to facilitate delivery of course content, with each lecture corresponding to one educational objective. Lectures were created using Microsoft PowerPoint presentation software, with content derived from a variety of academic sources listed within each presentation. All course content was reviewed and edited by experts in hematology/oncology, geriatric medicine, and oncology specialty pharmacy for accuracy and readability.

## Methods

### Target Audience

The target audience for this educational activity included hematology/medical oncology fellows, geriatric medicine fellows, and integrated geriatrics/palliative medicine fellows at all levels of training. This educational activity was conducted with fellows at the University of Texas Health Science Center at San Antonio during the 2022–2023 academic year. Curriculum instructors included faculty in hematology/oncology and geriatrics departments, fellows in the hematology/oncology and geriatrics/palliative medicine programs, and faculty from the clinical pharmacy department. All instructors were required to have general knowledge of topics in geriatric oncology and, preferably, expertise in managing older adult patients with cancer. No particular prerequisite knowledge or learning activities/modules were required for learners, but prior knowledge in geriatrics and/or medical oncology helped orient learners to curriculum content.

### Curriculum Development

Lectures were delivered to fellows during regularly scheduled weekly didactic sessions with approval from fellowship program leadership. Lectures included both in-person and remote viewing (Microsoft Teams) formats. Each lecture contained footnotes used to describe content in the slide deck and references for source material to aid presenters giving the lecture. The five lecture topics included Introduction to Geriatric Oncology (Appendix A), The Comprehensive Geriatric Assessment (CGA; Appendix B), Geriatric Screening Tools (Appendix C), The Biology of Aging and Final Domain of the CGA (Appendix D), and Cancer Therapy in the Older Adult (Appendix E).

To increase learner engagement, we outlined case-based scenarios pertaining to the lecture content and included novel interactive sessions in each lecture. Interactive sessions were optional and not required for course completion but were recommended for improved content delivery and learner engagement. Examples of these sessions included a frailty analogy exercise, a geriatric syndromes exercise, multimorbidity simulations, and evaluation of cases using geriatric assessment tools. Descriptions and instructions for each interactive session can be found in Appendix F and clinical cases and geriatric assessments for the fifth lecture (Cancer Therapy in the Older Adult) in Appendices G (case 1), H (case 2), and I (case 3). Toxicity assessments used in these cases were based on prior work by Extermann and colleagues^19^ and Hurria and colleagues.^20^ Materials used for the optional interactive sessions are listed in Table 1. While not required for course completion, the lecture session of Appendix B may be supplemented by providing printed copies of the Montreal Cognitive Assessment form.^21^ Similarly, the lecture session of Appendix C may be supplemented with printed copies of the 2023 AGS Beers Criteria^22^ and the G-8 assessment form.^23^ Lectures were presented using digital projection to a computer monitor located within an on-site conference room.

**Table 1.**
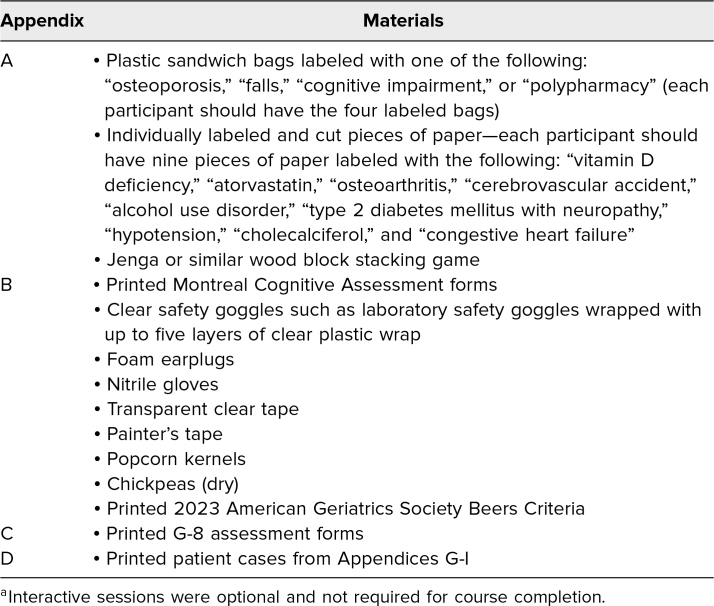
Additional Materials Used for Interactive Sessions^a^

### Evaluation

A pre-/postcurricular knowledge assessment (Appendix J) was created for this learning activity. The assessment was a 15-item multiple-choice knowledge assessment. Each curriculum objective was represented by three separate multiple-choice questions. Questions were designed to address major topics of the geriatric oncology curriculum, and the knowledge needed to answer each question was presented during didactic lectures. Questions were reviewed and edited for content accuracy and readability by experts in hematology/oncology and geriatric medicine and reviewed by medical exam question authors for fairness and adherence to best item-writing practices. Best answers to the knowledge assessment questions are listed in Appendix K. Each learner was asked to fill out the knowledge assessment before and immediately after completion of the curriculum lecture series.

A self-perceived competency evaluation (Appendix L) was also included with the pre- and postcurricular knowledge assessment. The evaluations were distributed to all learners to be filled out prior to and immediately after completion of the lecture series. The evaluation was adapted from the prior work of Denson, Manzi, Foy, Giever, and Rehm^12^ and modified with permission; it evaluated learners’ self-perceived competency, knowledge level, and comfort in performing core tasks necessary to the care of older adult patients with cancer. Self-perceived competency was rated on a 5-point Likert-type scale (1 = *unable to perform*, 2 = *perform with moderate supervision*, 3 = *perform with minimal supervision*, 4 = *perform independently*, 5 = *teach others*). Lastly, each lecture session was evaluated by learners on nine items using a 5-point Likert scale (1 = *strongly disagree*, 2 = *somewhat disagree*, 3 = *neither agree nor disagree*, 4 = *somewhat agree*, 5 = *strongly agree*) and two free-response questions pertaining to the delivery of course content during lectures (Appendix M). Lecture evaluations were also adapted from prior work by Denson and colleagues^12^ and modified with permission.

### Statistical Analysis

The Student *t* test was used to compare differences between the pre- and postcourse knowledge assessment exam scores. A two-sided *p* value less than .05 was considered statistically significant. Statistical analysis was performed using software from Social Science Statistics.^24^

## Results

Fifteen (11 hematology/oncology and four geriatric medicine) clinical fellows participated in the first presentation of this geriatric oncology curriculum during the 2022–2023 academic year. The mean number of lecture sessions attended by each fellow was 3.8 (*SD* = 0.9). Ninety-three percent of participating learners (14 of 15) completed the precourse knowledge assessment compared with 87% (13 of 15) completing the postcourse knowledge assessment. The mean score on the precourse knowledge assessment was 7.1 (*SD* = 2.5) out of a maximum score of 15 compared with a mean score on the postcourse knowledge assessment of 9.8 (*SD* = 3.0; CI, 8.0–11.6; *t* = −2.5; *p* = .02). Additionally, pre- and postcourse knowledge assessment scores demonstrated numerical improvement under each of the outlined curriculum objectives (Table 2).

**Table 2.**
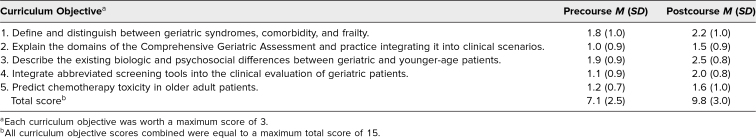
Pre- and Postcourse Knowledge Assessment Scores by Curricular Objective

Learners demonstrated numerical improvement in ratings on postcourse surveys regarding their self-perceived competency to perform tasks in pharmacology, medication adjustments, oncology screening tests, assessment tools, care coordination, capacity determination, determining cancer treatment, addressing financial burden, palliative care/hospice determination, goals-of-care discussion, managing expectations, and supportive services for older adult patients with cancer (Table 3).

**Table 3.**
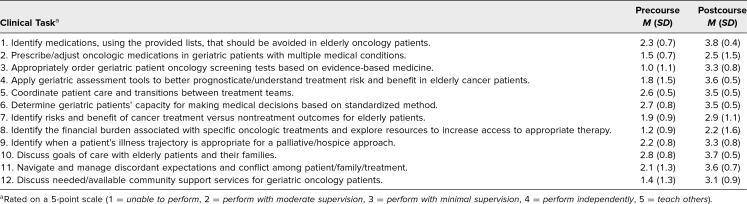
Pre- and Postcourse Self-Perceived Competency Assessment Performance According to Clinical Task

Postsession surveys (Appendix M) were collected after each lecture. Quantitative results from these surveys were mainly for curricular improvement and are beyond the scope of this publication. Qualitative information gathered from participants’ free-text responses indicated strengths of the curriculum listed as “clarity and organization” and “interesting, kept us engaged.” When learners were asked to list two things they would do differently as a result of the lecture sessions, their written responses included “apply geriatric assessment tools to better understand treatment risk,” “implementation of deprescribing tools in polypharmacy,” and “focus on patient-centered goals.”

## Discussion

Our aim with the creation of this geriatric oncology curriculum was to implement a formalized introductory course to teach hematology/medical oncology and geriatric medicine fellows the principles of geriatric oncology to apply in caring for older adults with cancer. Older adult patients pose unique challenges and differences in oncologic management in many ways from other age-range patients, making curricula devoted to this patient population necessary to successful oncology training and future practice.

Overall, delivery of the curriculum was successful in achieving the educational objectives outlined prior to course creation in several ways. First, learners demonstrated excellent participation in the curriculum. We chose to promote lectures as in-person events to both foster better collaboration between participants and encourage participation in interactive sessions. However, we still offered a videoconferencing option if learners could not attend in person, which made presentations available to a wider audience. With this method, the mean number of sessions attended by each fellow was nearly four out of five total lectures. Also, nearly all participants completed pre- and postcourse knowledge assessments and competency evaluations, as outlined above.

Next, numerically improved scores on knowledge and competency assessments were seen between pre- and postcourse evaluations. Postcourse mean score increases were statistically significant compared to precourse evaluations. When knowledge assessment items were grouped to evaluate educational objectives, numerical increases in mean postcourse scores were seen in all objectives compared to precourse assessments. On competency assessments, numerical increases in self-reported performance were seen in every clinical task listed in Appendix L on postcourse assessments compared to precourse.

Creation of this curriculum was a collaborative, multidisciplinary effort between specialists in medical hematology/oncology, geriatric medicine, and clinical pharmacy. The care of older adult patients is often multifaceted, requiring expertise in multiple disciplines to identify and manage geriatric syndromes, frailty, and comorbidity. Several prior studies and publications have highlighted the importance of multidisciplinary input for intervention in geriatric oncology.^25,26^ Lastly, overall positive feedback obtained from learners on postcourse assessment surveys indicated their satisfaction with course delivery and content.

Limitations of this curriculum include the small number of participants in its first presentation and single-site institutional presentation. These may limit generalizability of the assessment findings to larger participant audiences. Some reasons for reduced attendance included clinical service duties of fellows and absence for illness/medical leave, but other factors remain unknown because this question was not included in curriculum assessments. Future presentations should aim to increase the number of participants to help reduce this limitation with consideration of using incentives for participation, such as the creation of a geriatric oncology certification for full attendance or offering continuing education credits for licensed participants as examples. Furthermore, we hope collaborative relationships can be formed between other institutions to deliver content to a wider audience of oncology and geriatric trainees. Next, the knowledge assessment tool we used to evaluate participants was created de novo for the purposes of this curriculum. Ideally, a validated assessment would be available; however, no current validated knowledge assessments have been published for educational activities in geriatric oncology. Refinement of our knowledge assessment in future iterations would be beneficial given poor performance of several assessment items. Another limitation of the listed assessments includes their relatively long length (knowledge and self-perceived competency). This may have led to assessment fatigue in several participants and is postulated to be the reason for incomplete assessment submission. Lastly, while educational content was created by authors in hematology/medical oncology, geriatric medicine, and clinical pharmacy, further multidisciplinary and interprofessional input is lacking. Educational content input from other disciplines (e.g., surgical and radiation oncology; palliative medicine; physical, occupational, and speech therapy; psychiatry; social work; and oncology and geriatric nursing) and from advanced practice providers should be included.

Future directions include presentation of the curriculum to incoming classes of hematology/medical oncology and geriatric medicine fellows. We plan to further refine course content and assessments based on participant feedback from the initial presentation. Curriculum assessments, particularly the self-perceived competency assessment (Appendix L) will be modified to improve assessment length and clarity. We plan to broaden the audience within our institution to include trainees in surgical oncology and radiation oncology subspecialties, advanced practice providers in medical oncology, and oncology and geriatric nurse professionals. We also aim to create a geriatric oncology lecture series certification for future participants. We hope other institutions/fellowship programs can adopt this curriculum to further distribute knowledge to a broader audience of oncology providers. With further adoption, validation studies of the included geriatric oncology knowledge assessment can also take place, enabling it to be used as a broad educational assessment for geriatric oncology curricula in the future.

## Appendices


Introduction to Geriatric Oncology.pptxThe Comprehensive Geriatric Assessment.pptxGeriatric Screening Tools.pptxBiology of Aging.pptxCancer Therapy in the Older Adult.pptxSummary of Interactive Sessions.docxSession 5 Patient Case 1.docxSession 5 Patient Case 2.docxSession 5 Patient Case 3.docxGeriatric Oncology Knowledge Assessment.docxKnowledge Assessment Answer Key.docxSelf-Perceived Competency Assessment.docxCurriculum Session Assessment.docx
*All appendices are peer reviewed as integral parts of the Original Publication.*
