# Supplementary material for: An Introductory Course on Geriatric Oncology
Source: MedEdPORTAL. 2024 Nov 14;20:11471. doi: 10.15766/mep_2374-8265.11471 (PMC11561070; doi:10.15766/mep_2374-8265.11471)
Supplement: Supplementary file 1 — Introduction to Geriatric Oncology.pptxThe Comprehensive Geriatric Assessment.pptxGeriatric Screening Tools.pptxBiology of Aging.pptxCancer Therapy in the Older Adult.pptxSummary of Interactive Sessions.docxSession 5 Patient Case 1.docxSession 5 Patient Case 2.docxSession 5 Patient Case 3.docxGeriatric Oncology Knowledge Assessment.docxKnowledge Assessment Answer Key.docxSelf-Perceived Competency Assessment.docxCurriculum Session Assessment.docx [file mep_2374-8265.11471-s001.zip › J. Geriatric Oncology Knowledge Assessment.docx]

An Introductory Course to Geriatric Oncology

Knowledge Assessment

**Please circle one of the following describing your training program:**

Geriatric Medicine Fellow Integrated Geriatric/Palliative Care Fellow

Hematology/Oncology Fellow

**Please circle one of the following that describes your training level:**

PGY4 PGY5 PGY6

**How many course sessions did you attend**? _____/5

**Multiple-choice. Please circle the letter that corresponds to the best answer from the given options.**

1. A 77-year-old woman presents to clinic as a new patient to establish care for

biopsy-proven metastatic renal cell carcinoma. She has not received therapy for this diagnosis in the past and her ECOG performance status is estimated as 1. A geriatric consultant discusses polypharmacy, mild urinary incontinence, multiple recent falls at home, and the need for assistance from her daughter with daily household chores and managing her finances.

The conditions identified by the geriatrician may be collectively referred to as:

1. Frailty
2. Geriatric syndromes
3. Multimorbidity
4. Normal aging

2. A 78-year-old woman with a PMH of localized HR+, HER2 negative breast

cancer comes to clinic accompanied by her daughter for a 3-month checkup. Six months ago, she completed lumpectomy and adjuvant radiation therapy. She was not recommended for systemic chemotherapy. She reports excellent compliance with adjuvant hormonal therapy. Her daughter states over the last 6 months her mother has become more “frail” and is engaging less with other community members.

Which intervention can best slow progression of frailty?

1. Cognitive stimulation
2. Deprescribing
3. Depression evaluation and treatment
4. Nutritional supplements
5. Physical activity and balance training
6. Systemic therapy

3. According to current clinical practice guidelines, in patients aged 65 years and older receiving chemotherapy which of the following should be performed/evaluated:

1. Assessment for caregiver stress
2. Pedigree for inherited genomic risk
3. Assessment of financial distress
4. Geriatric assessment
5. Monofilament test to evaluate for pre-therapy peripheral neuropathy

4. List the 5 M’s assessed with the use of the comprehensive geriatric assessment:

_____________________

_____________________

_____________________

_____________________

_____________________

5. You receive a phone-call from a geriatrician regarding an 82-year-old patient with a newly diagnosed colon cancer. The patient presented to the ER with hematochezia, was admitted, underwent colonoscopy and was found to have an ascending colon mass. Biopsy was consistent with adenocarcinoma. Further testing reveals KRAS mutant status. The patient underwent resection, but had a slow post-operative recovery complicated by transient atrial fibrillation and pneumonia. The patient followed up with a geriatrician three weeks later. The geriatrician tells you that she has performed a comprehensive geriatric assessment (CGA) of this patient and calls to discuss those results with you, as the patient has an appointment to see you in two days. The geriatrician asks you how the use of the CGA may affect this older patient with cancer.

Which of the following BEST describes the use of the CGA for an older patient with cancer?

1. It decreases the rate of hospitalizations for geriatric oncology patients
2. It has no impact on quality of life for geriatric oncology patients
3. It has not been prospectively validated in geriatric oncology patients with specific cancer types
4. It improves mobility in geriatric oncology patients
5. It increases survival for geriatric oncology patients

6. A colleague of yours asks for your opinion on a patient case. She is caring for a 68-year-old woman with locally advanced, unresectable pancreatic cancer. She is planning to receive full dose, combination triplet chemotherapy with a goal of downstaging her tumor. Her ECOG performance status is 1. The patient was recently evaluated by a geriatric specialist and a comprehensive geriatric assessment was performed with the following results listed in the medical record.

Basic ADL Score __6/6____ IADL Score __6/8____

Montreal Cognitive Assessment (MoCA) __26/30___

Matters Most: Quality of life, being at home with her family, and caring for her mother who lives in the same household. Patient’s Zarit Screening: __3/16___

Falls in the past year __1___

Timed-Up-and-Go __8 sec___

Your colleague asks what she should do with this information. What would you recommend as the next best step?

1. Diagnose the patient with mild cognitive impairment
2. Refer to physical therapy for gait training
3. Refer to social work for caregiver stress
4. Schedule a return visit to discuss alternative treatment options
5. Refer to occupational therapy for a home safety evaluation

7. Many changes occur as a part of physiologic aging that may affect pharmacokinetics of prescribed medication. Which of the following is increased in geriatric patients?

1. Arterial compliance
2. Cytochrome P450 system
3. Drug absorption
4. Glomerular filtration rate
5. Percentage of body fat

8. Considering the effects of aging on the cardiovascular system, which of the following statements is true?

1. Decreased arterial stiffness
2. Decreased left ventricular ejection fraction
3. Increased baroreceptor responsiveness
4. Increased left ventricular stiffness
5. Increased maximum heart rate

9. An 82-year-old male patient with a history of stage III A non-small cell lung cancer who received combination chemoradiotherapy 7 years ago returns to clinic for surveillance. A recent chest CT shows no evidence of disease recurrence. He lives at home with his wife.. He states that he feels well, but he notes he has weakness and that it takes him more time to get up from his recliner. He denies dietary changes, dysphagia, difficulty chewing, early satiety, nausea, constipation, mood, or cognitive changes. He has continued the walking program he started when he was diagnosed with cancer. Recent surveillance imaging was negative for recurrent disease. Physical exam is overall non-focal, but he does have symmetric quadricep and calf atrophy.

What term best describes the most likely cause of this patient’s muscle atrophy?

1. Anorexia
2. Depression
3. Frailty
4. Non-small cell lung cancer
5. Sarcopenia

10. A 72-year-old man with a PMH of Stage IV hepatocellular carcinoma with metastasis to the bone and peritoneum presents as a new patient for evaluation.

What screening tool score would best indicate the patient would benefit from a comprehensive geriatric assessment?

1. ECOG score of 2
2. G8 score of 8
3. Mini-Cog score of 4

d. Zarit score of 2

1. VES13 score of 2

11. Which of the following is a tool to predict chemotherapy complications in patients with cancer based on data generated by the comprehensive geriatric assessment?

1. Abbreviated CGA
2. Cancer and Age Research Group score
3. ePrognosis
4. FAST Scale
5. G8 screening tool

12. A 78-year-old man presents for a routine annual and physical exam. His wife accompanies him at today’s visit and states that he is less active over the last few months. His wife asks if there are any interventions to help improve his strength. You decide to assess his degree of frailty.

Which of the following is not included as positive in the Frail scale or Fried Frailty index ?

1. A “yes” response to “Do you have difficulty walking one block?”
2. A “no” response to the question “Do you feel full of energy?”
3. Decreased grip strength

d. Five or more comorbidities

1. Unintentional weight gain

13. Which of the following has been demonstrated to be a risk factor for increased grade 3-5 chemotherapy toxicity in adult patients ≥ 65-years-old with cancer?

1. Cancer type: Lung
2. Hemoglobin <11g/dL (male) and <10g/dL (female)
3. Reduced dose of chemotherapy
4. Reduced Karnofsky performance status
5. Reduced visual acuity

14. A 72-year-old woman presents to your clinic with a newly diagnosed IgG kappa multiple myeloma, which was found by her primary physician after noticing progressive renal dysfunction. She is married and has an active social life in her community. Her ECOG performance status is 1. She would like to receive treatment and is contemplating a combination induction regimen with intention to proceed to autologous stem cell transplant.

What is most likely to help her complete treatment as planned?

1. Comprehensive geriatric assessment
2. Deprescribing
3. Physical therapy and strength training exercise to preserve her performance status
4. Referral to nutrition for dietary supplements
5. She is not considered a transplant candidate so change therapy to a reduced-intensity regimen

15. A 76-year-old woman is admitted to the hospital with signs and symptoms of volume overload. Her HR is 109, BP is 159/95, O2 sat is 93% on 2L NC. The patient appears uncomfortable. This is her third admission in the past 2 months for similar symptoms. A recent echocardiogram shows reduced left ventricular ejection fraction. Her past medical history includes a history of breast cancer treated by a local medical oncologist 12 years ago. The patient is unsure about prior hormone receptor status of her tumor or the exact treatments she received, but she does recall having surgery and chemotherapy. No medical records are available at this time.

What is the most likely cause of her presenting symptoms?

1. Chemotherapy toxicity
2. Hormone therapy toxicity
3. Recurrent breast cancer
4. Toxicity from a monoclonal antibody treatment
5. Uncontrolled hypertension
