# Supplementary material for: An Introductory Course on Geriatric Oncology
Source: MedEdPORTAL. 2024 Nov 14;20:11471. doi: 10.15766/mep_2374-8265.11471 (PMC11561070; doi:10.15766/mep_2374-8265.11471)
Supplement: Supplementary file 1 — Introduction to Geriatric Oncology.pptxThe Comprehensive Geriatric Assessment.pptxGeriatric Screening Tools.pptxBiology of Aging.pptxCancer Therapy in the Older Adult.pptxSummary of Interactive Sessions.docxSession 5 Patient Case 1.docxSession 5 Patient Case 2.docxSession 5 Patient Case 3.docxGeriatric Oncology Knowledge Assessment.docxKnowledge Assessment Answer Key.docxSelf-Perceived Competency Assessment.docxCurriculum Session Assessment.docx [file mep_2374-8265.11471-s001.zip › L. Self-Perceived Competency Assessment.docx]

**Please CIRCLE as appropriate:** PGY-4 PGY-5 PGY-6 **How many course sessions did you attend?** _____/5

**Please CIRCLE as appropriate:** Hematology/Oncology Fellow Geriatric Medicine Fellow

**Age** _________

| **Directions: Rate Your Current Level of Competence to Perform the tasks listed below:** | **For this task are you?** | |  | **How is your Performance?** | | | | |
| --- | --- | --- | --- | --- | --- | --- | --- | --- |
|  | **Knowledgeable**  **Yes (Y) / No (N)** | **Comfortable**  **Yes (Y) / No (N)** | | **Unable to Perform** | Perform with **MODERATE Supervision** | Perform with **MINIMAL Supervision** | Perform **Independently** | **TEACH Others** |
| **Patient Care** |  |  | |  |  |  |  |  |
| 1. Identify medications, using the BEERS/FACE Lists that should be avoided in elderly oncology patients (May increase delirium, heart failure, renal failure). | __Y  __N | __Y  __N | |  |  |  |  |  |
| 1. Prescribe/adjust oncologic medications in geriatric patients with multiple medical conditions (e.g., heart, lung, renal, liver disease). | __Y  __N | __Y  __N | |  |  |  |  |  |
| 1. Appropriately order geriatric patient oncology screening tests based on evidence based medicine (avoid false positives and underdiagnosis). | __Y  __N | __Y  __N | |  |  |  |  |  |
| 1. Apply geriatric assessment tools to better prognosticate/understand treatment risk and benefit in elderly cancer patients (e.g., MOCA, Mini-Cog, Barthel ADL scale). | __Y  __N | __Y  __N | |  |  |  |  |  |
| 1. Coordinate patient care and transitions between treatment teams (e.g., primary care, oncology, radiation oncology, palliative care, hospice). | __Y  __N | __Y  __N | |  |  |  |  |  |
| **Medical Decision Making** |  |  | |  |  |  |  |  |
| 1. Determine geriatric patient’s capacity for making medical decisions based on standardized method (1. gather information, 2. process information, 3. apply to self, 4. express choice). | __Y  __N | __Y  __N | |  |  |  |  |  |
| 1. Identify risks and benefit of cancer treatment versus non-treatment outcomes for elderly patients (Life expectancy, treatment side effects, complications, “quality of life”). | __Y  __N | __Y  __N | |  |  |  |  |  |
| 1. Identify the financial burden associated with specific oncologic treatments and explore resources to increase access to appropriate therapy. | __Y  __N | __Y  __N | |  |  |  |  |  |
| 1. Identify when a patient’s illness trajectory is appropriate for a palliative/hospice approach (e.g, matching disease course to palliative/home care or Medicare Hospice Benefit). | __Y  __N | __Y  __N | |  |  |  |  |  |
| **Patient Centered Communication** |  |  | |  |  |  |  |  |
| 1. Discuss goals of care with elderly patients and their families (patient/family preference, principles of medical ethics). | __Y  __N | __Y  __N | |  |  |  |  |  |
| 1. Navigate & manage discordant expectations and conflict among patient/family/treatment team. | __Y  __N | __Y  __N | |  |  |  |  |  |
| 1. Discuss needed/available community support services for geriatric oncology patients. | __Y  __N | __Y  __N | |  |  |  |  |  |

Image by Kathryn Denson, retrieved from: https://www.mededportal.org/doi/10.15766/mep_2374-8265.9860 on 9/1/2023. Creative Commons License associated: https://creativecommons.org/licenses/by/4.0/legalcode

Reference:

Denson K, Manzi G, Foy P, Giever T, Rehm J. Geriatric Oncology OSCE: Using Geriatric Assessment Tools to Guide Patient Treatment Decisions. *MedEdPORTAL*. 2014;10:9860. doi:doi:10.15766/mep_2374-8265.9860
