# Supplementary material for: An Introductory Course on Geriatric Oncology
Source: MedEdPORTAL. 2024 Nov 14;20:11471. doi: 10.15766/mep_2374-8265.11471 (PMC11561070; doi:10.15766/mep_2374-8265.11471)
Supplement: Supplementary file 1 — Introduction to Geriatric Oncology.pptxThe Comprehensive Geriatric Assessment.pptxGeriatric Screening Tools.pptxBiology of Aging.pptxCancer Therapy in the Older Adult.pptxSummary of Interactive Sessions.docxSession 5 Patient Case 1.docxSession 5 Patient Case 2.docxSession 5 Patient Case 3.docxGeriatric Oncology Knowledge Assessment.docxKnowledge Assessment Answer Key.docxSelf-Perceived Competency Assessment.docxCurriculum Session Assessment.docx [file mep_2374-8265.11471-s001.zip › M. Curriculum Session Assessment.docx]

Please circle one of the following describing your training program:

Hematology/Oncology Fellow Geriatric Medicine Fellow

Please circle one of the following that describes your level of training:

PGY-4 PGY-5 PGY-6

| Please circle the number that reflects the extent to which you *agree* or *disagree* with the following statements | **Strongly Disagree** | **Disagree Somewhat** | **Neither Agree or Disagree** | **Agree Somewhat** | **Strongly Agree** |
| --- | --- | --- | --- | --- | --- |
| 1. Session objectives were clearly stated. | 1 | 2 | 3 | 4 | 5 |
| 1. Session objectives were accomplished by the presenters. | 1 | 2 | 3 | 4 | 5 |
| 1. Material presented in a clear, organized manner | 1 | 2 | 3 | 4 | 5 |
| 1. The teaching session was interesting | 1 | 2 | 3 | 4 | 5 |
| 1. The session content was related to the skills and knowledge I need | 1 | 2 | 3 | 4 | 5 |
| 1. I have the confidence to use the knowledge gained from the workshop in patient care | 1 | 2 | 3 | 4 | 5 |
| 1. Instructional strategies/approaches advanced my learning about caring for my elderly patients | 1 | 2 | 3 | 4 | 5 |
| 1. Overall, the presenters were effective teachers | 1 | 2 | 3 | 4 | 5 |
| 1. Overall content of this session was excellent | 1 | 2 | 3 | 4 | 5 |
| 1. Overall effectiveness of today’s session was excellent | 1 | 2 | 3 | 4 | 5 |

Image by Kathryn Denson, retrieved from: https://www.mededportal.org/doi/10.15766/mep_2374-8265.9860 on 9/1/2023. Creative Commons License associated: https://creativecommons.org/licenses/by/4.0/legalcode

1. List 2 things you will do differently as a result of today’s session when caring for your next elderly oncology patient.
2. List strengths associated with the session.
3. List areas for improvement with the session.

Cited reference: Denson K, Manzi G, Foy P, Giever T, Rehm J. Geriatric Oncology OSCE: Using Geriatric Assessment Tools to Guide Patient Treatment Decisions. *MedEdPORTAL*. 2014;10:9860. doi:doi:10.15766/mep_2374-8265.9860
