# Supplementary material for: An Introductory Course on Geriatric Oncology
Source: MedEdPORTAL. 2024 Nov 14;20:11471. doi: 10.15766/mep_2374-8265.11471 (PMC11561070; doi:10.15766/mep_2374-8265.11471)
Supplement: Supplementary file 1 — Introduction to Geriatric Oncology.pptxThe Comprehensive Geriatric Assessment.pptxGeriatric Screening Tools.pptxBiology of Aging.pptxCancer Therapy in the Older Adult.pptxSummary of Interactive Sessions.docxSession 5 Patient Case 1.docxSession 5 Patient Case 2.docxSession 5 Patient Case 3.docxGeriatric Oncology Knowledge Assessment.docxKnowledge Assessment Answer Key.docxSelf-Perceived Competency Assessment.docxCurriculum Session Assessment.docx [file mep_2374-8265.11471-s001.zip › A. Introduction to Geriatric Oncology.pptx]

## Slide 1
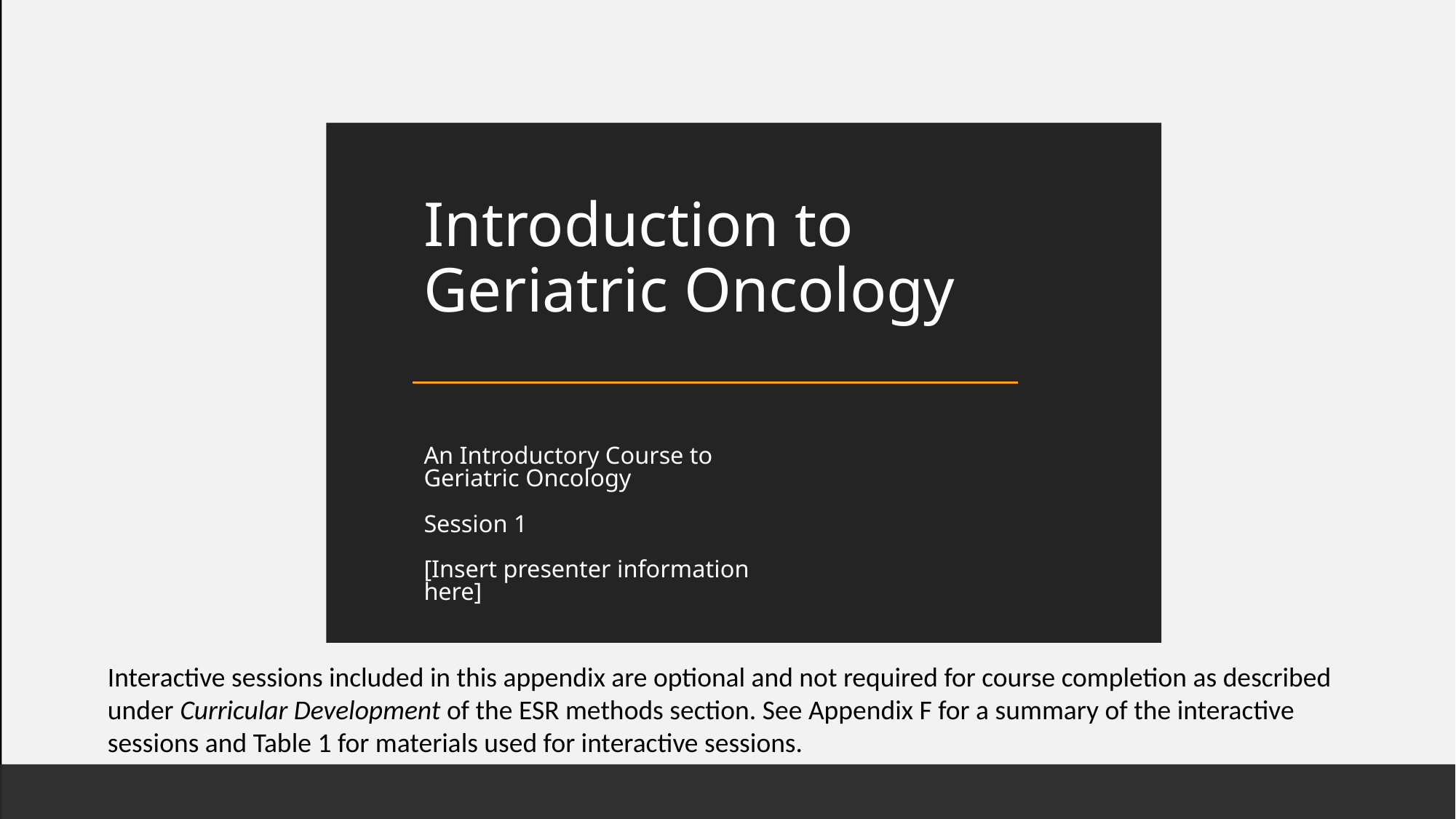

# Introduction to Geriatric Oncology
An Introductory Course to Geriatric Oncology
Session 1
[Insert presenter information here]
Interactive sessions included in this appendix are optional and not required for course completion as described under Curricular Development of the ESR methods section. See Appendix F for a summary of the interactive sessions and Table 1 for materials used for interactive sessions.

## Slide 2
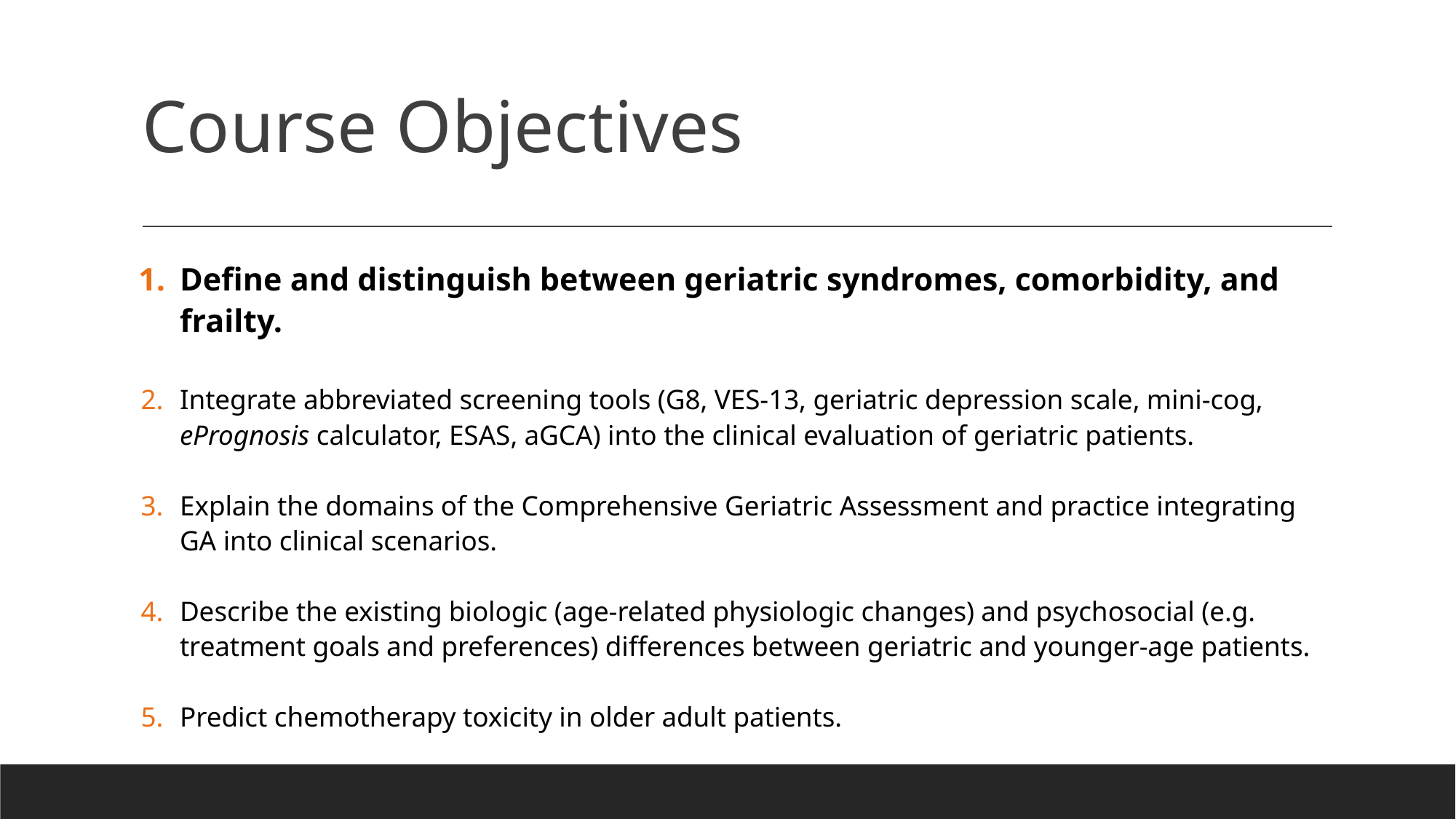

# Course Objectives
Define and distinguish between geriatric syndromes, comorbidity, and frailty. ​
Integrate abbreviated screening tools (G8, VES-13, geriatric depression scale, mini-cog, ePrognosis calculator, ESAS, aGCA) into the clinical evaluation of geriatric patients.
Explain the domains of the Comprehensive Geriatric Assessment and practice integrating GA into clinical scenarios. ​
Describe the existing biologic (age-related physiologic changes) and psychosocial (e.g. treatment goals and preferences) differences between geriatric and younger-age patients. ​​
Predict chemotherapy toxicity in older adult patients.​

## Slide 3
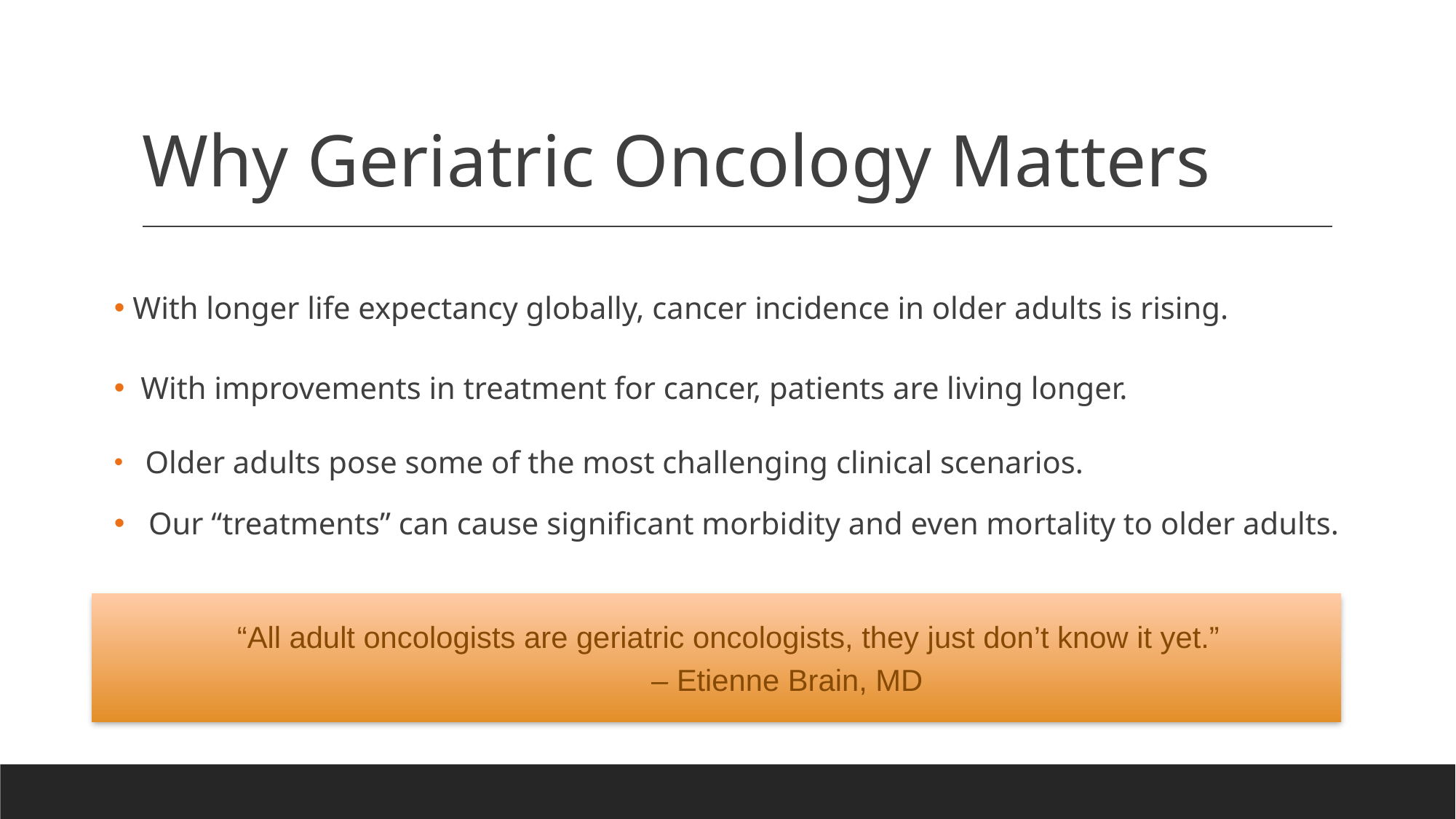

# Why Geriatric Oncology Matters
 With longer life expectancy globally, cancer incidence in older adults is rising.
 With improvements in treatment for cancer, patients are living longer.
 Older adults pose some of the most challenging clinical scenarios.
 Our “treatments” can cause significant morbidity and even mortality to older adults.
“All adult oncologists are geriatric oncologists, they just don’t know it yet.”
	 – Etienne Brain, MD

## Slide 4
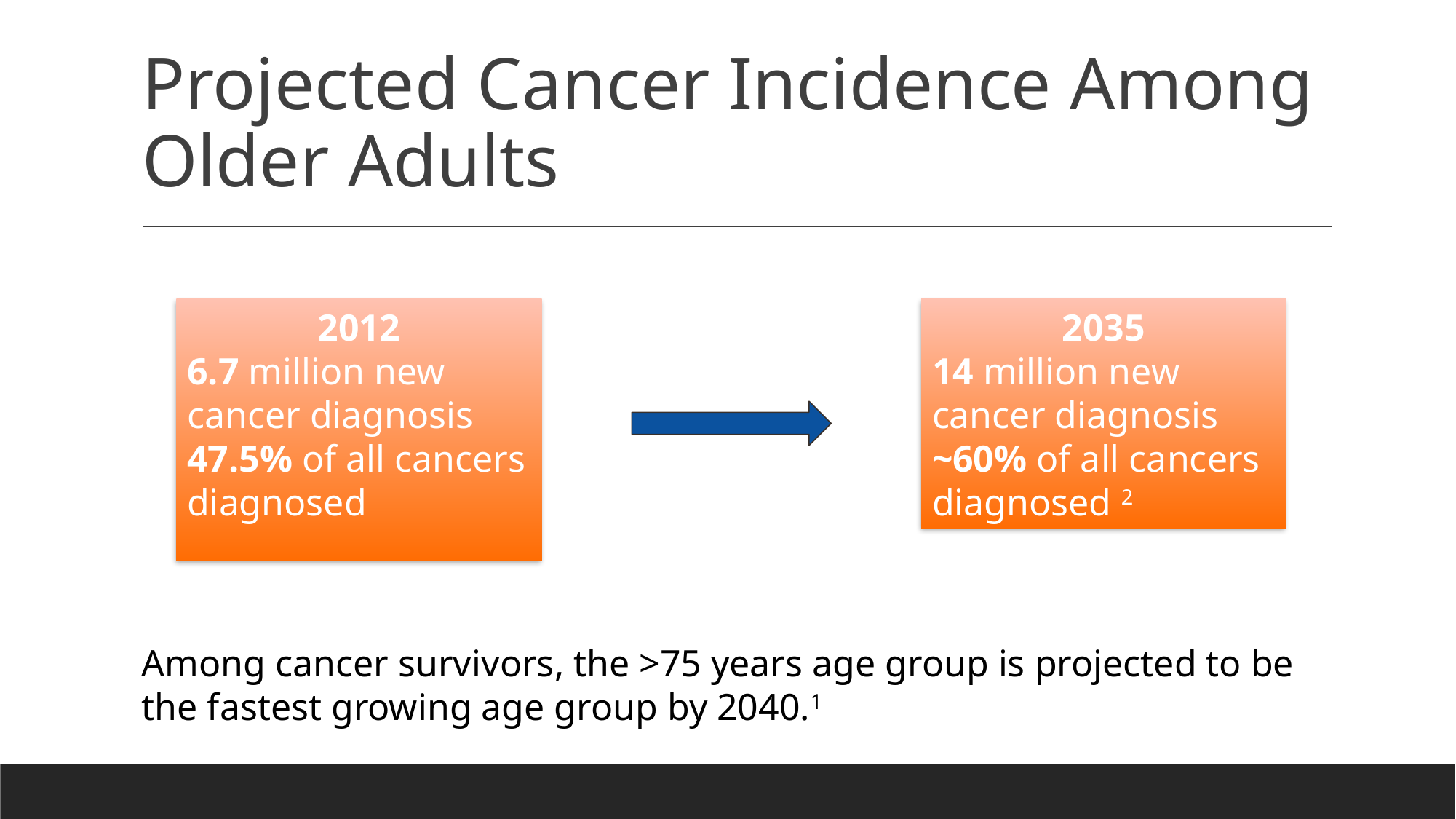

# Projected Cancer Incidence Among Older Adults
2012
6.7 million new cancer diagnosis
47.5% of all cancers diagnosed
2035
14 million new cancer diagnosis
~60% of all cancers diagnosed 2
Among cancer survivors, the >75 years age group is projected to be the fastest growing age group by 2040.1

## Slide 5
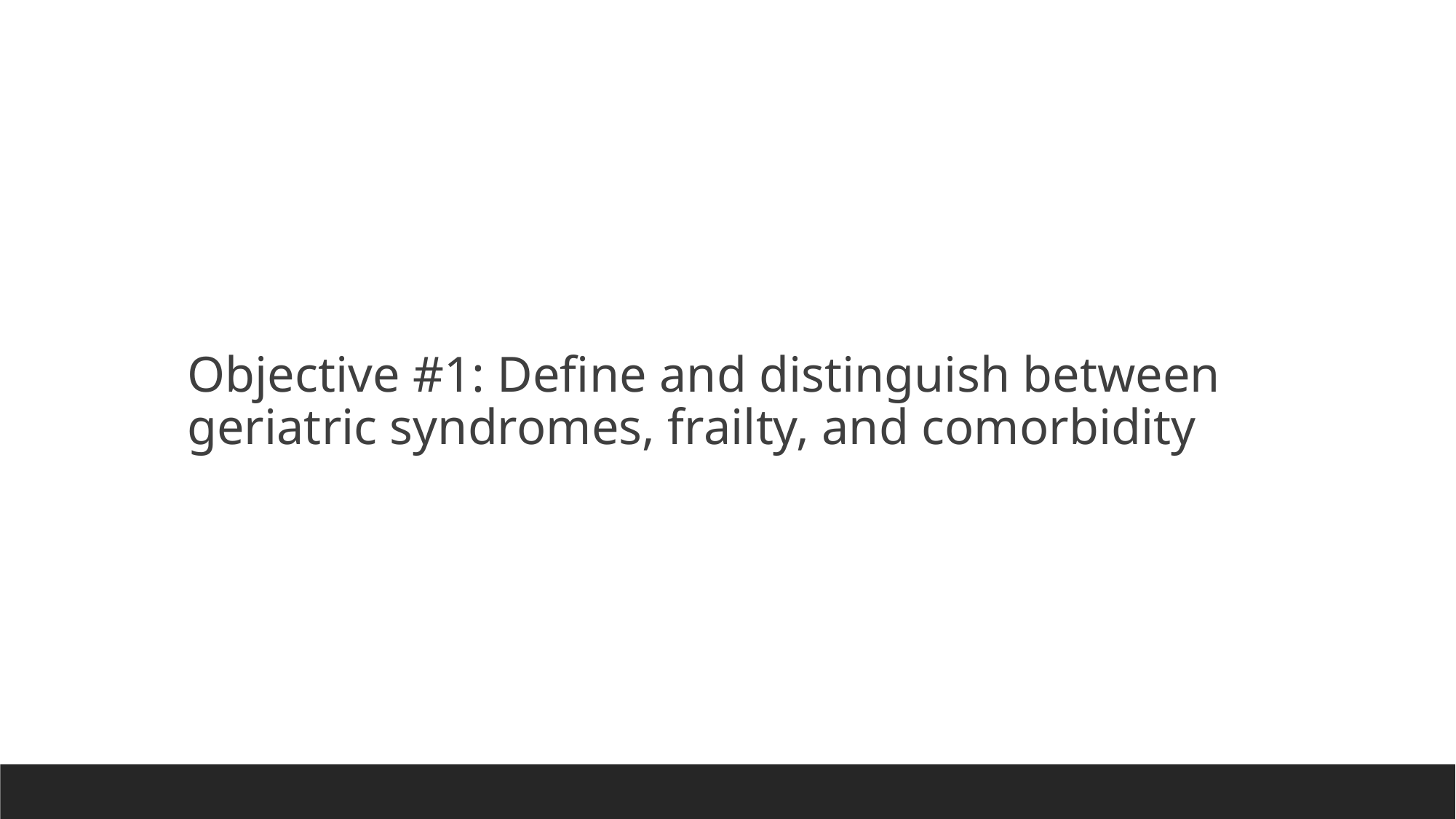

Objective #1: Define and distinguish between geriatric syndromes, frailty, and comorbidity

## Slide 6
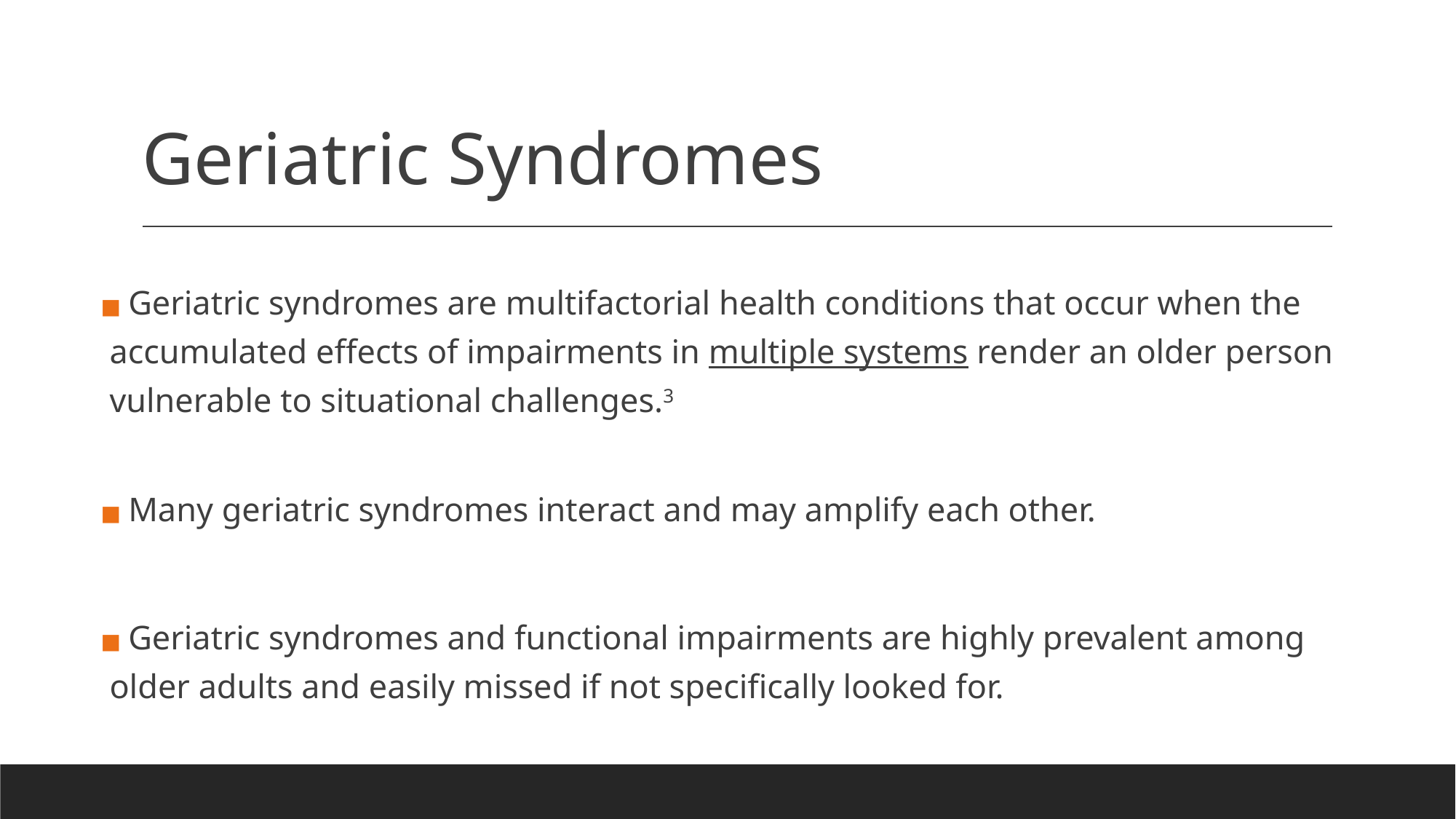

# Geriatric Syndromes
 Geriatric syndromes are multifactorial health conditions that occur when the accumulated effects of impairments in multiple systems render an older person vulnerable to situational challenges.3
 Many geriatric syndromes interact and may amplify each other.
 Geriatric syndromes and functional impairments are highly prevalent among older adults and easily missed if not specifically looked for.

## Slide 7
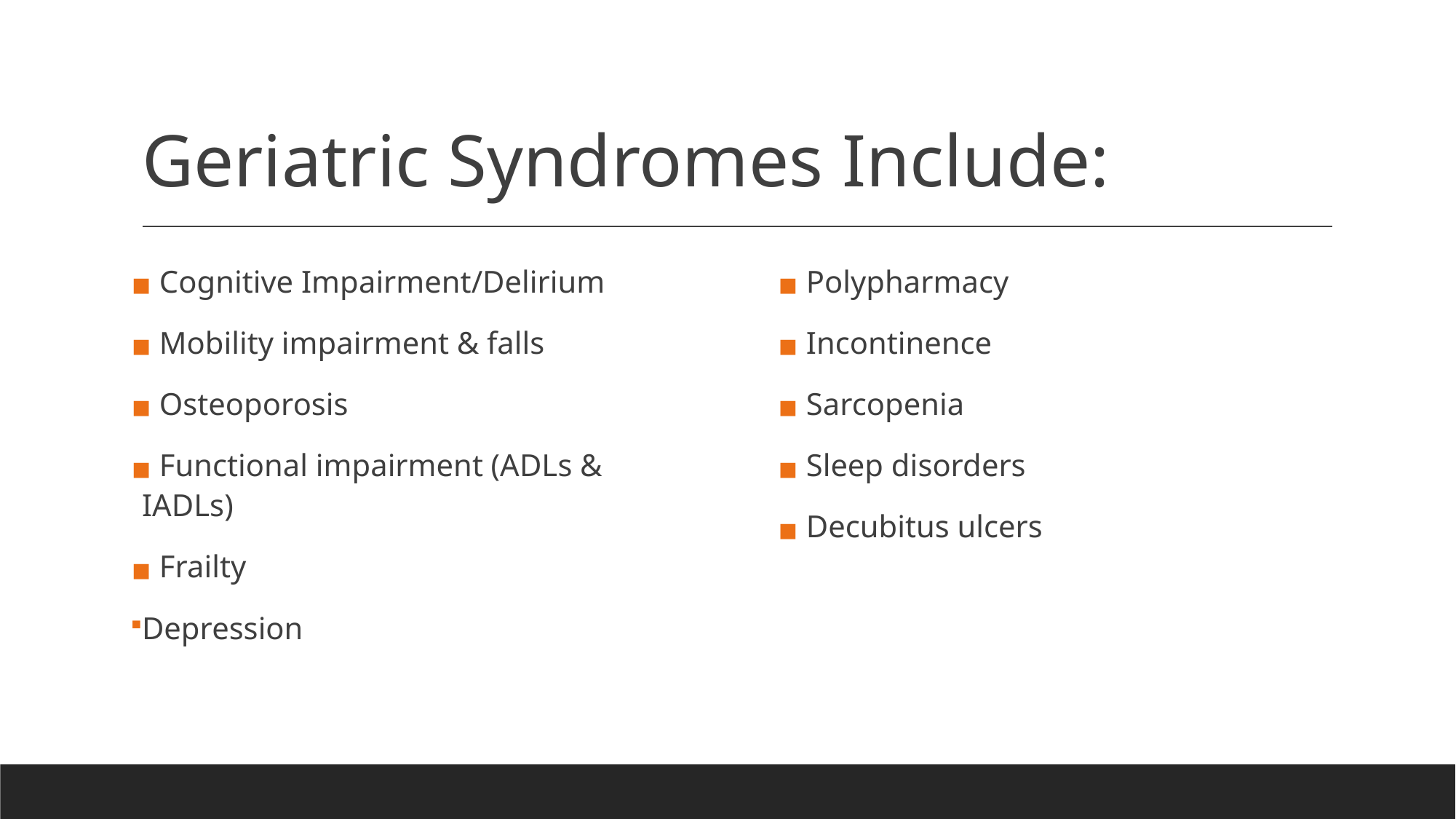

# Geriatric Syndromes Include:
 Cognitive Impairment/Delirium
 Mobility impairment & falls
 Osteoporosis
 Functional impairment (ADLs & IADLs)
 Frailty
Depression
 Polypharmacy
 Incontinence
 Sarcopenia
 Sleep disorders
 Decubitus ulcers

## Slide 8
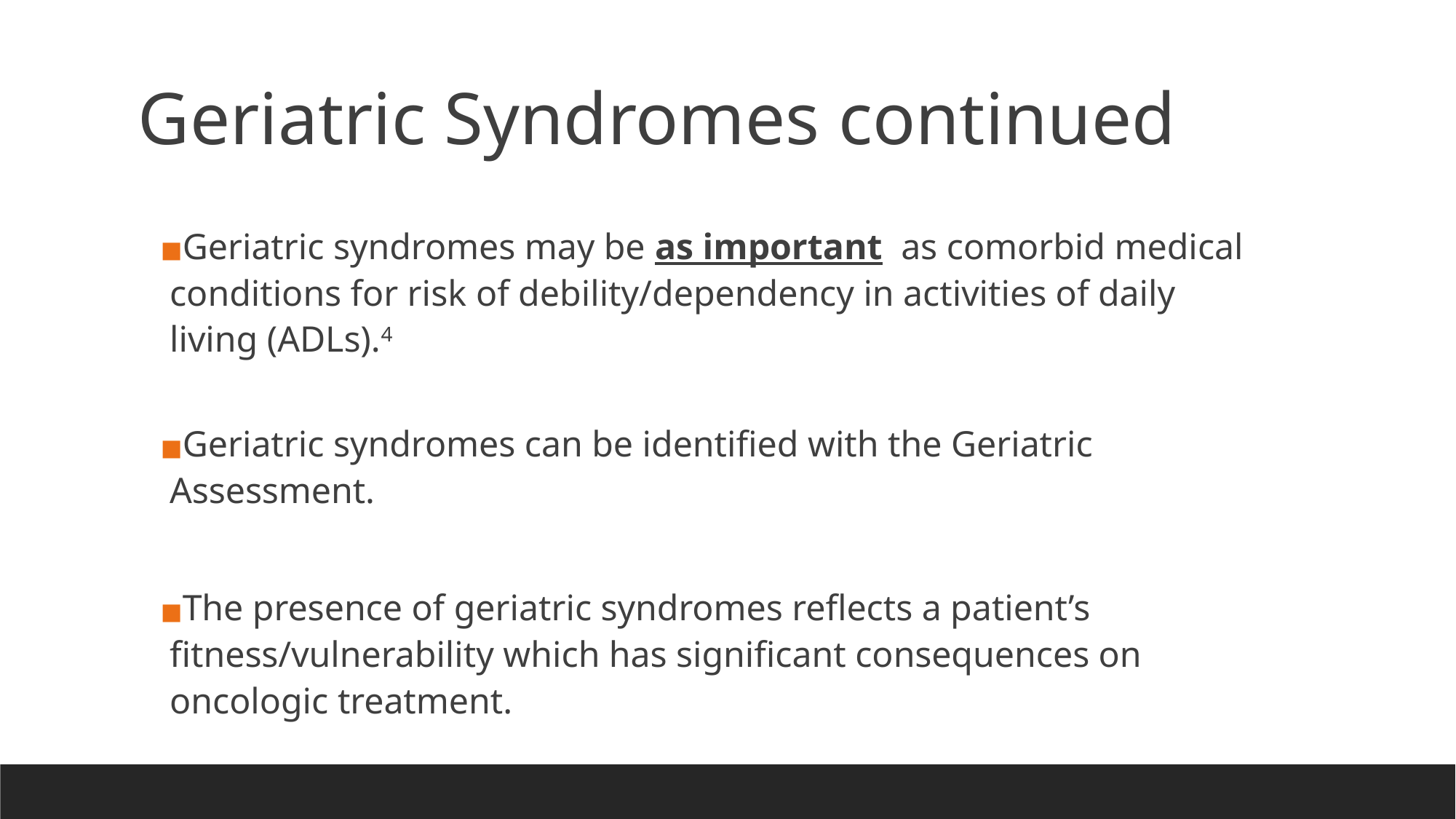

Geriatric Syndromes continued
 Geriatric syndromes may be as important as comorbid medical conditions for risk of debility/dependency in activities of daily living (ADLs).4
 Geriatric syndromes can be identified with the Geriatric Assessment.
 The presence of geriatric syndromes reflects a patient’s fitness/vulnerability which has significant consequences on oncologic treatment.

## Slide 9
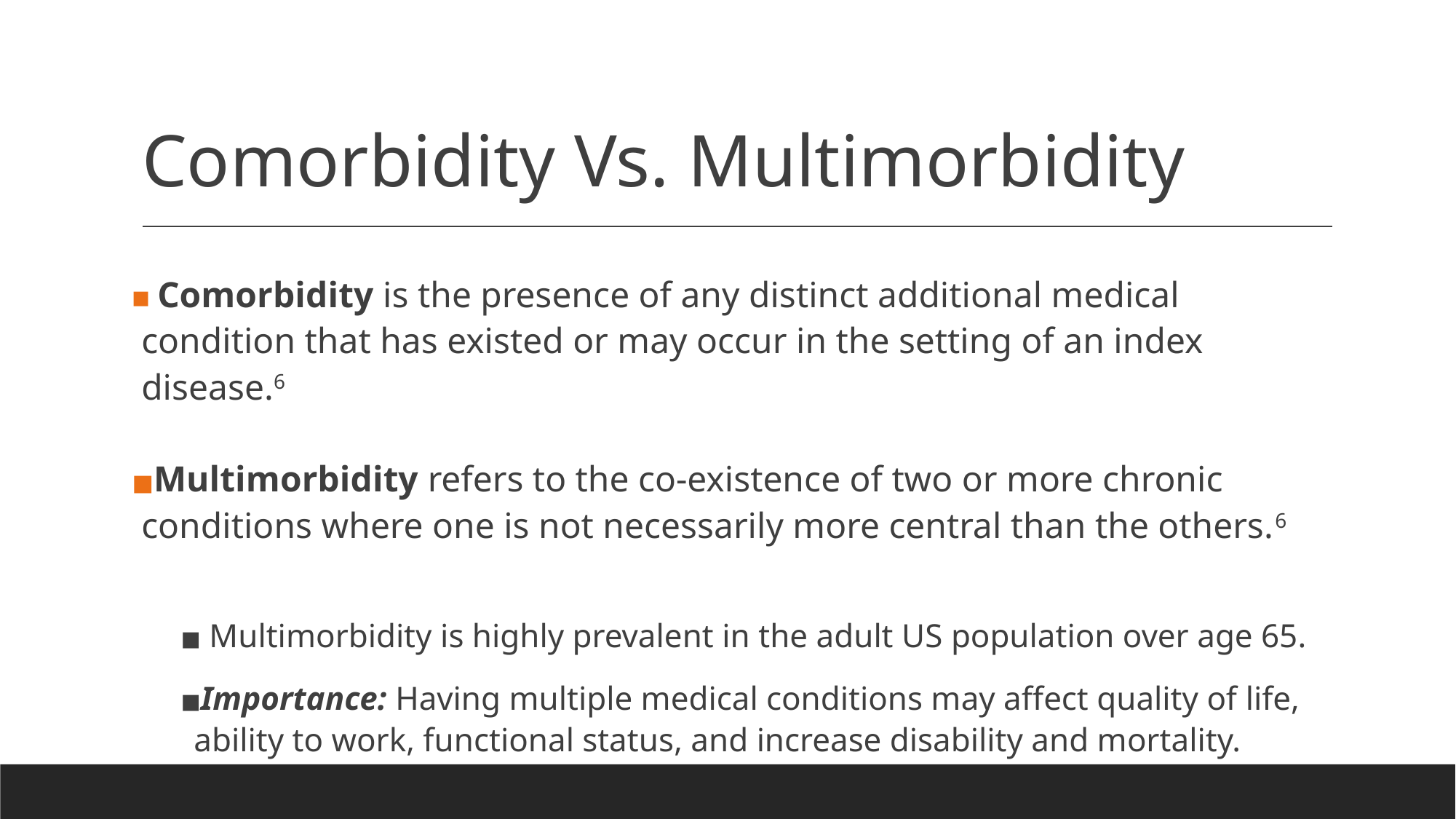

# Comorbidity Vs. Multimorbidity
 Comorbidity is the presence of any distinct additional medical condition that has existed or may occur in the setting of an index disease.6
Multimorbidity refers to the co-existence of two or more chronic conditions where one is not necessarily more central than the others.6
 Multimorbidity is highly prevalent in the adult US population over age 65.
Importance: Having multiple medical conditions may affect quality of life, ability to work, functional status, and increase disability and mortality.

## Slide 10
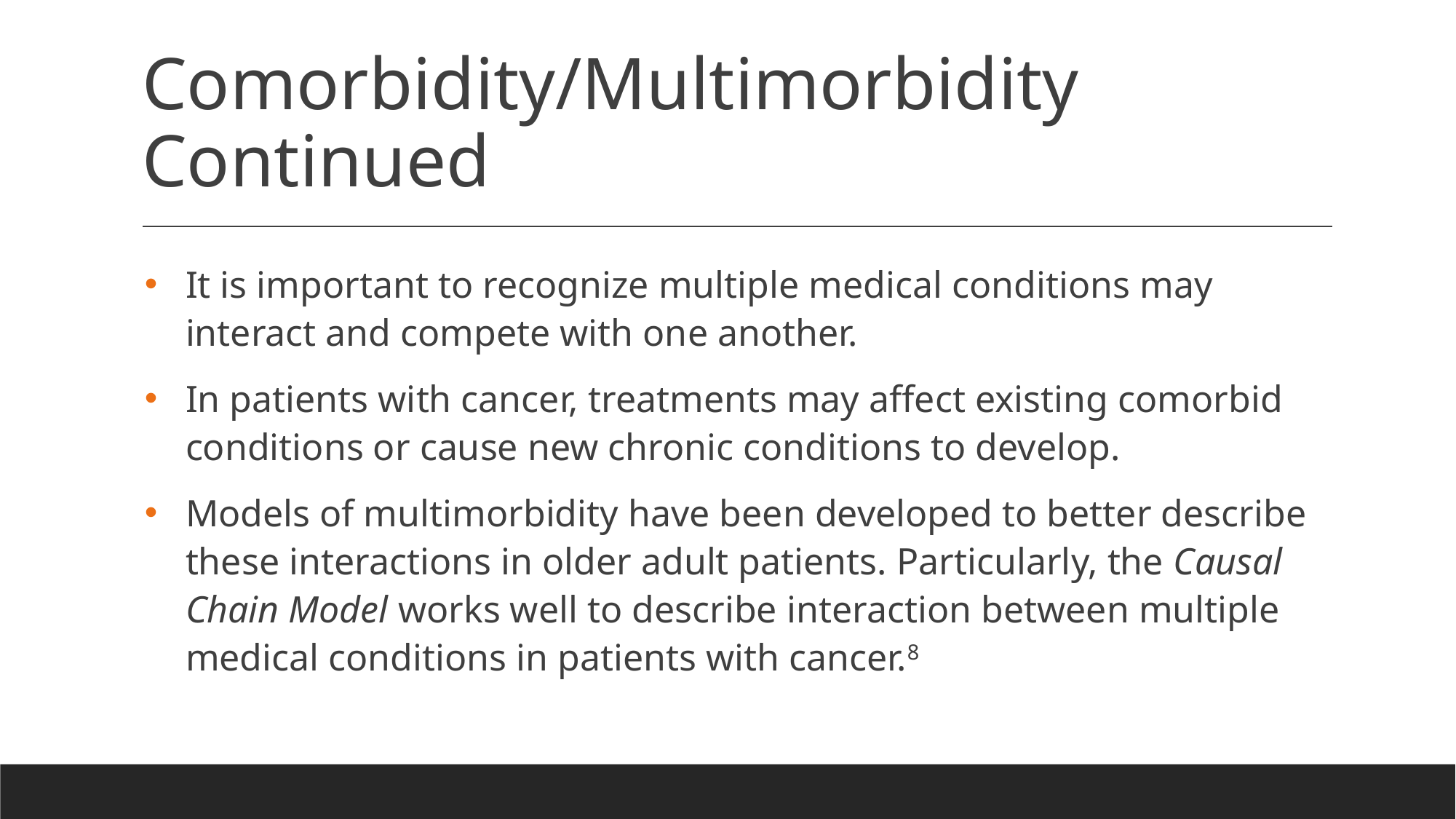

# Comorbidity/Multimorbidity Continued
It is important to recognize multiple medical conditions may interact and compete with one another.
In patients with cancer, treatments may affect existing comorbid conditions or cause new chronic conditions to develop.
Models of multimorbidity have been developed to better describe these interactions in older adult patients. Particularly, the Causal Chain Model works well to describe interaction between multiple medical conditions in patients with cancer.8

## Slide 11
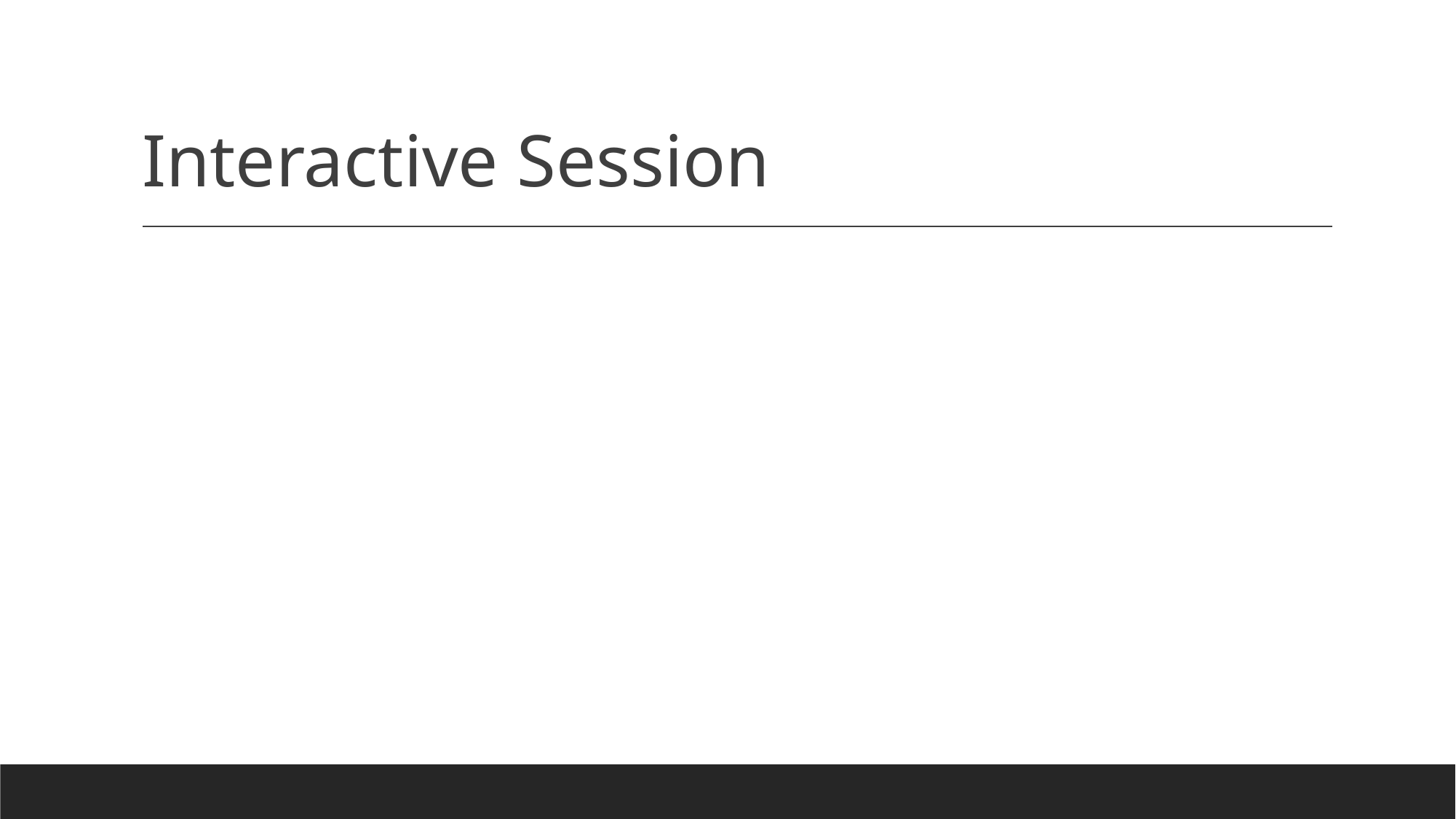

# Interactive Session

## Slide 12
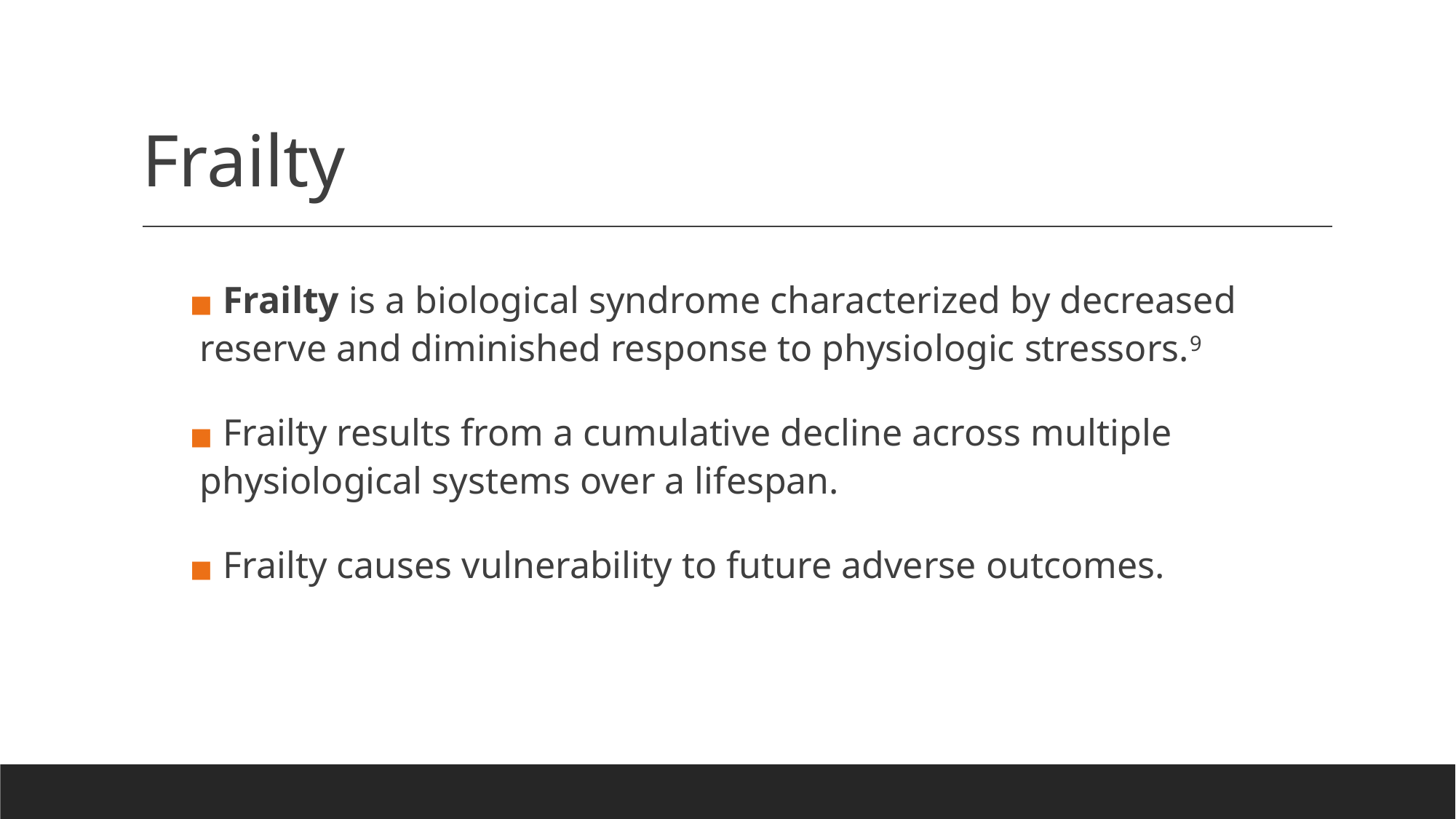

# Frailty
 Frailty is a biological syndrome characterized by decreased reserve and diminished response to physiologic stressors.9
 Frailty results from a cumulative decline across multiple physiological systems over a lifespan.
 Frailty causes vulnerability to future adverse outcomes.

## Slide 13
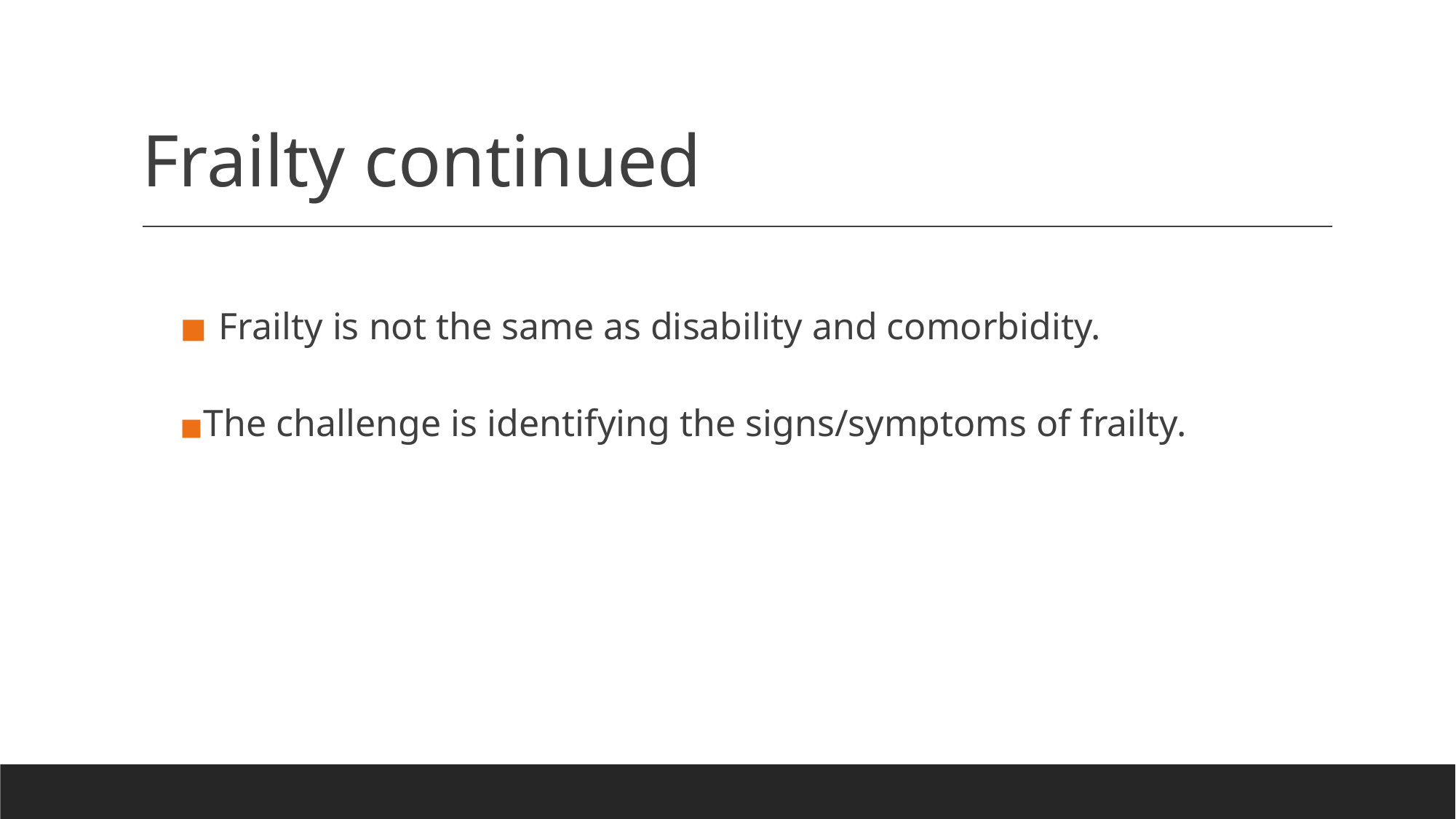

# Frailty continued
 Frailty is not the same as disability and comorbidity.
The challenge is identifying the signs/symptoms of frailty.

## Slide 14
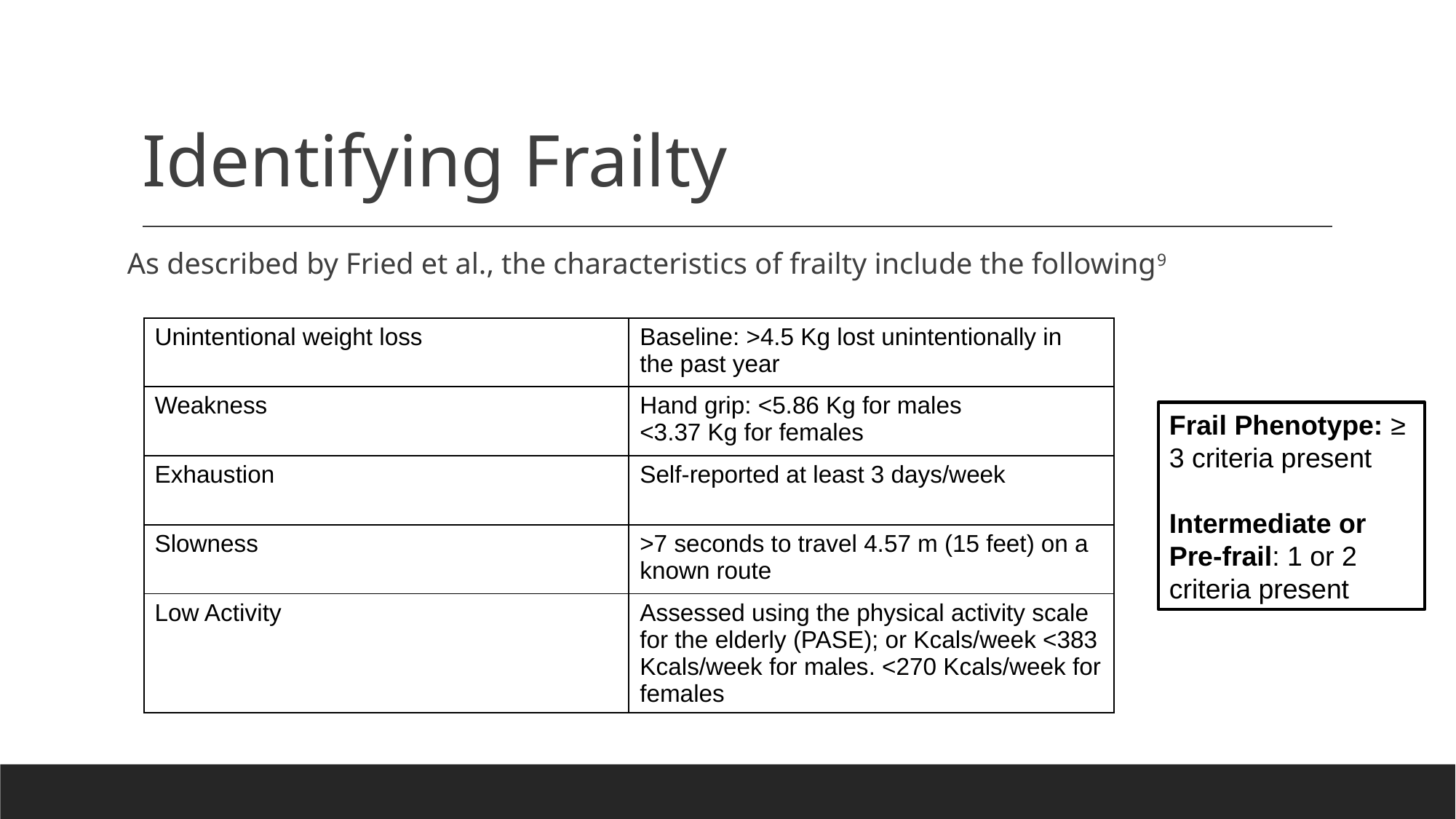

# Identifying Frailty
As described by Fried et al., the characteristics of frailty include the following9
| Unintentional weight loss | Baseline: >4.5 Kg lost unintentionally in the past year |
| --- | --- |
| Weakness | Hand grip: <5.86 Kg for males <3.37 Kg for females |
| Exhaustion | Self-reported at least 3 days/week |
| Slowness | >7 seconds to travel 4.57 m (15 feet) on a known route |
| Low Activity | Assessed using the physical activity scale for the elderly (PASE); or Kcals/week <383 Kcals/week for males. <270 Kcals/week for females |
Frail Phenotype: ≥ 3 criteria present
Intermediate or Pre-frail: 1 or 2 criteria present

## Slide 15
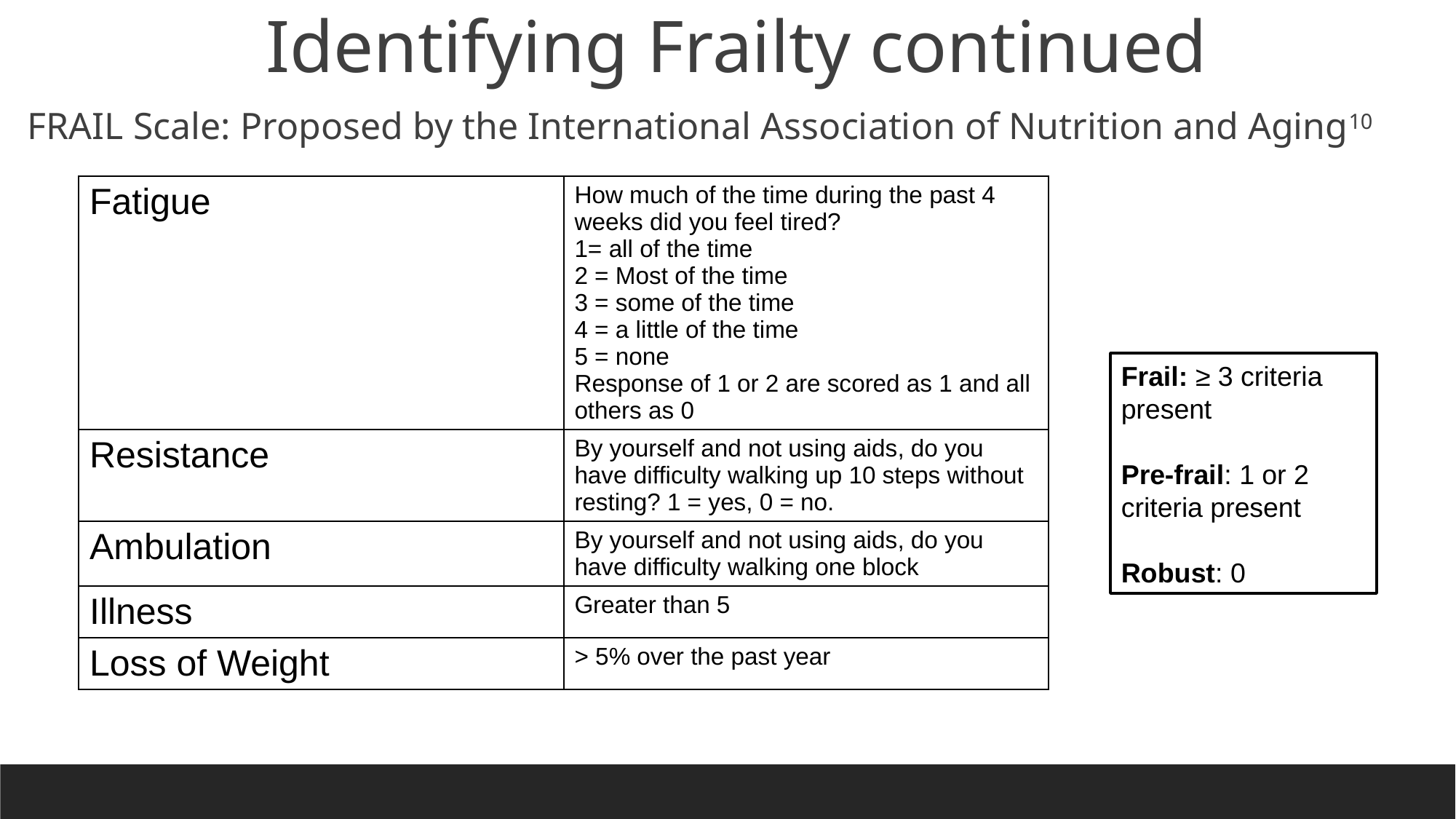

Identifying Frailty continued
FRAIL Scale: Proposed by the International Association of Nutrition and Aging10
| Fatigue | How much of the time during the past 4 weeks did you feel tired? 1= all of the time 2 = Most of the time 3 = some of the time 4 = a little of the time 5 = none Response of 1 or 2 are scored as 1 and all others as 0 |
| --- | --- |
| Resistance | By yourself and not using aids, do you have difficulty walking up 10 steps without resting? 1 = yes, 0 = no. |
| Ambulation | By yourself and not using aids, do you have difficulty walking one block |
| Illness | Greater than 5 |
| Loss of Weight | > 5% over the past year |
Frail: ≥ 3 criteria present
Pre-frail: 1 or 2 criteria present
Robust: 0

## Slide 16
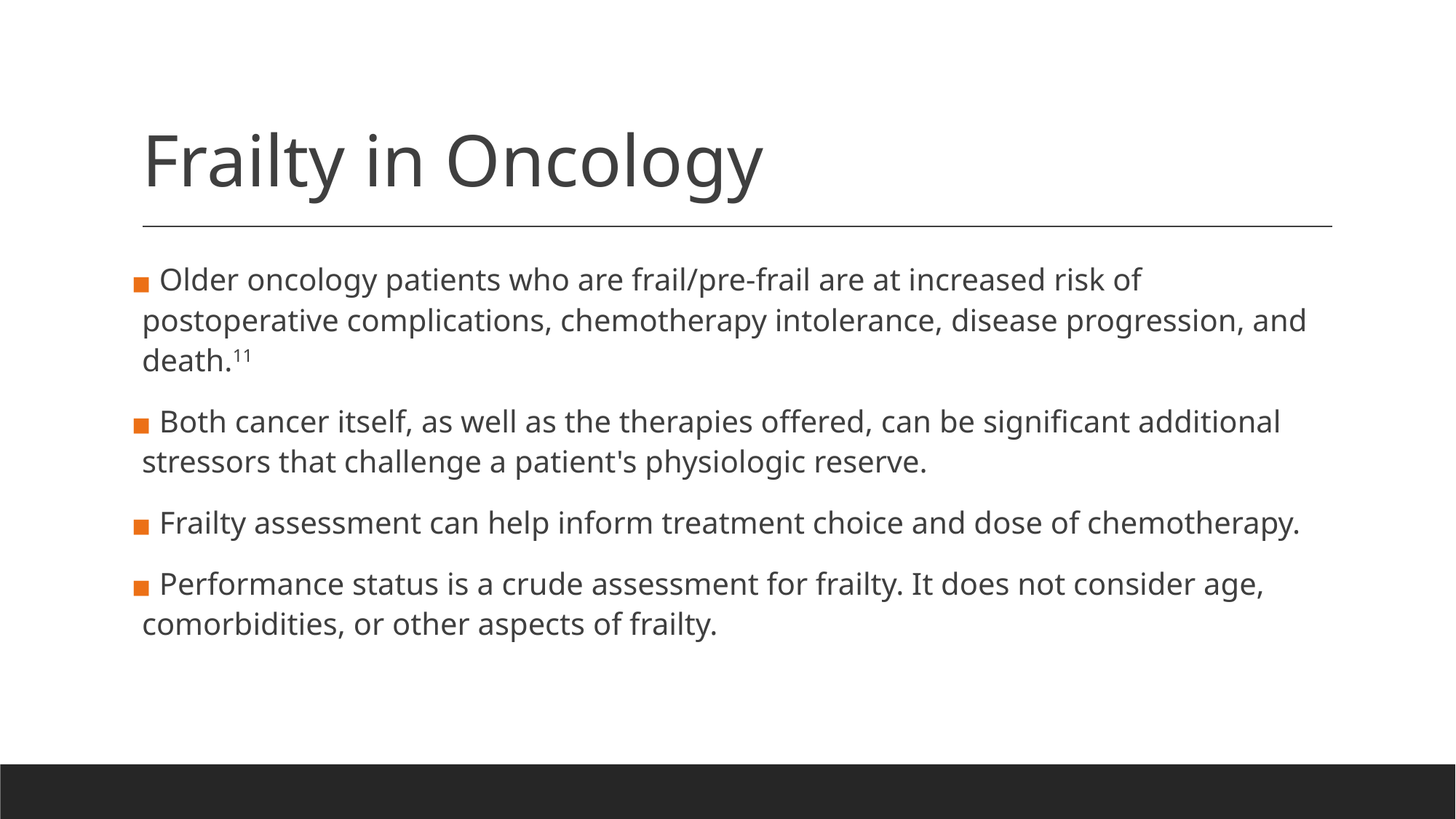

# Frailty in Oncology
 Older oncology patients who are frail/pre-frail are at increased risk of postoperative complications, chemotherapy intolerance, disease progression, and death.11
 Both cancer itself, as well as the therapies offered, can be significant additional stressors that challenge a patient's physiologic reserve.
 Frailty assessment can help inform treatment choice and dose of chemotherapy.
 Performance status is a crude assessment for frailty. It does not consider age, comorbidities, or other aspects of frailty.

## Slide 17
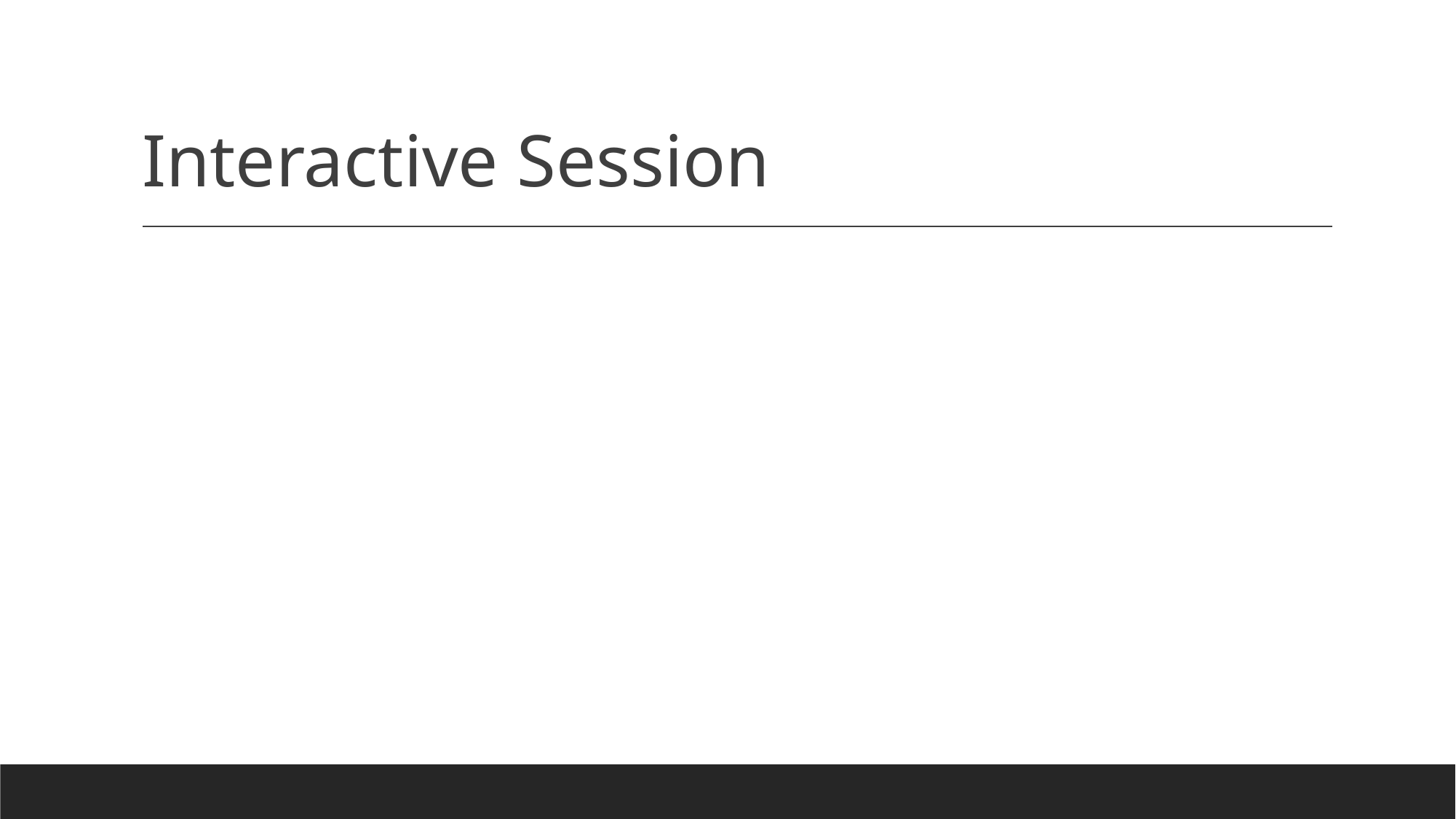

# Interactive Session

## Slide 18
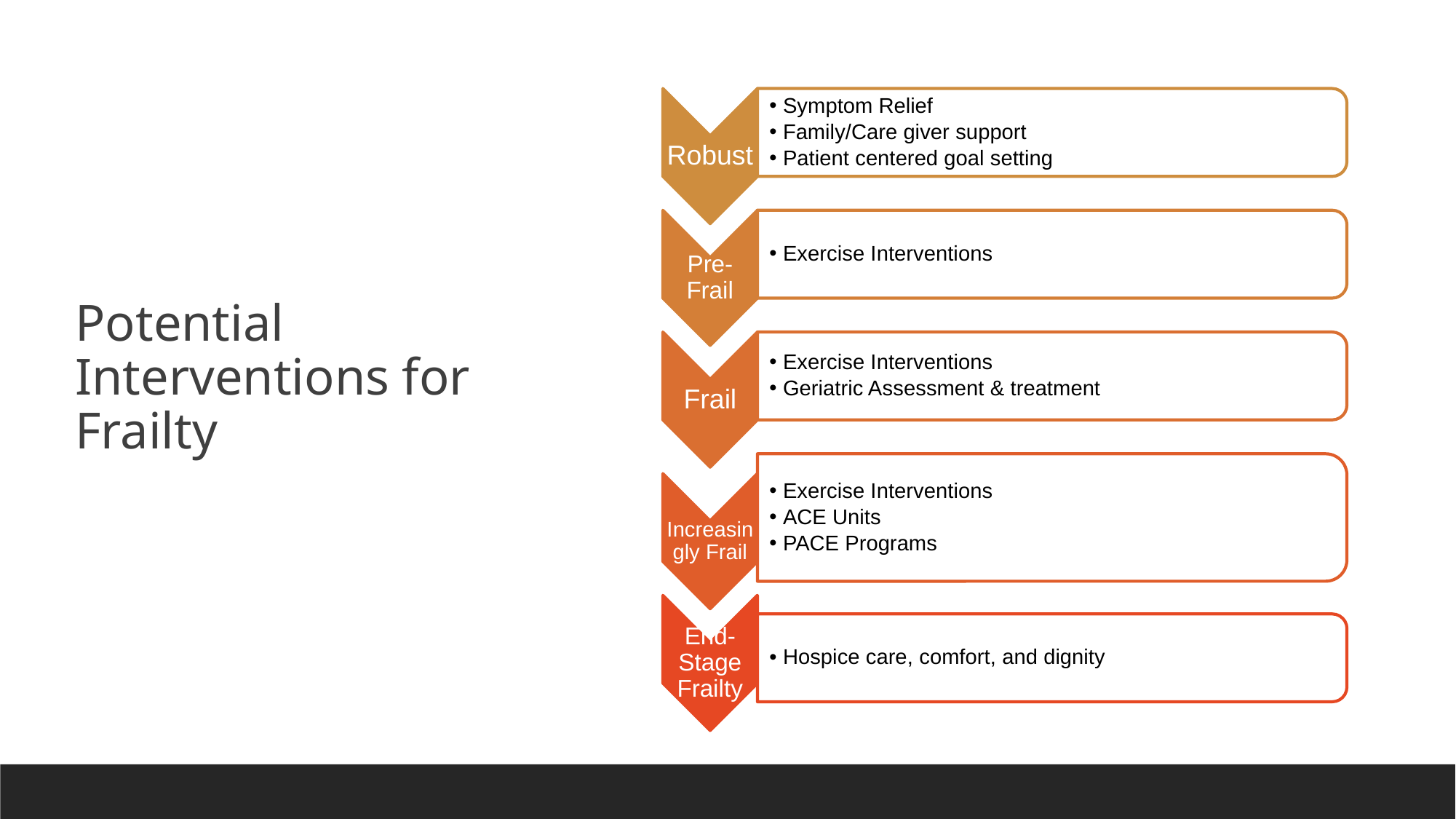

Potential Interventions for Frailty

## Slide 19
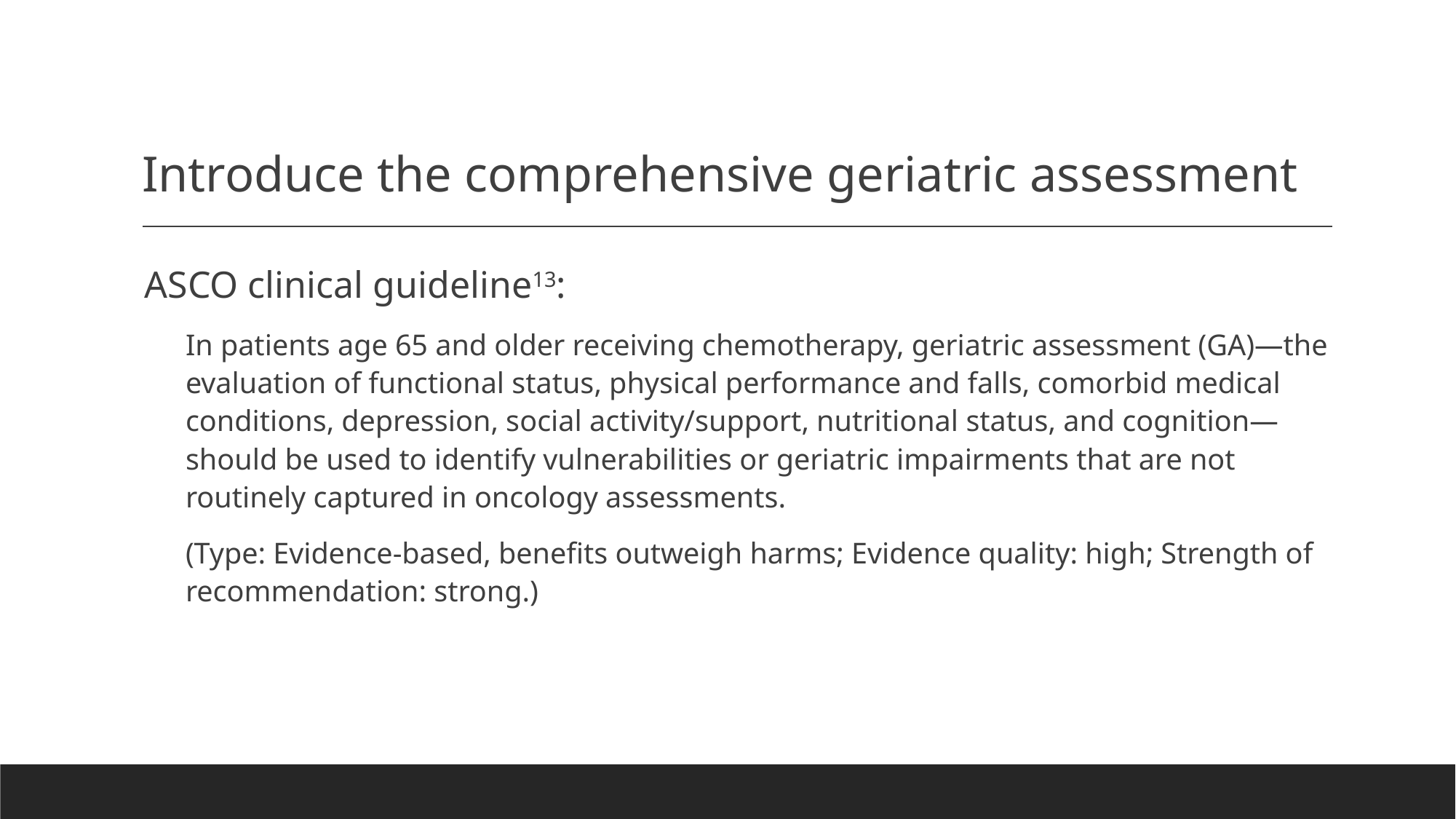

# Introduce the comprehensive geriatric assessment
ASCO clinical guideline13:
In patients age 65 and older receiving chemotherapy, geriatric assessment (GA)—the evaluation of functional status, physical performance and falls, comorbid medical conditions, depression, social activity/support, nutritional status, and cognition—should be used to identify vulnerabilities or geriatric impairments that are not routinely captured in oncology assessments.
(Type: Evidence-based, benefits outweigh harms; Evidence quality: high; Strength of recommendation: strong.)

## Slide 20
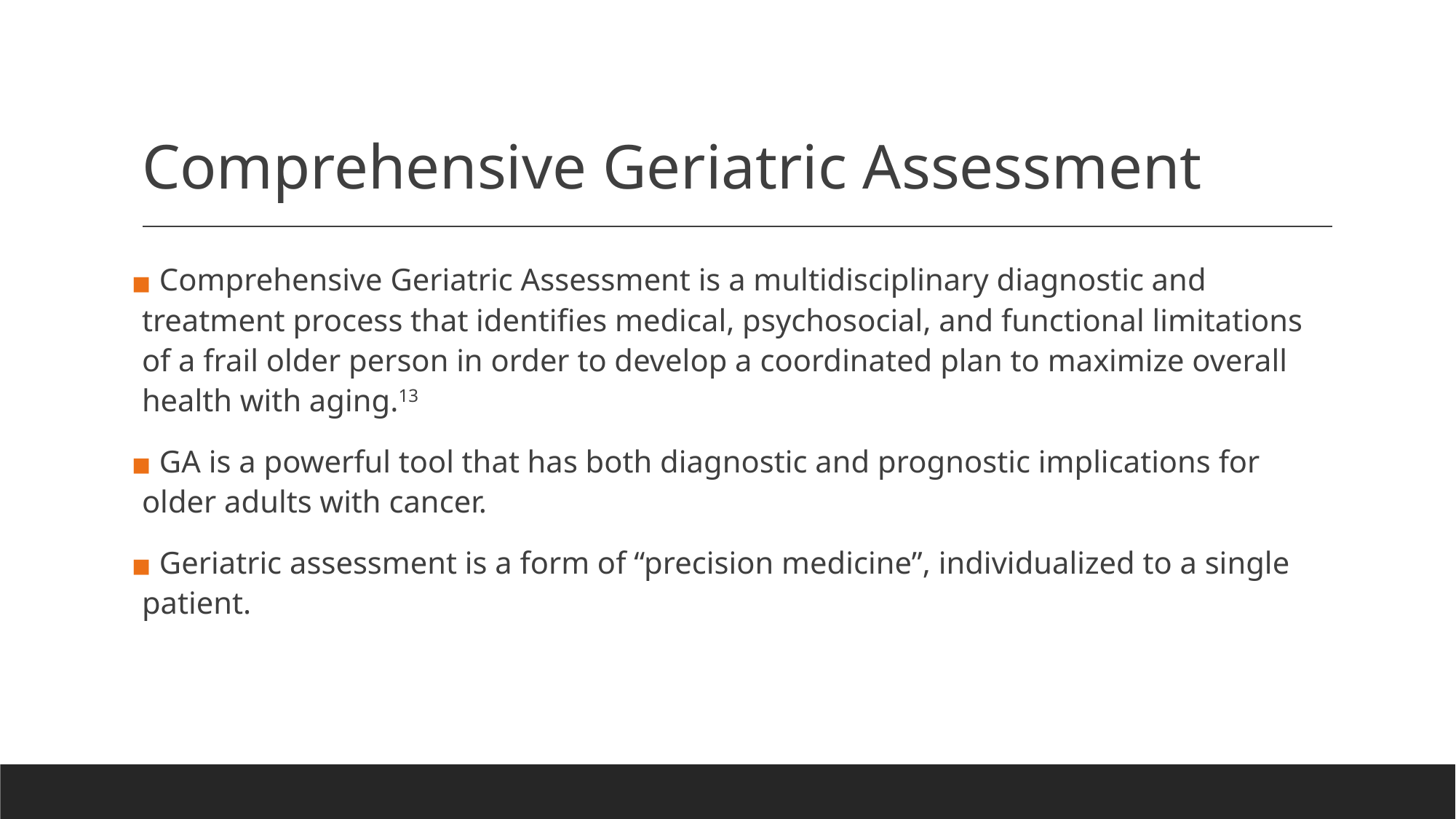

# Comprehensive Geriatric Assessment
 Comprehensive Geriatric Assessment is a multidisciplinary diagnostic and treatment process that identifies medical, psychosocial, and functional limitations of a frail older person in order to develop a coordinated plan to maximize overall health with aging.13
 GA is a powerful tool that has both diagnostic and prognostic implications for older adults with cancer.
 Geriatric assessment is a form of “precision medicine”, individualized to a single patient.

## Slide 21
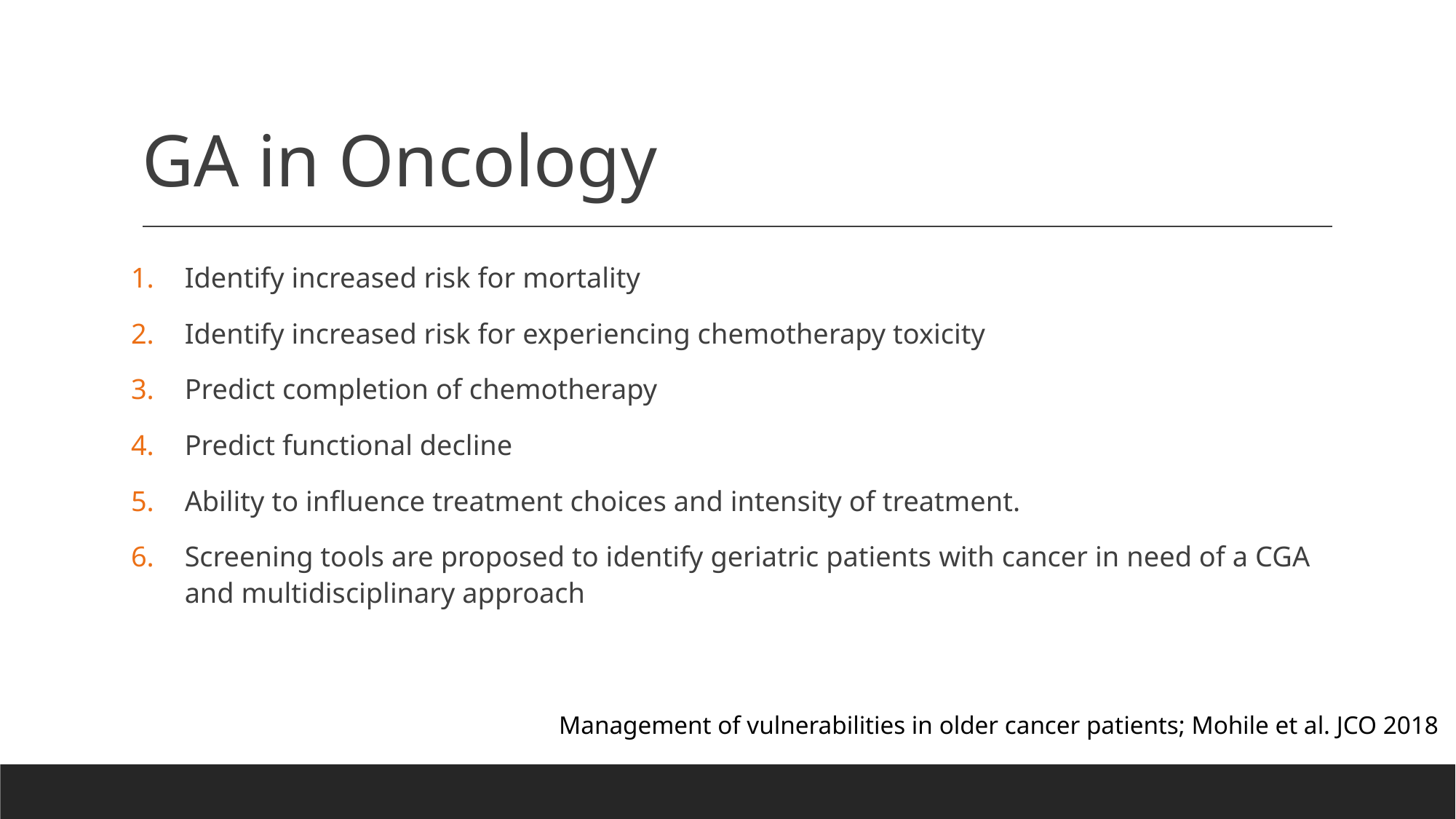

# GA in Oncology
Identify increased risk for mortality
Identify increased risk for experiencing chemotherapy toxicity
Predict completion of chemotherapy
Predict functional decline
Ability to influence treatment choices and intensity of treatment.
Screening tools are proposed to identify geriatric patients with cancer in need of a CGA and multidisciplinary approach
Management of vulnerabilities in older cancer patients; Mohile et al. JCO 2018

## Slide 22
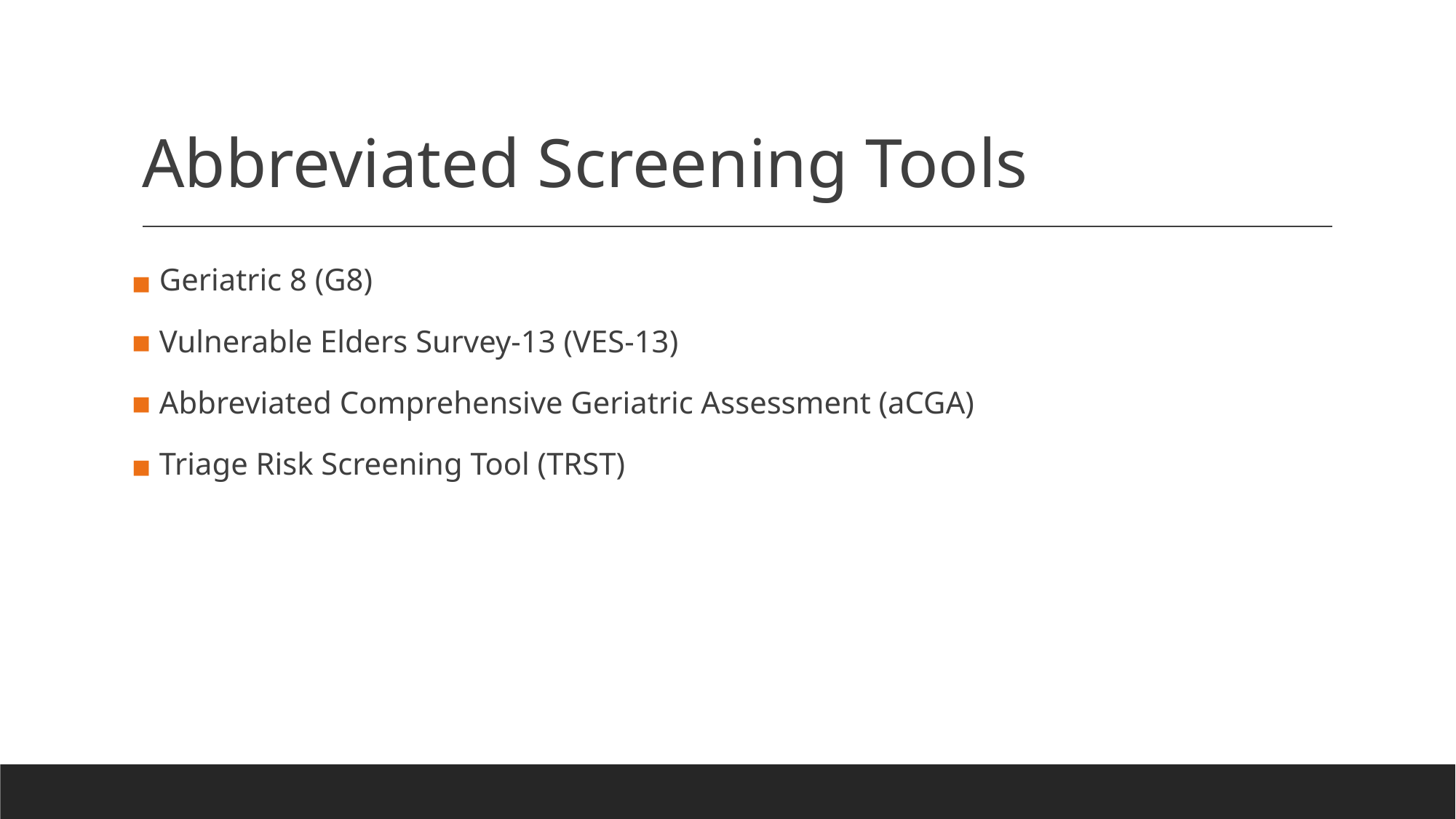

# Abbreviated Screening Tools
 Geriatric 8 (G8)
 Vulnerable Elders Survey-13 (VES-13)
 Abbreviated Comprehensive Geriatric Assessment (aCGA)
 Triage Risk Screening Tool (TRST)

## Slide 23
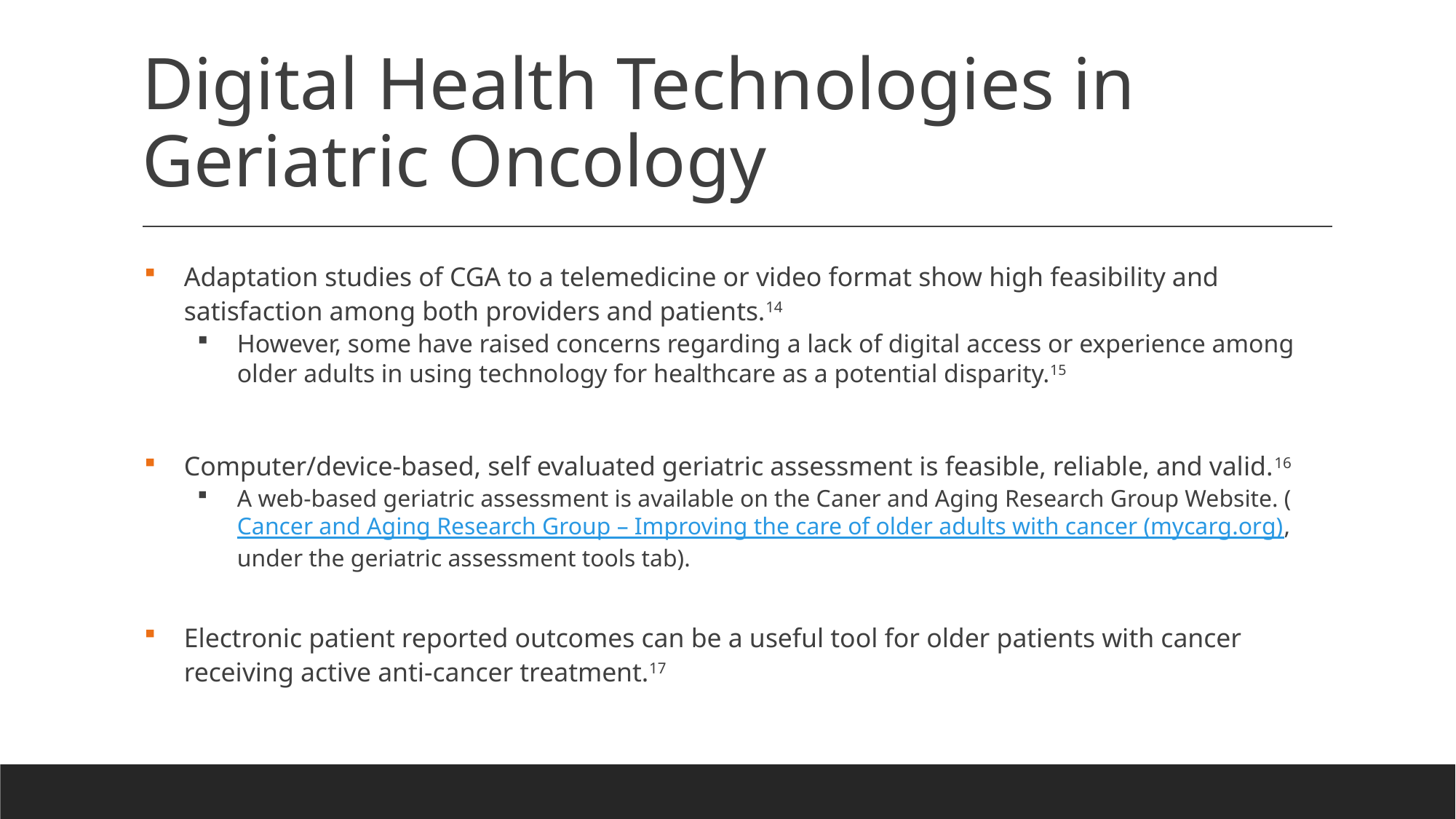

# Digital Health Technologies in Geriatric Oncology
Adaptation studies of CGA to a telemedicine or video format show high feasibility and satisfaction among both providers and patients.14
However, some have raised concerns regarding a lack of digital access or experience among older adults in using technology for healthcare as a potential disparity.15
Computer/device-based, self evaluated geriatric assessment is feasible, reliable, and valid.16
A web-based geriatric assessment is available on the Caner and Aging Research Group Website. (Cancer and Aging Research Group – Improving the care of older adults with cancer (mycarg.org), under the geriatric assessment tools tab).
Electronic patient reported outcomes can be a useful tool for older patients with cancer receiving active anti-cancer treatment.17

## Slide 24
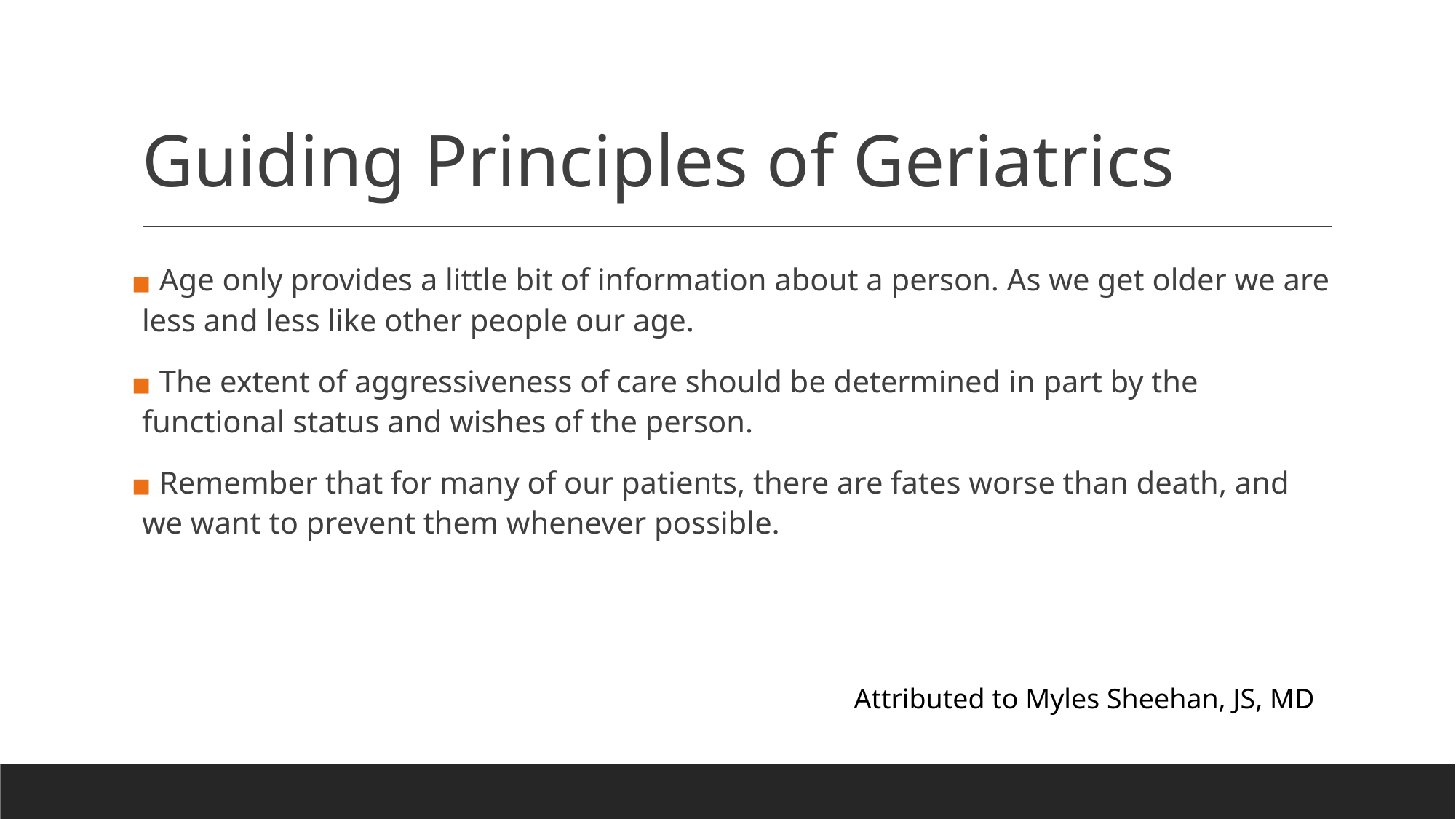

# Guiding Principles of Geriatrics
 Age only provides a little bit of information about a person. As we get older we are less and less like other people our age.
 The extent of aggressiveness of care should be determined in part by the functional status and wishes of the person.
 Remember that for many of our patients, there are fates worse than death, and we want to prevent them whenever possible.
Attributed to Myles Sheehan, JS, MD

## Slide 25
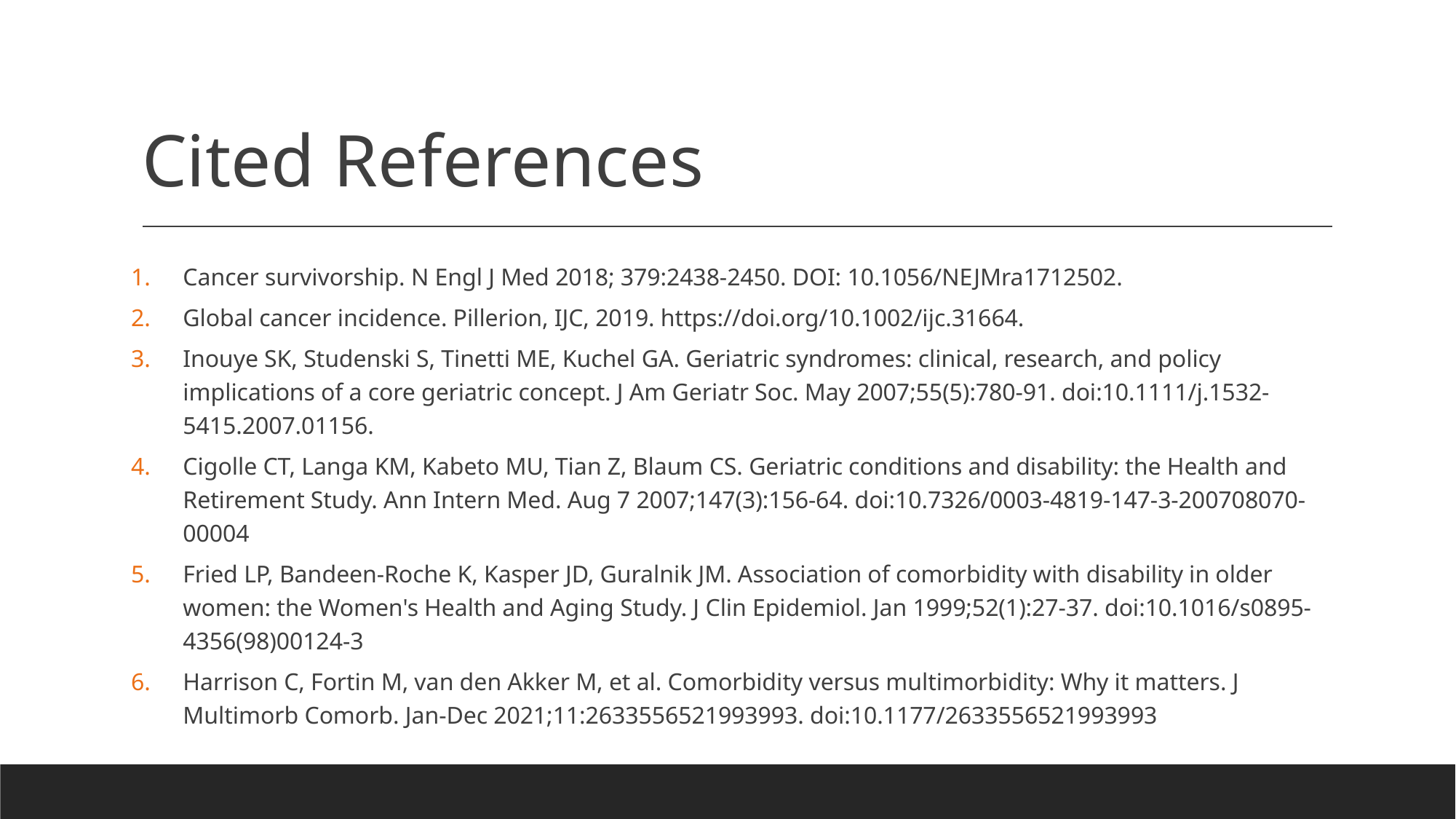

# Cited References
Cancer survivorship. N Engl J Med 2018; 379:2438-2450. DOI: 10.1056/NEJMra1712502.
Global cancer incidence. Pillerion, IJC, 2019. https://doi.org/10.1002/ijc.31664.
Inouye SK, Studenski S, Tinetti ME, Kuchel GA. Geriatric syndromes: clinical, research, and policy implications of a core geriatric concept. J Am Geriatr Soc. May 2007;55(5):780-91. doi:10.1111/j.1532-5415.2007.01156.
Cigolle CT, Langa KM, Kabeto MU, Tian Z, Blaum CS. Geriatric conditions and disability: the Health and Retirement Study. Ann Intern Med. Aug 7 2007;147(3):156-64. doi:10.7326/0003-4819-147-3-200708070-00004
Fried LP, Bandeen-Roche K, Kasper JD, Guralnik JM. Association of comorbidity with disability in older women: the Women's Health and Aging Study. J Clin Epidemiol. Jan 1999;52(1):27-37. doi:10.1016/s0895-4356(98)00124-3
Harrison C, Fortin M, van den Akker M, et al. Comorbidity versus multimorbidity: Why it matters. J Multimorb Comorb. Jan-Dec 2021;11:2633556521993993. doi:10.1177/2633556521993993

## Slide 26
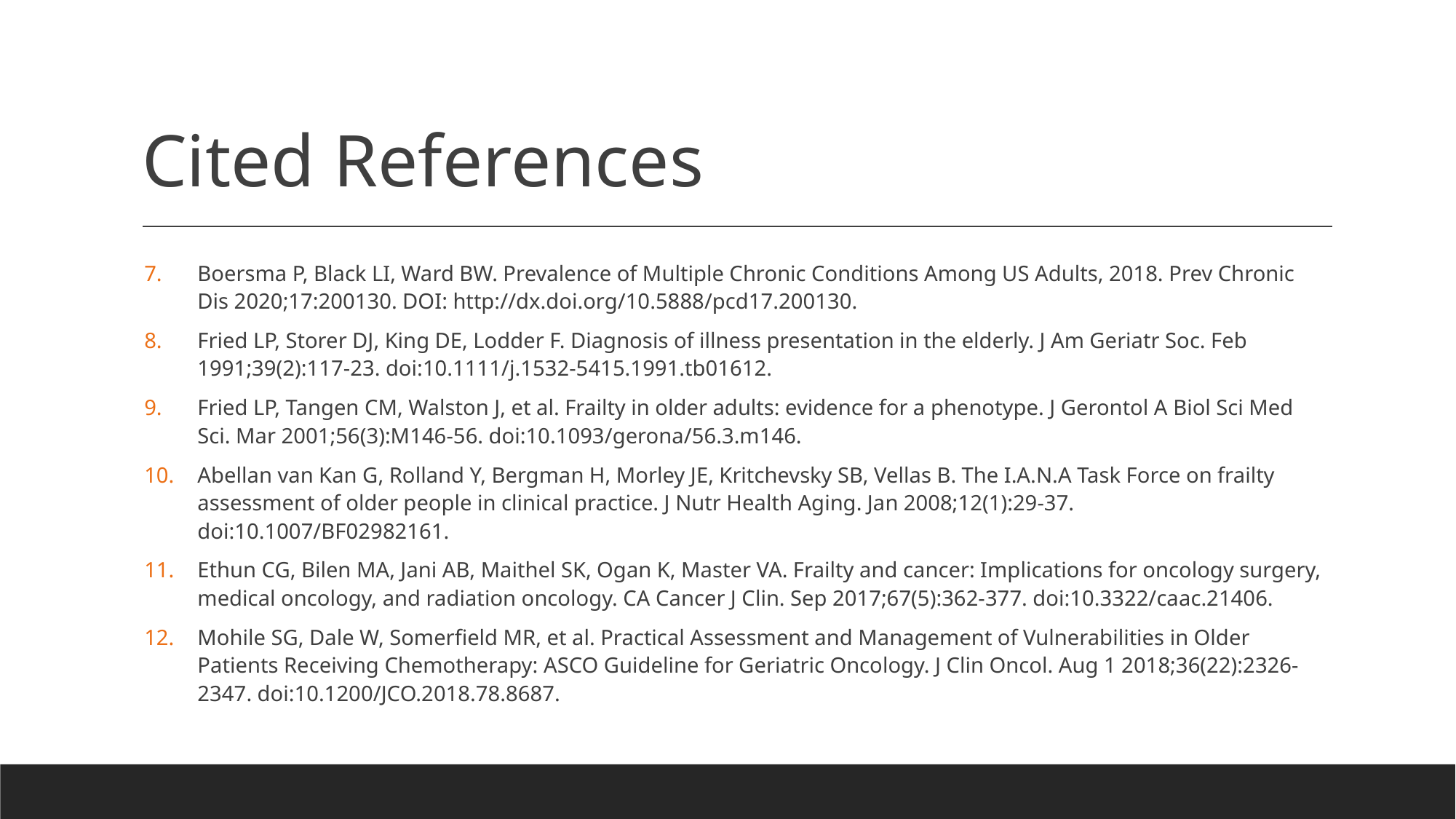

# Cited References
Boersma P, Black LI, Ward BW. Prevalence of Multiple Chronic Conditions Among US Adults, 2018. Prev Chronic Dis 2020;17:200130. DOI: http://dx.doi.org/10.5888/pcd17.200130.
Fried LP, Storer DJ, King DE, Lodder F. Diagnosis of illness presentation in the elderly. J Am Geriatr Soc. Feb 1991;39(2):117-23. doi:10.1111/j.1532-5415.1991.tb01612.
Fried LP, Tangen CM, Walston J, et al. Frailty in older adults: evidence for a phenotype. J Gerontol A Biol Sci Med Sci. Mar 2001;56(3):M146-56. doi:10.1093/gerona/56.3.m146.
Abellan van Kan G, Rolland Y, Bergman H, Morley JE, Kritchevsky SB, Vellas B. The I.A.N.A Task Force on frailty assessment of older people in clinical practice. J Nutr Health Aging. Jan 2008;12(1):29-37. doi:10.1007/BF02982161.
Ethun CG, Bilen MA, Jani AB, Maithel SK, Ogan K, Master VA. Frailty and cancer: Implications for oncology surgery, medical oncology, and radiation oncology. CA Cancer J Clin. Sep 2017;67(5):362-377. doi:10.3322/caac.21406.
Mohile SG, Dale W, Somerfield MR, et al. Practical Assessment and Management of Vulnerabilities in Older Patients Receiving Chemotherapy: ASCO Guideline for Geriatric Oncology. J Clin Oncol. Aug 1 2018;36(22):2326-2347. doi:10.1200/JCO.2018.78.8687.

## Slide 27
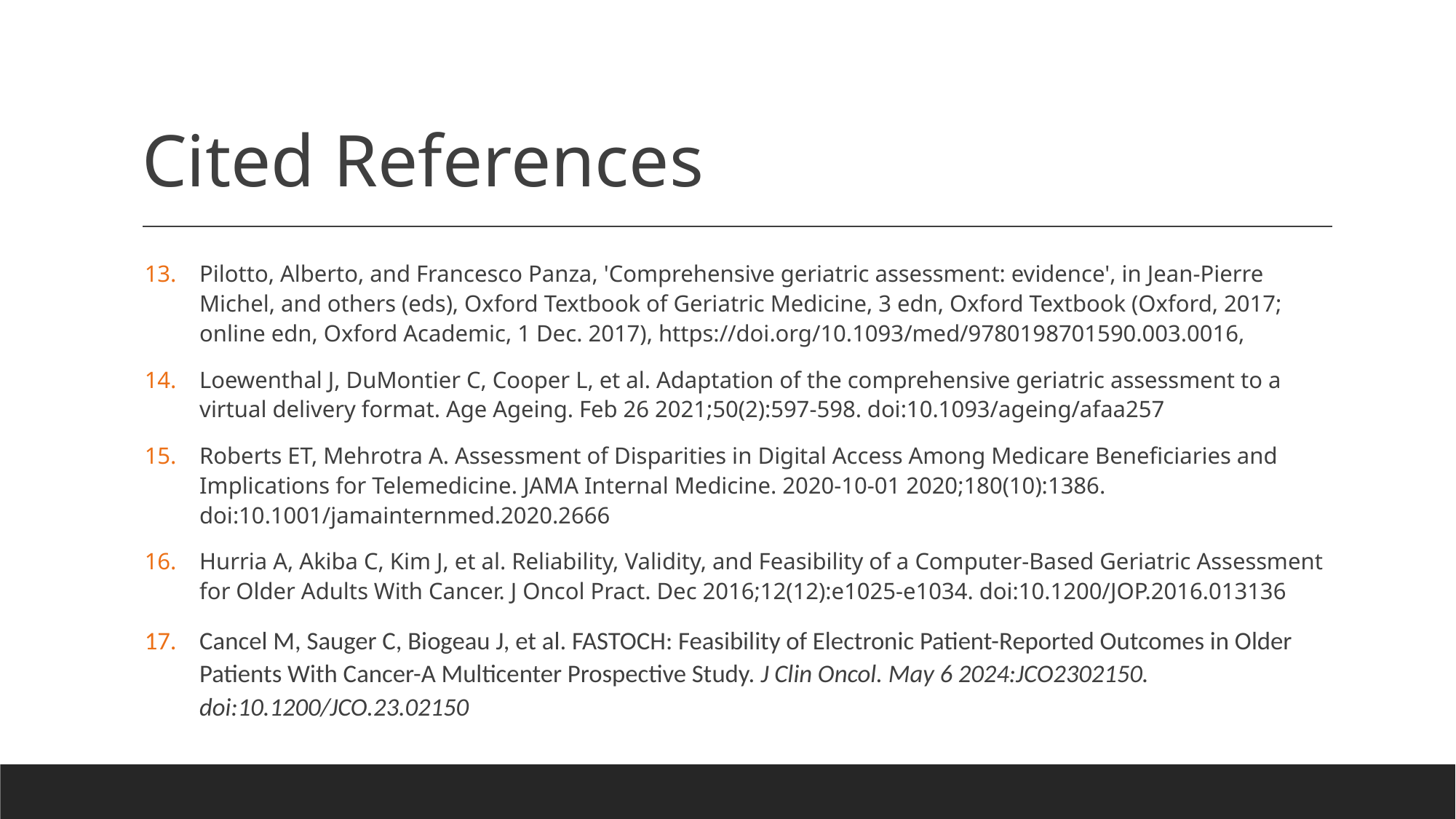

# Cited References
Pilotto, Alberto, and Francesco Panza, 'Comprehensive geriatric assessment: evidence', in Jean-Pierre Michel, and others (eds), Oxford Textbook of Geriatric Medicine, 3 edn, Oxford Textbook (Oxford, 2017; online edn, Oxford Academic, 1 Dec. 2017), https://doi.org/10.1093/med/9780198701590.003.0016,
Loewenthal J, DuMontier C, Cooper L, et al. Adaptation of the comprehensive geriatric assessment to a virtual delivery format. Age Ageing. Feb 26 2021;50(2):597-598. doi:10.1093/ageing/afaa257
Roberts ET, Mehrotra A. Assessment of Disparities in Digital Access Among Medicare Beneficiaries and Implications for Telemedicine. JAMA Internal Medicine. 2020-10-01 2020;180(10):1386. doi:10.1001/jamainternmed.2020.2666
Hurria A, Akiba C, Kim J, et al. Reliability, Validity, and Feasibility of a Computer-Based Geriatric Assessment for Older Adults With Cancer. J Oncol Pract. Dec 2016;12(12):e1025-e1034. doi:10.1200/JOP.2016.013136
Cancel M, Sauger C, Biogeau J, et al. FASTOCH: Feasibility of Electronic Patient-Reported Outcomes in Older Patients With Cancer-A Multicenter Prospective Study. J Clin Oncol. May 6 2024:JCO2302150. doi:10.1200/JCO.23.02150
