# Supplementary material for: An Introductory Course on Geriatric Oncology
Source: MedEdPORTAL. 2024 Nov 14;20:11471. doi: 10.15766/mep_2374-8265.11471 (PMC11561070; doi:10.15766/mep_2374-8265.11471)
Supplement: Supplementary file 1 — Introduction to Geriatric Oncology.pptxThe Comprehensive Geriatric Assessment.pptxGeriatric Screening Tools.pptxBiology of Aging.pptxCancer Therapy in the Older Adult.pptxSummary of Interactive Sessions.docxSession 5 Patient Case 1.docxSession 5 Patient Case 2.docxSession 5 Patient Case 3.docxGeriatric Oncology Knowledge Assessment.docxKnowledge Assessment Answer Key.docxSelf-Perceived Competency Assessment.docxCurriculum Session Assessment.docx [file mep_2374-8265.11471-s001.zip › B. The Comprehensive Geriatric Assessment.pptx]

## Slide 1
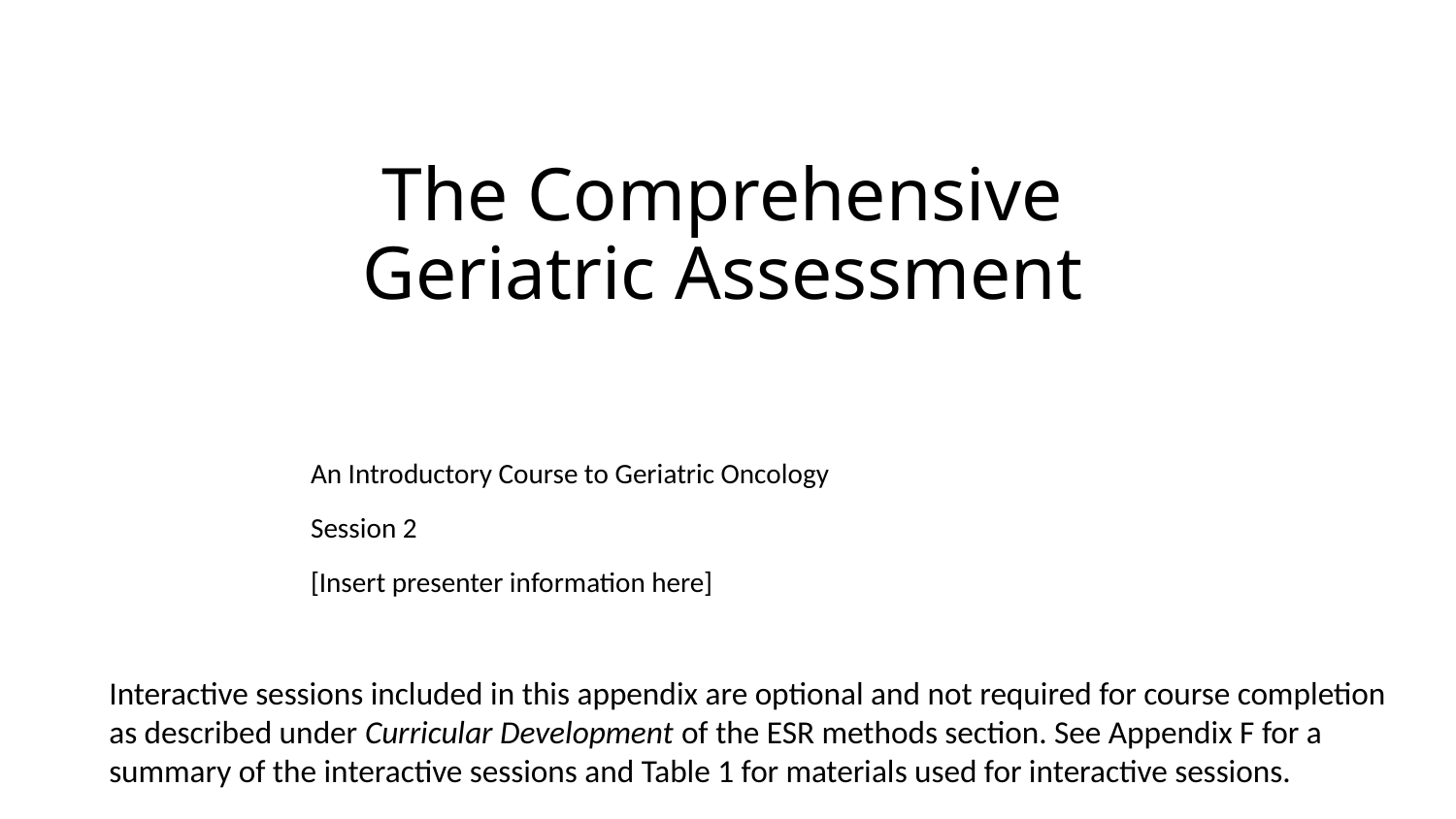

# The Comprehensive Geriatric Assessment
An Introductory Course to Geriatric Oncology
Session 2
[Insert presenter information here]
Interactive sessions included in this appendix are optional and not required for course completion as described under Curricular Development of the ESR methods section. See Appendix F for a summary of the interactive sessions and Table 1 for materials used for interactive sessions.

## Slide 2
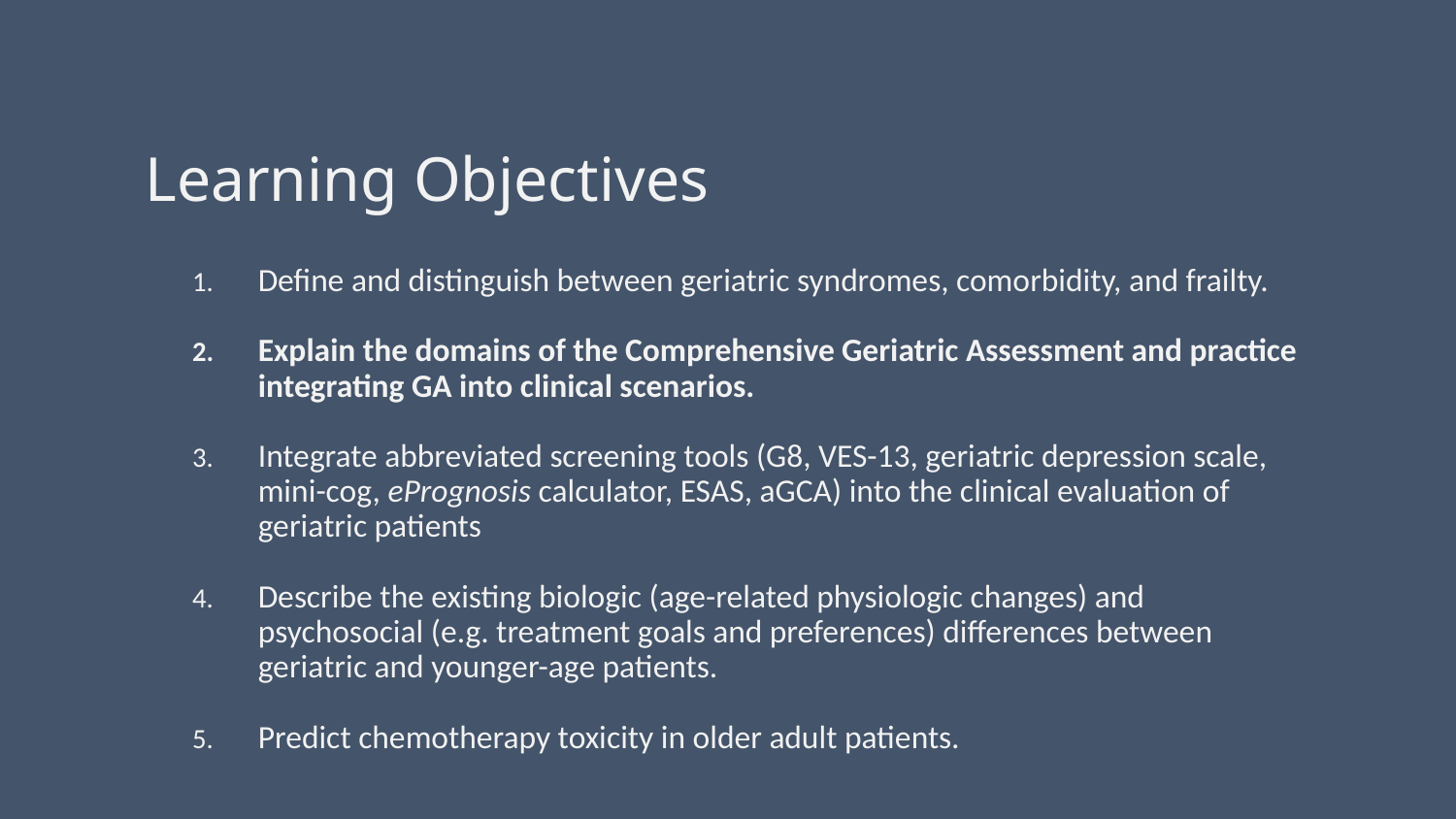

# Learning Objectives
Define and distinguish between geriatric syndromes, comorbidity, and frailty. ​
Explain the domains of the Comprehensive Geriatric Assessment and practice integrating GA into clinical scenarios.
Integrate abbreviated screening tools (G8, VES-13, geriatric depression scale, mini-cog, ePrognosis calculator, ESAS, aGCA) into the clinical evaluation of geriatric patients
Describe the existing biologic (age-related physiologic changes) and psychosocial (e.g. treatment goals and preferences) differences between geriatric and younger-age patients. ​​
Predict chemotherapy toxicity in older adult patients.​

## Slide 3
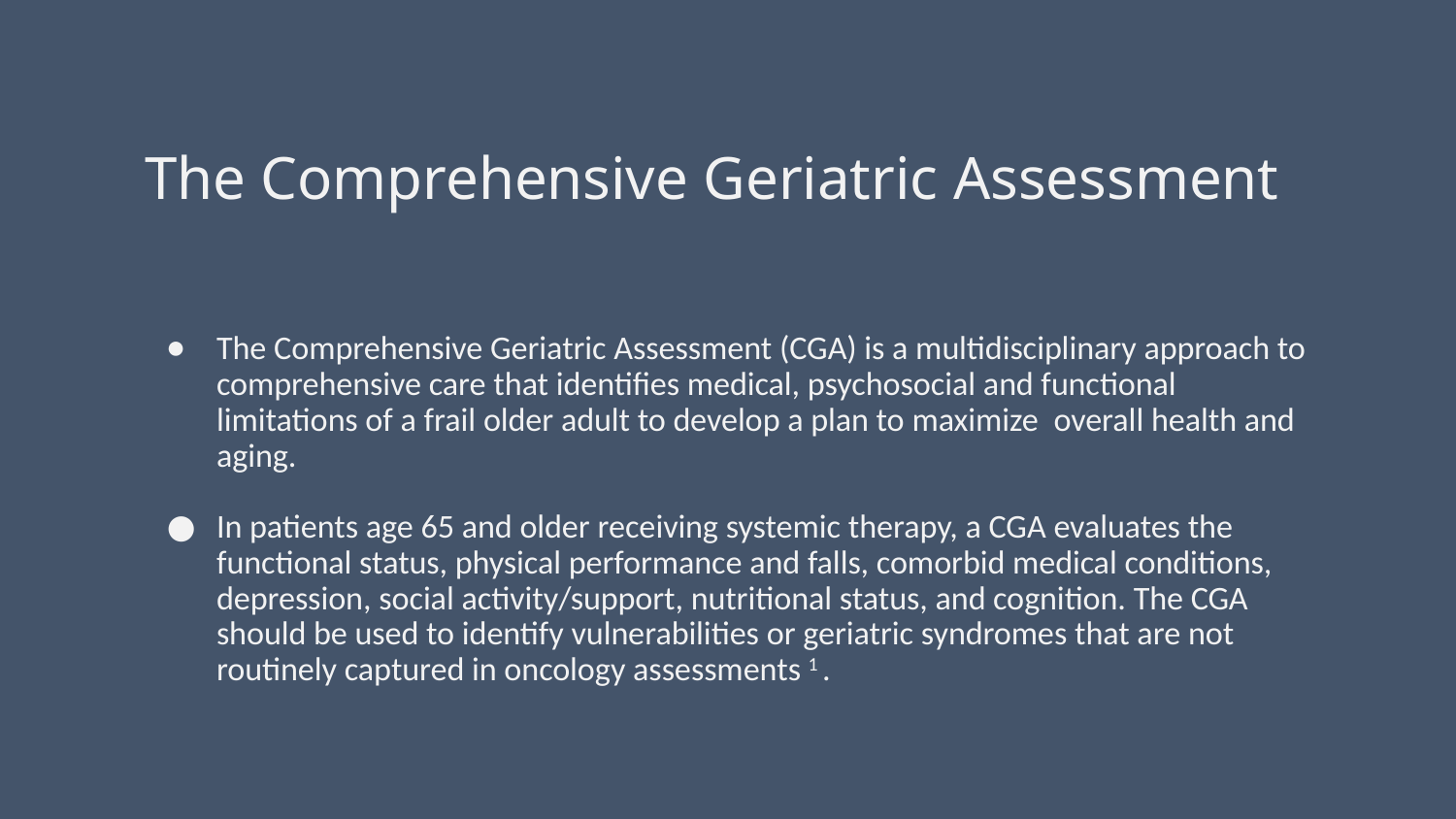

# The Comprehensive Geriatric Assessment
The Comprehensive Geriatric Assessment (CGA) is a multidisciplinary approach to comprehensive care that identifies medical, psychosocial and functional limitations of a frail older adult to develop a plan to maximize overall health and aging.
In patients age 65 and older receiving systemic therapy, a CGA evaluates the functional status, physical performance and falls, comorbid medical conditions, depression, social activity/support, nutritional status, and cognition. The CGA should be used to identify vulnerabilities or geriatric syndromes that are not routinely captured in oncology assessments 1 .

## Slide 4
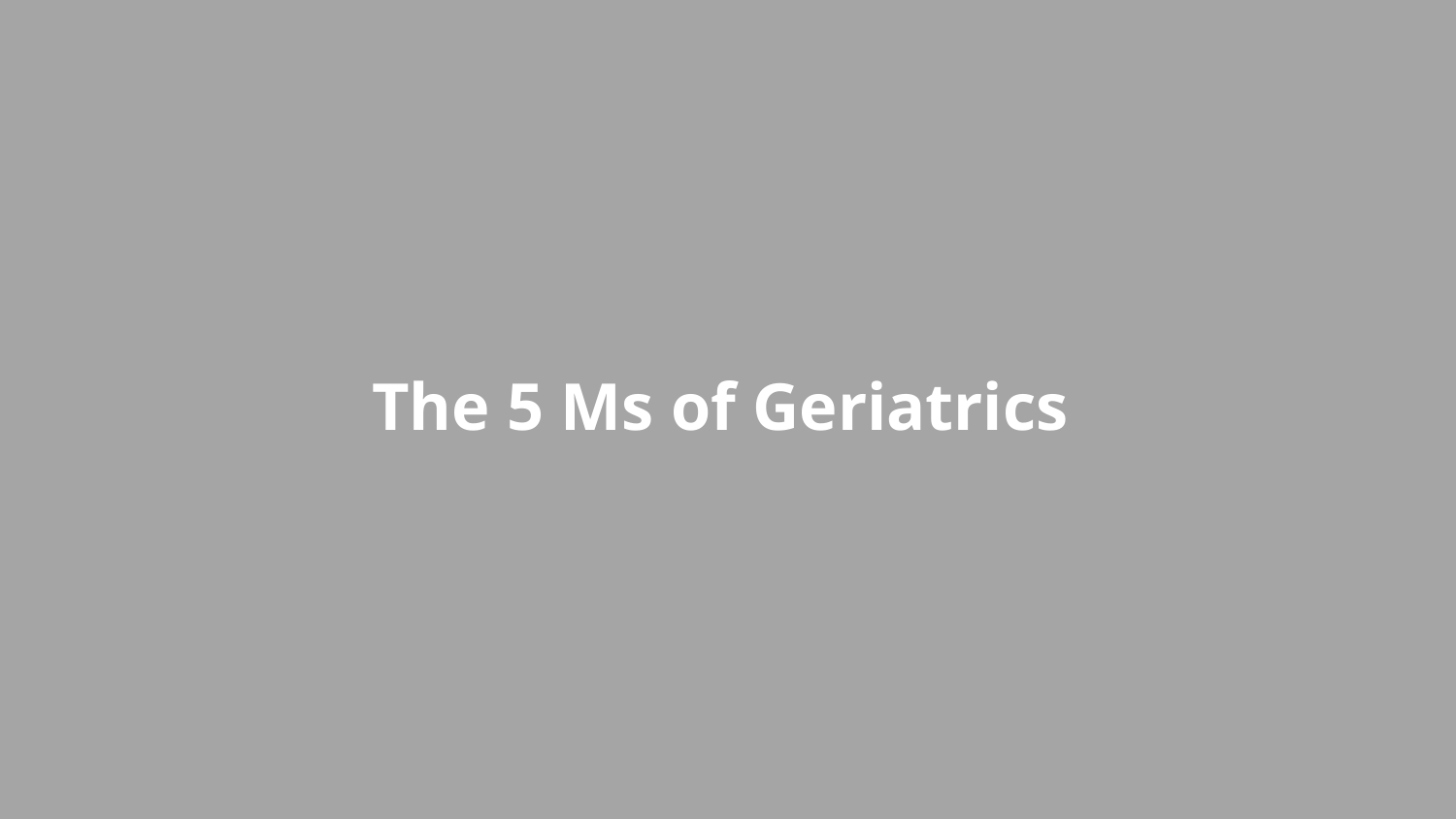

# The 5 Ms of Geriatrics

## Slide 5
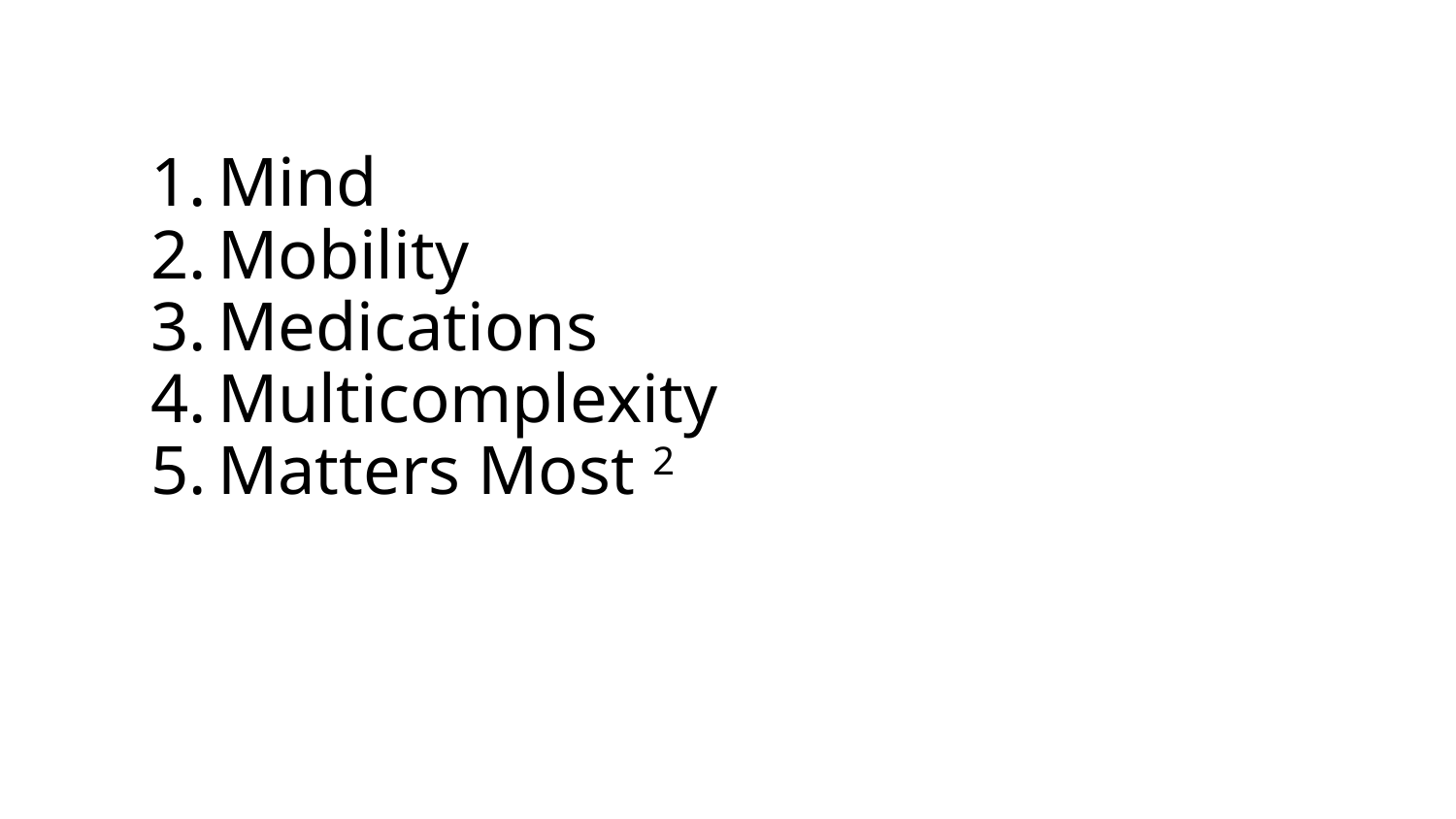

# Mind
Mobility
Medications
Multicomplexity
Matters Most 2

## Slide 6
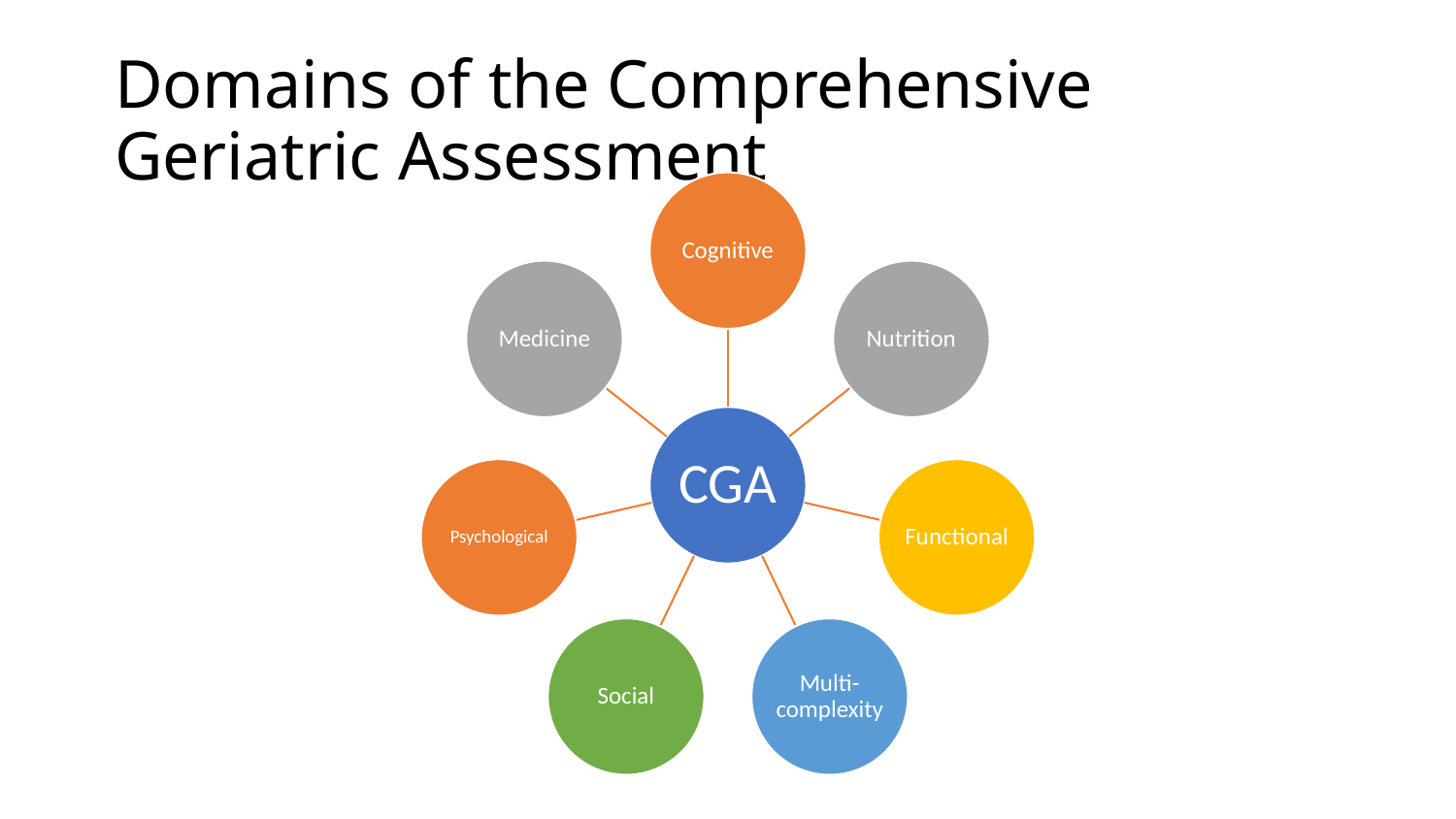

# Domains of the Comprehensive Geriatric Assessment

## Slide 7
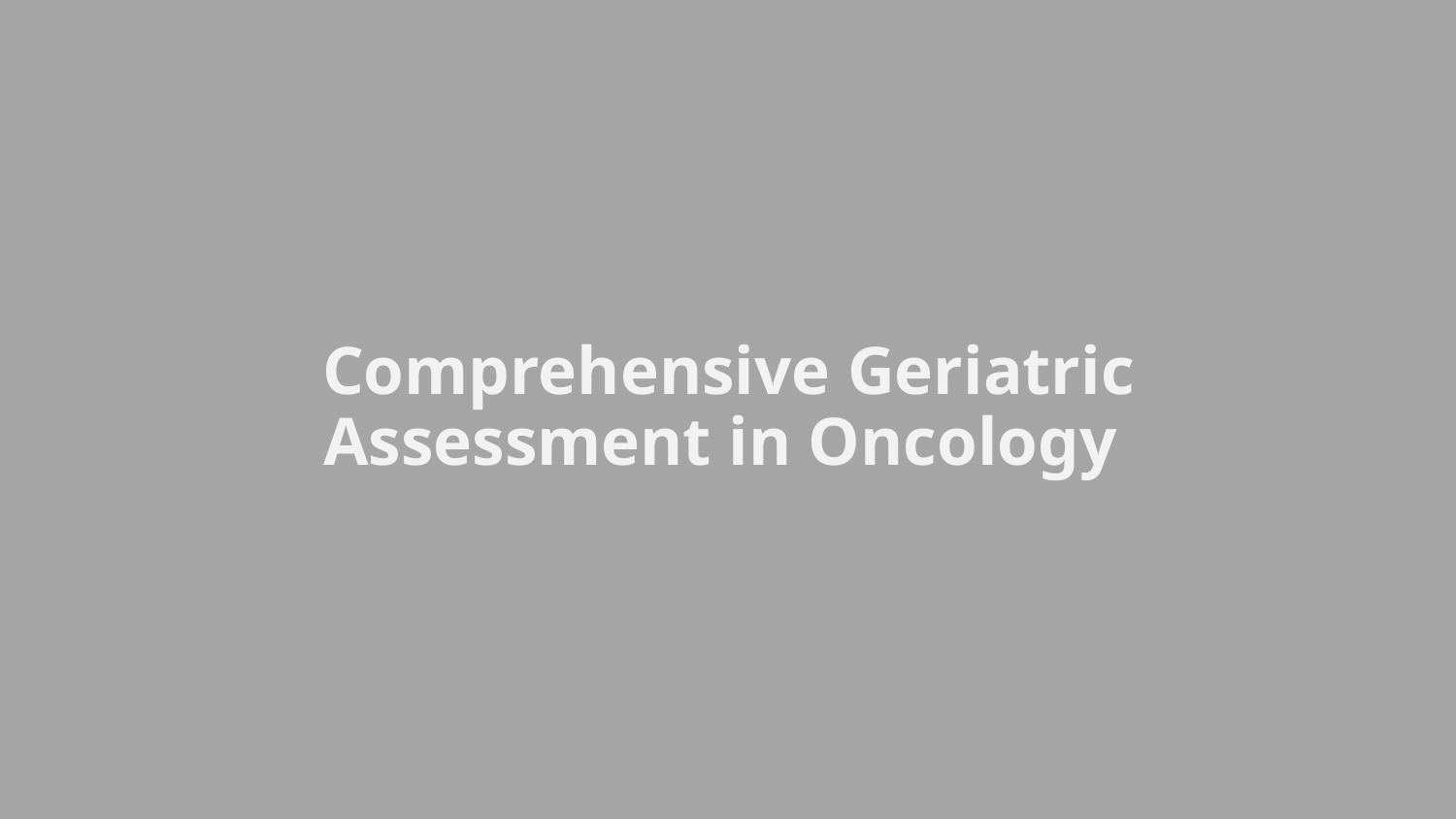

# Comprehensive Geriatric Assessment in Oncology

## Slide 8
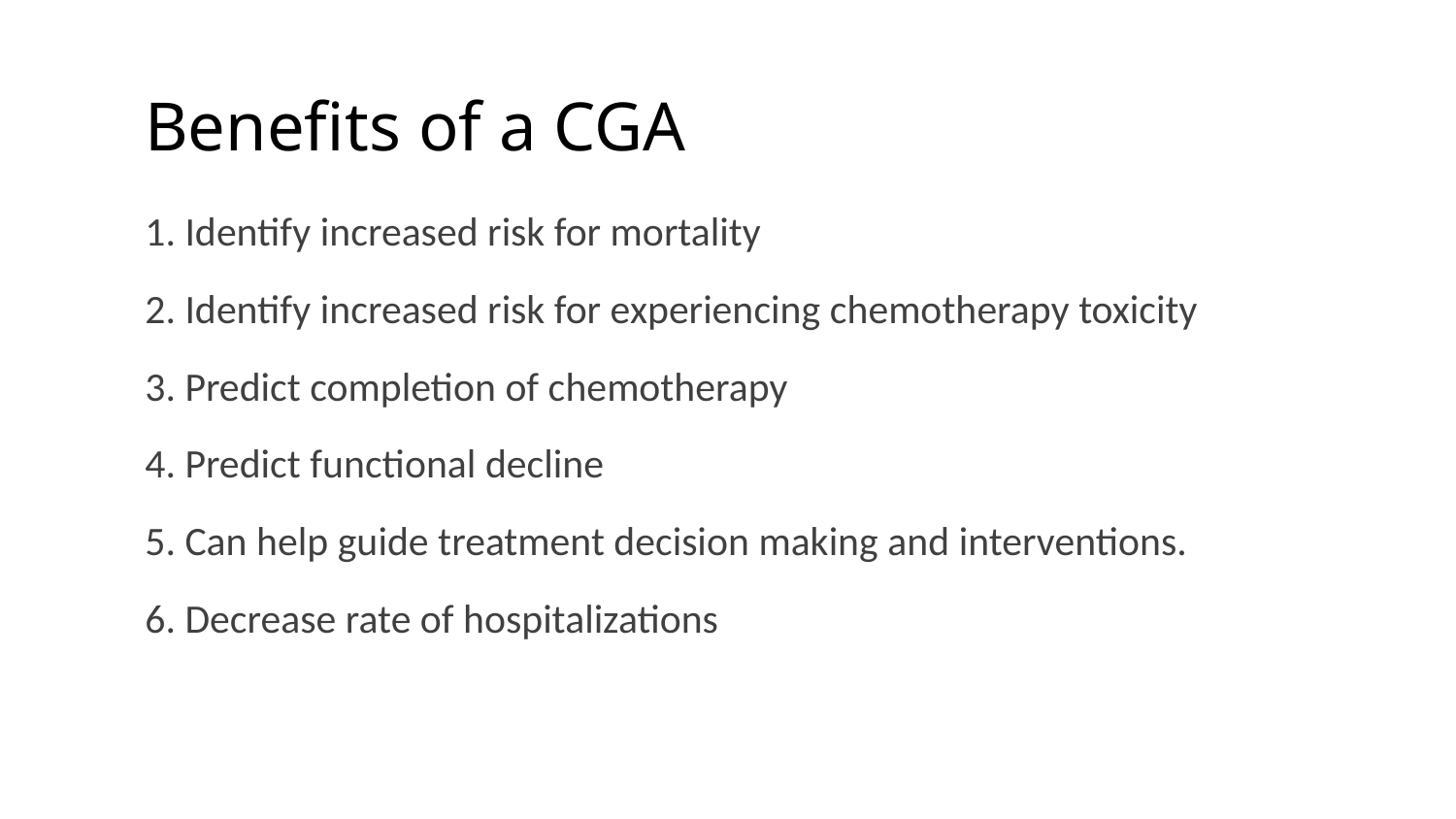

# Benefits of a CGA
1. Identify increased risk for mortality
2. Identify increased risk for experiencing chemotherapy toxicity
3. Predict completion of chemotherapy
4. Predict functional decline
5. Can help guide treatment decision making and interventions.
6. Decrease rate of hospitalizations

## Slide 9
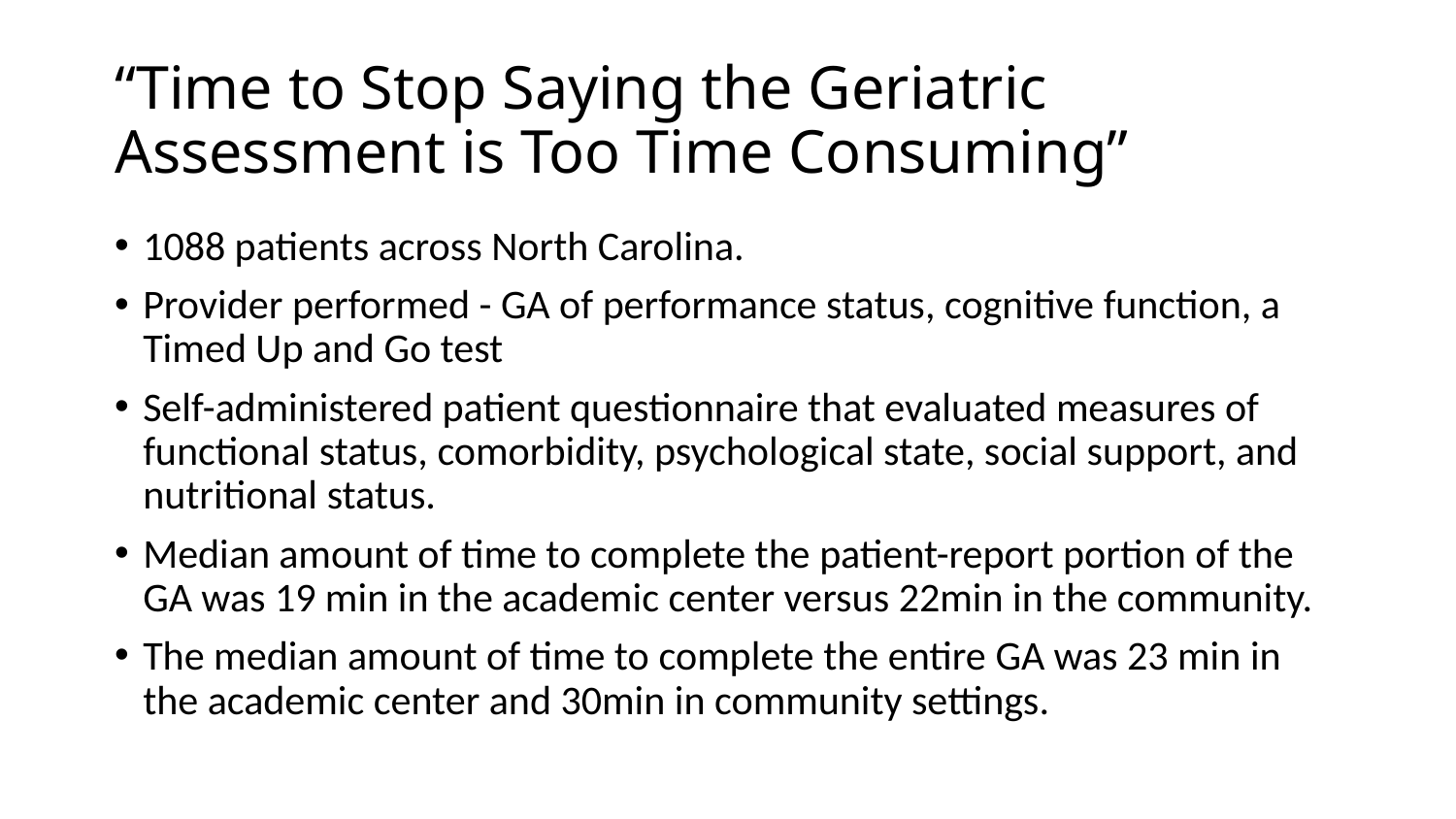

# “Time to Stop Saying the Geriatric Assessment is Too Time Consuming”
1088 patients across North Carolina.
Provider performed - GA of performance status, cognitive function, a Timed Up and Go test
Self-administered patient questionnaire that evaluated measures of functional status, comorbidity, psychological state, social support, and nutritional status.
Median amount of time to complete the patient-report portion of the GA was 19 min in the academic center versus 22min in the community.
The median amount of time to complete the entire GA was 23 min in the academic center and 30min in community settings.

## Slide 10
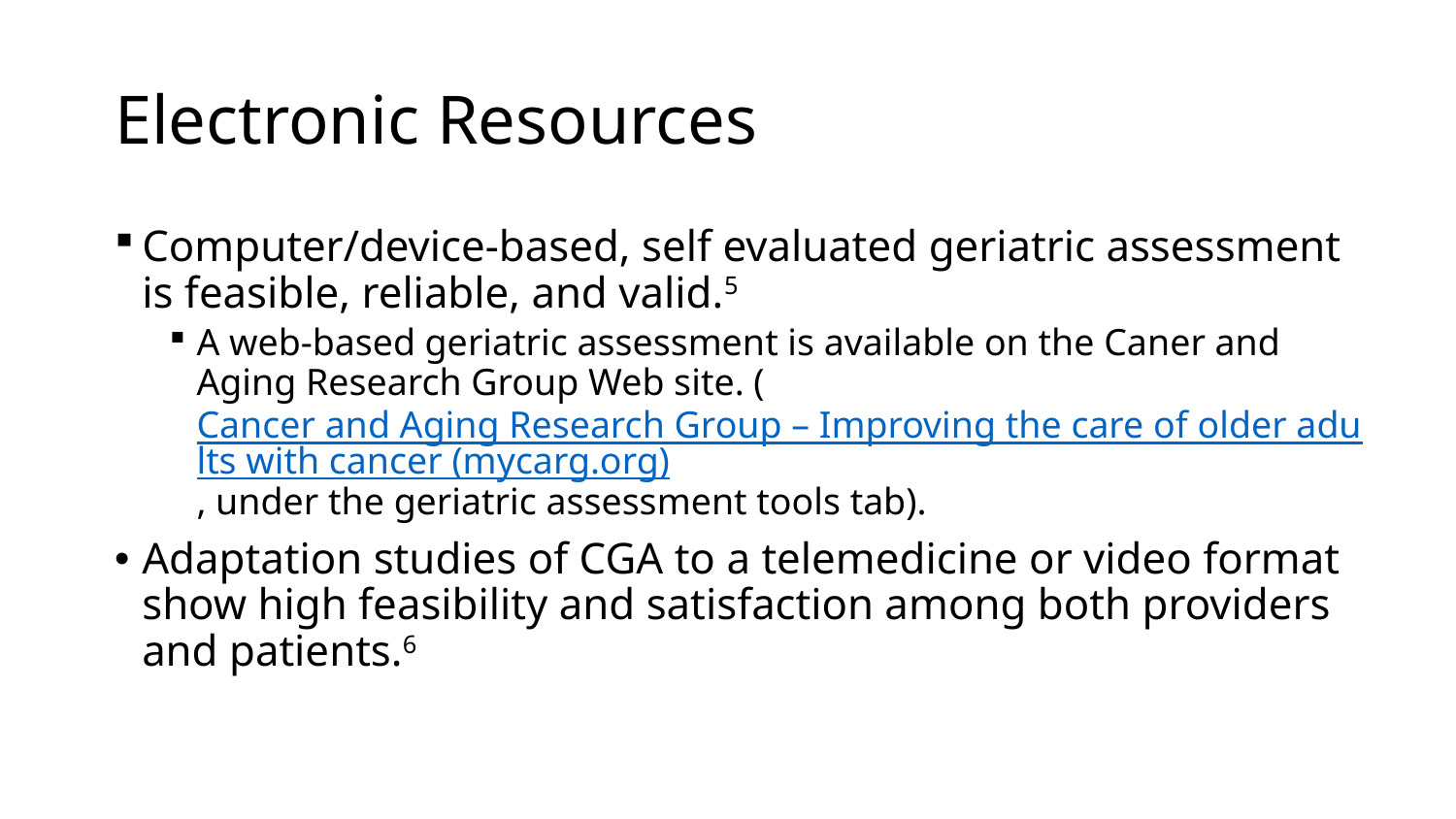

# Electronic Resources
Computer/device-based, self evaluated geriatric assessment is feasible, reliable, and valid.5
A web-based geriatric assessment is available on the Caner and Aging Research Group Web site. (Cancer and Aging Research Group – Improving the care of older adults with cancer (mycarg.org), under the geriatric assessment tools tab).
Adaptation studies of CGA to a telemedicine or video format show high feasibility and satisfaction among both providers and patients.6

## Slide 11
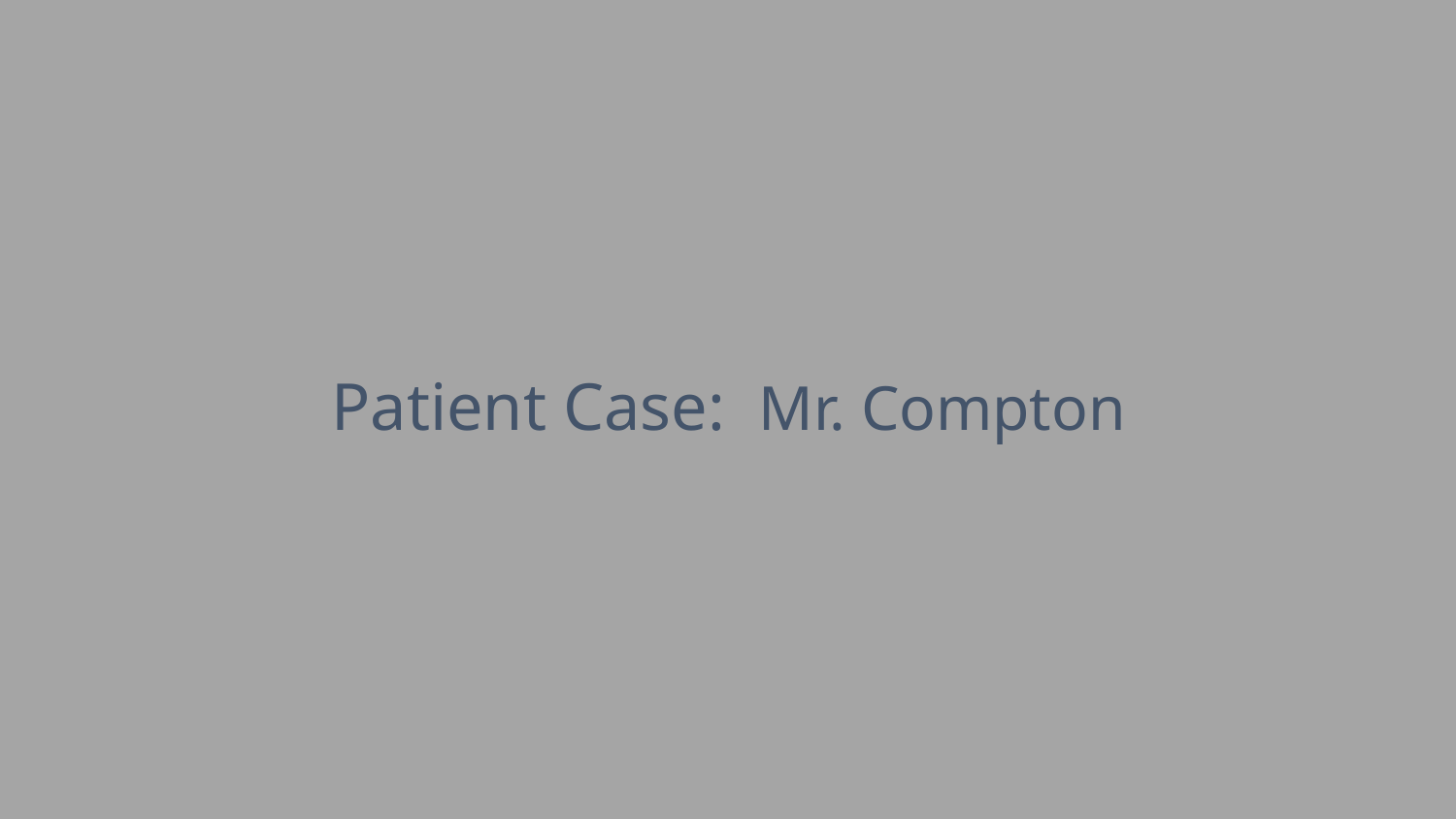

# Patient Case: Mr. Compton

## Slide 12
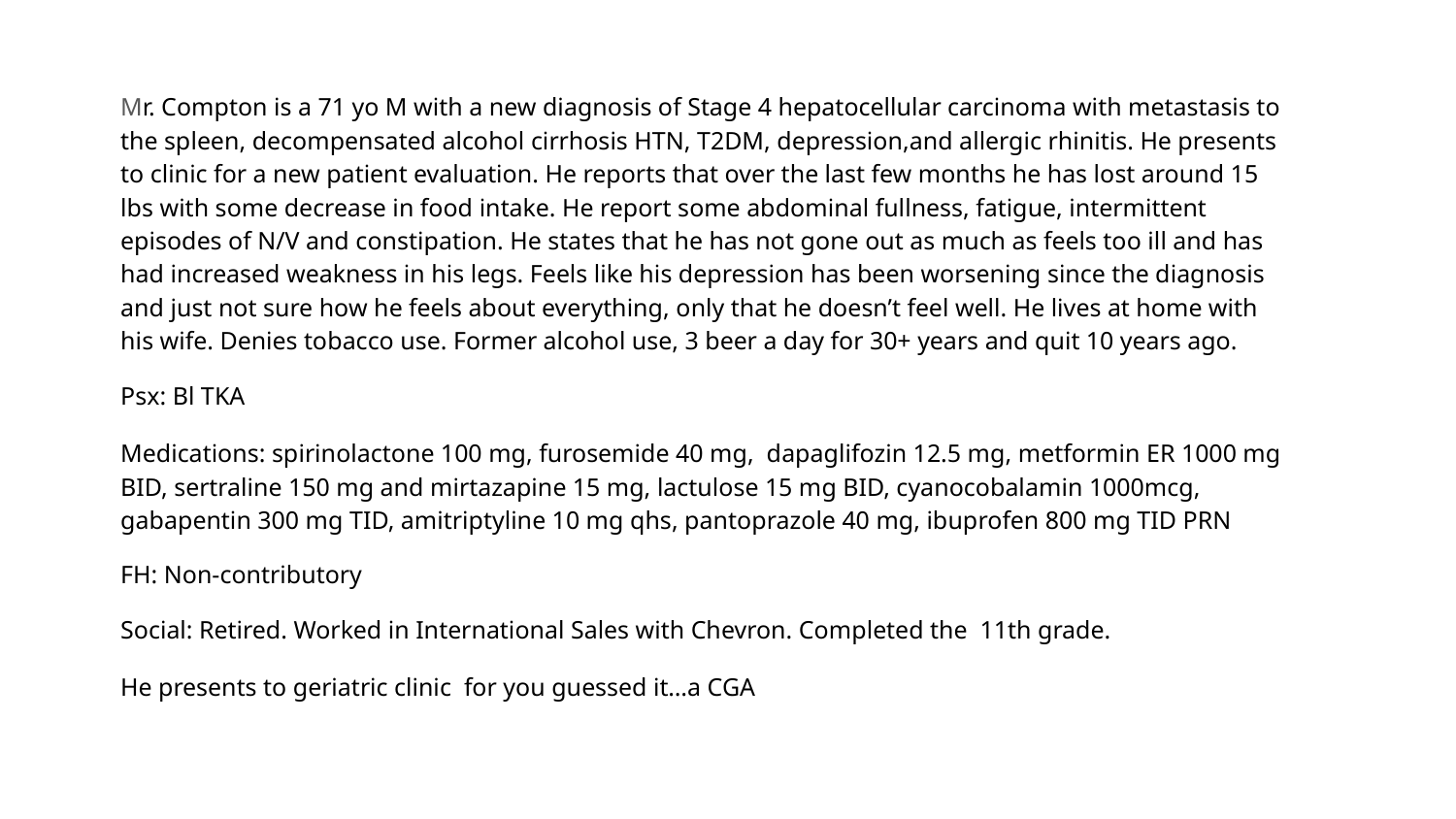

# Mr. Compton is a 71 yo M with a new diagnosis of Stage 4 hepatocellular carcinoma with metastasis to the spleen, decompensated alcohol cirrhosis HTN, T2DM, depression,and allergic rhinitis. He presents to clinic for a new patient evaluation. He reports that over the last few months he has lost around 15 lbs with some decrease in food intake. He report some abdominal fullness, fatigue, intermittent episodes of N/V and constipation. He states that he has not gone out as much as feels too ill and has had increased weakness in his legs. Feels like his depression has been worsening since the diagnosis and just not sure how he feels about everything, only that he doesn’t feel well. He lives at home with his wife. Denies tobacco use. Former alcohol use, 3 beer a day for 30+ years and quit 10 years ago.
Psx: Bl TKA
Medications: spirinolactone 100 mg, furosemide 40 mg, dapaglifozin 12.5 mg, metformin ER 1000 mg BID, sertraline 150 mg and mirtazapine 15 mg, lactulose 15 mg BID, cyanocobalamin 1000mcg, gabapentin 300 mg TID, amitriptyline 10 mg qhs, pantoprazole 40 mg, ibuprofen 800 mg TID PRN
FH: Non-contributory
Social: Retired. Worked in International Sales with Chevron. Completed the 11th grade.
He presents to geriatric clinic for you guessed it…a CGA

## Slide 13
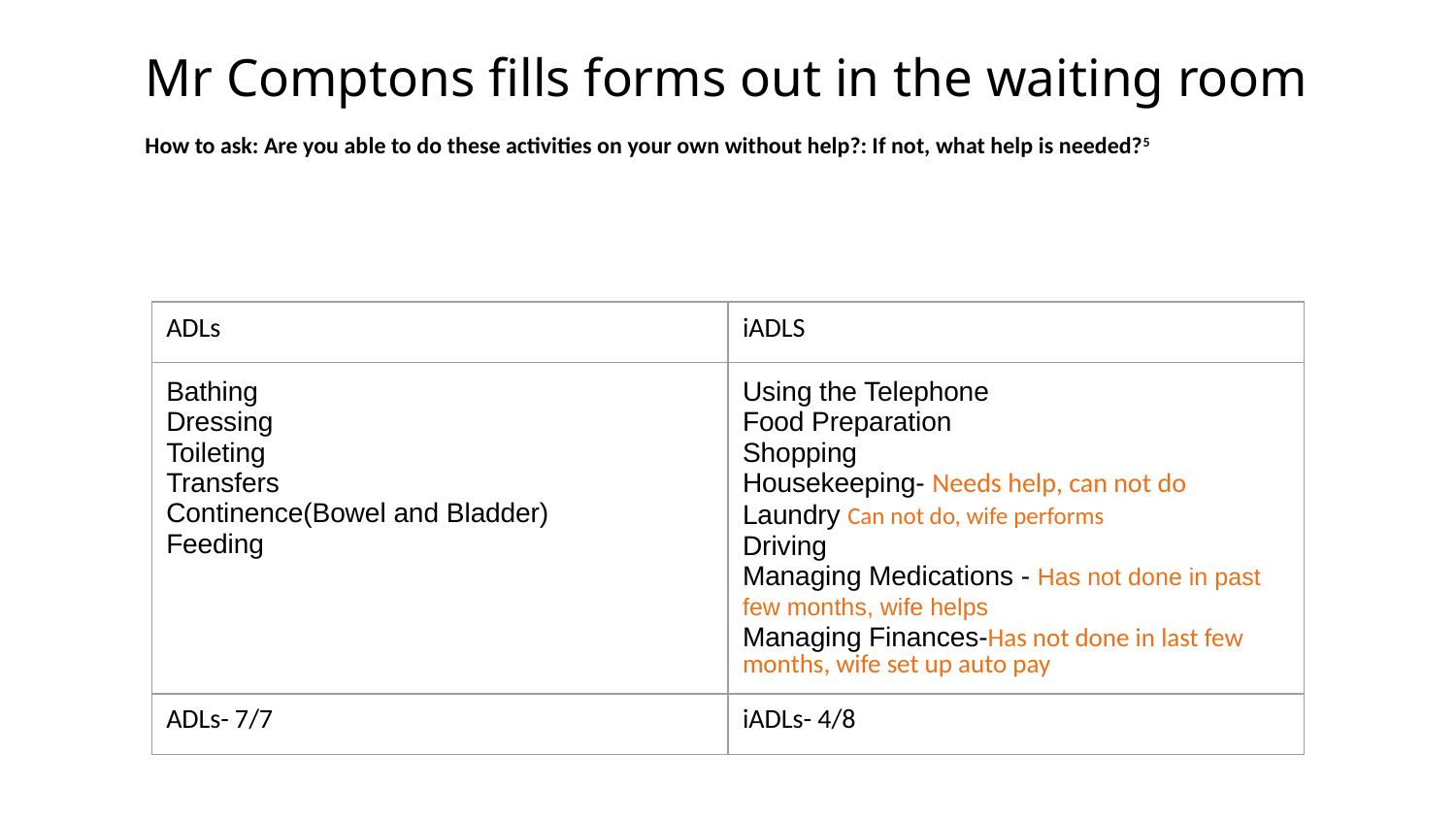

# Mr Comptons fills forms out in the waiting room
How to ask: Are you able to do these activities on your own without help?: If not, what help is needed?5
| ADLs | iADLS |
| --- | --- |
| Bathing Dressing Toileting Transfers Continence(Bowel and Bladder) Feeding | Using the Telephone Food Preparation Shopping Housekeeping- Needs help, can not do Laundry Can not do, wife performs Driving Managing Medications - Has not done in past few months, wife helps Managing Finances-Has not done in last few months, wife set up auto pay |
| ADLs- 7/7 | iADLs- 4/8 |

## Slide 14
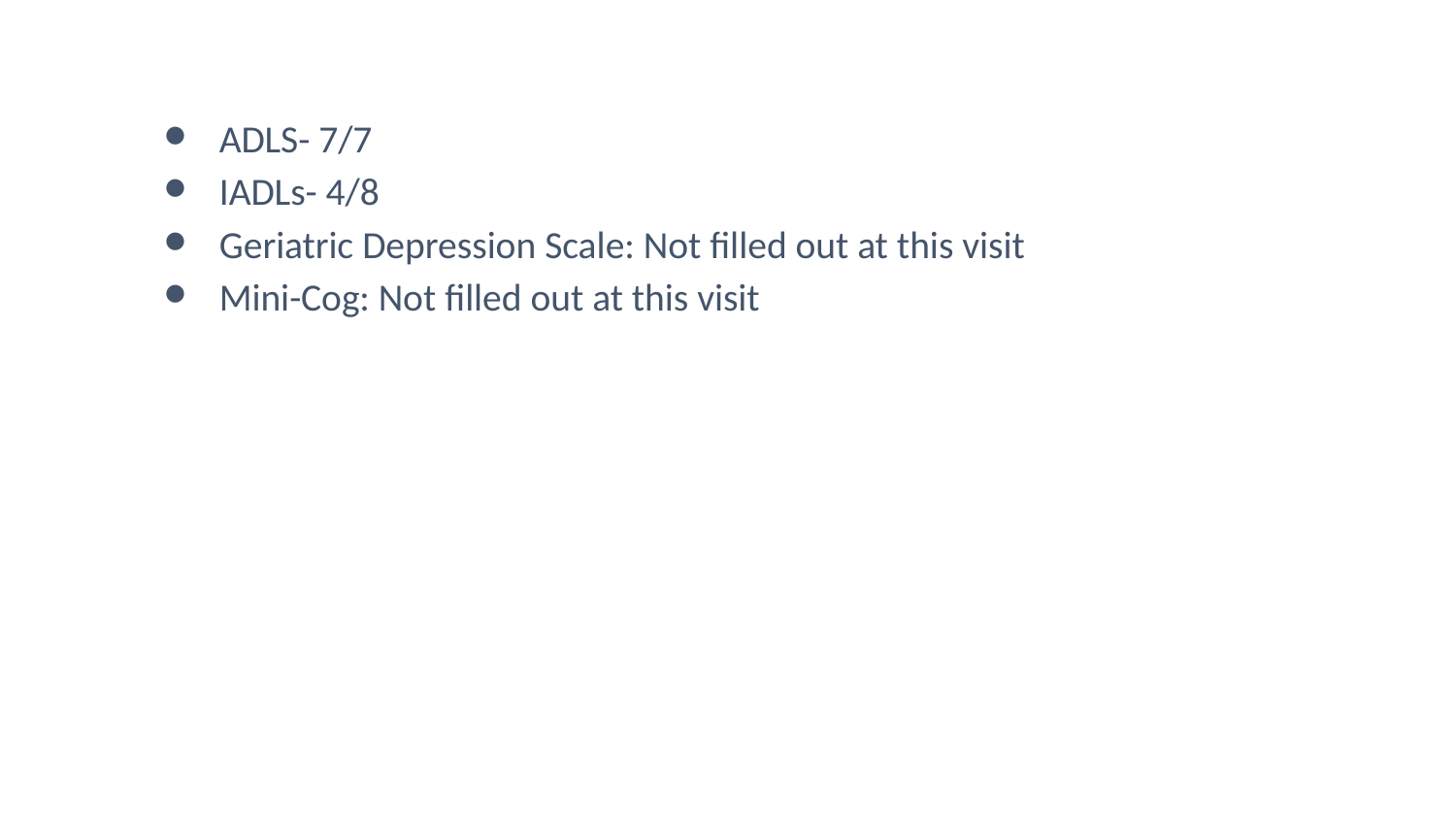

ADLS- 7/7
IADLs- 4/8
Geriatric Depression Scale: Not filled out at this visit
Mini-Cog: Not filled out at this visit

## Slide 15
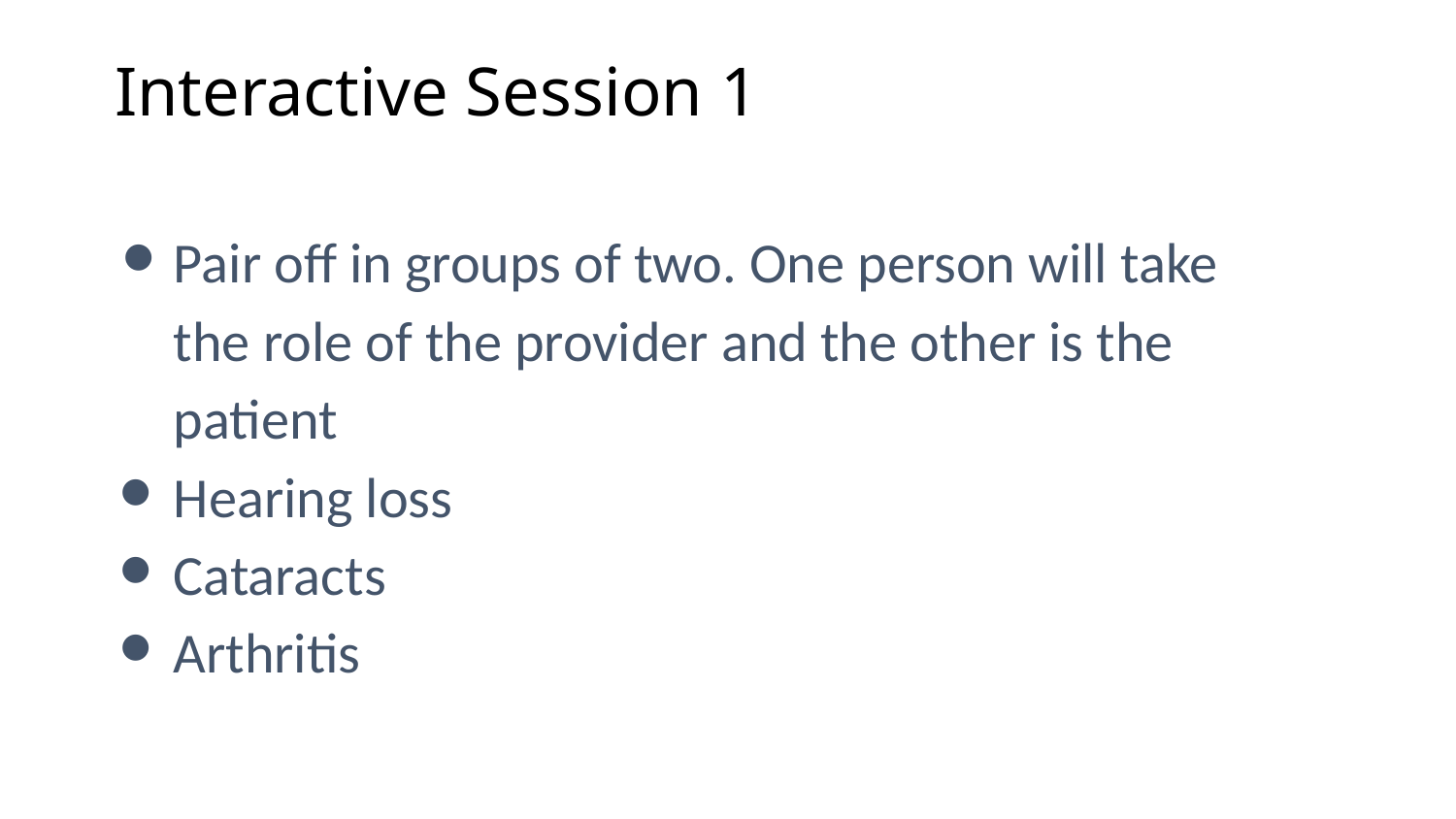

# Interactive Session 1
Pair off in groups of two. One person will take the role of the provider and the other is the patient
Hearing loss
Cataracts
Arthritis

## Slide 16
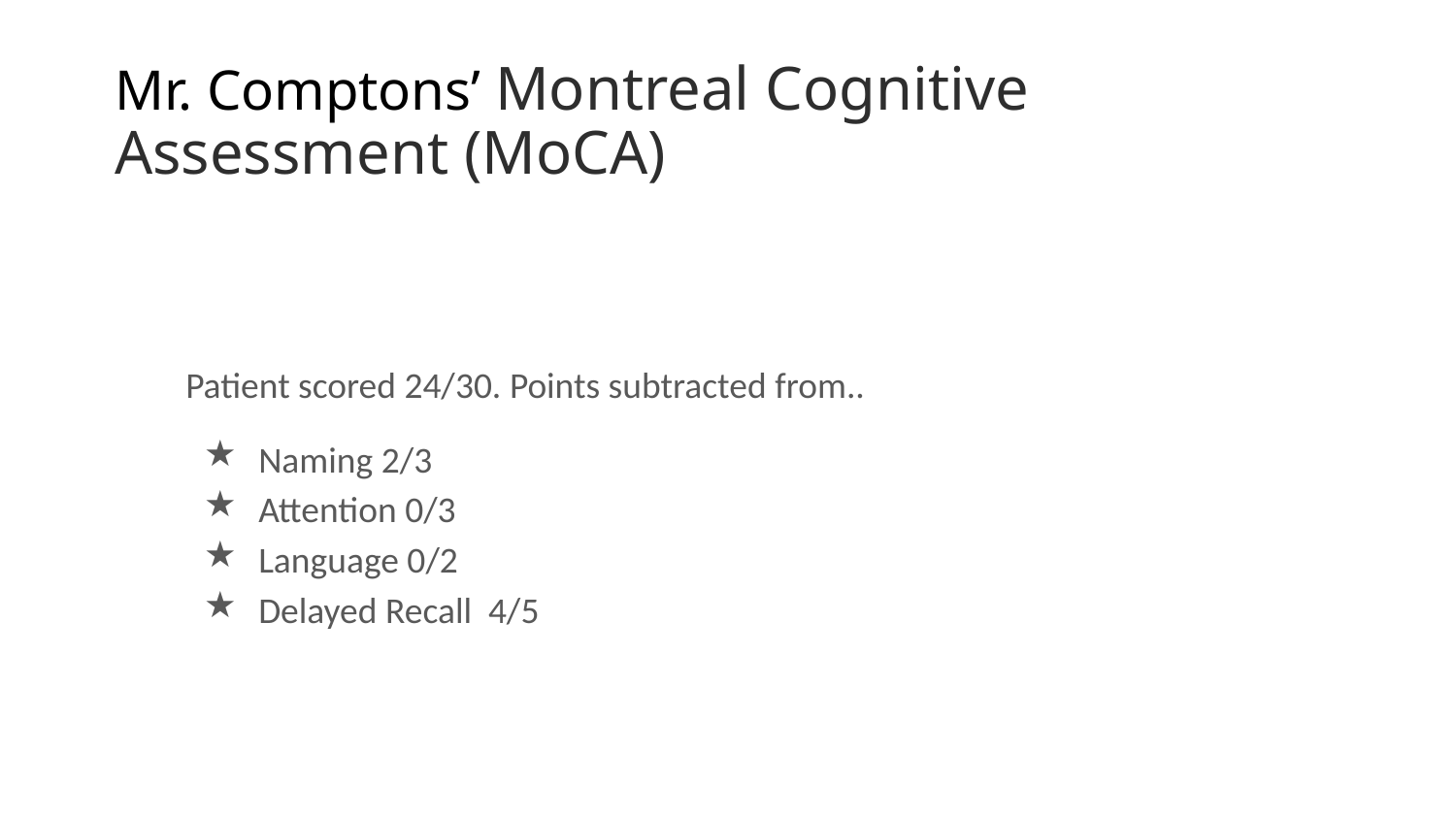

# Mr. Comptons’ Montreal Cognitive Assessment (MoCA)
Patient scored 24/30. Points subtracted from..
Naming 2/3
Attention 0/3
Language 0/2
Delayed Recall 4/5

## Slide 17
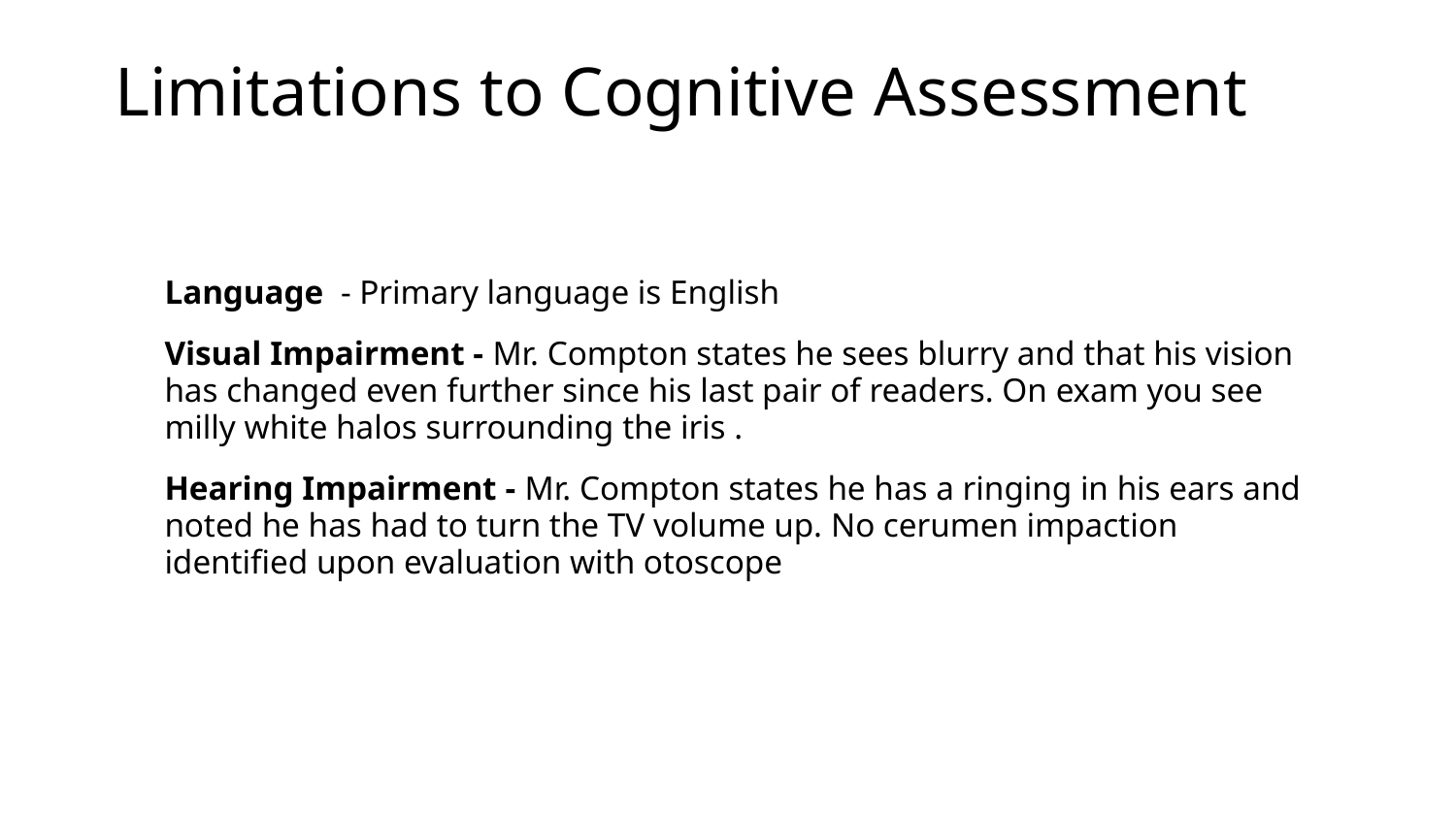

# Limitations to Cognitive Assessment
Language - Primary language is English
Visual Impairment - Mr. Compton states he sees blurry and that his vision has changed even further since his last pair of readers. On exam you see milly white halos surrounding the iris .
Hearing Impairment - Mr. Compton states he has a ringing in his ears and noted he has had to turn the TV volume up. No cerumen impaction identified upon evaluation with otoscope

## Slide 18
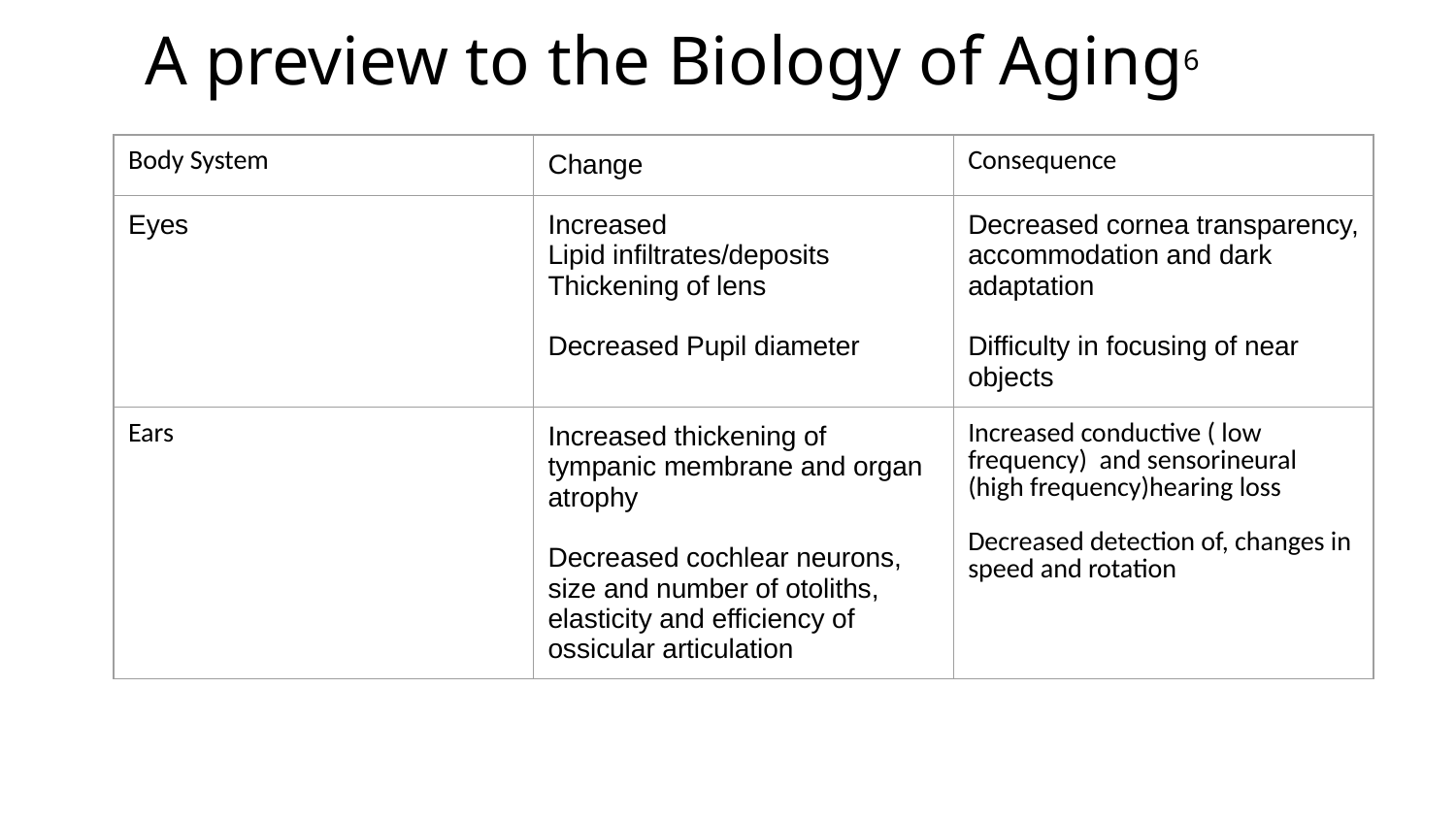

# A preview to the Biology of Aging6
| Body System | Change | Consequence |
| --- | --- | --- |
| Eyes | Increased Lipid infiltrates/deposits Thickening of lens Decreased Pupil diameter | Decreased cornea transparency, accommodation and dark adaptation Difficulty in focusing of near objects |
| Ears | Increased thickening of tympanic membrane and organ atrophy Decreased cochlear neurons, size and number of otoliths, elasticity and efficiency of ossicular articulation | Increased conductive ( low frequency) and sensorineural (high frequency)hearing loss Decreased detection of, changes in speed and rotation |

## Slide 19
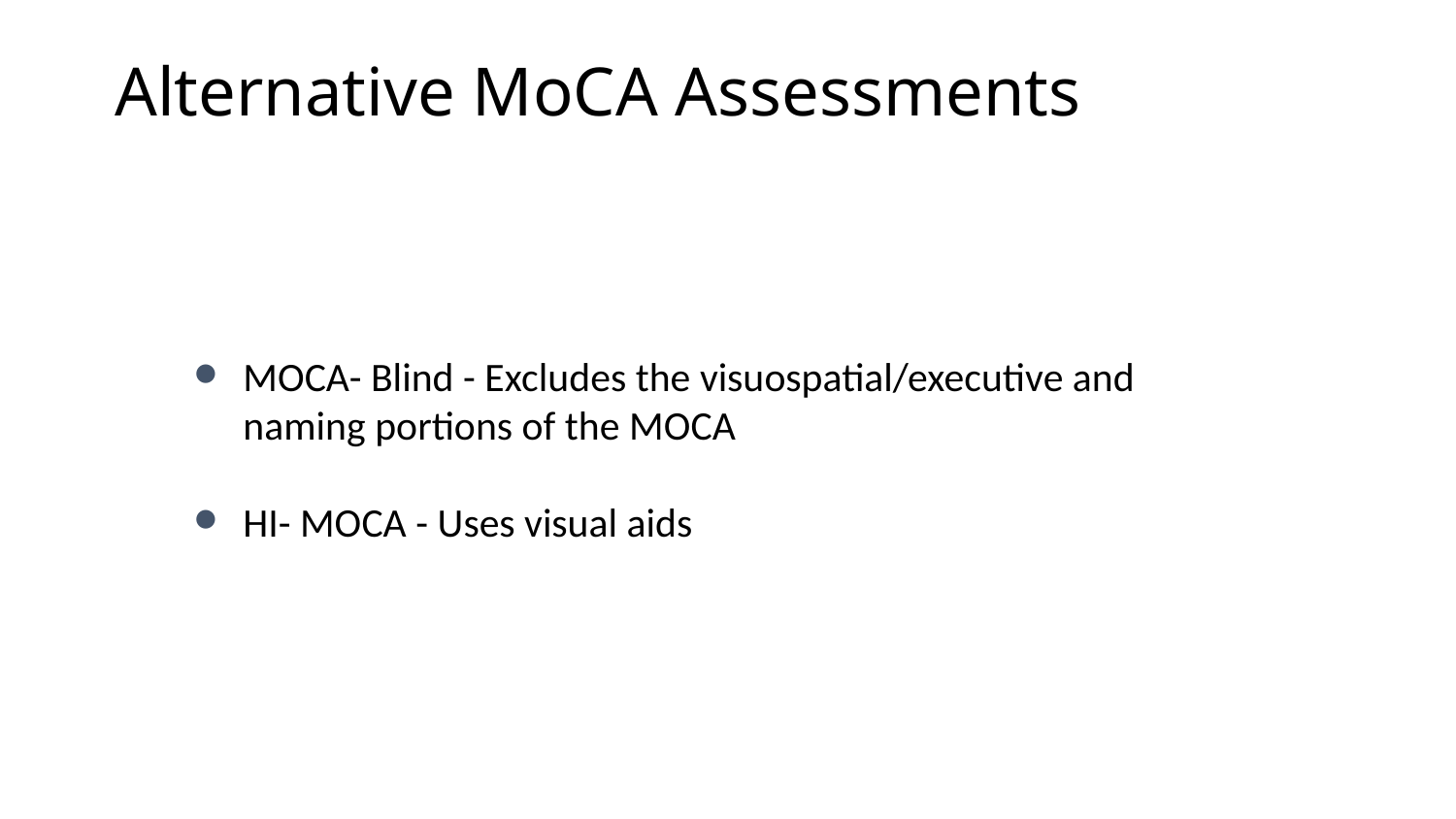

# Alternative MoCA Assessments
MOCA- Blind - Excludes the visuospatial/executive and naming portions of the MOCA
HI- MOCA - Uses visual aids

## Slide 20
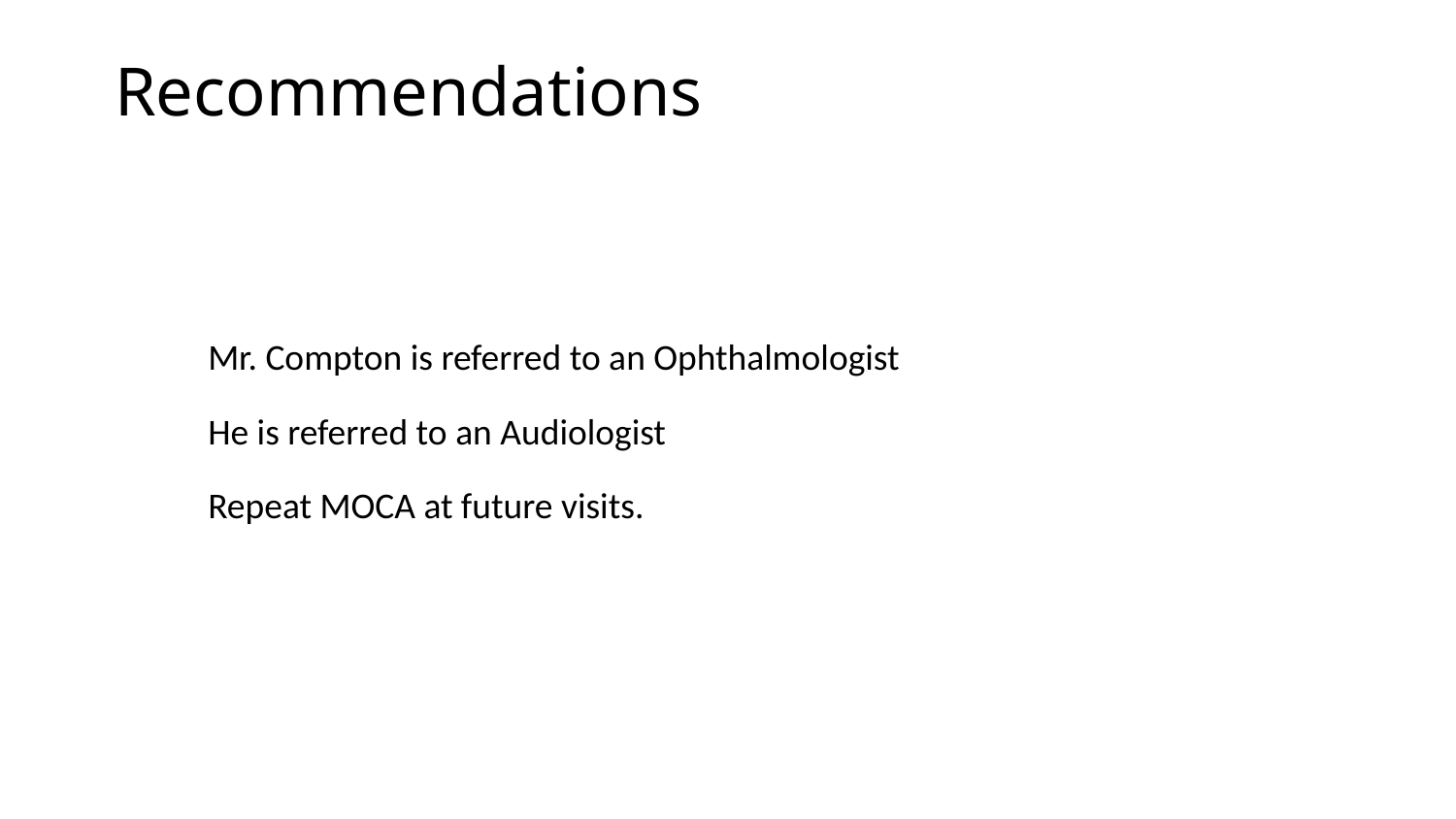

# Recommendations
Mr. Compton is referred to an Ophthalmologist
He is referred to an Audiologist
Repeat MOCA at future visits.

## Slide 21
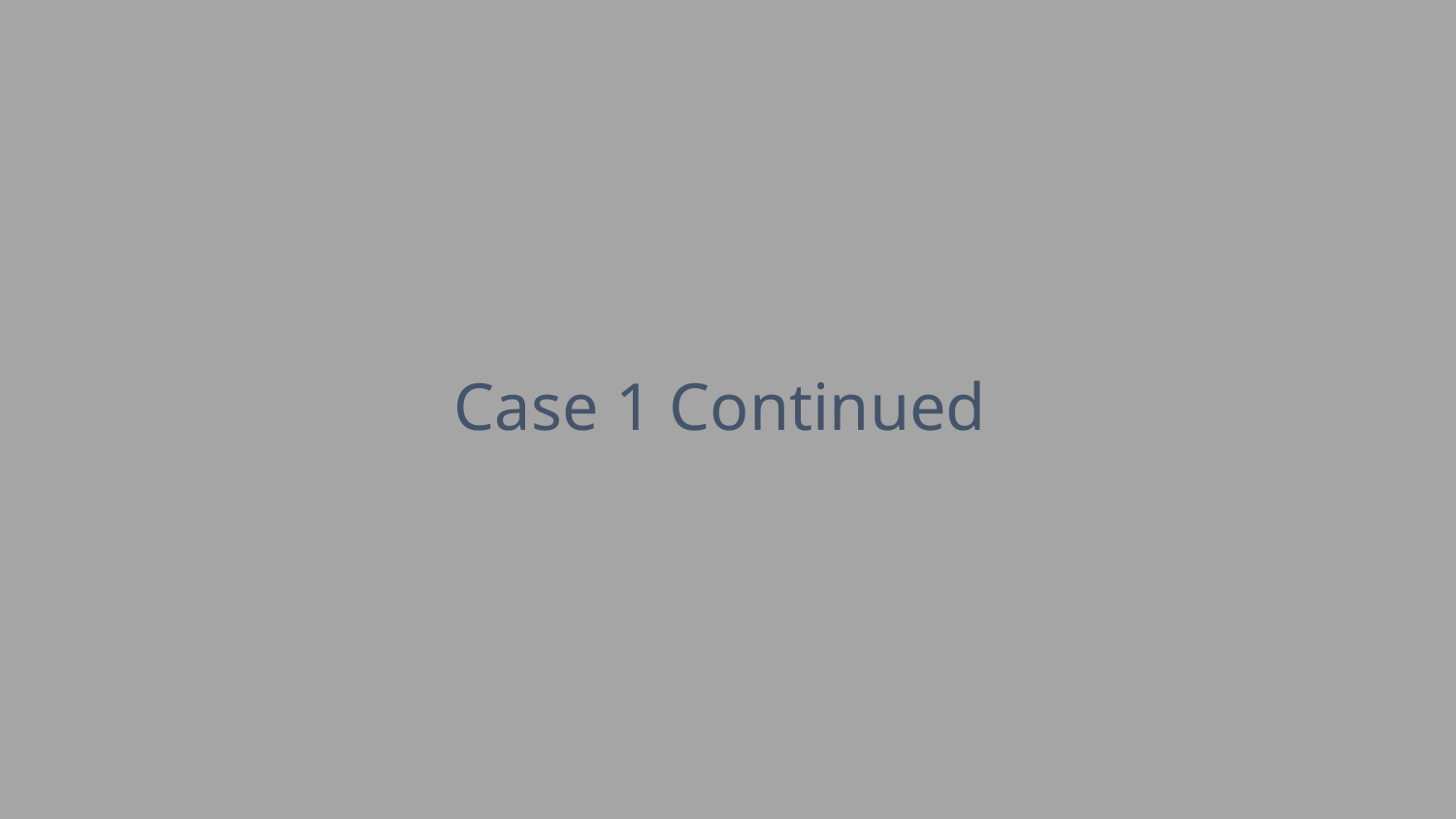

# Case 1 Continued

## Slide 22
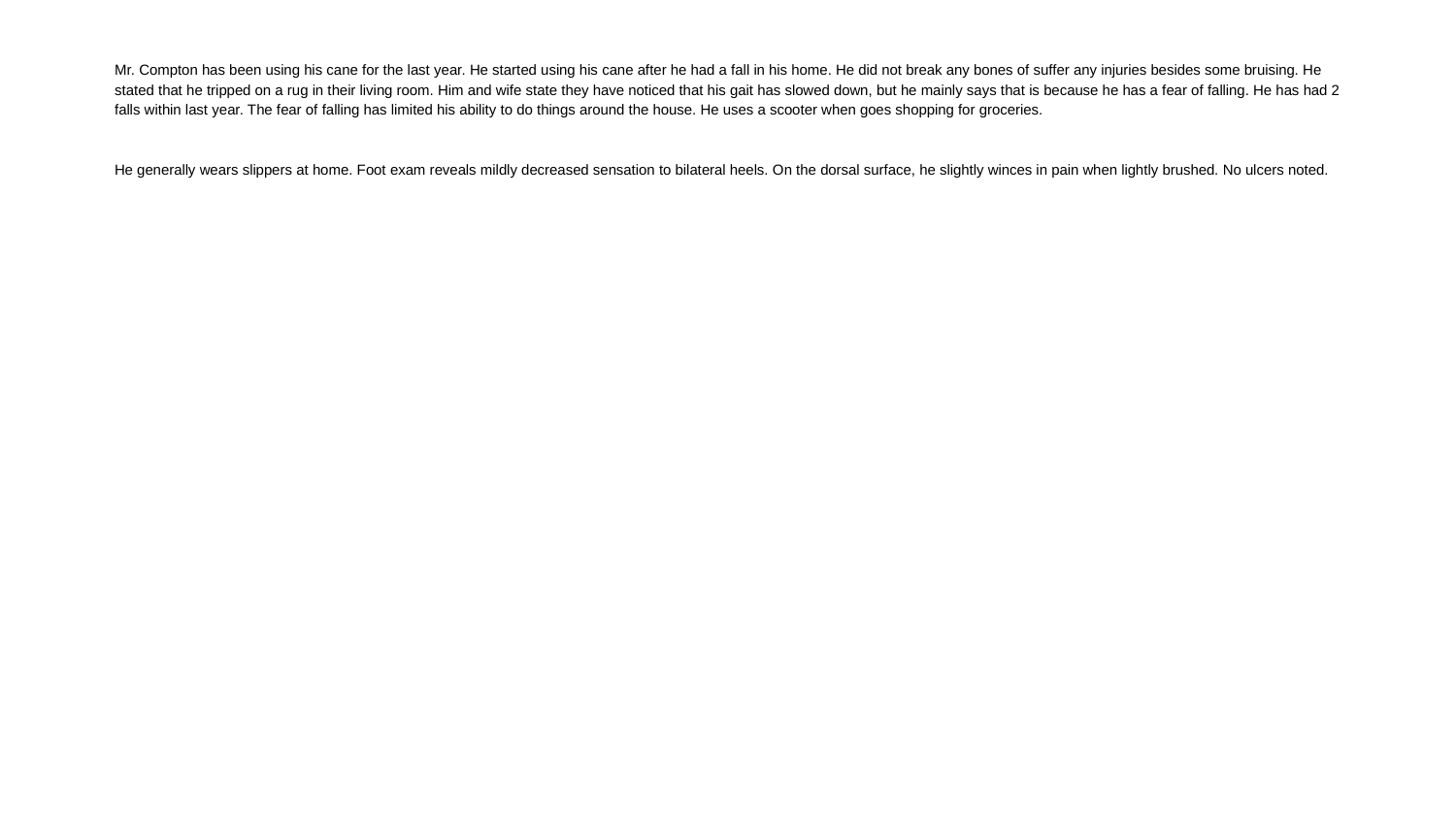

# Mr. Compton has been using his cane for the last year. He started using his cane after he had a fall in his home. He did not break any bones of suffer any injuries besides some bruising. He stated that he tripped on a rug in their living room. Him and wife state they have noticed that his gait has slowed down, but he mainly says that is because he has a fear of falling. He has had 2 falls within last year. The fear of falling has limited his ability to do things around the house. He uses a scooter when goes shopping for groceries.
He generally wears slippers at home. Foot exam reveals mildly decreased sensation to bilateral heels. On the dorsal surface, he slightly winces in pain when lightly brushed. No ulcers noted.

## Slide 23
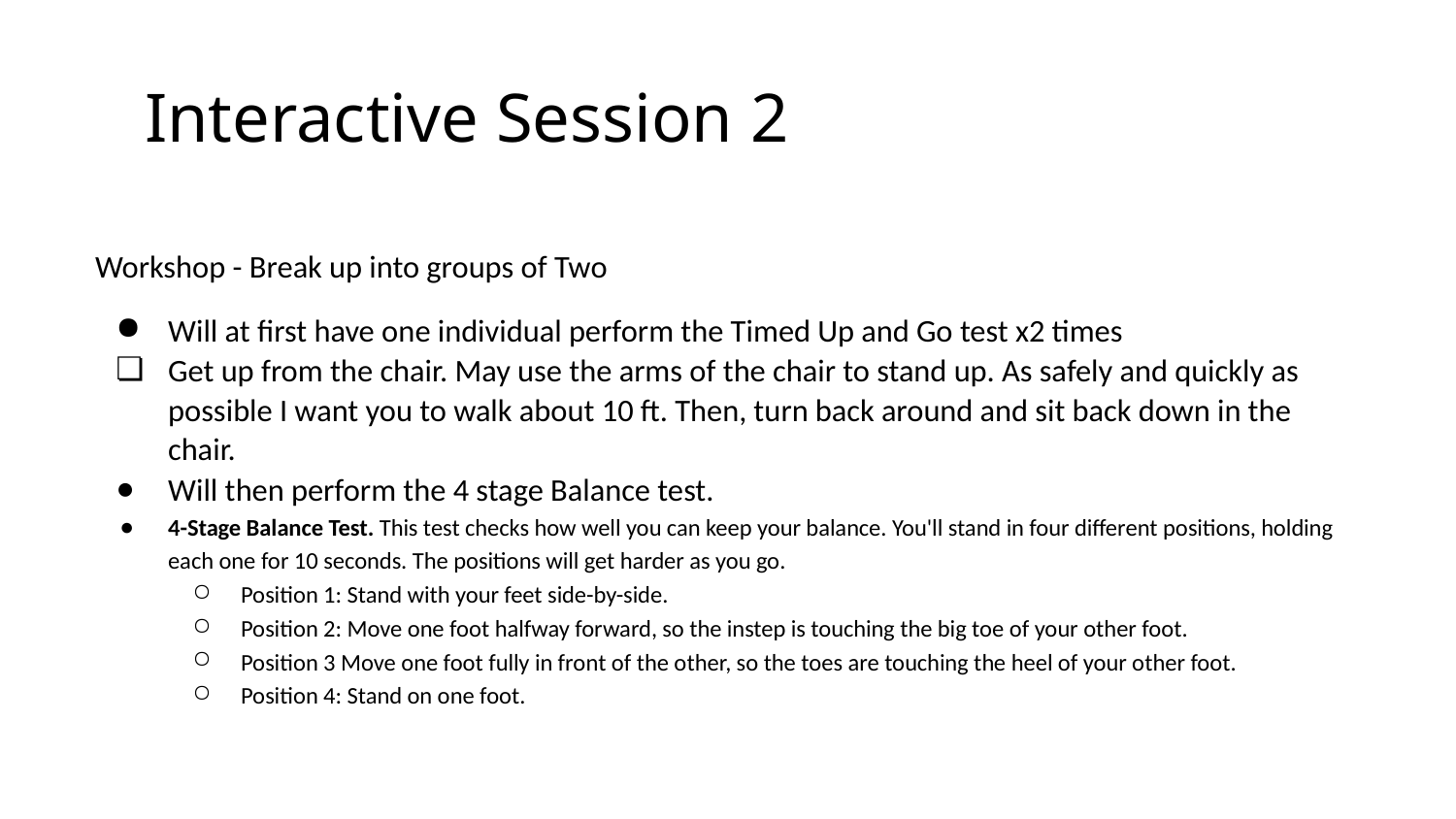

# Interactive Session 2
Workshop - Break up into groups of Two
Will at first have one individual perform the Timed Up and Go test x2 times
Get up from the chair. May use the arms of the chair to stand up. As safely and quickly as possible I want you to walk about 10 ft. Then, turn back around and sit back down in the chair.
Will then perform the 4 stage Balance test.
4-Stage Balance Test. This test checks how well you can keep your balance. You'll stand in four different positions, holding each one for 10 seconds. The positions will get harder as you go.
Position 1: Stand with your feet side-by-side.
Position 2: Move one foot halfway forward, so the instep is touching the big toe of your other foot.
Position 3 Move one foot fully in front of the other, so the toes are touching the heel of your other foot.
Position 4: Stand on one foot.

## Slide 24
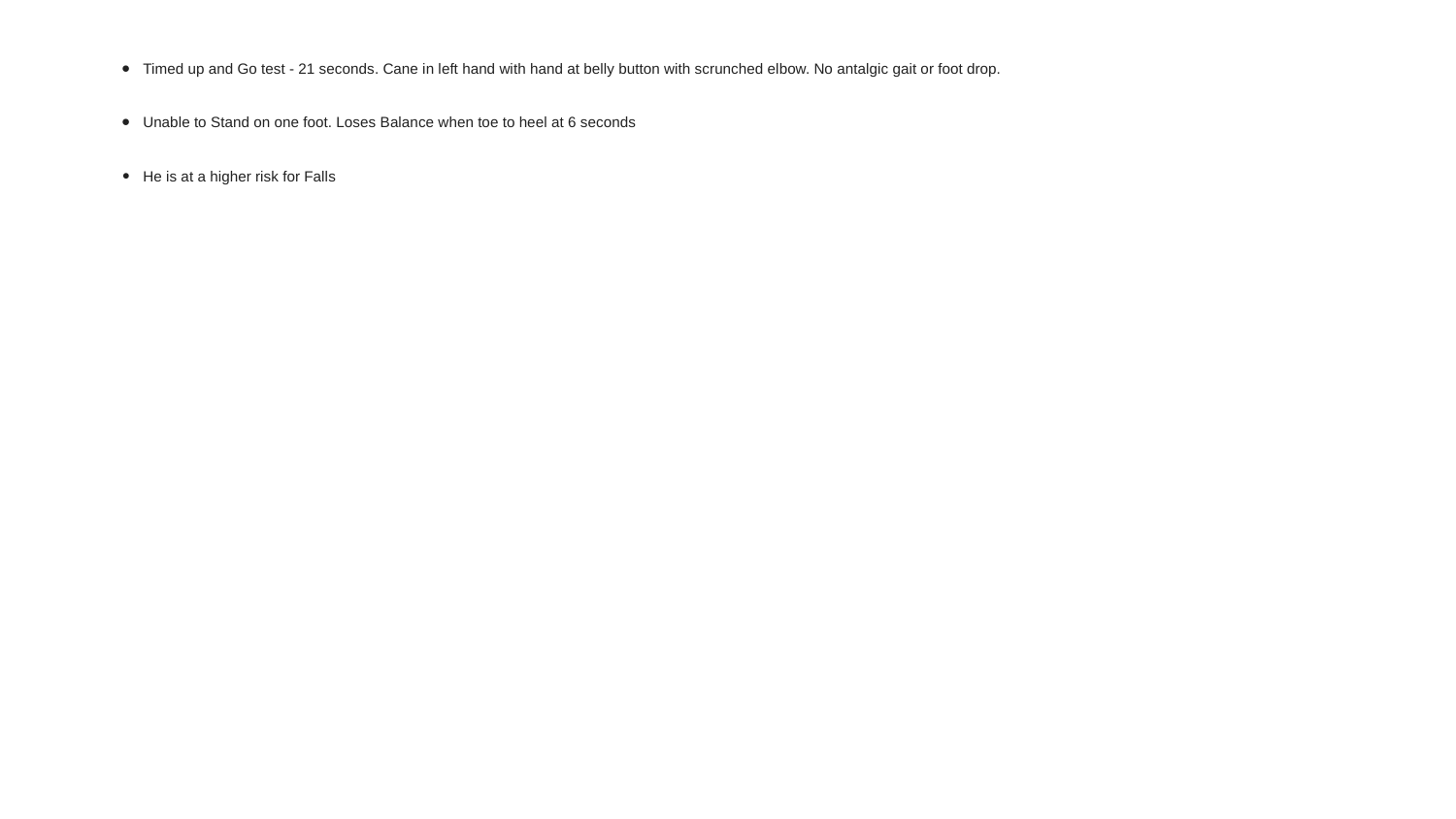

# Timed up and Go test - 21 seconds. Cane in left hand with hand at belly button with scrunched elbow. No antalgic gait or foot drop.
Unable to Stand on one foot. Loses Balance when toe to heel at 6 seconds
He is at a higher risk for Falls

## Slide 25
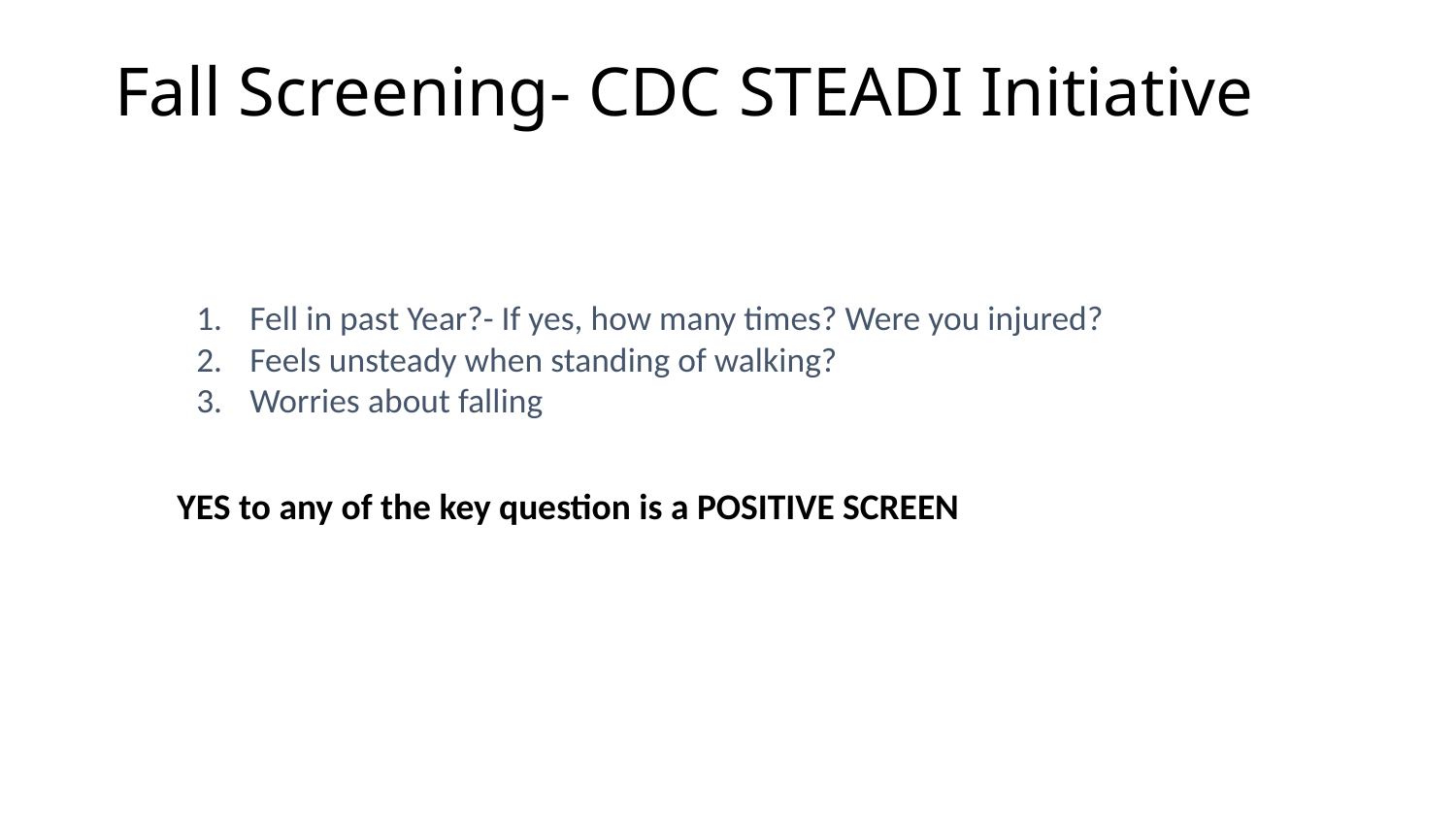

# Fall Screening- CDC STEADI Initiative
Fell in past Year?- If yes, how many times? Were you injured?
Feels unsteady when standing of walking?
Worries about falling
YES to any of the key question is a POSITIVE SCREEN

## Slide 26
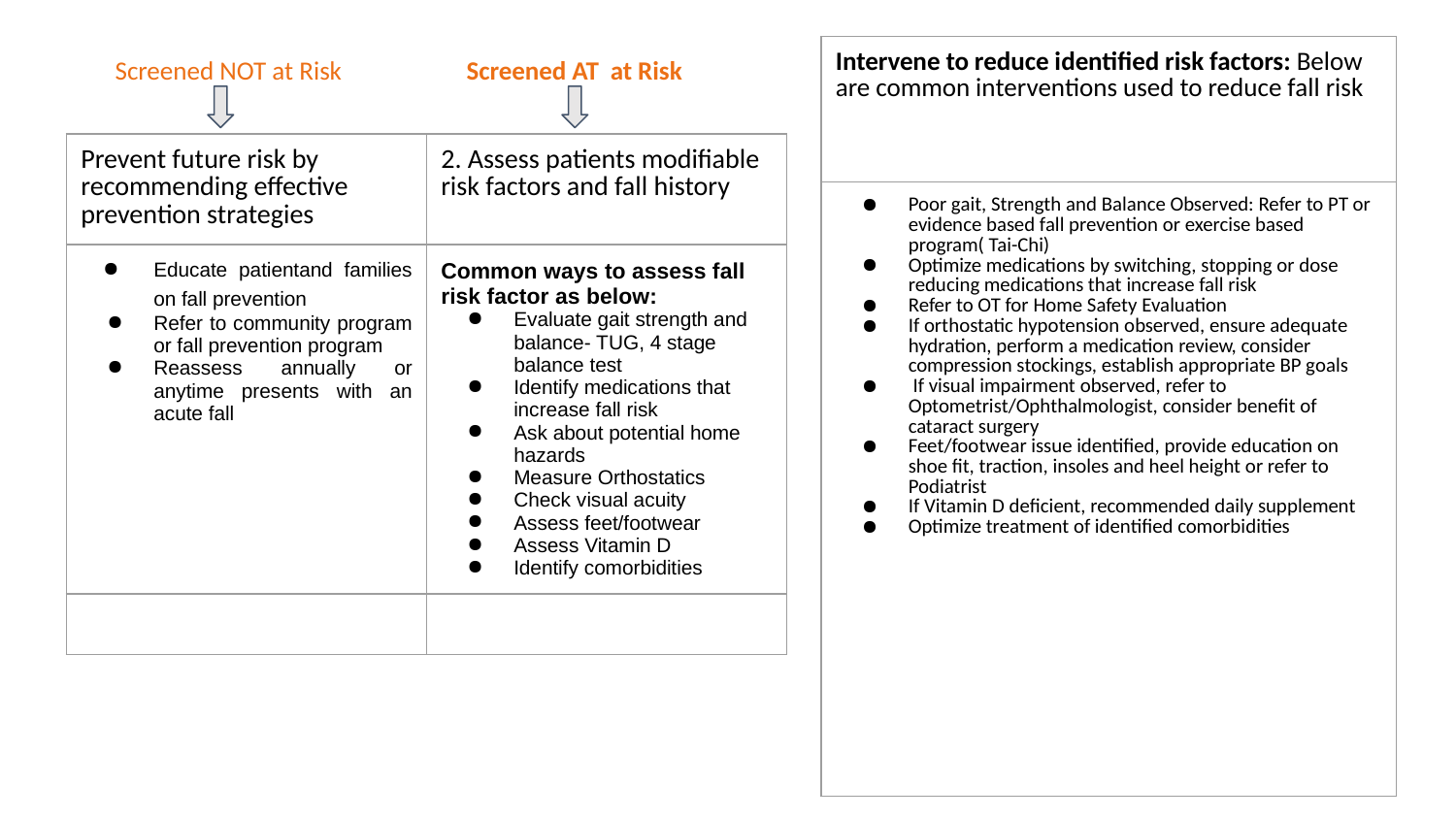

| Intervene to reduce identified risk factors: Below are common interventions used to reduce fall risk |
| --- |
| Poor gait, Strength and Balance Observed: Refer to PT or evidence based fall prevention or exercise based program( Tai-Chi) Optimize medications by switching, stopping or dose reducing medications that increase fall risk Refer to OT for Home Safety Evaluation If orthostatic hypotension observed, ensure adequate hydration, perform a medication review, consider compression stockings, establish appropriate BP goals If visual impairment observed, refer to Optometrist/Ophthalmologist, consider benefit of cataract surgery Feet/footwear issue identified, provide education on shoe fit, traction, insoles and heel height or refer to Podiatrist If Vitamin D deficient, recommended daily supplement Optimize treatment of identified comorbidities |
Screened NOT at Risk
Screened AT at Risk
| Prevent future risk by recommending effective prevention strategies | 2. Assess patients modifiable risk factors and fall history |
| --- | --- |
| Educate patientand families on fall prevention Refer to community program or fall prevention program Reassess annually or anytime presents with an acute fall | Common ways to assess fall risk factor as below: Evaluate gait strength and balance- TUG, 4 stage balance test Identify medications that increase fall risk Ask about potential home hazards Measure Orthostatics Check visual acuity Assess feet/footwear Assess Vitamin D Identify comorbidities |
| | |

## Slide 27
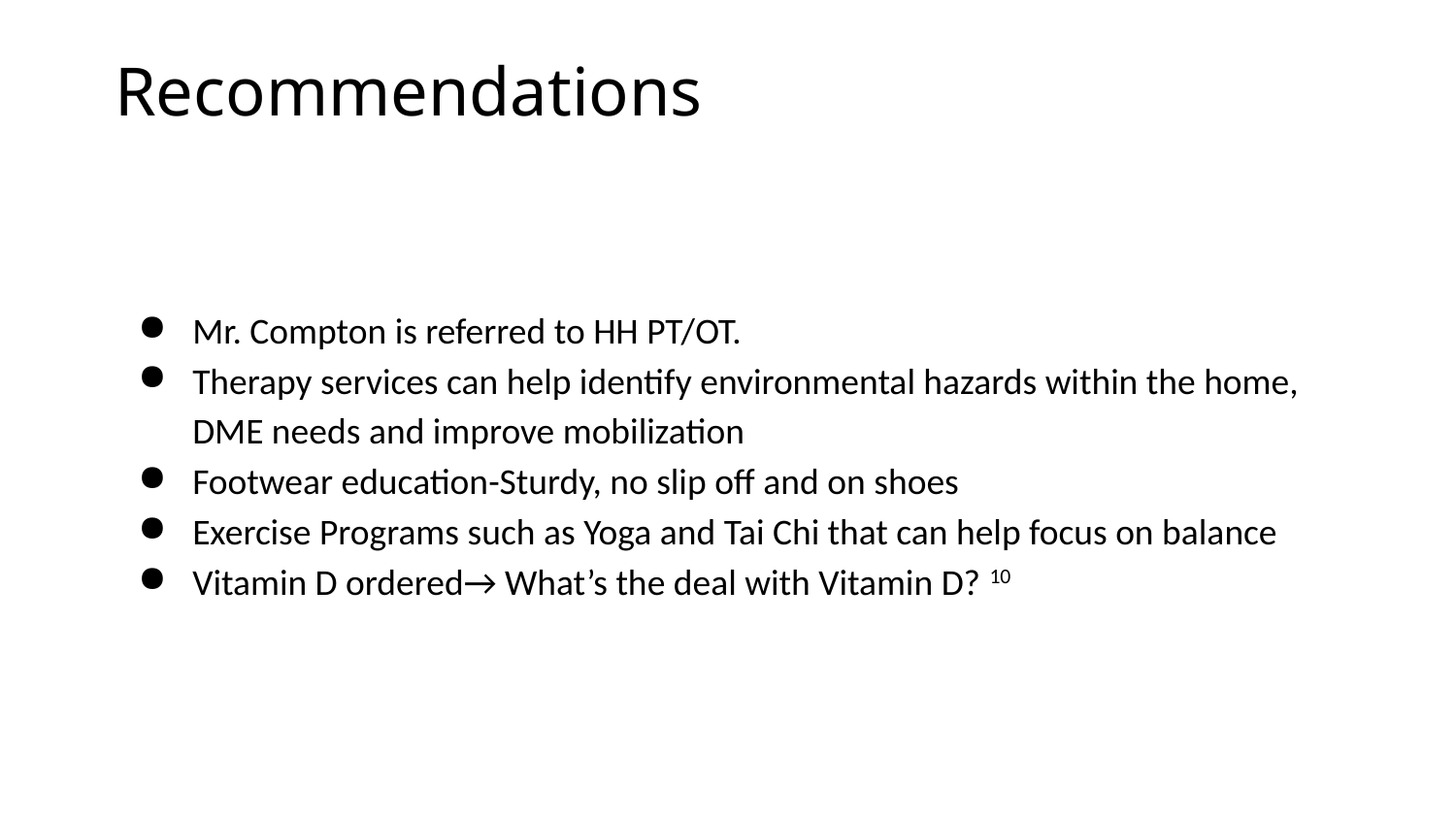

# Recommendations
Mr. Compton is referred to HH PT/OT.
Therapy services can help identify environmental hazards within the home, DME needs and improve mobilization
Footwear education-Sturdy, no slip off and on shoes
Exercise Programs such as Yoga and Tai Chi that can help focus on balance
Vitamin D ordered→ What’s the deal with Vitamin D? 10

## Slide 28
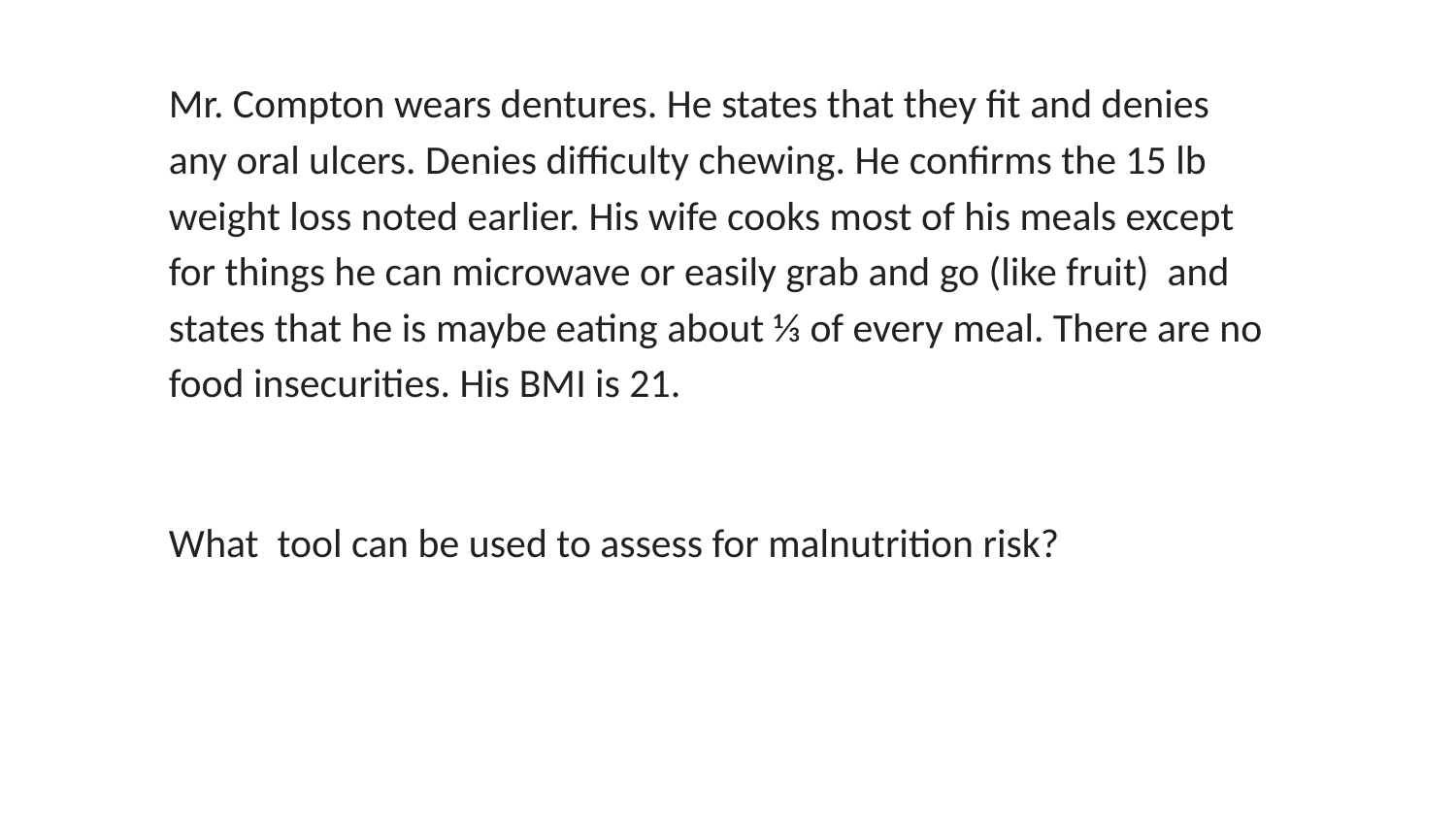

Mr. Compton wears dentures. He states that they fit and denies any oral ulcers. Denies difficulty chewing. He confirms the 15 lb weight loss noted earlier. His wife cooks most of his meals except for things he can microwave or easily grab and go (like fruit) and states that he is maybe eating about ⅓ of every meal. There are no food insecurities. His BMI is 21.
What tool can be used to assess for malnutrition risk?

## Slide 29
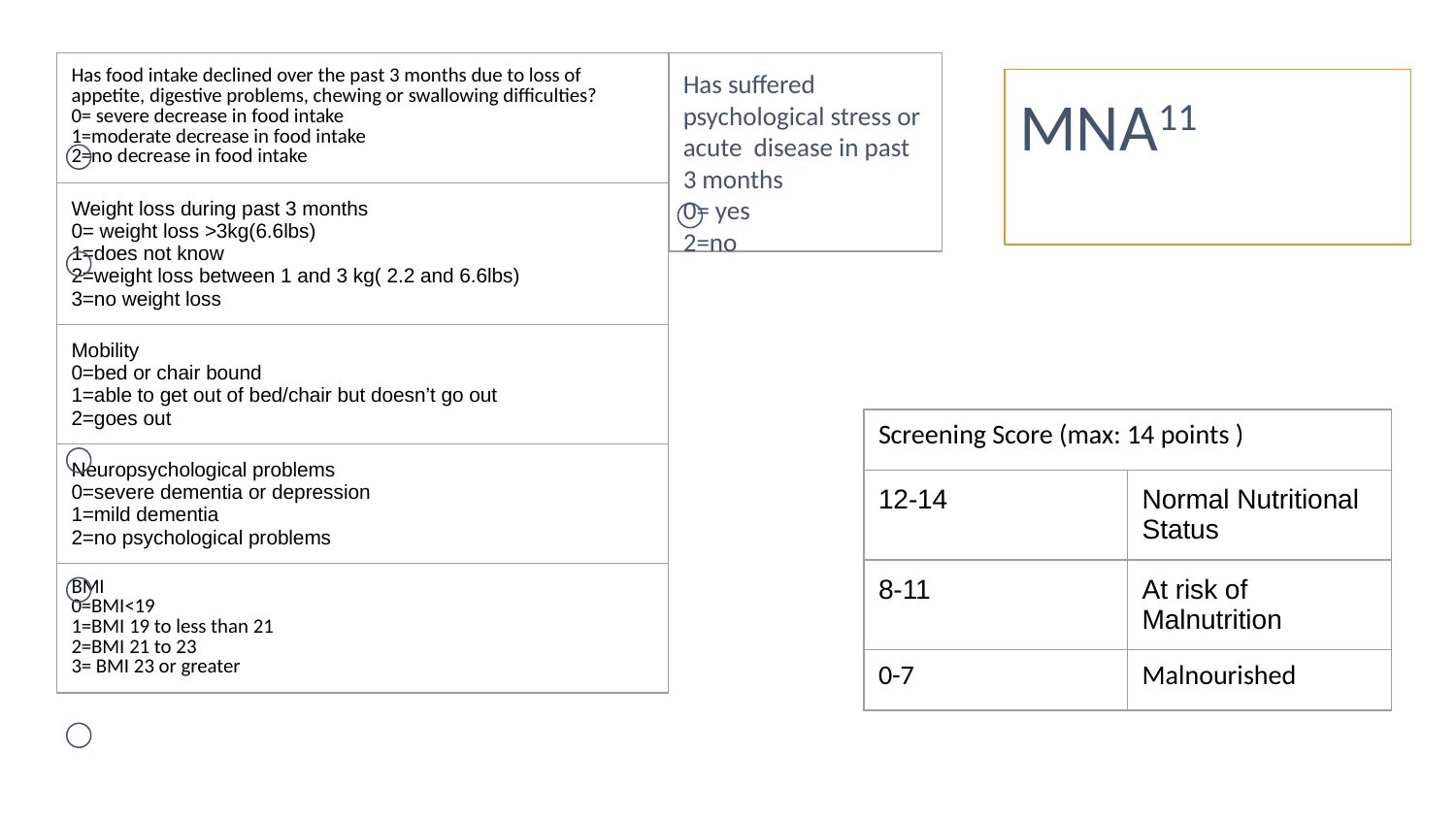

| Has food intake declined over the past 3 months due to loss of appetite, digestive problems, chewing or swallowing difficulties? 0= severe decrease in food intake 1=moderate decrease in food intake 2=no decrease in food intake |
| --- |
| Weight loss during past 3 months 0= weight loss >3kg(6.6lbs) 1=does not know 2=weight loss between 1 and 3 kg( 2.2 and 6.6lbs) 3=no weight loss |
| Mobility 0=bed or chair bound 1=able to get out of bed/chair but doesn’t go out 2=goes out |
| Neuropsychological problems 0=severe dementia or depression 1=mild dementia 2=no psychological problems |
| BMI 0=BMI<19 1=BMI 19 to less than 21 2=BMI 21 to 23 3= BMI 23 or greater |
Has suffered psychological stress or acute disease in past 3 months
0= yes
2=no
MNA11
| Screening Score (max: 14 points ) | |
| --- | --- |
| 12-14 | Normal Nutritional Status |
| 8-11 | At risk of Malnutrition |
| 0-7 | Malnourished |

## Slide 30
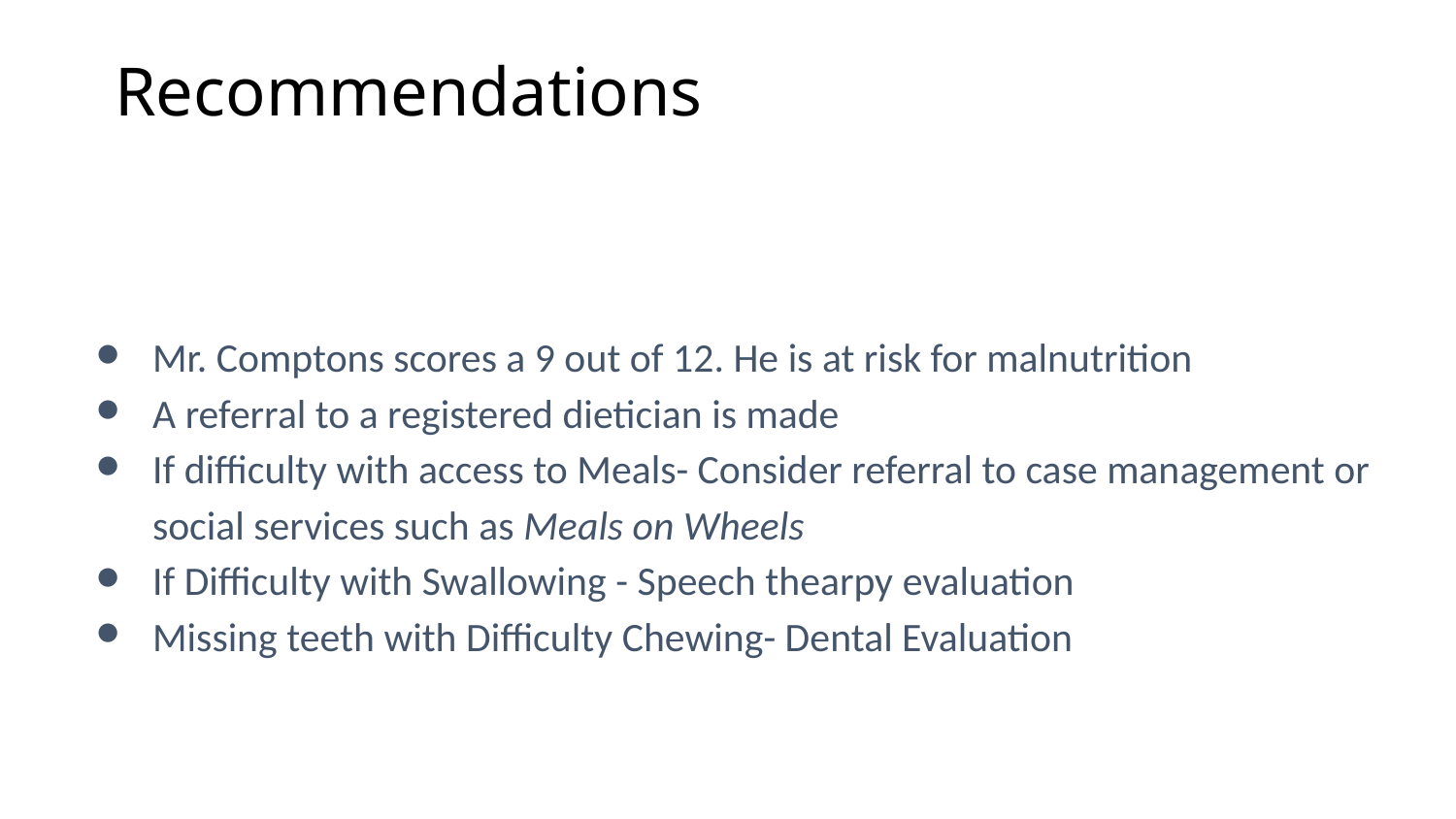

# Recommendations
Mr. Comptons scores a 9 out of 12. He is at risk for malnutrition
A referral to a registered dietician is made
If difficulty with access to Meals- Consider referral to case management or social services such as Meals on Wheels
If Difficulty with Swallowing - Speech thearpy evaluation
Missing teeth with Difficulty Chewing- Dental Evaluation

## Slide 31
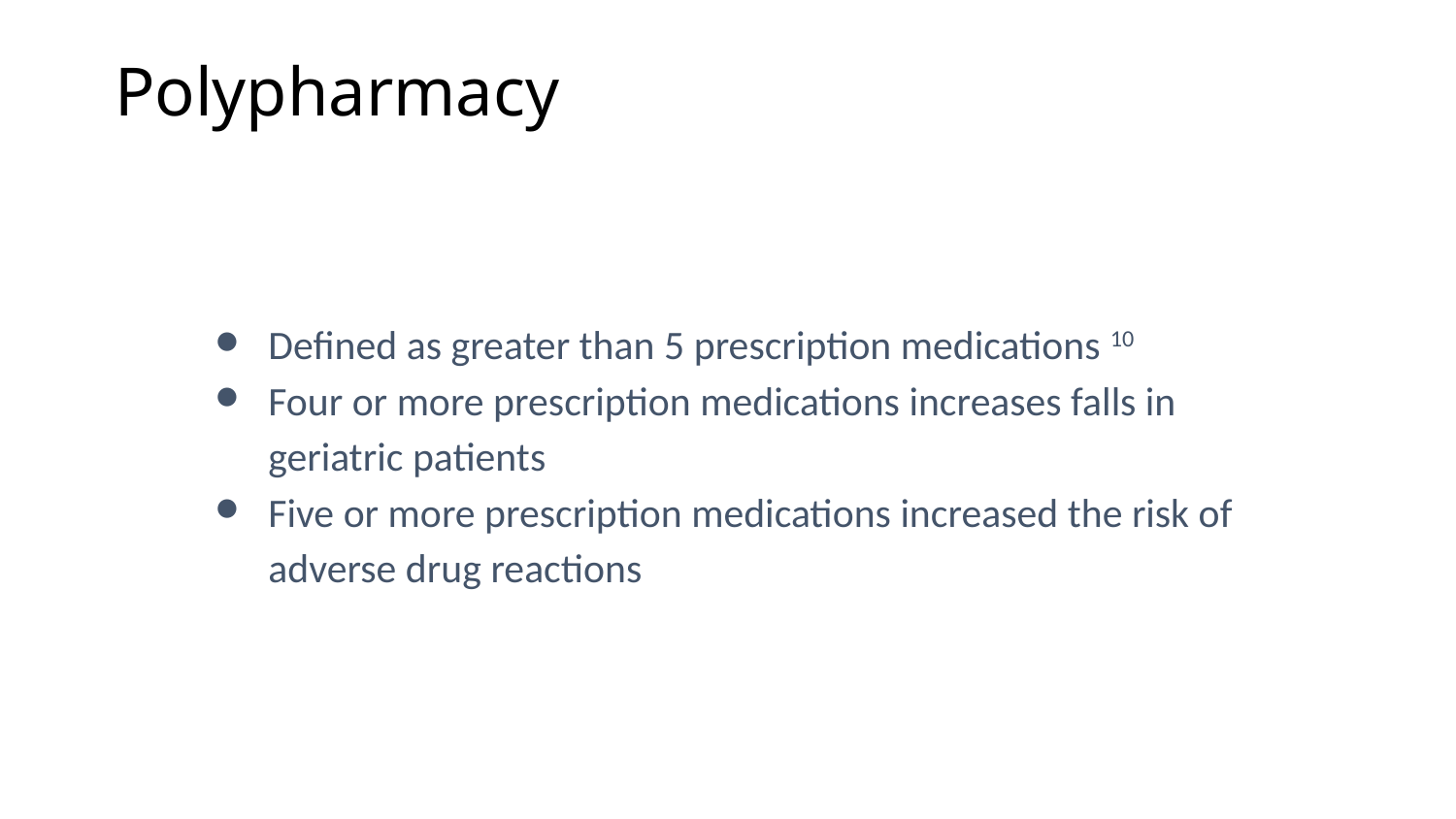

# Polypharmacy
Defined as greater than 5 prescription medications 10
Four or more prescription medications increases falls in geriatric patients
Five or more prescription medications increased the risk of adverse drug reactions

## Slide 32
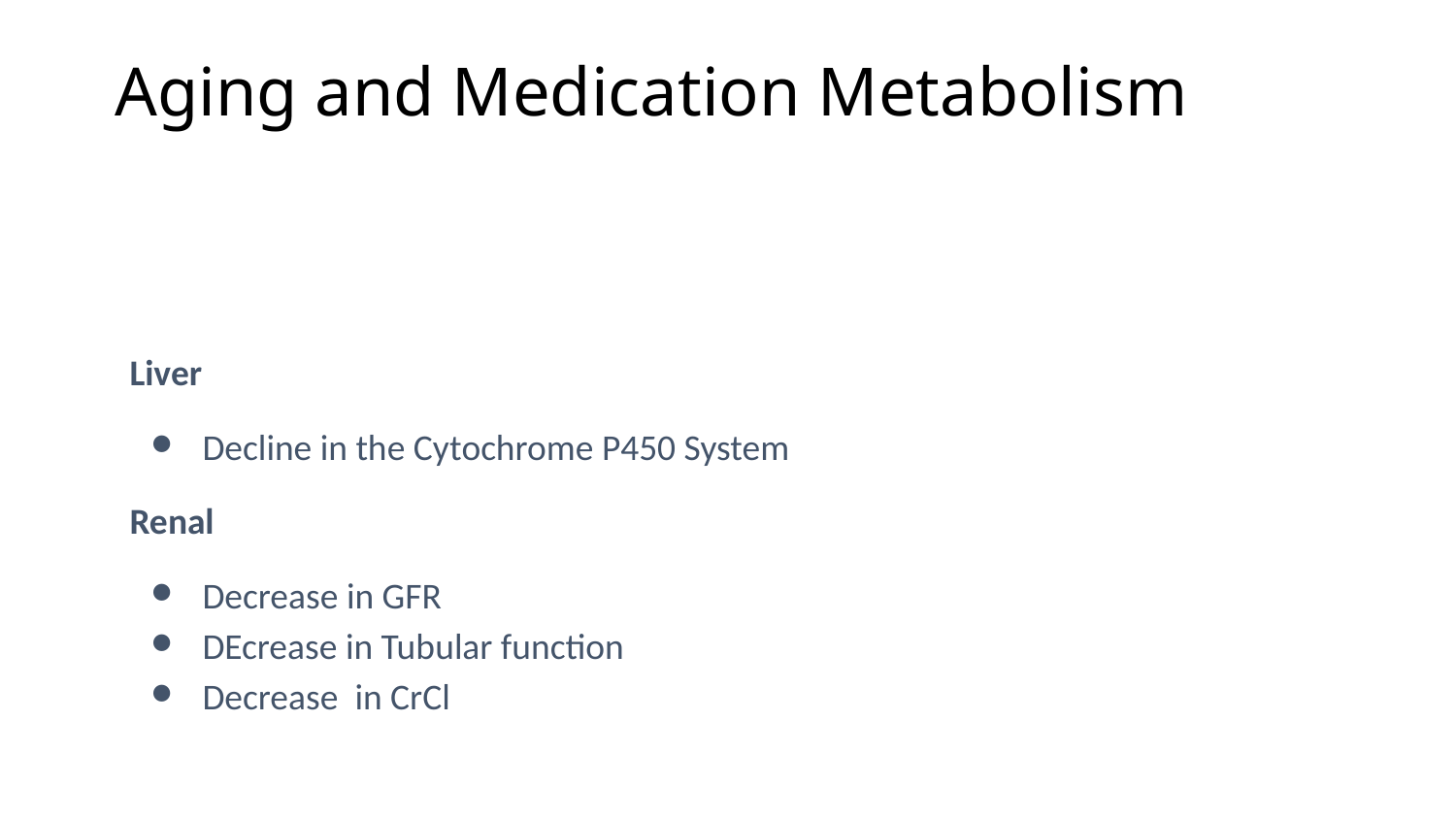

# Aging and Medication Metabolism
Liver
Decline in the Cytochrome P450 System
Renal
Decrease in GFR
DEcrease in Tubular function
Decrease in CrCl

## Slide 33
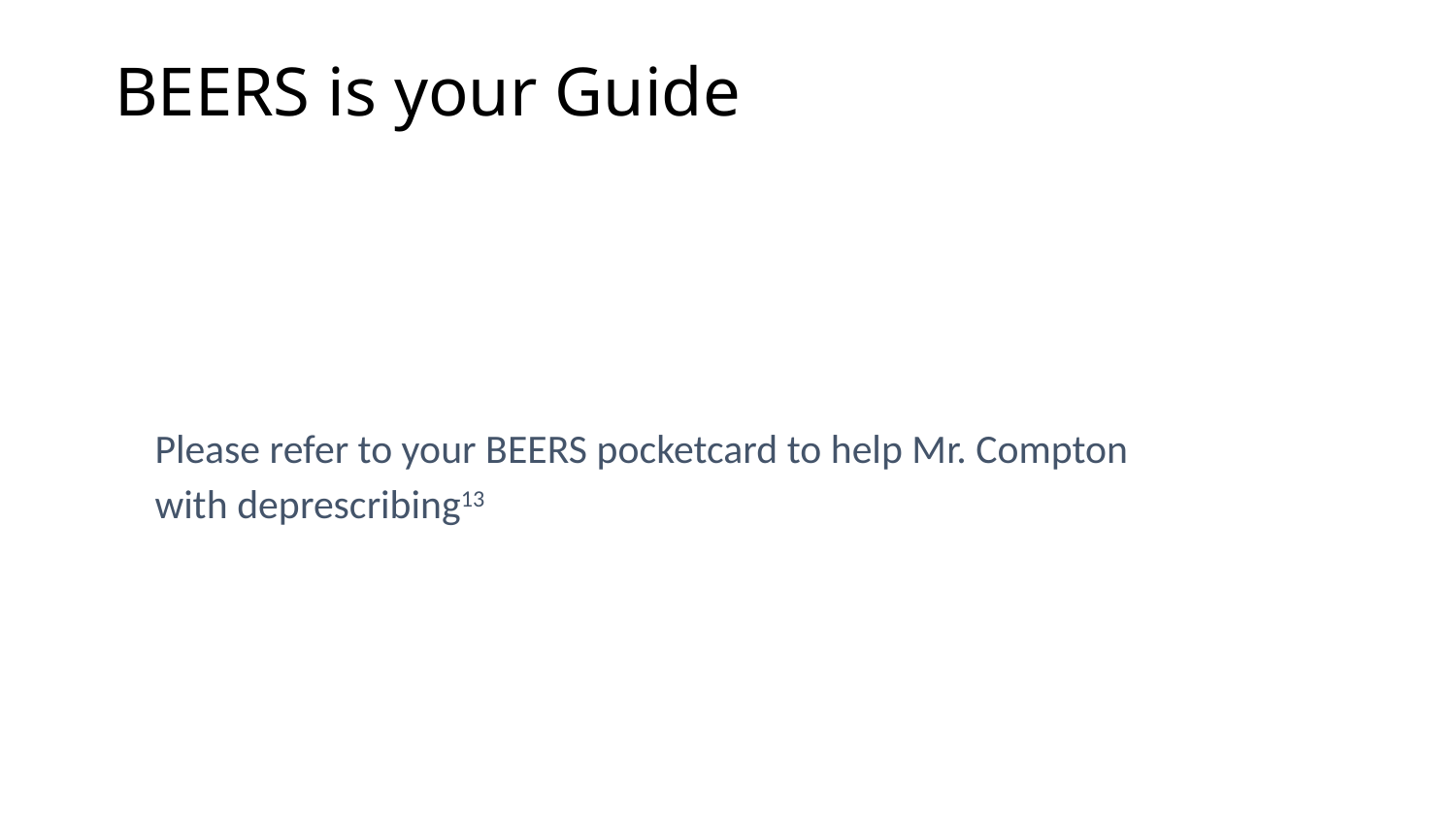

# BEERS is your Guide
Please refer to your BEERS pocketcard to help Mr. Compton with deprescribing13

## Slide 34
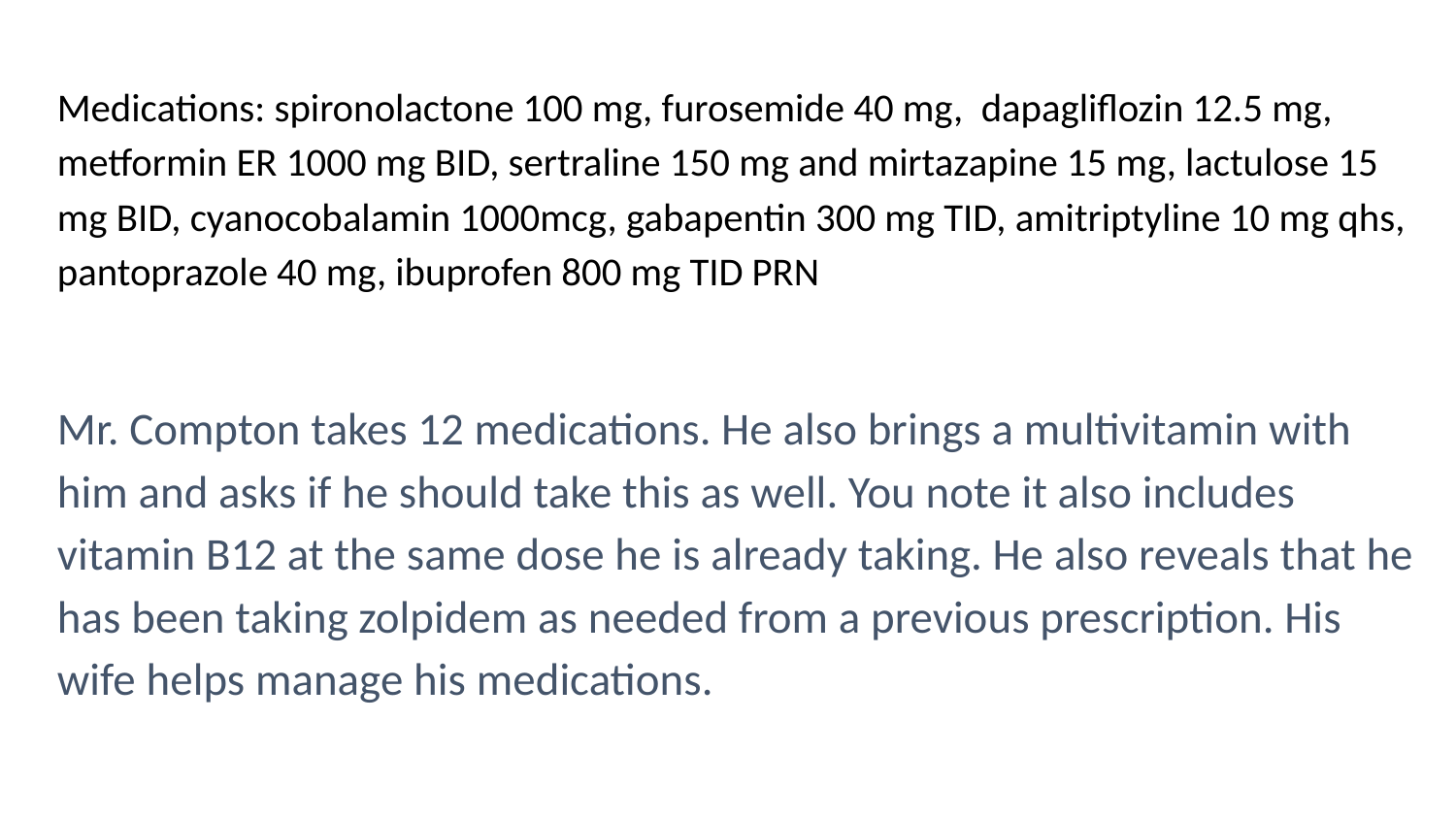

Medications: spironolactone 100 mg, furosemide 40 mg, dapagliflozin 12.5 mg, metformin ER 1000 mg BID, sertraline 150 mg and mirtazapine 15 mg, lactulose 15 mg BID, cyanocobalamin 1000mcg, gabapentin 300 mg TID, amitriptyline 10 mg qhs, pantoprazole 40 mg, ibuprofen 800 mg TID PRN
Mr. Compton takes 12 medications. He also brings a multivitamin with him and asks if he should take this as well. You note it also includes vitamin B12 at the same dose he is already taking. He also reveals that he has been taking zolpidem as needed from a previous prescription. His wife helps manage his medications.

## Slide 35
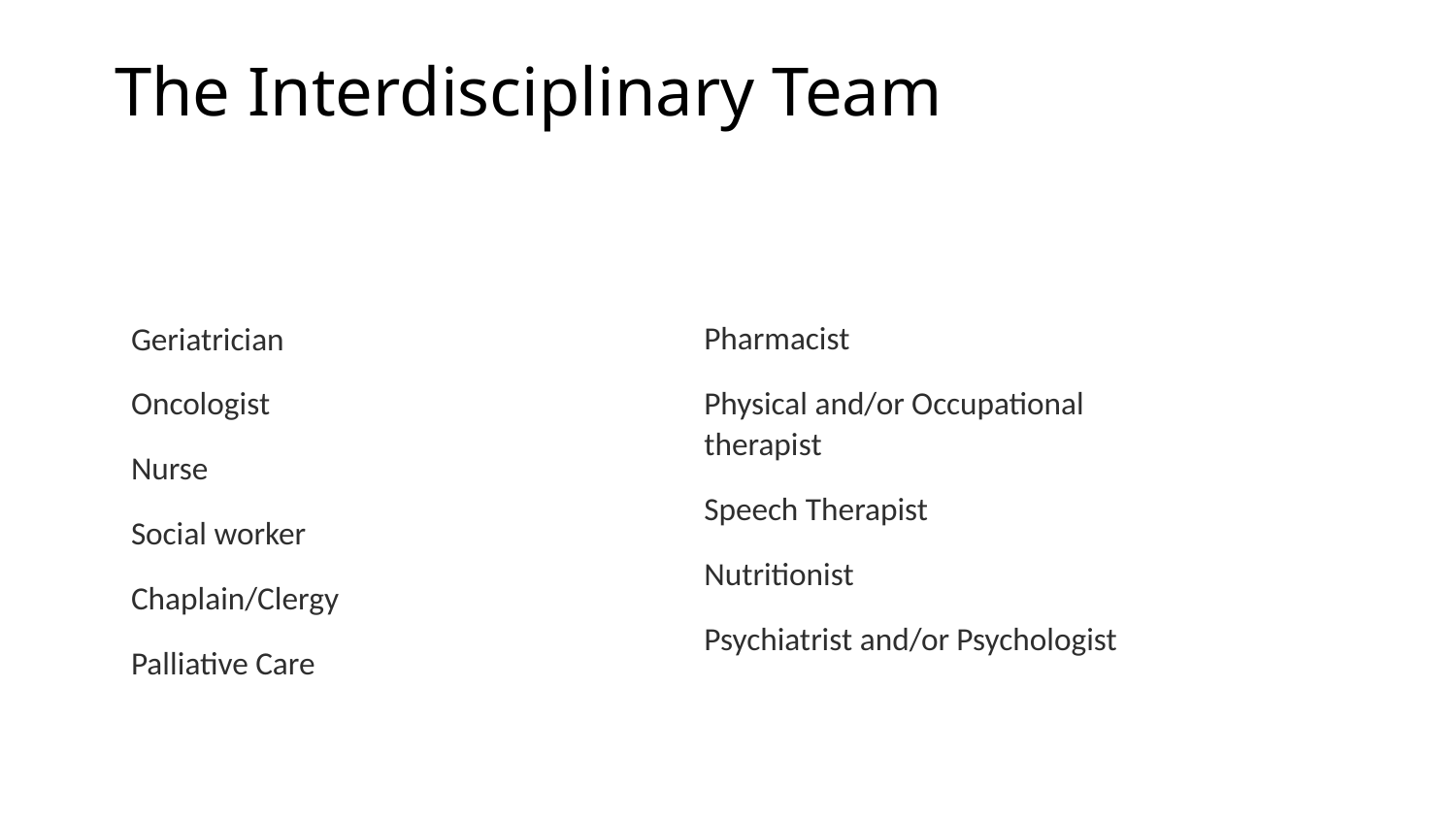

# The Interdisciplinary Team
Geriatrician
Oncologist
Nurse
Social worker
Chaplain/Clergy
Palliative Care
Pharmacist
Physical and/or Occupational therapist
Speech Therapist
Nutritionist
Psychiatrist and/or Psychologist

## Slide 36
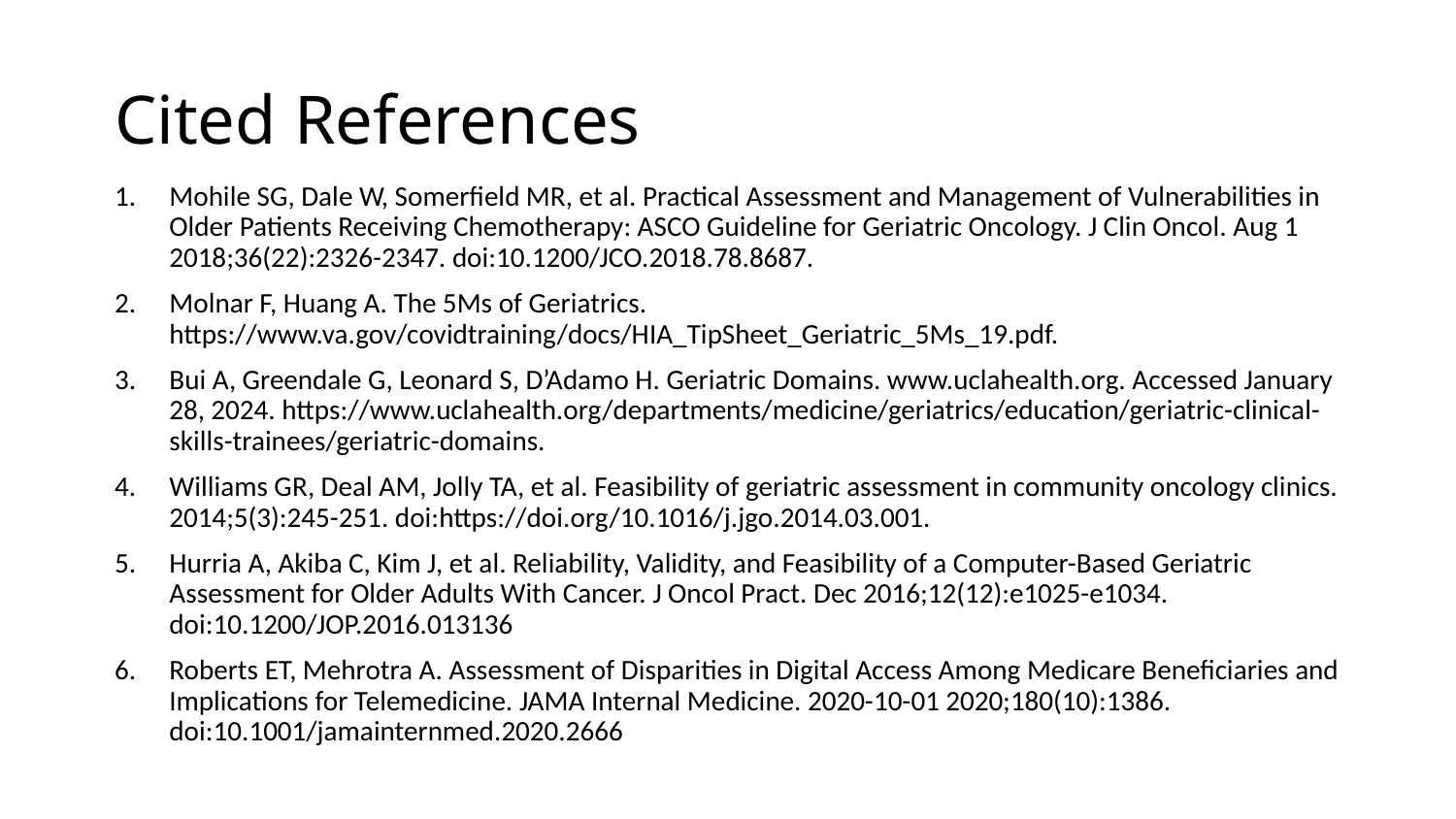

# Cited References
Mohile SG, Dale W, Somerfield MR, et al. Practical Assessment and Management of Vulnerabilities in Older Patients Receiving Chemotherapy: ASCO Guideline for Geriatric Oncology. J Clin Oncol. Aug 1 2018;36(22):2326-2347. doi:10.1200/JCO.2018.78.8687.
Molnar F, Huang A. The 5Ms of Geriatrics. https://www.va.gov/covidtraining/docs/HIA_TipSheet_Geriatric_5Ms_19.pdf.
‌Bui A, Greendale G, Leonard S, D’Adamo H. Geriatric Domains. www.uclahealth.org. Accessed January 28, 2024. https://www.uclahealth.org/departments/medicine/geriatrics/education/geriatric-clinical-skills-trainees/geriatric-domains.
Williams GR, Deal AM, Jolly TA, et al. Feasibility of geriatric assessment in community oncology clinics. 2014;5(3):245-251. doi:https://doi.org/10.1016/j.jgo.2014.03.001.
Hurria A, Akiba C, Kim J, et al. Reliability, Validity, and Feasibility of a Computer-Based Geriatric Assessment for Older Adults With Cancer. J Oncol Pract. Dec 2016;12(12):e1025-e1034. doi:10.1200/JOP.2016.013136
Roberts ET, Mehrotra A. Assessment of Disparities in Digital Access Among Medicare Beneficiaries and Implications for Telemedicine. JAMA Internal Medicine. 2020-10-01 2020;180(10):1386. doi:10.1001/jamainternmed.2020.2666

## Slide 37
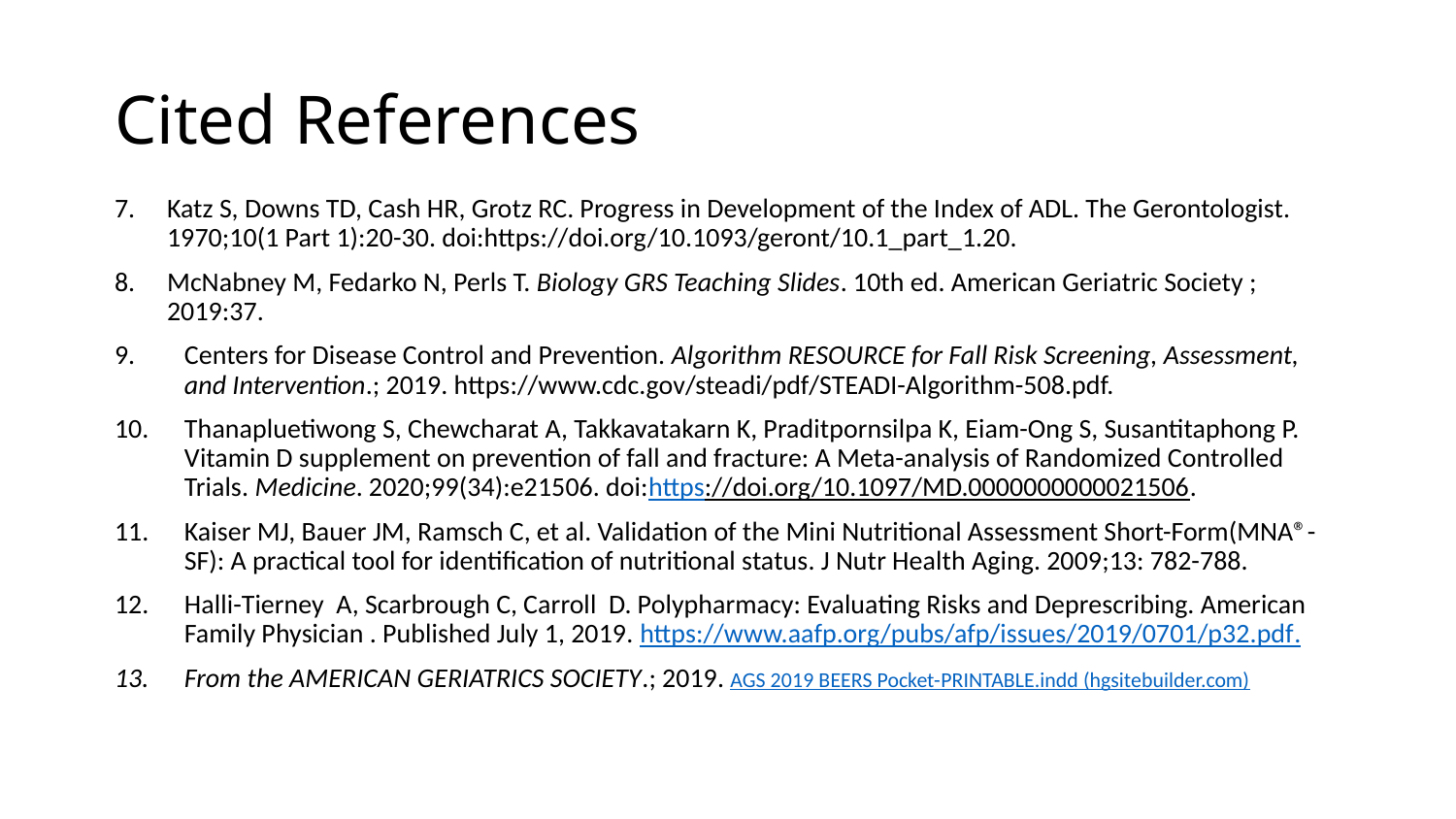

# Cited References
‌Katz S, Downs TD, Cash HR, Grotz RC. Progress in Development of the Index of ADL. The Gerontologist. 1970;10(1 Part 1):20-30. doi:https://doi.org/10.1093/geront/10.1_part_1.20.
‌McNabney M, Fedarko N, Perls T. Biology GRS Teaching Slides. 10th ed. American Geriatric Society ; 2019:37.
Centers for Disease Control and Prevention. Algorithm RESOURCE for Fall Risk Screening, Assessment, and Intervention.; 2019. https://www.cdc.gov/steadi/pdf/STEADI-Algorithm-508.pdf.
Thanapluetiwong S, Chewcharat A, Takkavatakarn K, Praditpornsilpa K, Eiam-Ong S, Susantitaphong P. Vitamin D supplement on prevention of fall and fracture: A Meta-analysis of Randomized Controlled Trials. Medicine. 2020;99(34):e21506. doi:https://doi.org/10.1097/MD.0000000000021506.
Kaiser MJ, Bauer JM, Ramsch C, et al. Validation of the Mini Nutritional Assessment Short-Form(MNA®-SF): A practical tool for identification of nutritional status. J Nutr Health Aging. 2009;13: 782-788.
Halli-Tierney A, Scarbrough C, Carroll D. Polypharmacy: Evaluating Risks and Deprescribing. American Family Physician . Published July 1, 2019. https://www.aafp.org/pubs/afp/issues/2019/0701/p32.pdf.
From the AMERICAN GERIATRICS SOCIETY.; 2019. AGS 2019 BEERS Pocket-PRINTABLE.indd (hgsitebuilder.com)
