# Supplementary material for: An Introductory Course on Geriatric Oncology
Source: MedEdPORTAL. 2024 Nov 14;20:11471. doi: 10.15766/mep_2374-8265.11471 (PMC11561070; doi:10.15766/mep_2374-8265.11471)
Supplement: Supplementary file 1 — Introduction to Geriatric Oncology.pptxThe Comprehensive Geriatric Assessment.pptxGeriatric Screening Tools.pptxBiology of Aging.pptxCancer Therapy in the Older Adult.pptxSummary of Interactive Sessions.docxSession 5 Patient Case 1.docxSession 5 Patient Case 2.docxSession 5 Patient Case 3.docxGeriatric Oncology Knowledge Assessment.docxKnowledge Assessment Answer Key.docxSelf-Perceived Competency Assessment.docxCurriculum Session Assessment.docx [file mep_2374-8265.11471-s001.zip › C. Geriatric Screening Tools.pptx]

## Slide 1
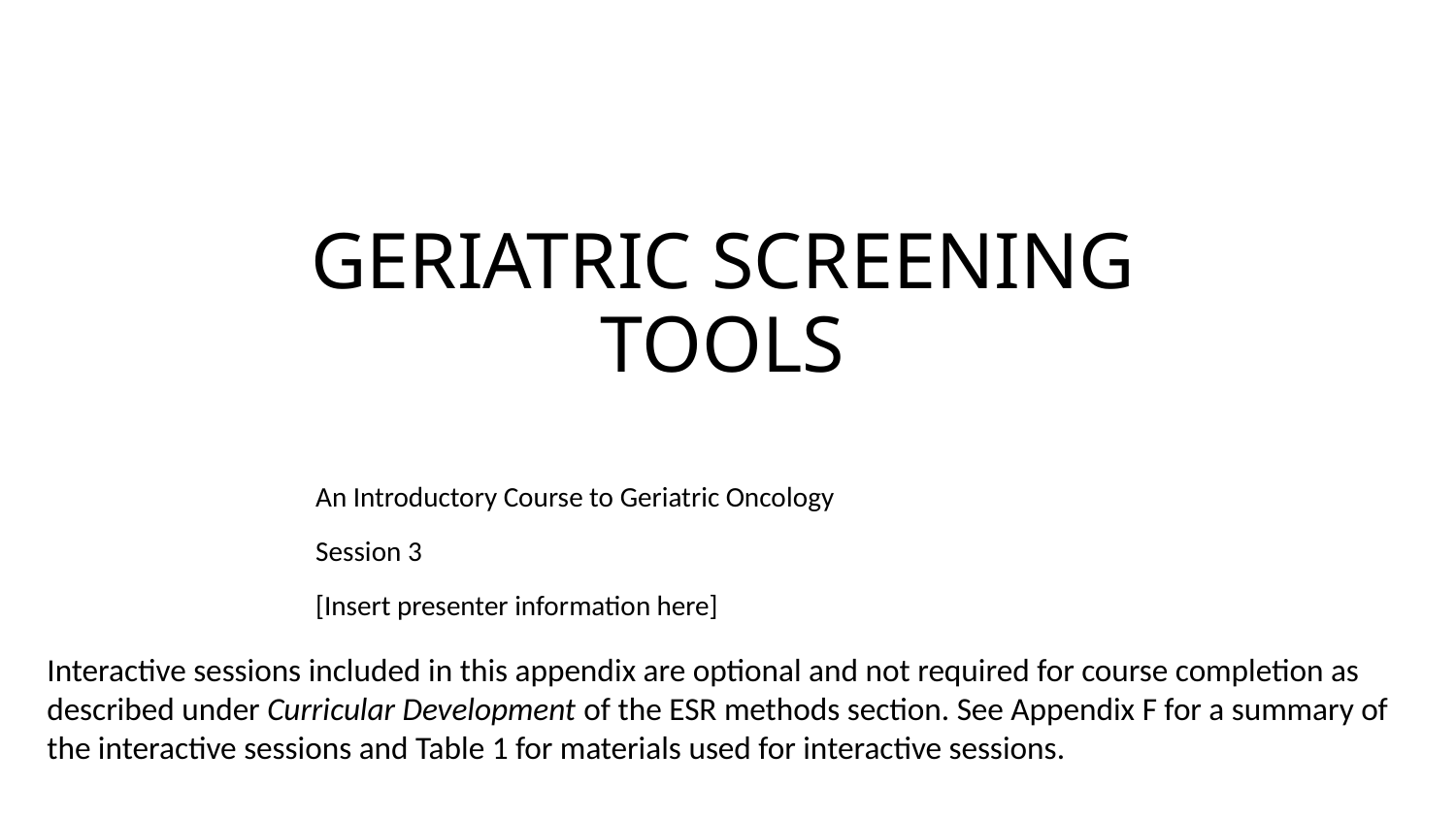

# GERIATRIC SCREENING TOOLS
An Introductory Course to Geriatric Oncology
Session 3
[Insert presenter information here]
Interactive sessions included in this appendix are optional and not required for course completion as described under Curricular Development of the ESR methods section. See Appendix F for a summary of the interactive sessions and Table 1 for materials used for interactive sessions.

## Slide 2
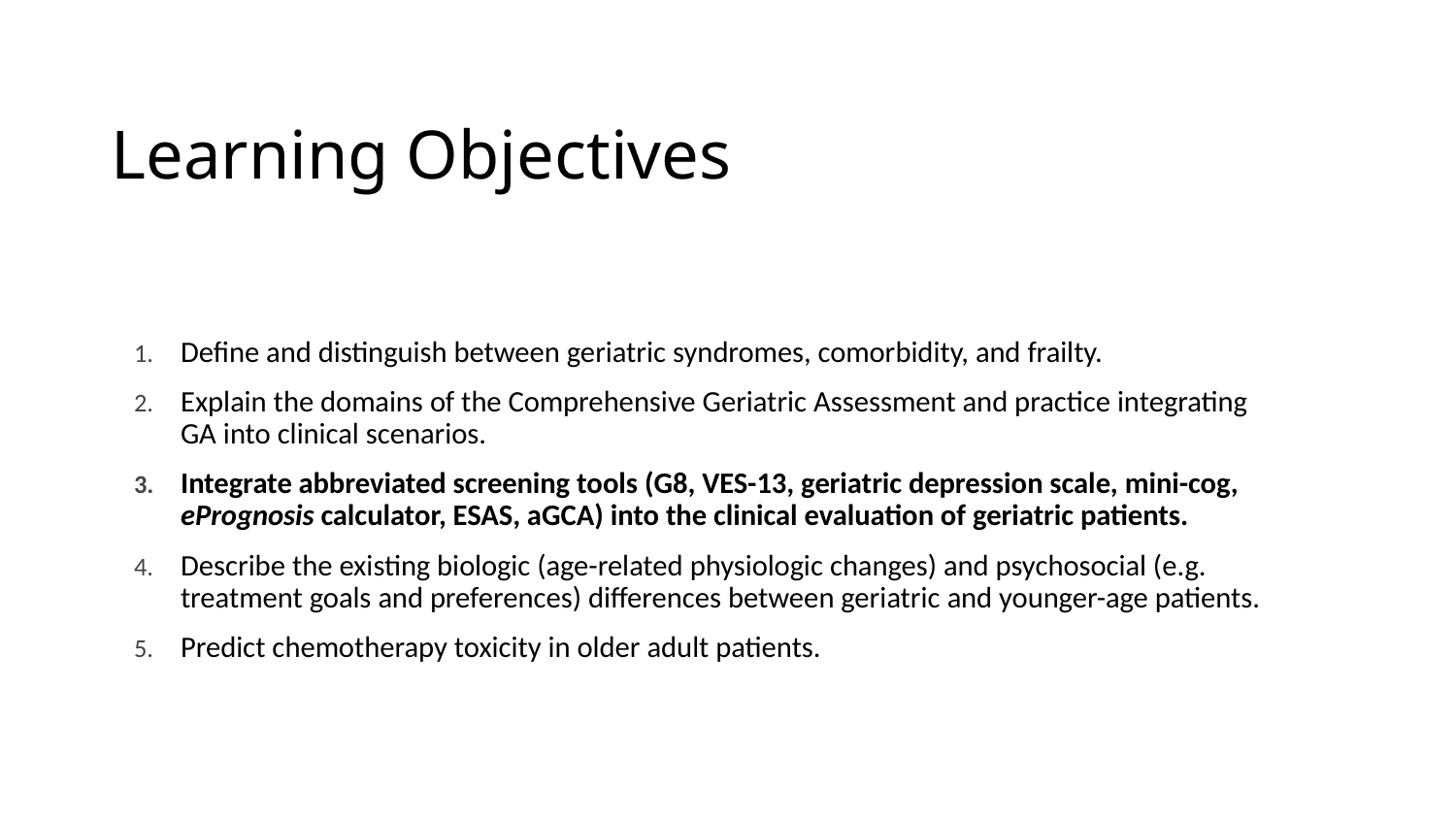

Learning Objectives
Define and distinguish between geriatric syndromes, comorbidity, and frailty. ​
Explain the domains of the Comprehensive Geriatric Assessment and practice integrating GA into clinical scenarios. ​
Integrate abbreviated screening tools (G8, VES-13, geriatric depression scale, mini-cog, ePrognosis calculator, ESAS, aGCA) into the clinical evaluation of geriatric patients.
Describe the existing biologic (age-related physiologic changes) and psychosocial (e.g. treatment goals and preferences) differences between geriatric and younger-age patients. ​​
Predict chemotherapy toxicity in older adult patients.​

## Slide 3
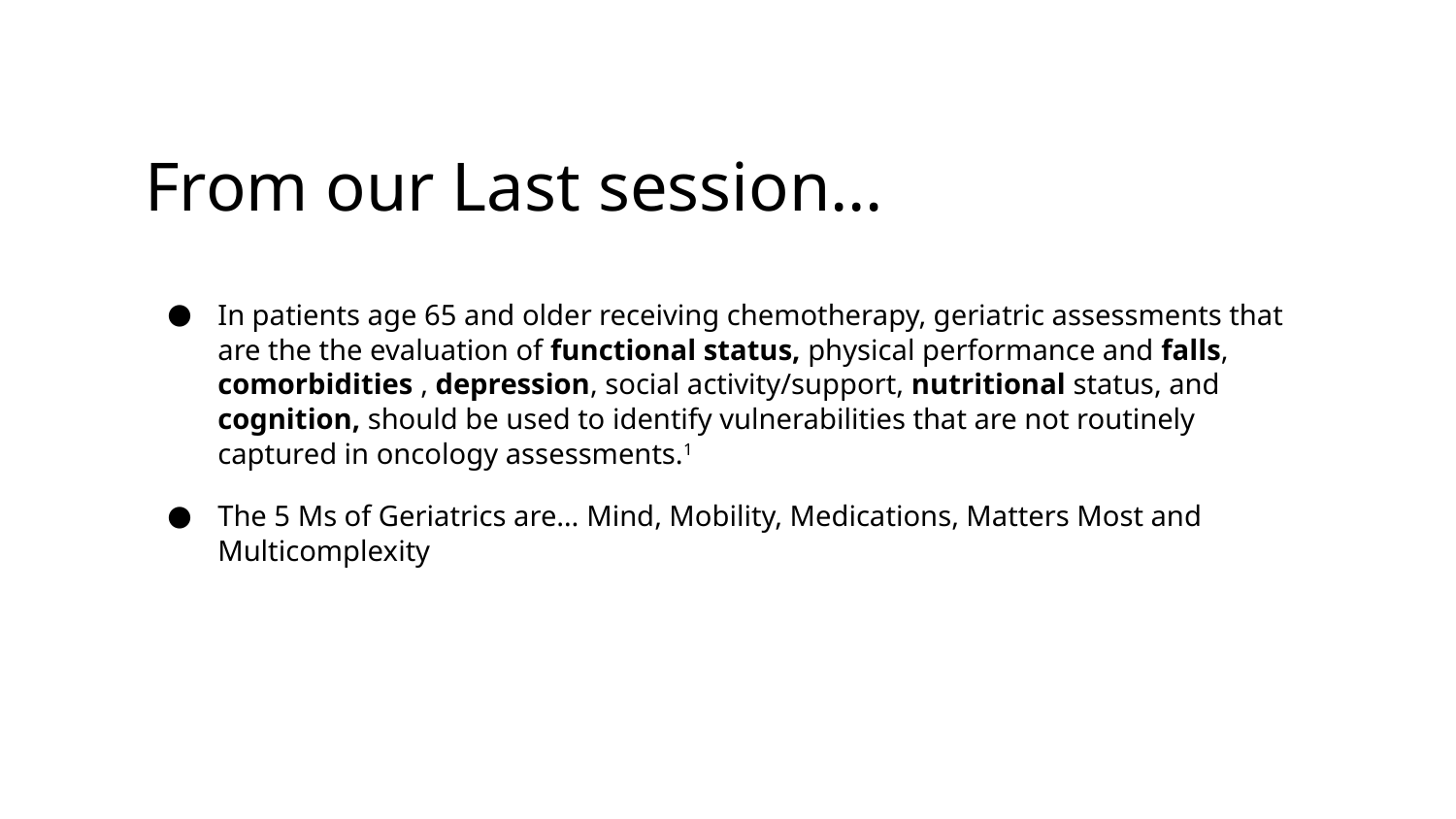

From our Last session…
In patients age 65 and older receiving chemotherapy, geriatric assessments that are the the evaluation of functional status, physical performance and falls, comorbidities , depression, social activity/support, nutritional status, and cognition, should be used to identify vulnerabilities that are not routinely captured in oncology assessments.1
The 5 Ms of Geriatrics are… Mind, Mobility, Medications, Matters Most and Multicomplexity

## Slide 4
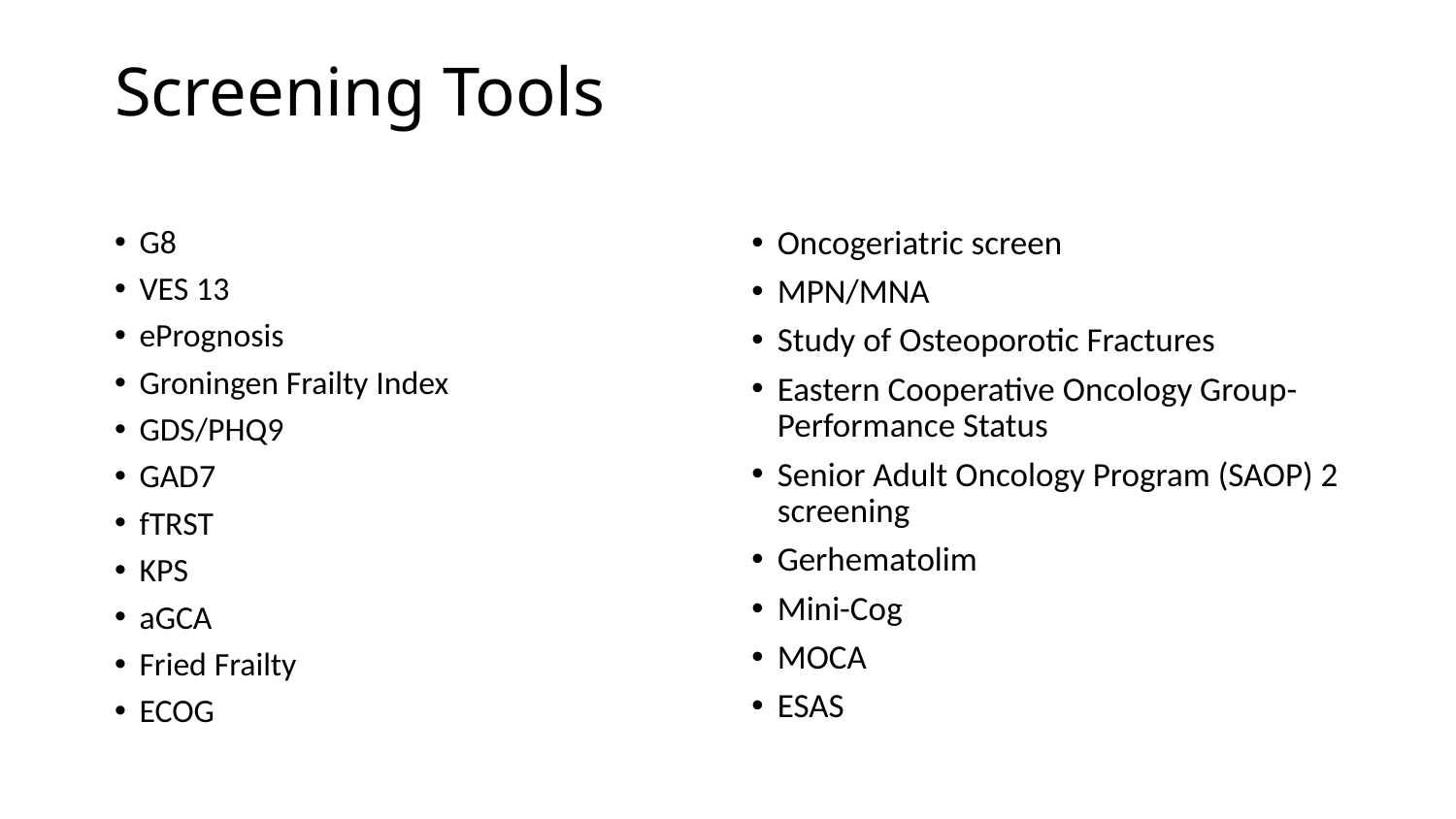

# Screening Tools
G8
VES 13
ePrognosis
Groningen Frailty Index
GDS/PHQ9
GAD7
fTRST
KPS
aGCA
Fried Frailty
ECOG
Oncogeriatric screen
MPN/MNA
Study of Osteoporotic Fractures
Eastern Cooperative Oncology Group-Performance Status
Senior Adult Oncology Program (SAOP) 2 screening
Gerhematolim
Mini-Cog
MOCA
ESAS

## Slide 5
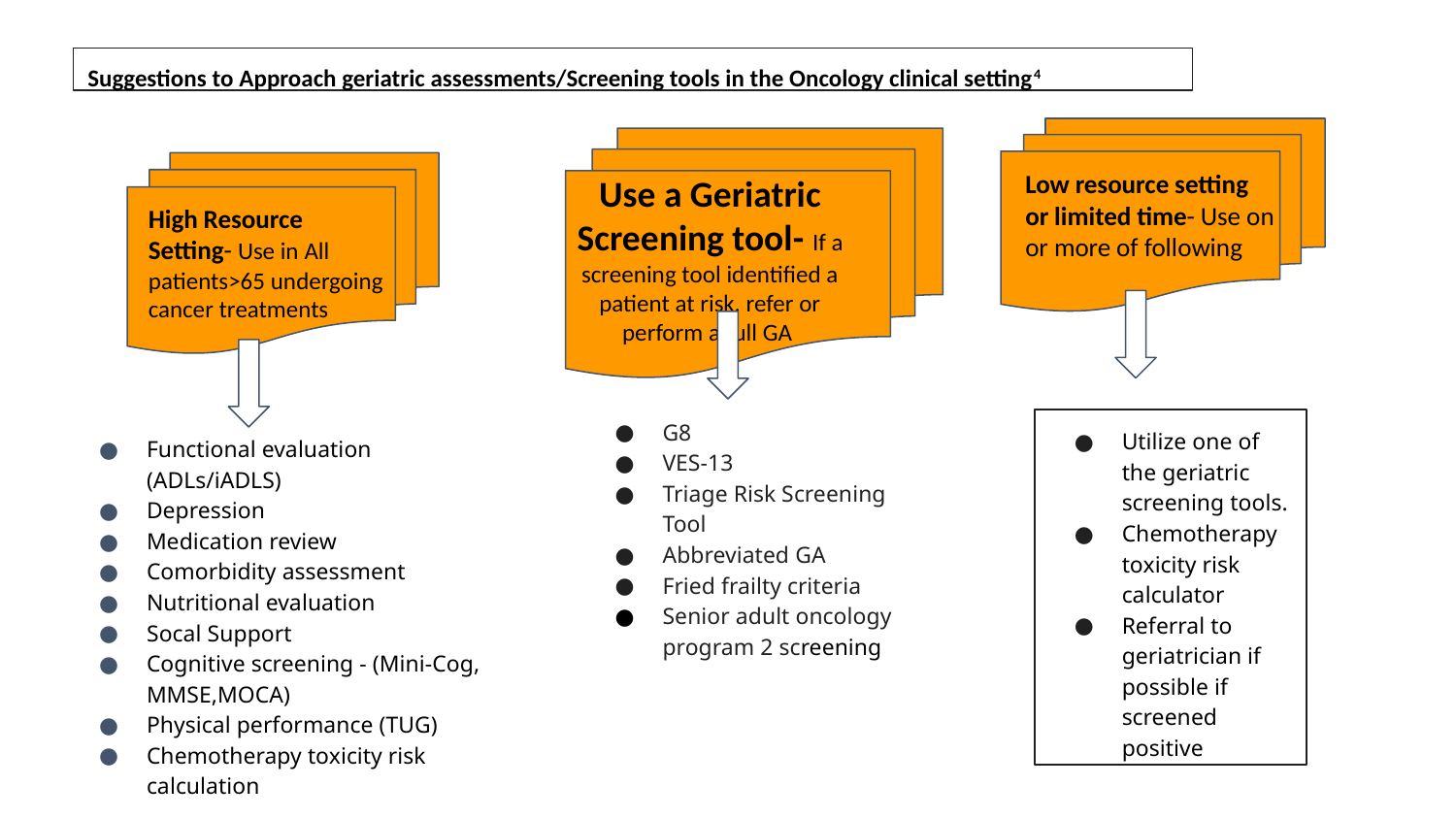

Suggestions to Approach geriatric assessments/Screening tools in the Oncology clinical setting4
Low resource setting or limited time- Use on or more of following
Use a Geriatric Screening tool- If a screening tool identified a patient at risk, refer or perform a full GA
High Resource Setting- Use in All patients>65 undergoing cancer treatments
G8
VES-13
Triage Risk Screening Tool
Abbreviated GA
Fried frailty criteria
Senior adult oncology program 2 screening
Utilize one of the geriatric screening tools.
Chemotherapy toxicity risk calculator
Referral to geriatrician if possible if screened positive
Functional evaluation (ADLs/iADLS)
Depression
Medication review
Comorbidity assessment
Nutritional evaluation
Socal Support
Cognitive screening - (Mini-Cog, MMSE,MOCA)
Physical performance (TUG)
Chemotherapy toxicity risk calculation

## Slide 6
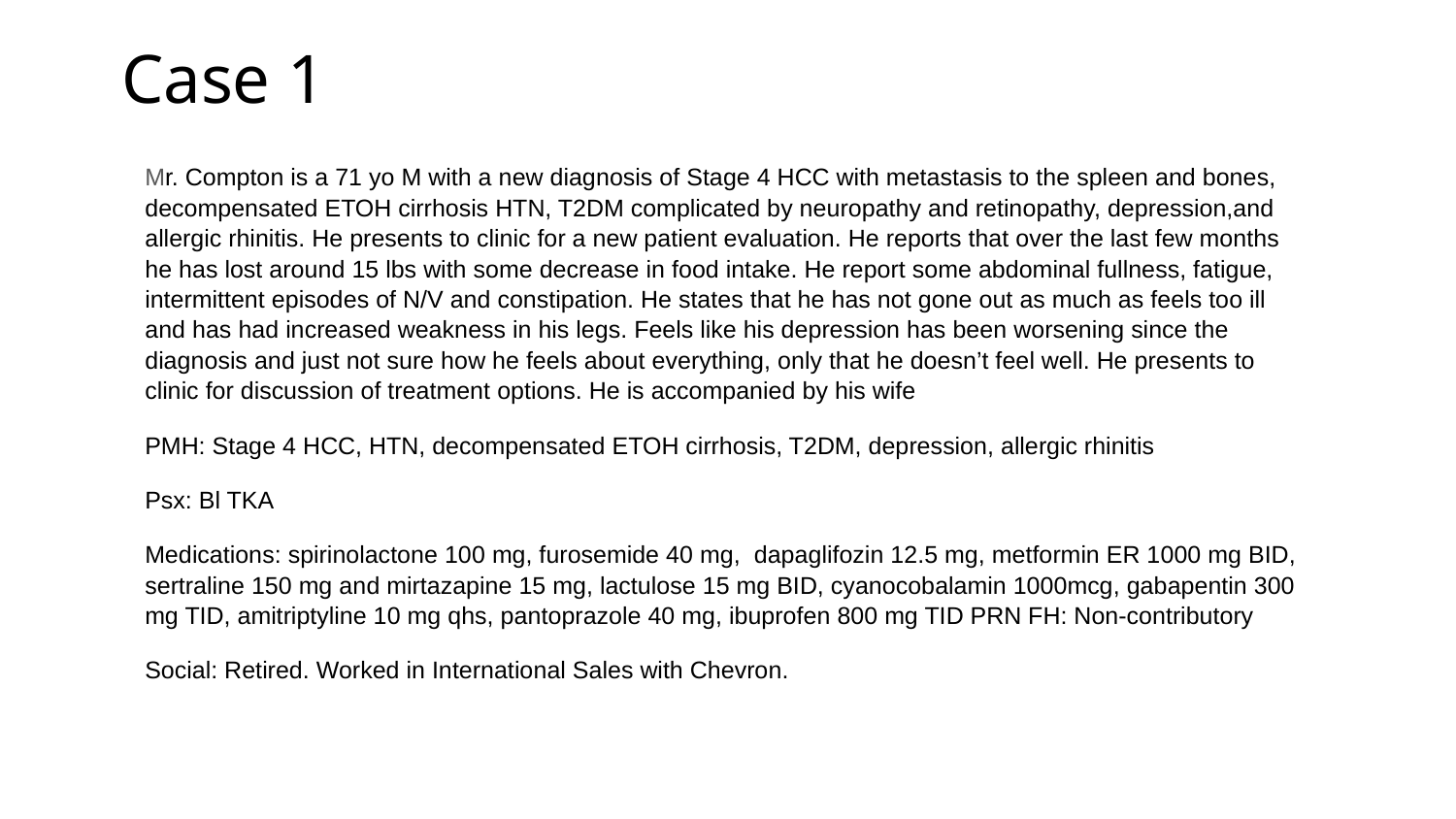

Case 1
Mr. Compton is a 71 yo M with a new diagnosis of Stage 4 HCC with metastasis to the spleen and bones, decompensated ETOH cirrhosis HTN, T2DM complicated by neuropathy and retinopathy, depression,and allergic rhinitis. He presents to clinic for a new patient evaluation. He reports that over the last few months he has lost around 15 lbs with some decrease in food intake. He report some abdominal fullness, fatigue, intermittent episodes of N/V and constipation. He states that he has not gone out as much as feels too ill and has had increased weakness in his legs. Feels like his depression has been worsening since the diagnosis and just not sure how he feels about everything, only that he doesn’t feel well. He presents to clinic for discussion of treatment options. He is accompanied by his wife
PMH: Stage 4 HCC, HTN, decompensated ETOH cirrhosis, T2DM, depression, allergic rhinitis
Psx: Bl TKA
Medications: spirinolactone 100 mg, furosemide 40 mg, dapaglifozin 12.5 mg, metformin ER 1000 mg BID, sertraline 150 mg and mirtazapine 15 mg, lactulose 15 mg BID, cyanocobalamin 1000mcg, gabapentin 300 mg TID, amitriptyline 10 mg qhs, pantoprazole 40 mg, ibuprofen 800 mg TID PRN FH: Non-contributory
Social: Retired. Worked in International Sales with Chevron.

## Slide 7
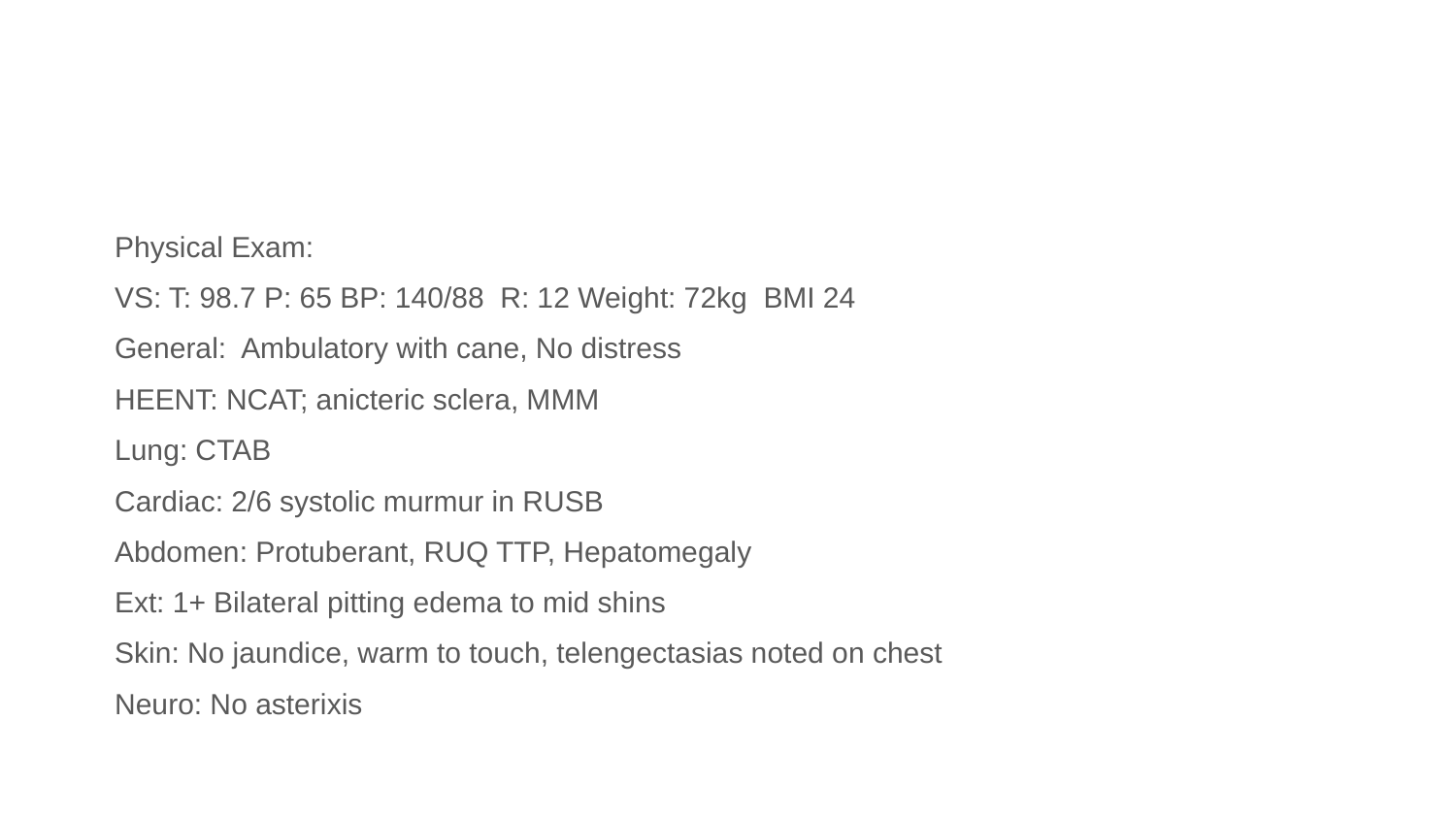

Physical Exam:
VS: T: 98.7 P: 65 BP: 140/88 R: 12 Weight: 72kg BMI 24
General: Ambulatory with cane, No distress
HEENT: NCAT; anicteric sclera, MMM
Lung: CTAB
Cardiac: 2/6 systolic murmur in RUSB
Abdomen: Protuberant, RUQ TTP, Hepatomegaly
Ext: 1+ Bilateral pitting edema to mid shins
Skin: No jaundice, warm to touch, telengectasias noted on chest
Neuro: No asterixis

## Slide 8
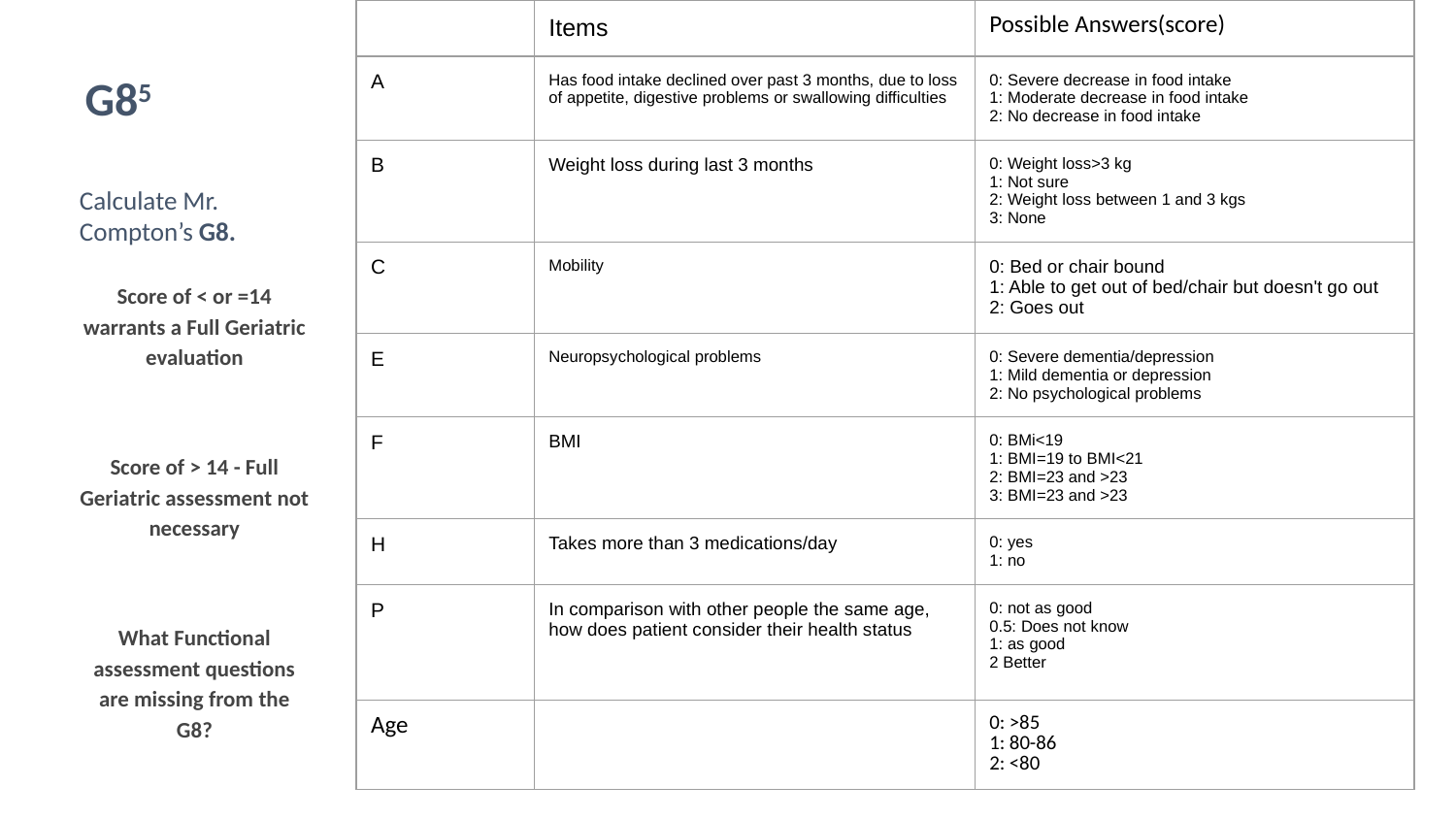

| | Items | Possible Answers(score) |
| --- | --- | --- |
| A | Has food intake declined over past 3 months, due to loss of appetite, digestive problems or swallowing difficulties | 0: Severe decrease in food intake 1: Moderate decrease in food intake 2: No decrease in food intake |
| B | Weight loss during last 3 months | 0: Weight loss>3 kg 1: Not sure 2: Weight loss between 1 and 3 kgs 3: None |
| C | Mobility | 0: Bed or chair bound 1: Able to get out of bed/chair but doesn't go out 2: Goes out |
| E | Neuropsychological problems | 0: Severe dementia/depression 1: Mild dementia or depression 2: No psychological problems |
| F | BMI | 0: BMi<19 1: BMI=19 to BMI<21 2: BMI=23 and >23 3: BMI=23 and >23 |
| H | Takes more than 3 medications/day | 0: yes 1: no |
| P | In comparison with other people the same age, how does patient consider their health status | 0: not as good 0.5: Does not know 1: as good 2 Better |
| Age | | 0: >85 1: 80-86 2: <80 |
G85
Calculate Mr. Compton’s G8.
Score of < or =14 warrants a Full Geriatric evaluation
Score of > 14 - Full Geriatric assessment not necessary
What Functional assessment questions are missing from the G8?

## Slide 9
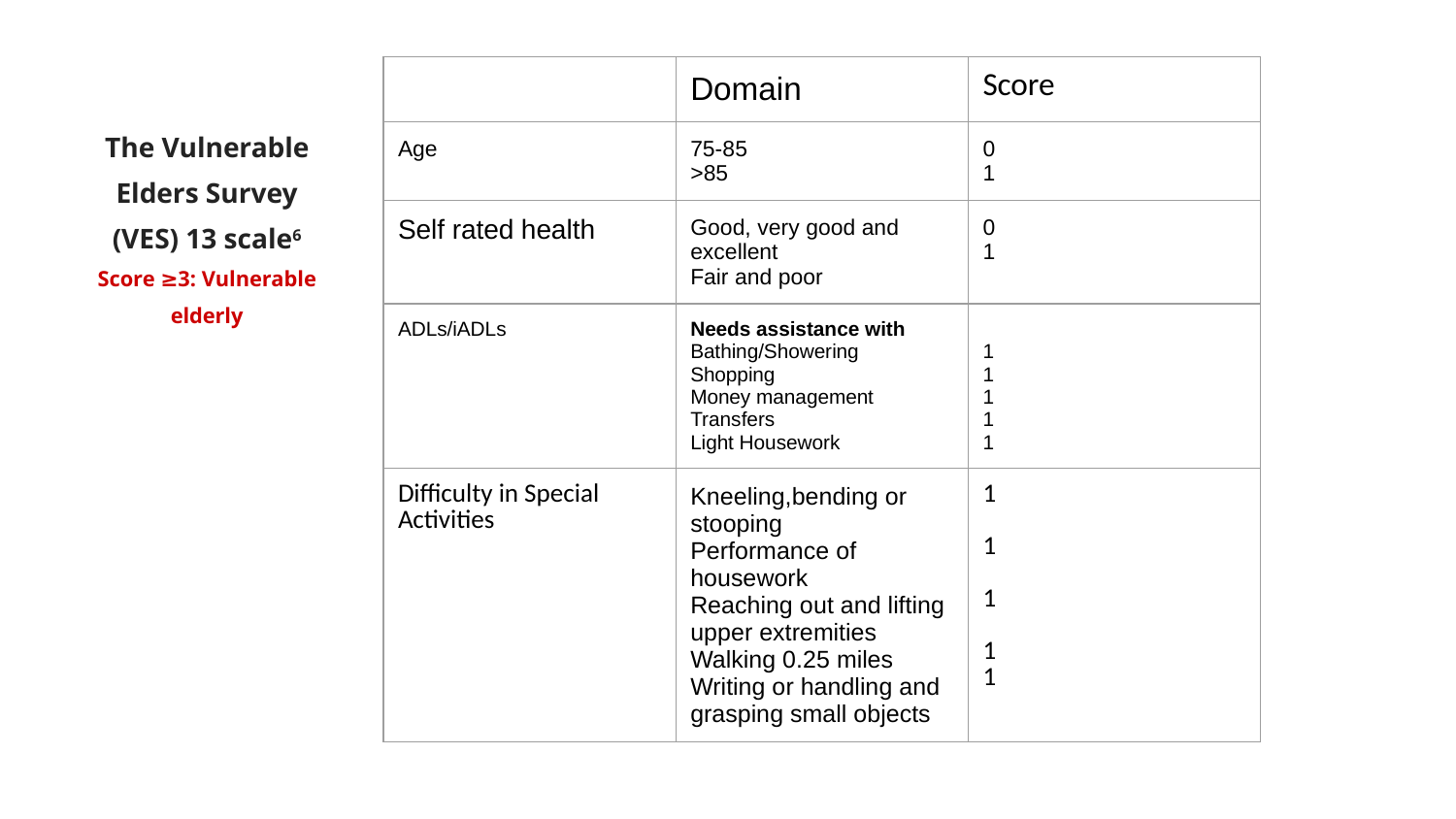

| | Domain | Score |
| --- | --- | --- |
| Age | 75-85 >85 | 0 1 |
| Self rated health | Good, very good and excellent Fair and poor | 0 1 |
| ADLs/iADLs | Needs assistance with Bathing/Showering Shopping Money management Transfers Light Housework | 1 1 1 1 1 |
| Difficulty in Special Activities | Kneeling,bending or stooping Performance of housework Reaching out and lifting upper extremities Walking 0.25 miles Writing or handling and grasping small objects | 1 1 1 1 1 |
The Vulnerable Elders Survey (VES) 13 scale6
Score ≥3: Vulnerable elderly

## Slide 10
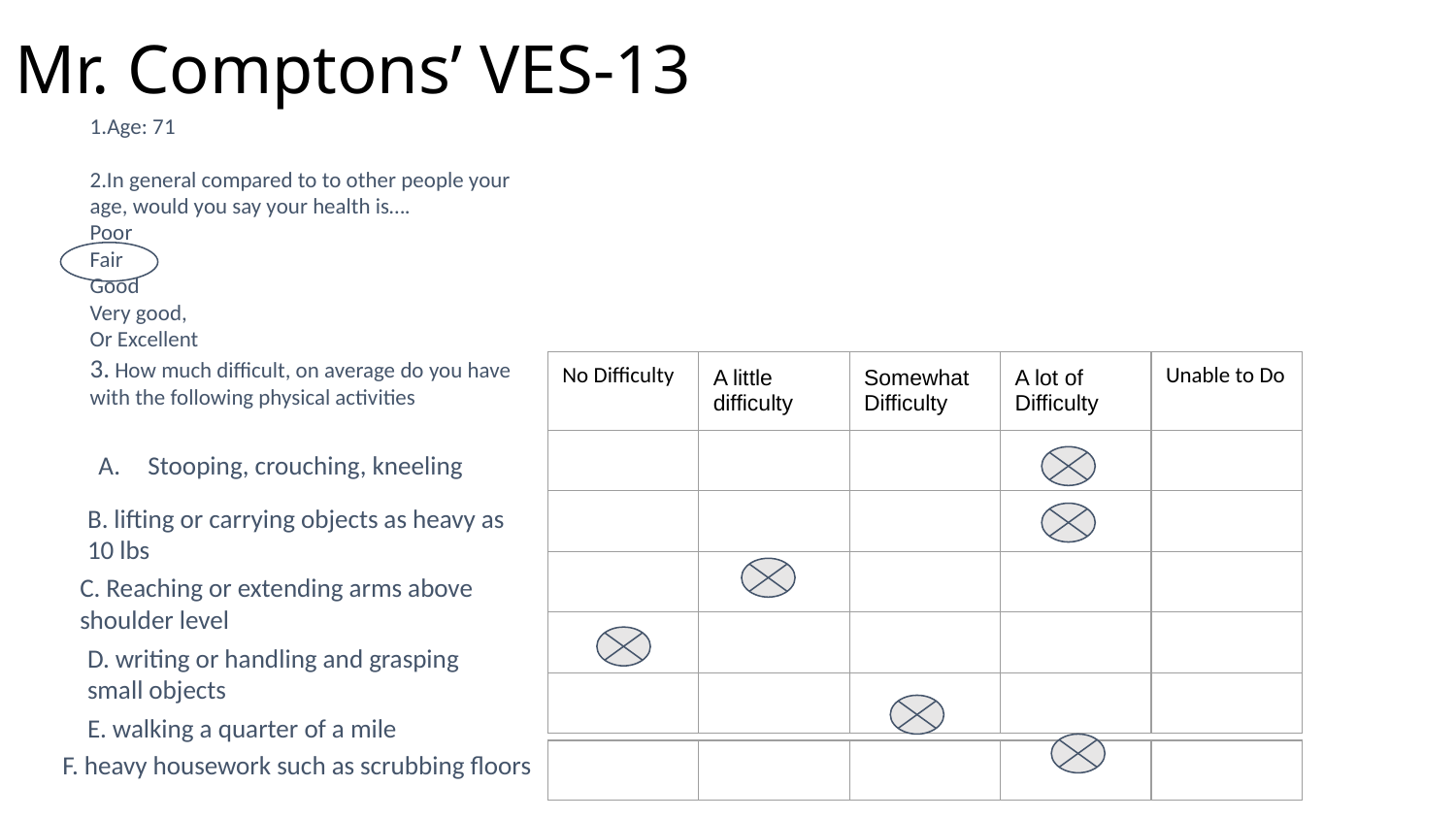

Mr. Comptons’ VES-13
1.Age: 71
2.In general compared to to other people your age, would you say your health is….
Poor
Fair
Good
Very good,
Or Excellent
3. How much difficult, on average do you have with the following physical activities
| No Difficulty | A little difficulty | Somewhat Difficulty | A lot of Difficulty | Unable to Do |
| --- | --- | --- | --- | --- |
| | | | | |
| | | | | |
| | | | | |
| | | | | |
| | | | | |
Stooping, crouching, kneeling
B. lifting or carrying objects as heavy as 10 lbs
C. Reaching or extending arms above shoulder level
D. writing or handling and grasping small objects
E. walking a quarter of a mile
F. heavy housework such as scrubbing floors
| | | | | |
| --- | --- | --- | --- | --- |

## Slide 11
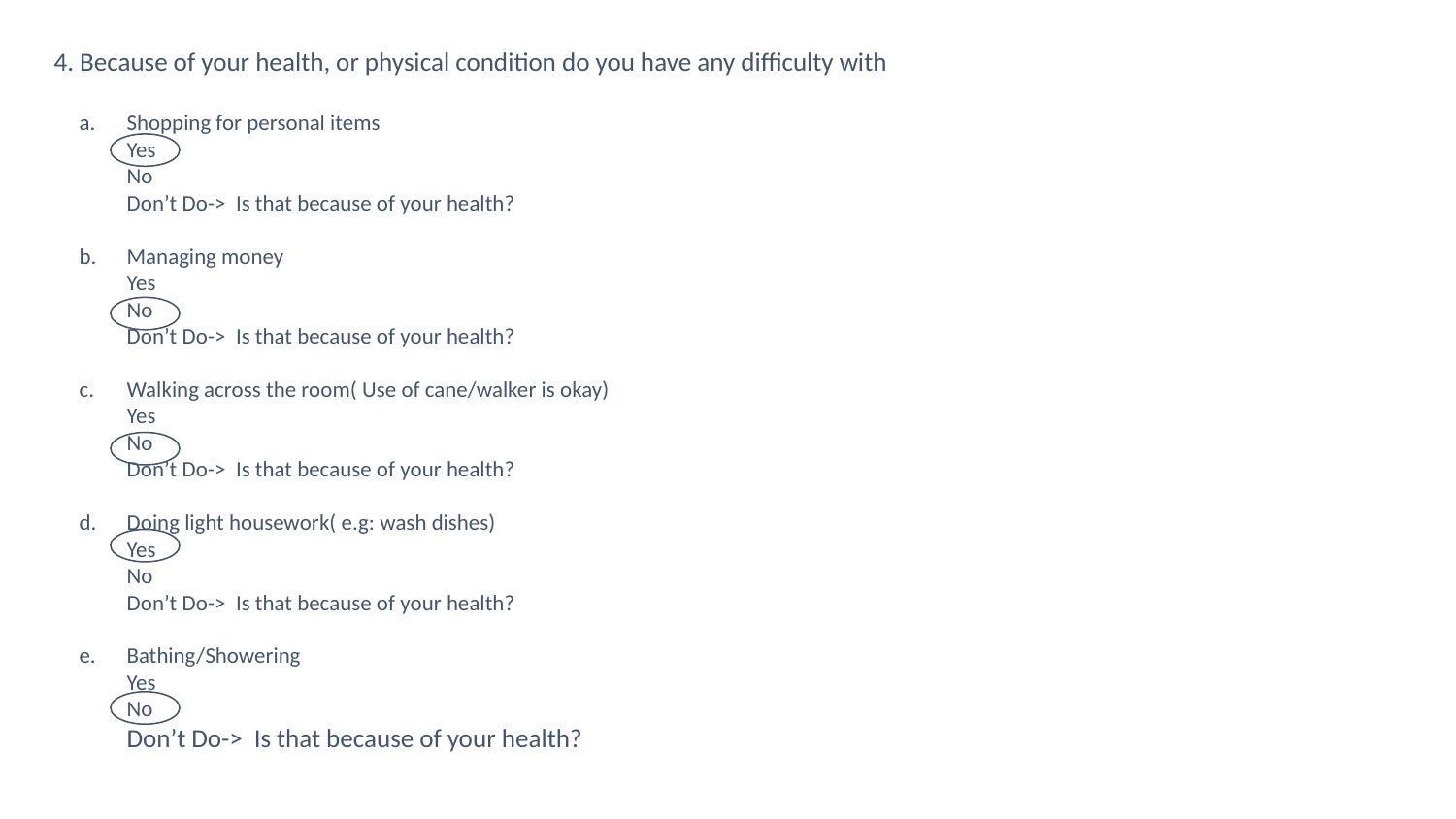

4. Because of your health, or physical condition do you have any difficulty with
Shopping for personal items
Yes
No
Don’t Do-> Is that because of your health?
Managing money
Yes
No
Don’t Do-> Is that because of your health?
Walking across the room( Use of cane/walker is okay)
Yes
No
Don’t Do-> Is that because of your health?
Doing light housework( e.g: wash dishes)
Yes
No
Don’t Do-> Is that because of your health?
Bathing/Showering
Yes
No
Don’t Do-> Is that because of your health?

## Slide 12
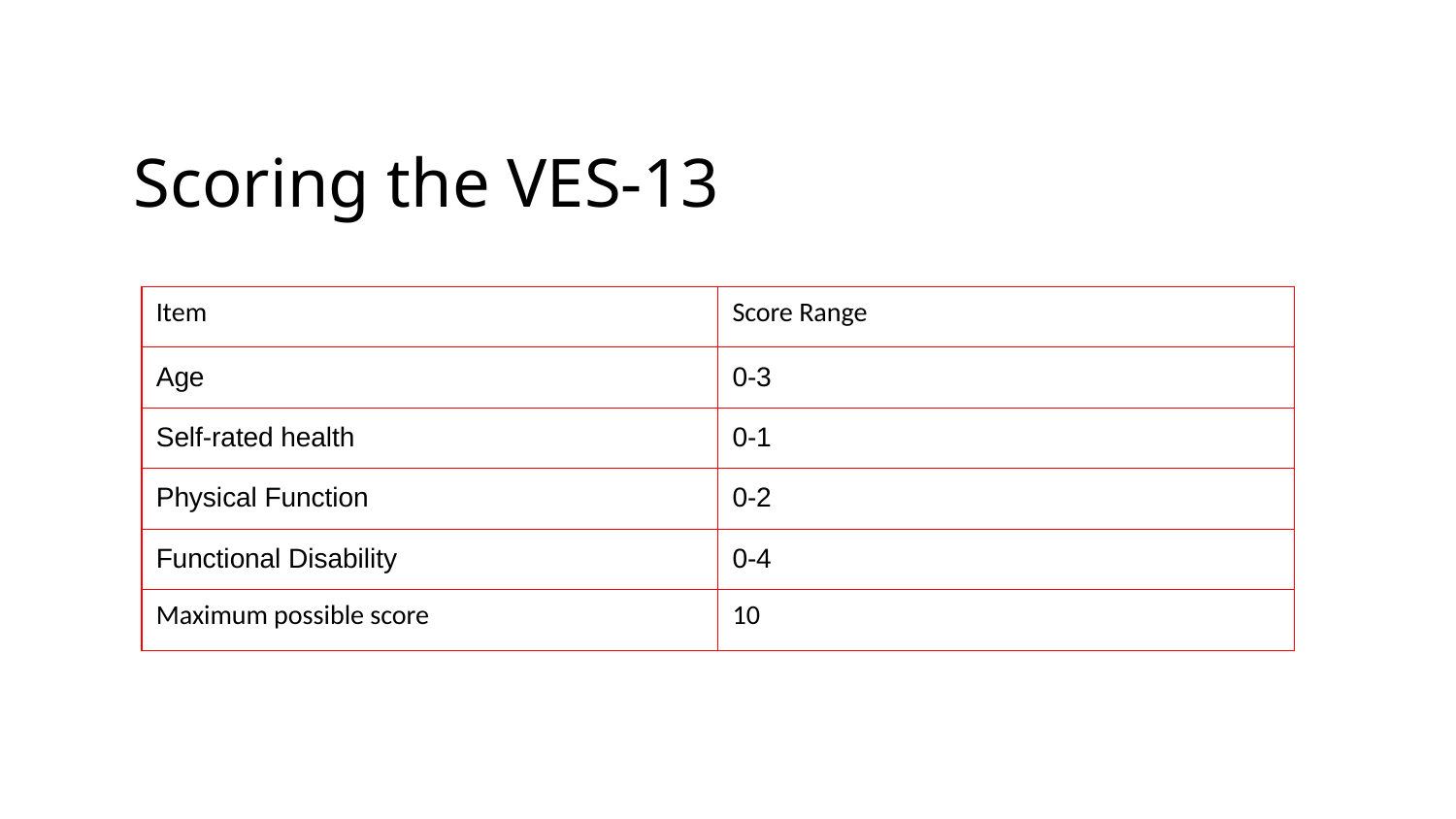

Scoring the VES-13
| Item | Score Range |
| --- | --- |
| Age | 0-3 |
| Self-rated health | 0-1 |
| Physical Function | 0-2 |
| Functional Disability | 0-4 |
| Maximum possible score | 10 |

## Slide 13
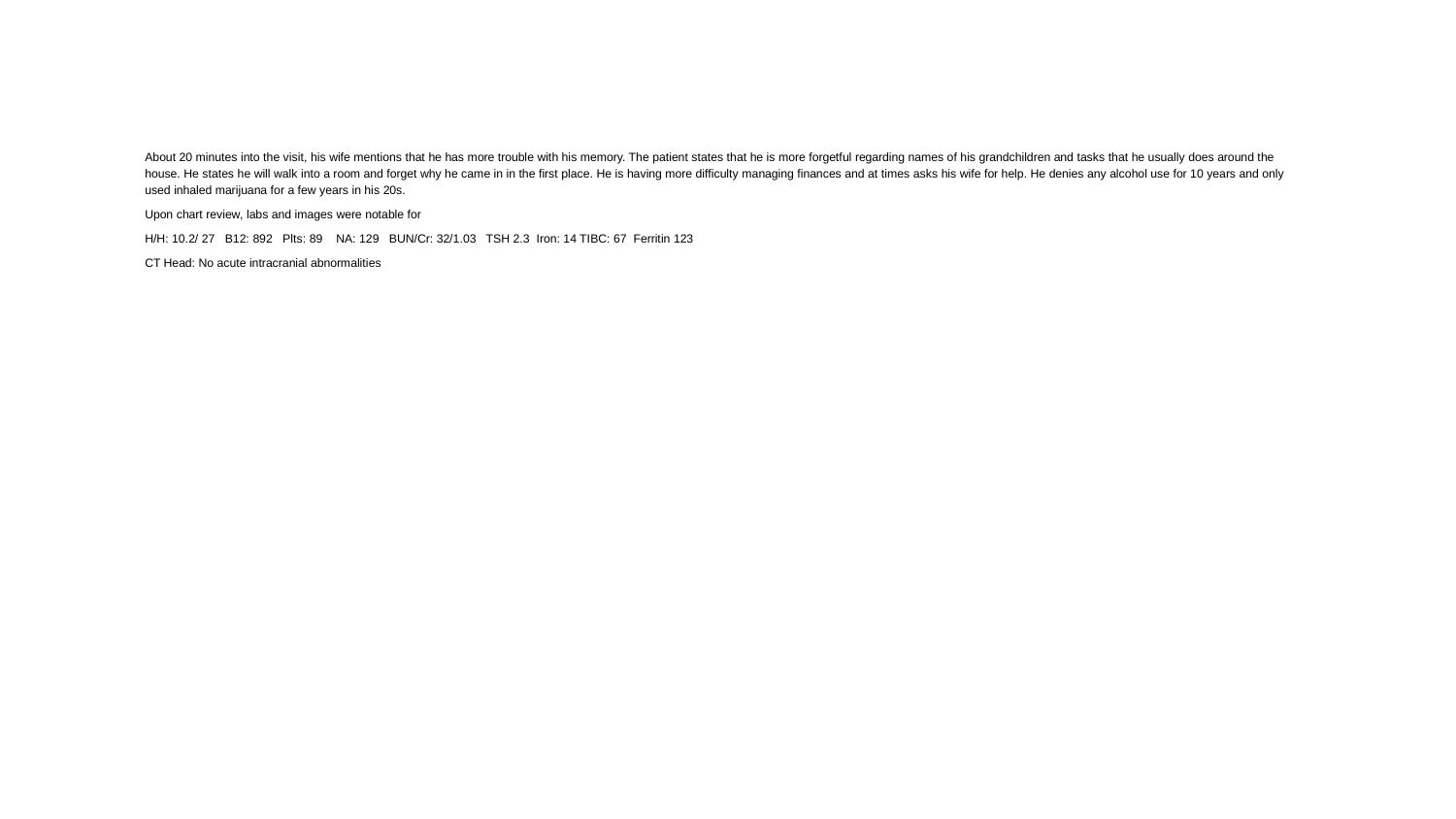

About 20 minutes into the visit, his wife mentions that he has more trouble with his memory. The patient states that he is more forgetful regarding names of his grandchildren and tasks that he usually does around the house. He states he will walk into a room and forget why he came in in the first place. He is having more difficulty managing finances and at times asks his wife for help. He denies any alcohol use for 10 years and only used inhaled marijuana for a few years in his 20s.
Upon chart review, labs and images were notable for
H/H: 10.2/ 27 B12: 892 Plts: 89 NA: 129 BUN/Cr: 32/1.03 TSH 2.3 Iron: 14 TIBC: 67 Ferritin 123
CT Head: No acute intracranial abnormalities

## Slide 14
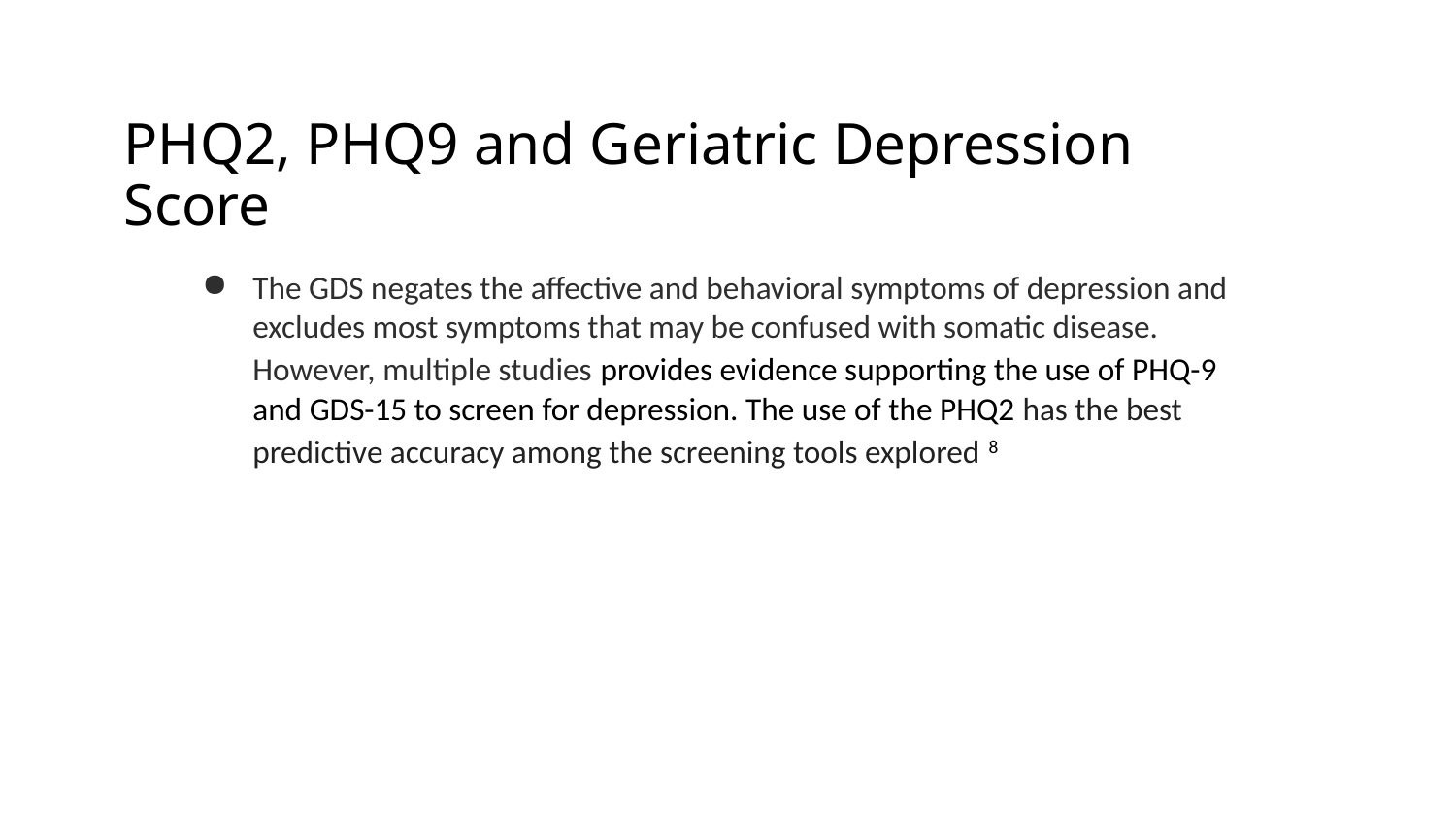

PHQ2, PHQ9 and Geriatric Depression Score
The GDS negates the affective and behavioral symptoms of depression and excludes most symptoms that may be confused with somatic disease. However, multiple studies provides evidence supporting the use of PHQ-9 and GDS-15 to screen for depression. The use of the PHQ2 has the best predictive accuracy among the screening tools explored 8

## Slide 15
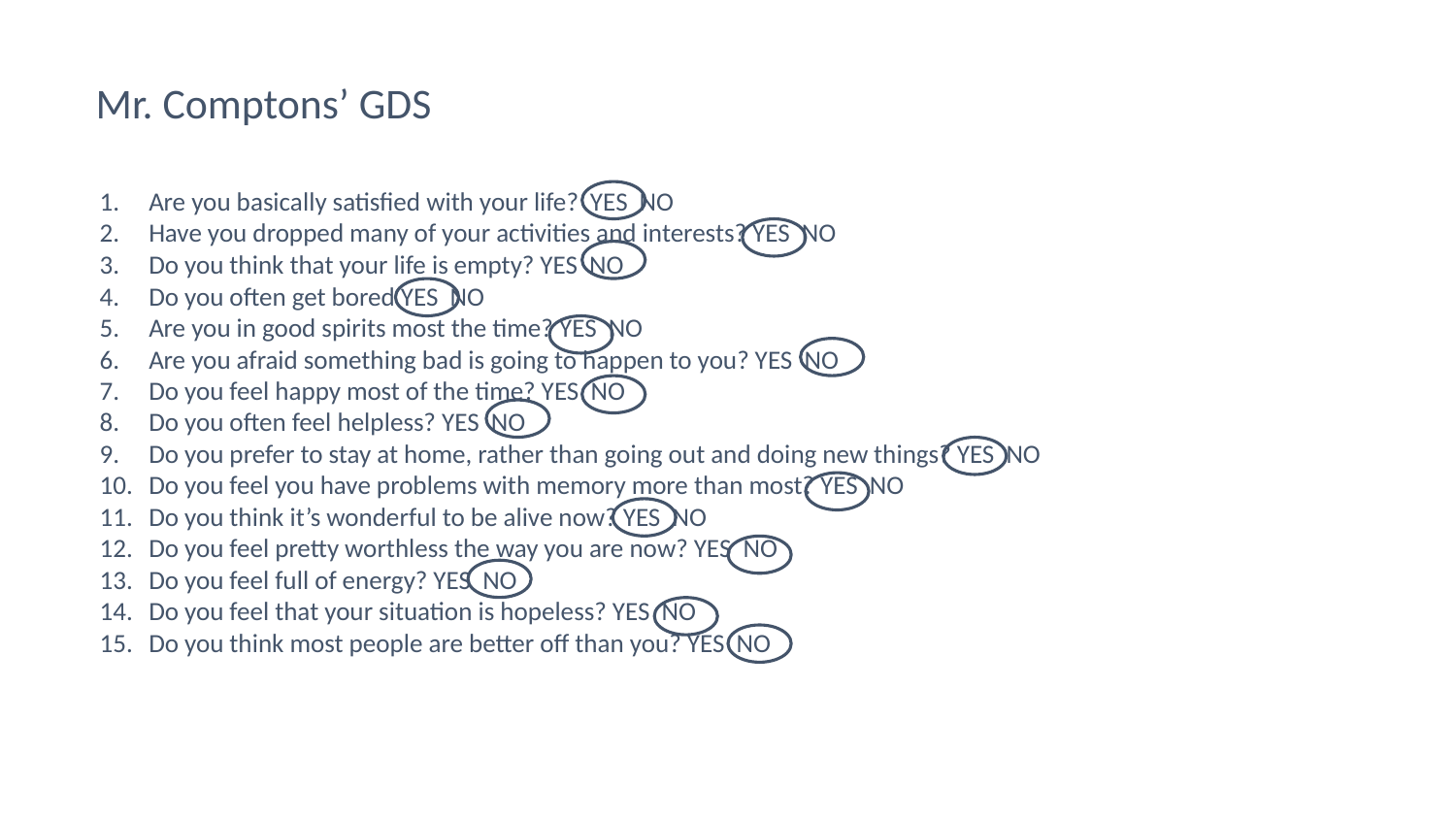

Mr. Comptons’ GDS
Are you basically satisfied with your life? YES NO
Have you dropped many of your activities and interests? YES NO
Do you think that your life is empty? YES NO
Do you often get bored YES NO
Are you in good spirits most the time? YES NO
Are you afraid something bad is going to happen to you? YES NO
Do you feel happy most of the time? YES NO
Do you often feel helpless? YES NO
Do you prefer to stay at home, rather than going out and doing new things? YES NO
Do you feel you have problems with memory more than most? YES NO
Do you think it’s wonderful to be alive now? YES NO
Do you feel pretty worthless the way you are now? YES NO
Do you feel full of energy? YES NO
Do you feel that your situation is hopeless? YES NO
Do you think most people are better off than you? YES NO

## Slide 16
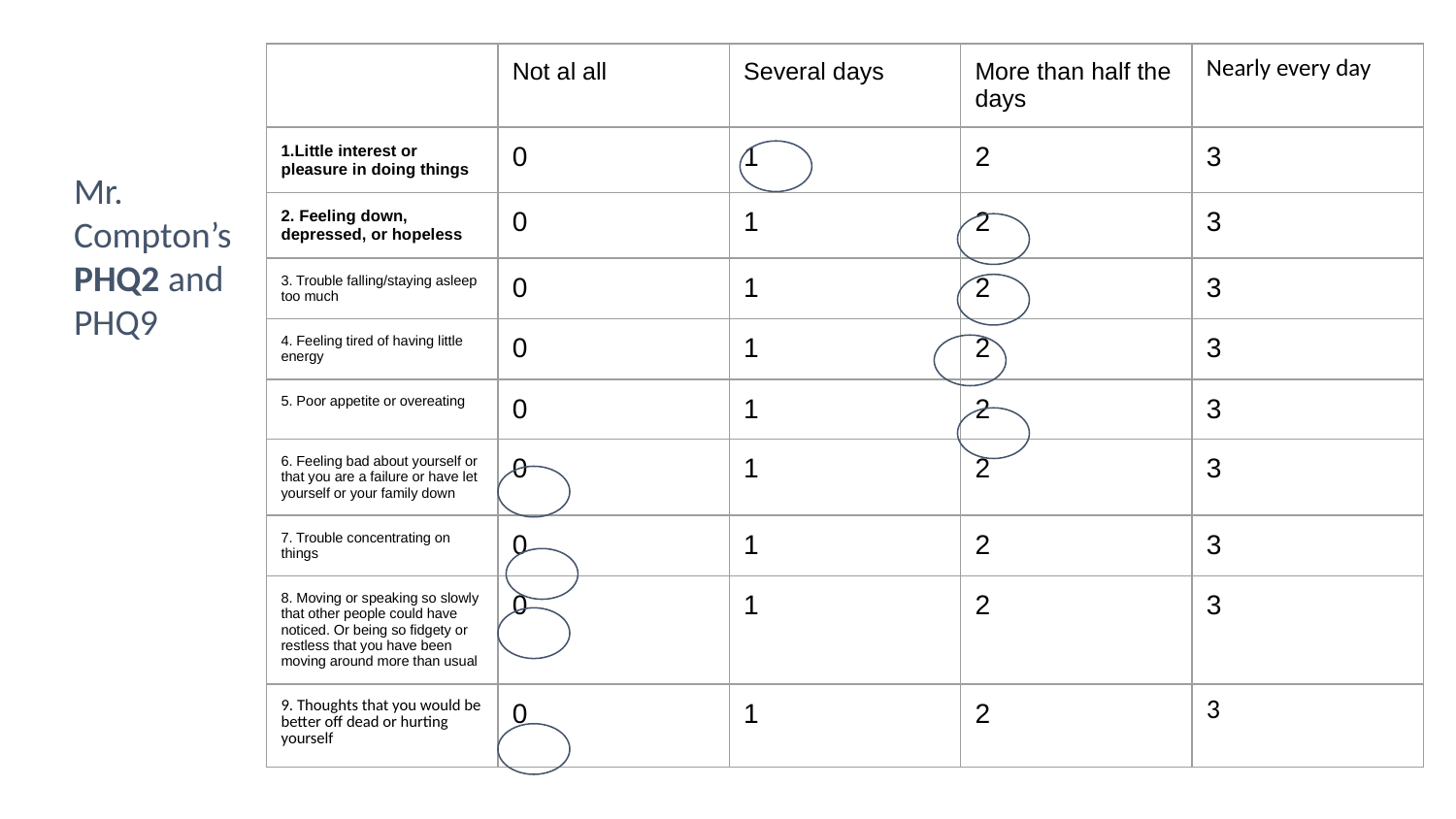

| | Not al all | Several days | More than half the days | Nearly every day |
| --- | --- | --- | --- | --- |
| 1.Little interest or pleasure in doing things | 0 | 1 | 2 | 3 |
| 2. Feeling down, depressed, or hopeless | 0 | 1 | 2 | 3 |
| 3. Trouble falling/staying asleep too much | 0 | 1 | 2 | 3 |
| 4. Feeling tired of having little energy | 0 | 1 | 2 | 3 |
| 5. Poor appetite or overeating | 0 | 1 | 2 | 3 |
| 6. Feeling bad about yourself or that you are a failure or have let yourself or your family down | 0 | 1 | 2 | 3 |
| 7. Trouble concentrating on things | 0 | 1 | 2 | 3 |
| 8. Moving or speaking so slowly that other people could have noticed. Or being so fidgety or restless that you have been moving around more than usual | 0 | 1 | 2 | 3 |
| 9. Thoughts that you would be better off dead or hurting yourself | 0 | 1 | 2 | 3 |
Mr. Compton’s PHQ2 and PHQ9

## Slide 17
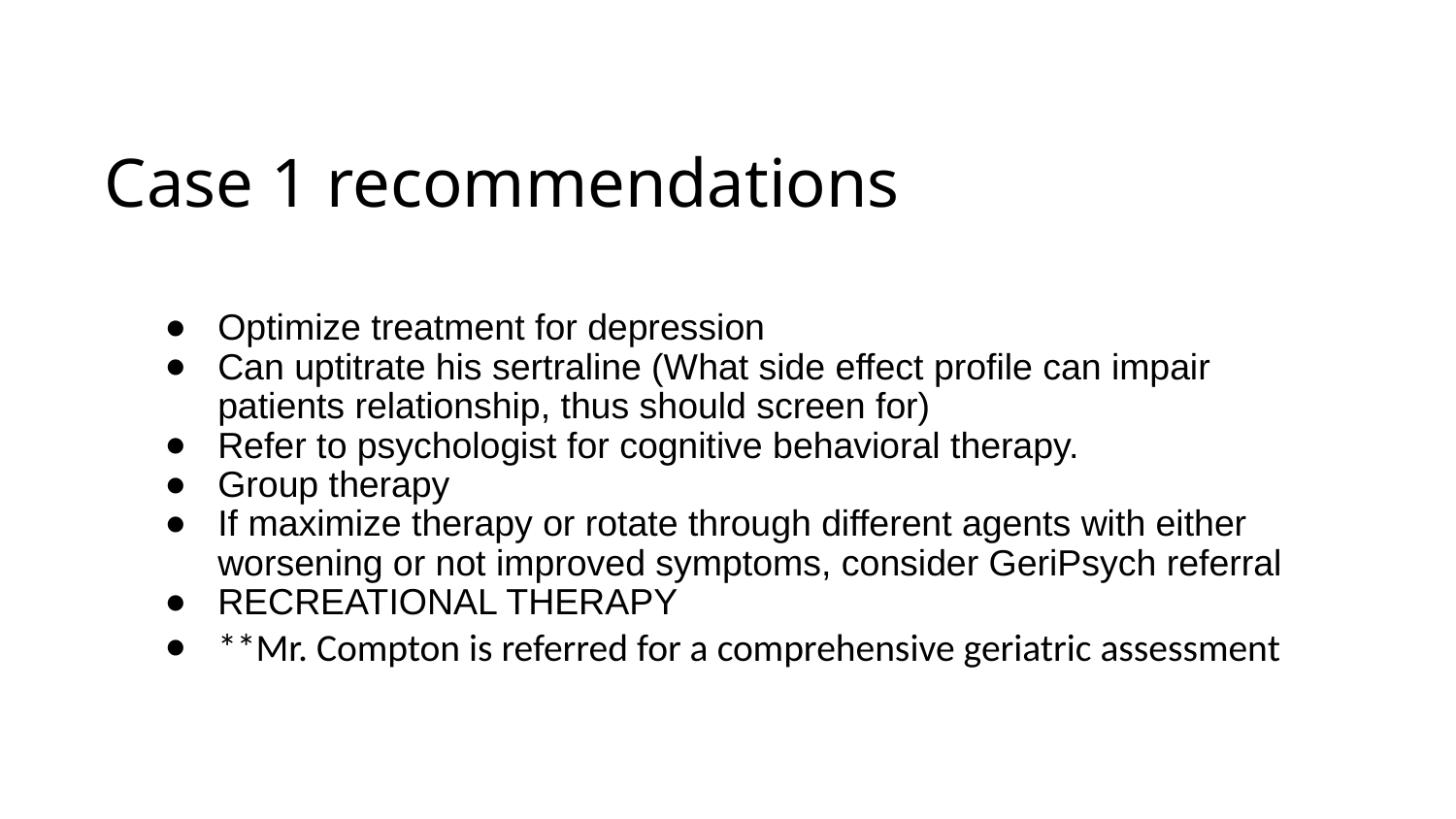

Case 1 recommendations
Optimize treatment for depression
Can uptitrate his sertraline (What side effect profile can impair patients relationship, thus should screen for)
Refer to psychologist for cognitive behavioral therapy.
Group therapy
If maximize therapy or rotate through different agents with either worsening or not improved symptoms, consider GeriPsych referral
RECREATIONAL THERAPY
**Mr. Compton is referred for a comprehensive geriatric assessment

## Slide 18
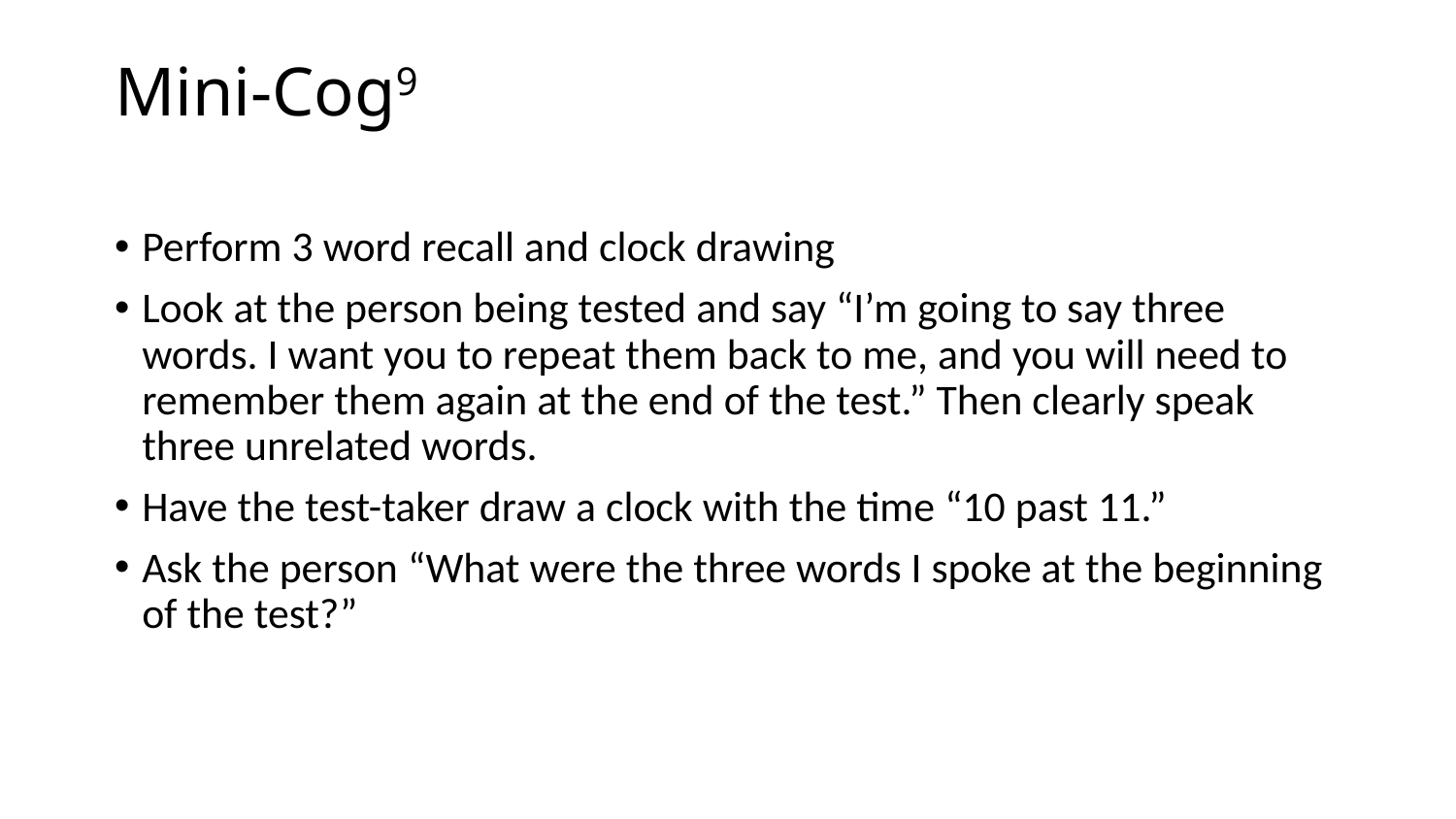

# Mini-Cog9
Perform 3 word recall and clock drawing
Look at the person being tested and say “I’m going to say three words. I want you to repeat them back to me, and you will need to remember them again at the end of the test.” Then clearly speak three unrelated words.
Have the test-taker draw a clock with the time “10 past 11.”
Ask the person “What were the three words I spoke at the beginning of the test?”

## Slide 19
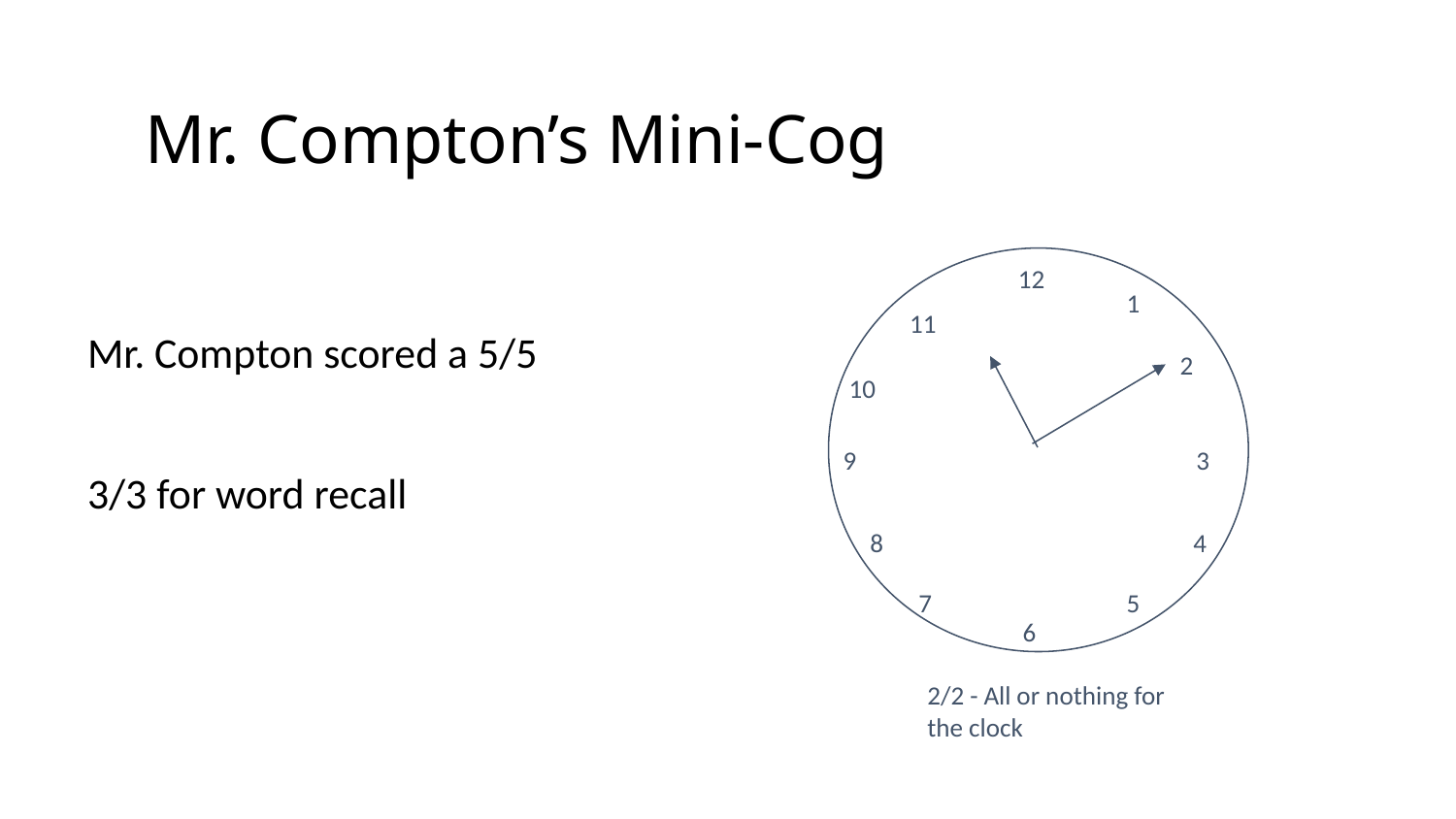

Mr. Compton’s Mini-Cog
12
1
11
Mr. Compton scored a 5/5
3/3 for word recall
2
10
9
3
8
4
7
5
6
2/2 - All or nothing for the clock

## Slide 20
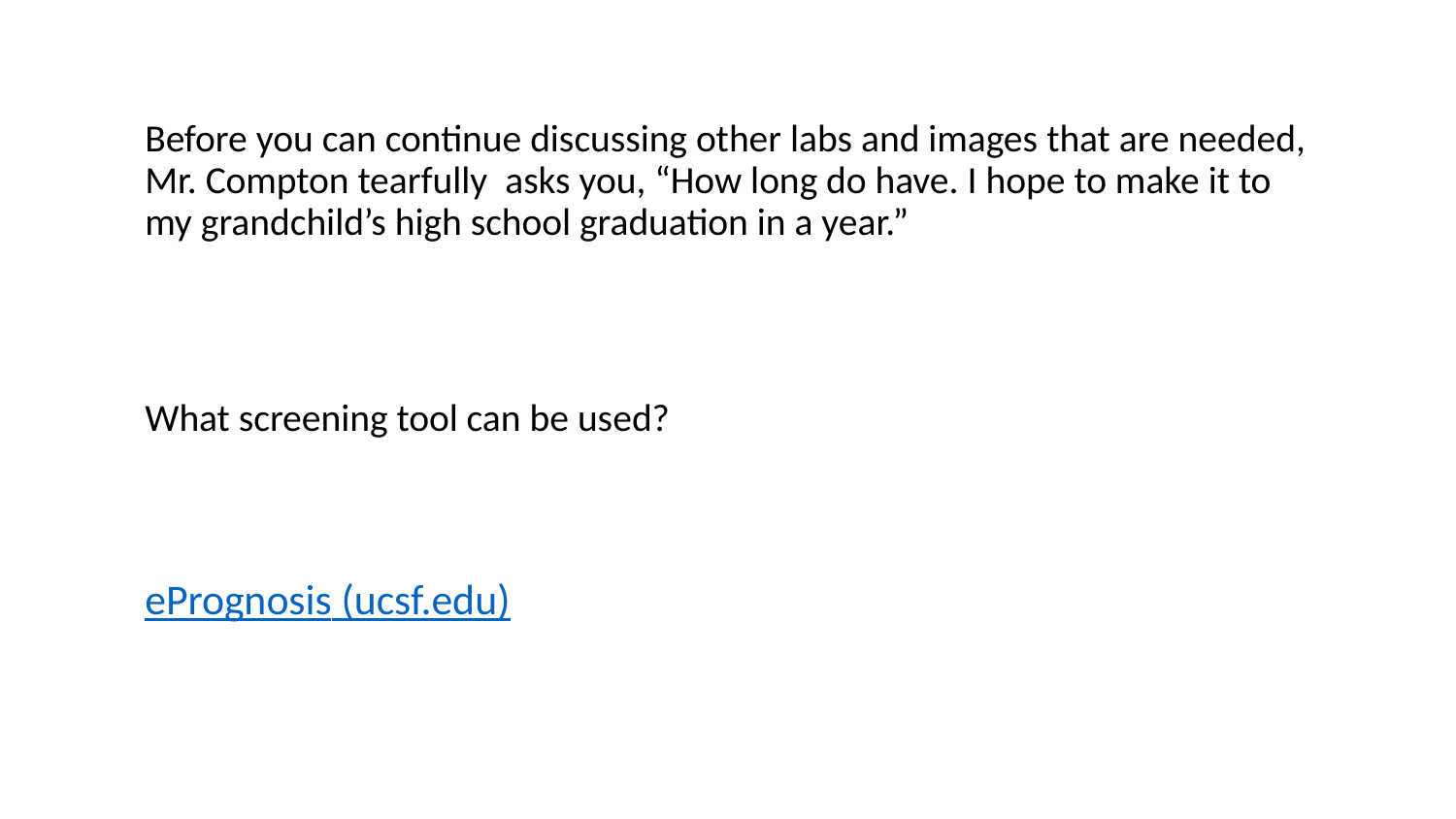

Before you can continue discussing other labs and images that are needed, Mr. Compton tearfully asks you, “How long do have. I hope to make it to my grandchild’s high school graduation in a year.”
What screening tool can be used?
ePrognosis (ucsf.edu)

## Slide 21
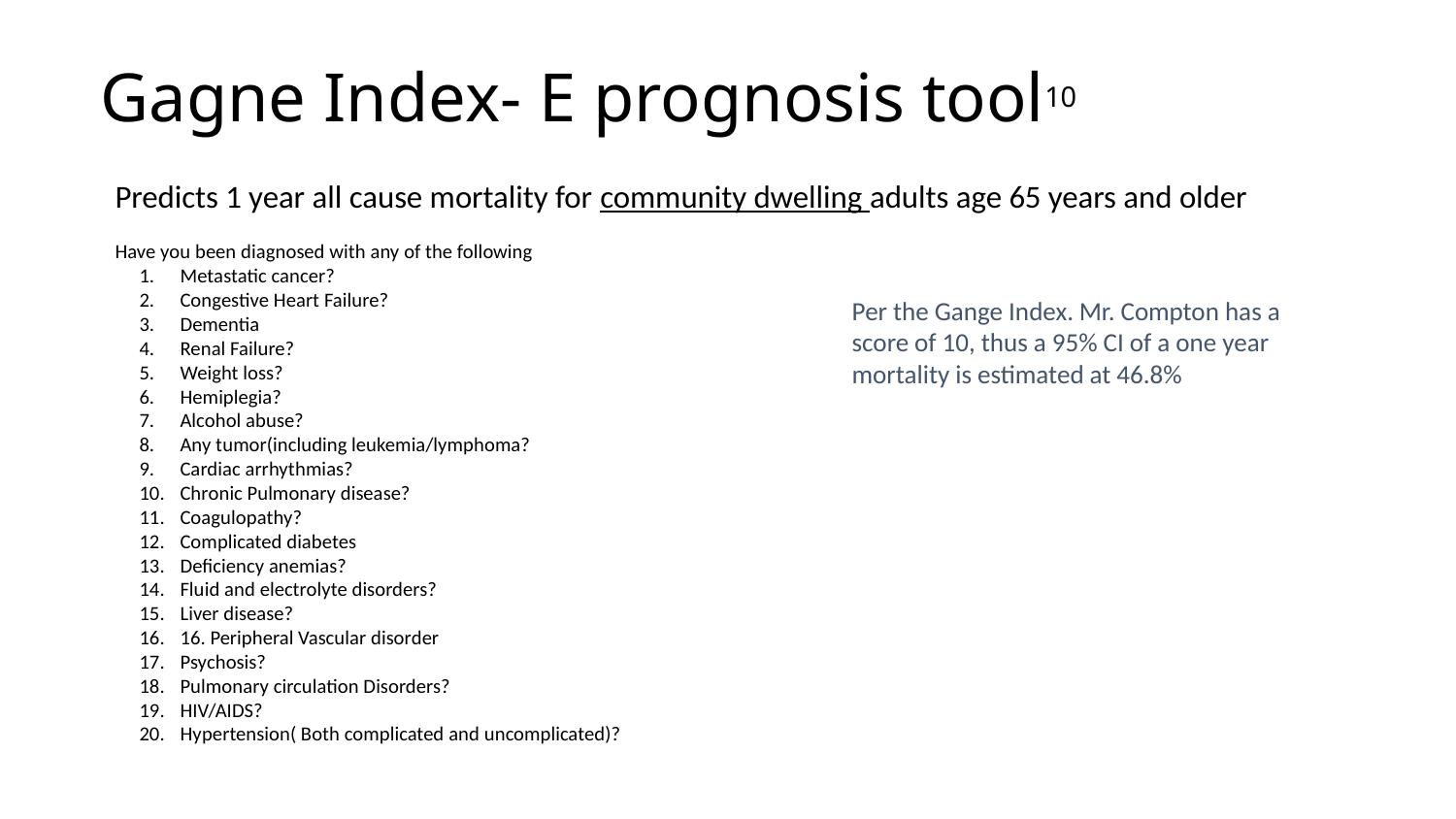

Gagne Index- E prognosis tool10
Predicts 1 year all cause mortality for community dwelling adults age 65 years and older
Have you been diagnosed with any of the following
Metastatic cancer?
Congestive Heart Failure?
Dementia
Renal Failure?
Weight loss?
Hemiplegia?
Alcohol abuse?
Any tumor(including leukemia/lymphoma?
Cardiac arrhythmias?
Chronic Pulmonary disease?
Coagulopathy?
Complicated diabetes
Deficiency anemias?
Fluid and electrolyte disorders?
Liver disease?
16. Peripheral Vascular disorder
Psychosis?
Pulmonary circulation Disorders?
HIV/AIDS?
Hypertension( Both complicated and uncomplicated)?
Per the Gange Index. Mr. Compton has a score of 10, thus a 95% CI of a one year mortality is estimated at 46.8%

## Slide 22
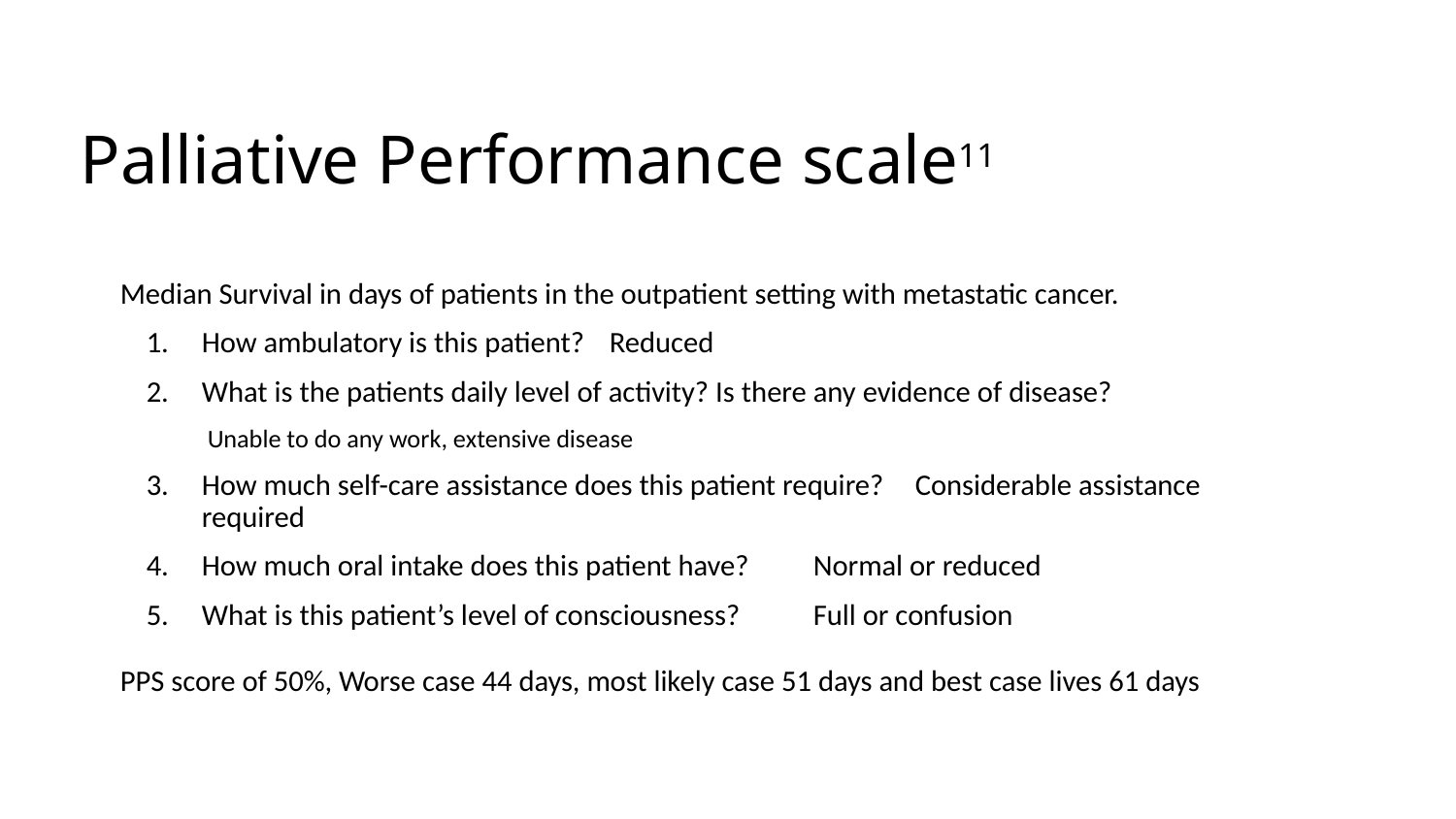

Palliative Performance scale11
Median Survival in days of patients in the outpatient setting with metastatic cancer.
How ambulatory is this patient? 		Reduced
What is the patients daily level of activity? Is there any evidence of disease?
	Unable to do any work, extensive disease
How much self-care assistance does this patient require? 	Considerable assistance required
How much oral intake does this patient have? 	Normal or reduced
What is this patient’s level of consciousness? 	Full or confusion
PPS score of 50%, Worse case 44 days, most likely case 51 days and best case lives 61 days

## Slide 23
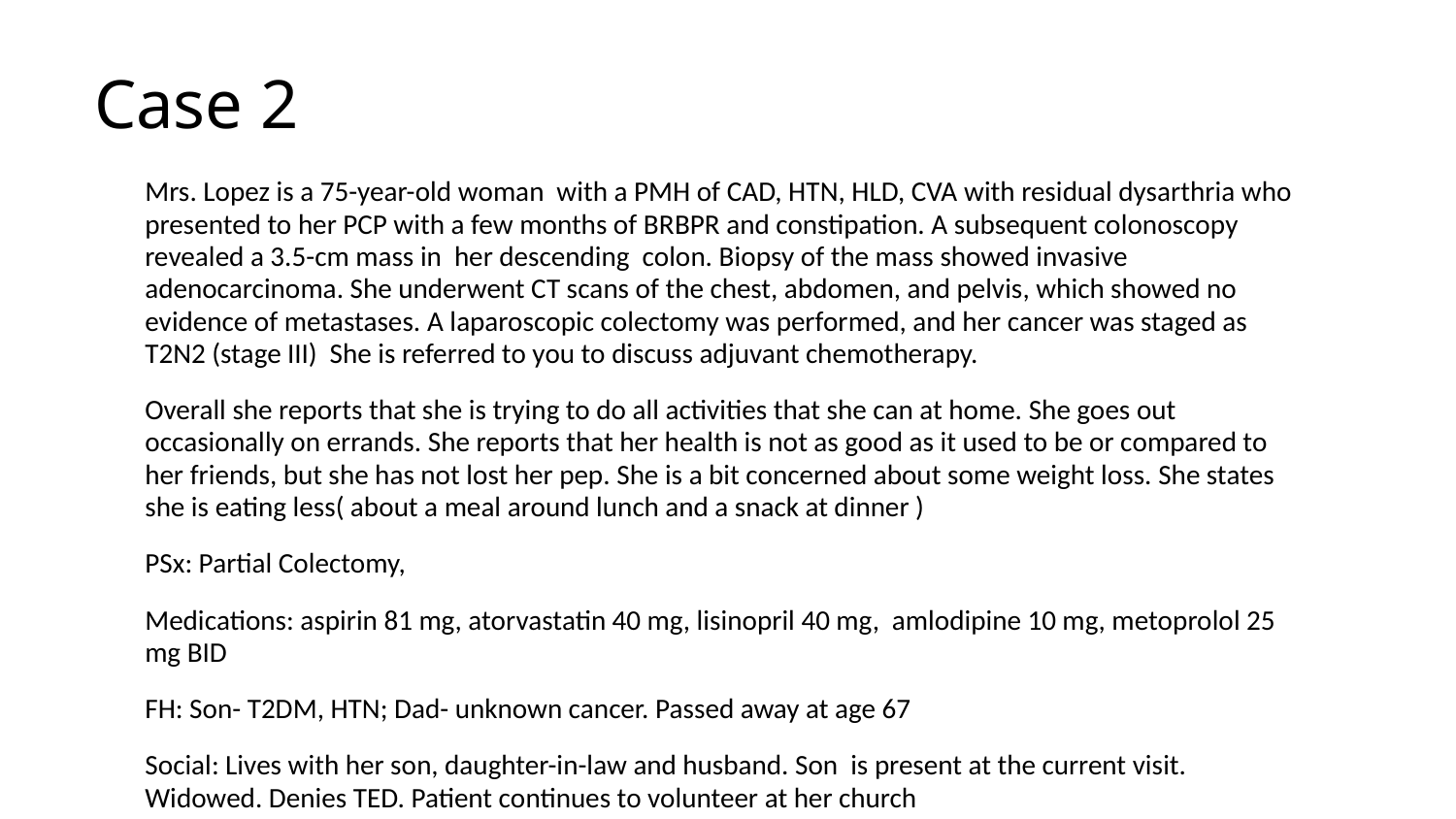

Case 2
Mrs. Lopez is a 75-year-old woman with a PMH of CAD, HTN, HLD, CVA with residual dysarthria who presented to her PCP with a few months of BRBPR and constipation. A subsequent colonoscopy revealed a 3.5-cm mass in her descending colon. Biopsy of the mass showed invasive adenocarcinoma. She underwent CT scans of the chest, abdomen, and pelvis, which showed no evidence of metastases. A laparoscopic colectomy was performed, and her cancer was staged as T2N2 (stage III) She is referred to you to discuss adjuvant chemotherapy.
Overall she reports that she is trying to do all activities that she can at home. She goes out occasionally on errands. She reports that her health is not as good as it used to be or compared to her friends, but she has not lost her pep. She is a bit concerned about some weight loss. She states she is eating less( about a meal around lunch and a snack at dinner )
PSx: Partial Colectomy,
Medications: aspirin 81 mg, atorvastatin 40 mg, lisinopril 40 mg, amlodipine 10 mg, metoprolol 25 mg BID
FH: Son- T2DM, HTN; Dad- unknown cancer. Passed away at age 67
Social: Lives with her son, daughter-in-law and husband. Son is present at the current visit. Widowed. Denies TED. Patient continues to volunteer at her church

## Slide 24
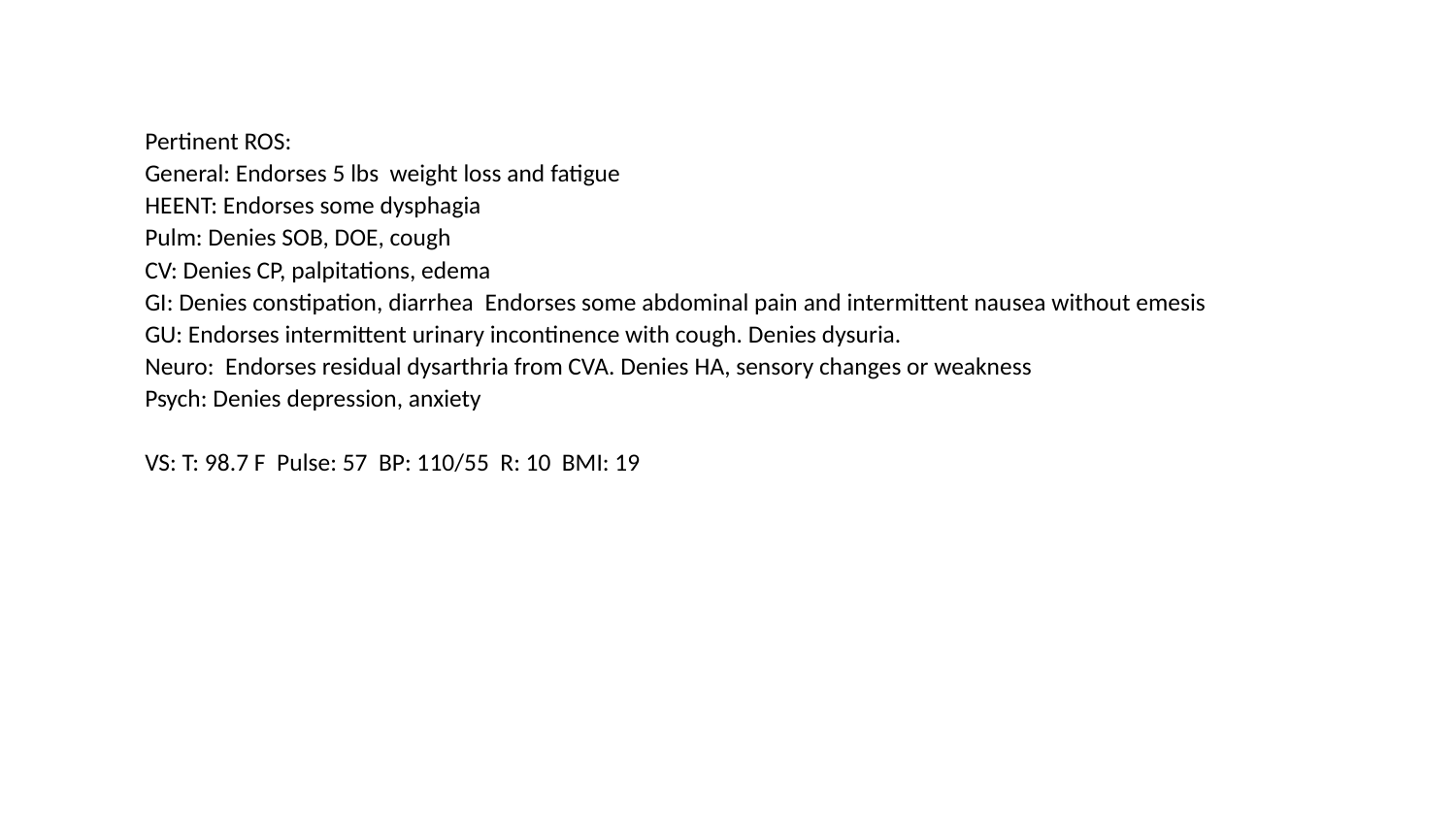

Pertinent ROS:
General: Endorses 5 lbs weight loss and fatigue
HEENT: Endorses some dysphagia
Pulm: Denies SOB, DOE, cough
CV: Denies CP, palpitations, edema
GI: Denies constipation, diarrhea Endorses some abdominal pain and intermittent nausea without emesis
GU: Endorses intermittent urinary incontinence with cough. Denies dysuria.
Neuro: Endorses residual dysarthria from CVA. Denies HA, sensory changes or weakness
Psych: Denies depression, anxiety
VS: T: 98.7 F Pulse: 57 BP: 110/55 R: 10 BMI: 19

## Slide 25
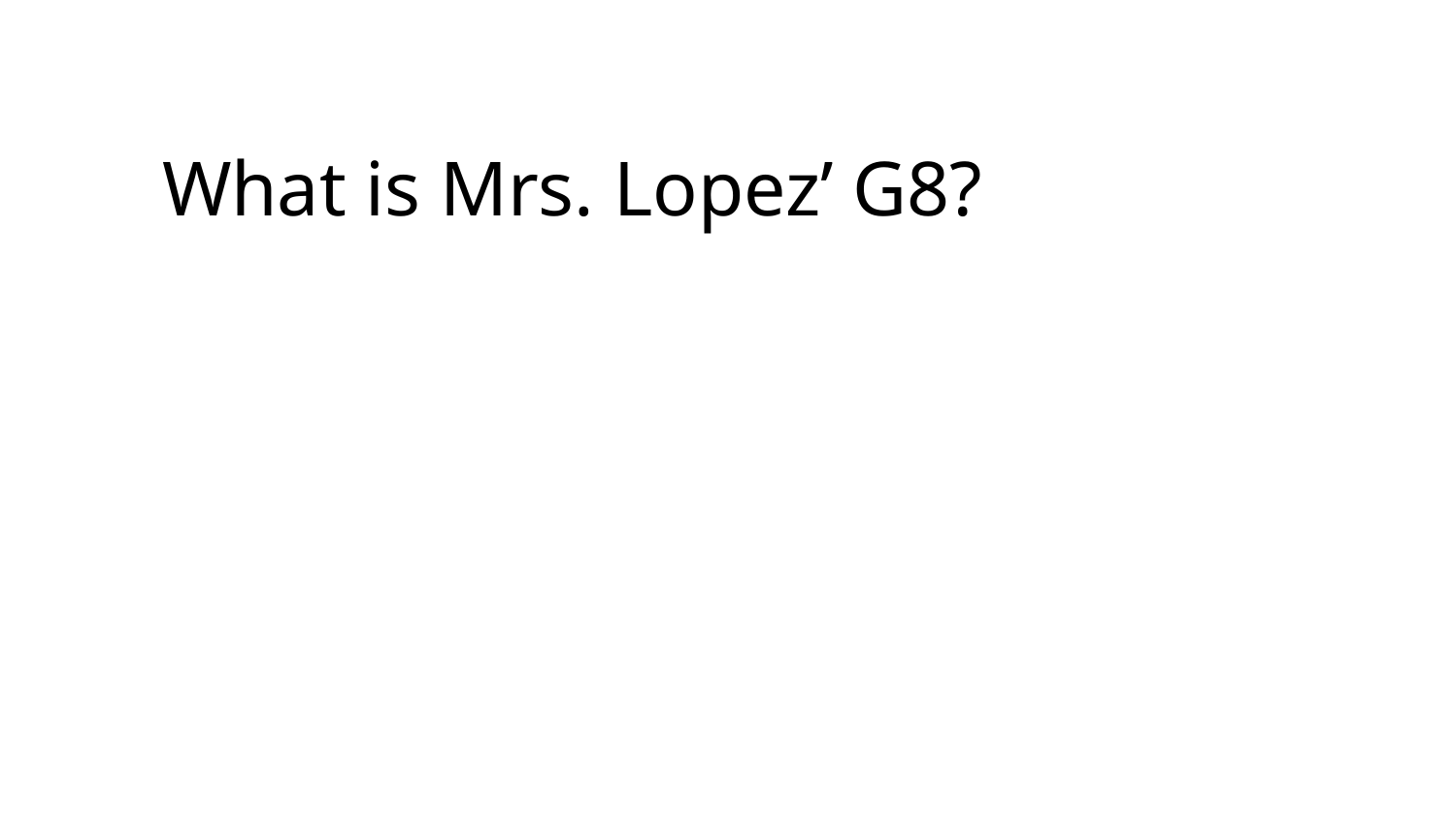

What is Mrs. Lopez’ G8?

## Slide 26
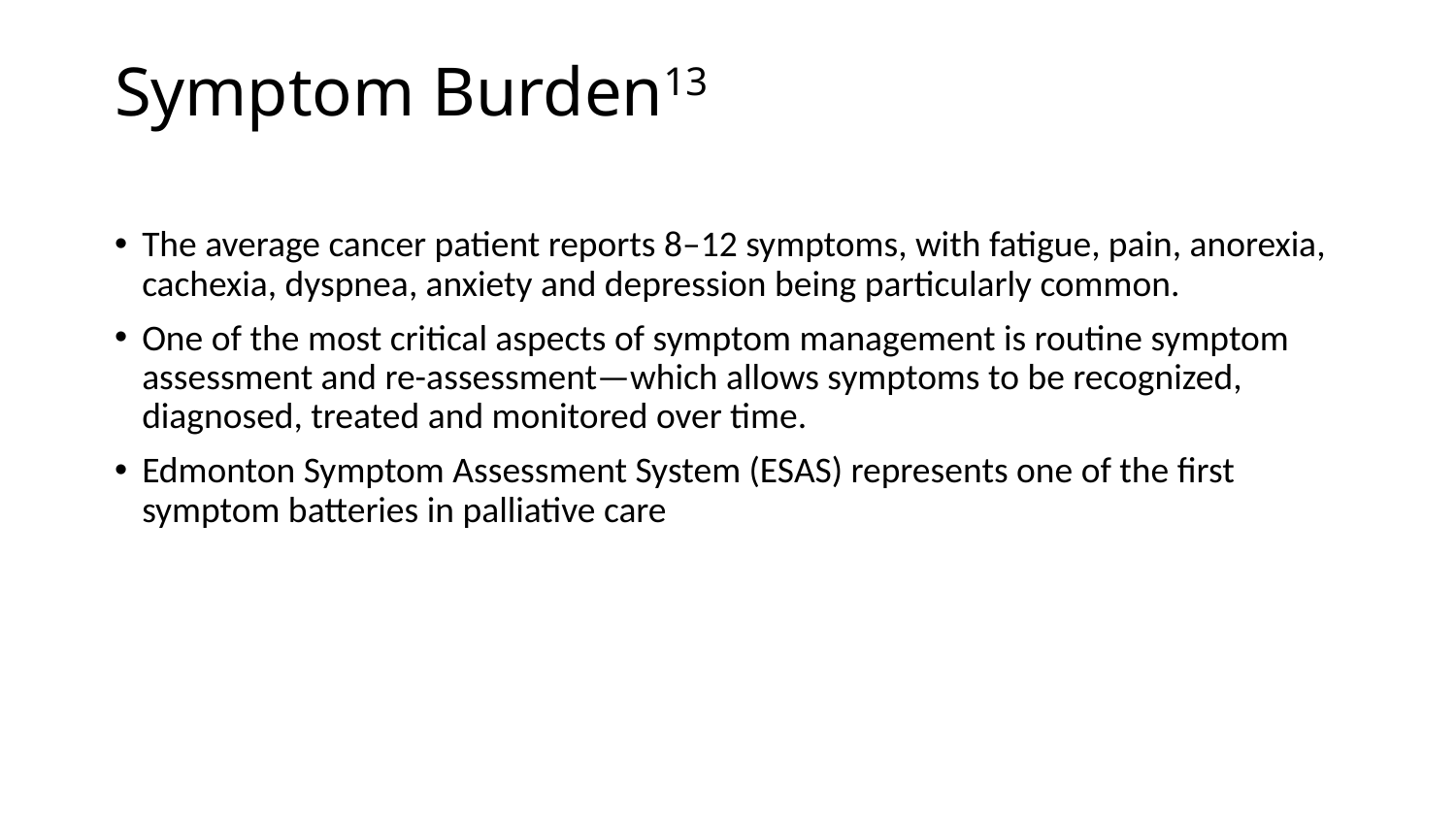

# Symptom Burden13
The average cancer patient reports 8–12 symptoms, with fatigue, pain, anorexia, cachexia, dyspnea, anxiety and depression being particularly common.
One of the most critical aspects of symptom management is routine symptom assessment and re-assessment—which allows symptoms to be recognized, diagnosed, treated and monitored over time.
Edmonton Symptom Assessment System (ESAS) represents one of the first symptom batteries in palliative care

## Slide 27
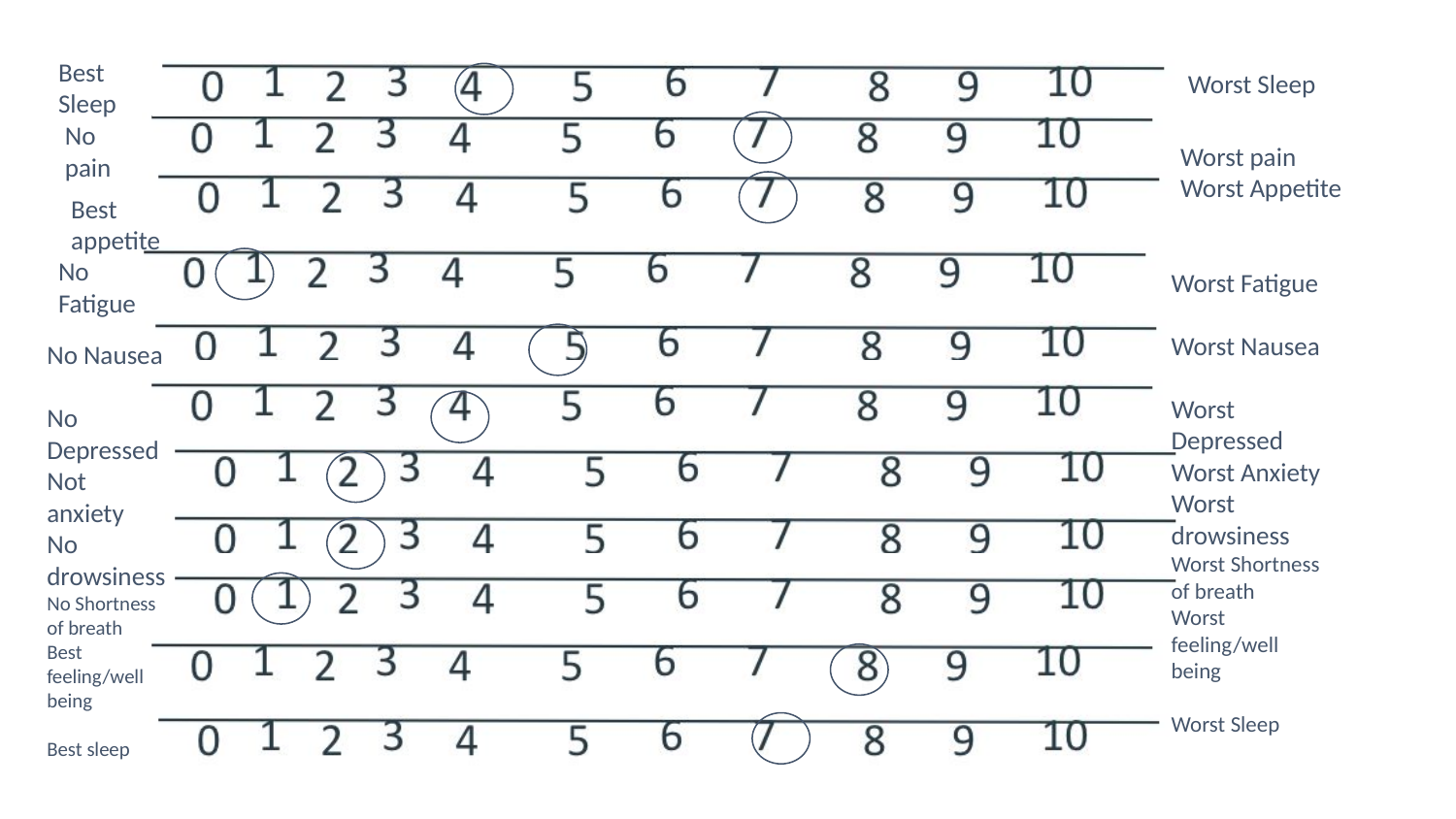

Best Sleep
Worst Sleep
No pain
Worst pain
Worst Appetite
Best appetite
No Fatigue
Worst Fatigue
Worst Nausea
Worst Depressed
Worst Anxiety
Worst drowsiness
Worst Shortness of breath
Worst feeling/well being
Worst Sleep
No Nausea
No Depressed
Not anxiety
No drowsiness
No Shortness of breath
Best feeling/well being
Best sleep

## Slide 28
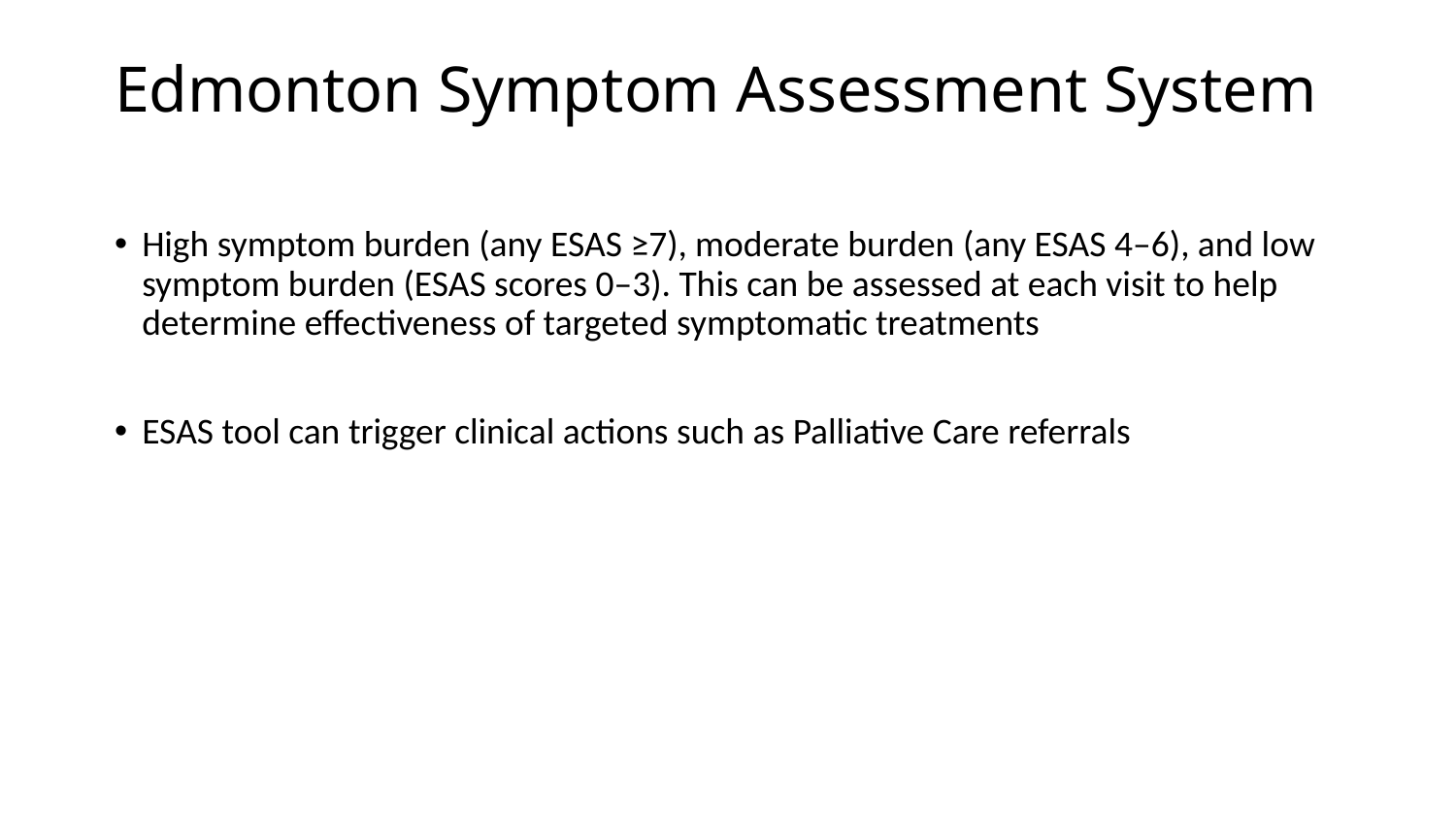

# Edmonton Symptom Assessment System
High symptom burden (any ESAS ≥7), moderate burden (any ESAS 4–6), and low symptom burden (ESAS scores 0–3). This can be assessed at each visit to help determine effectiveness of targeted symptomatic treatments
ESAS tool can trigger clinical actions such as Palliative Care referrals

## Slide 29
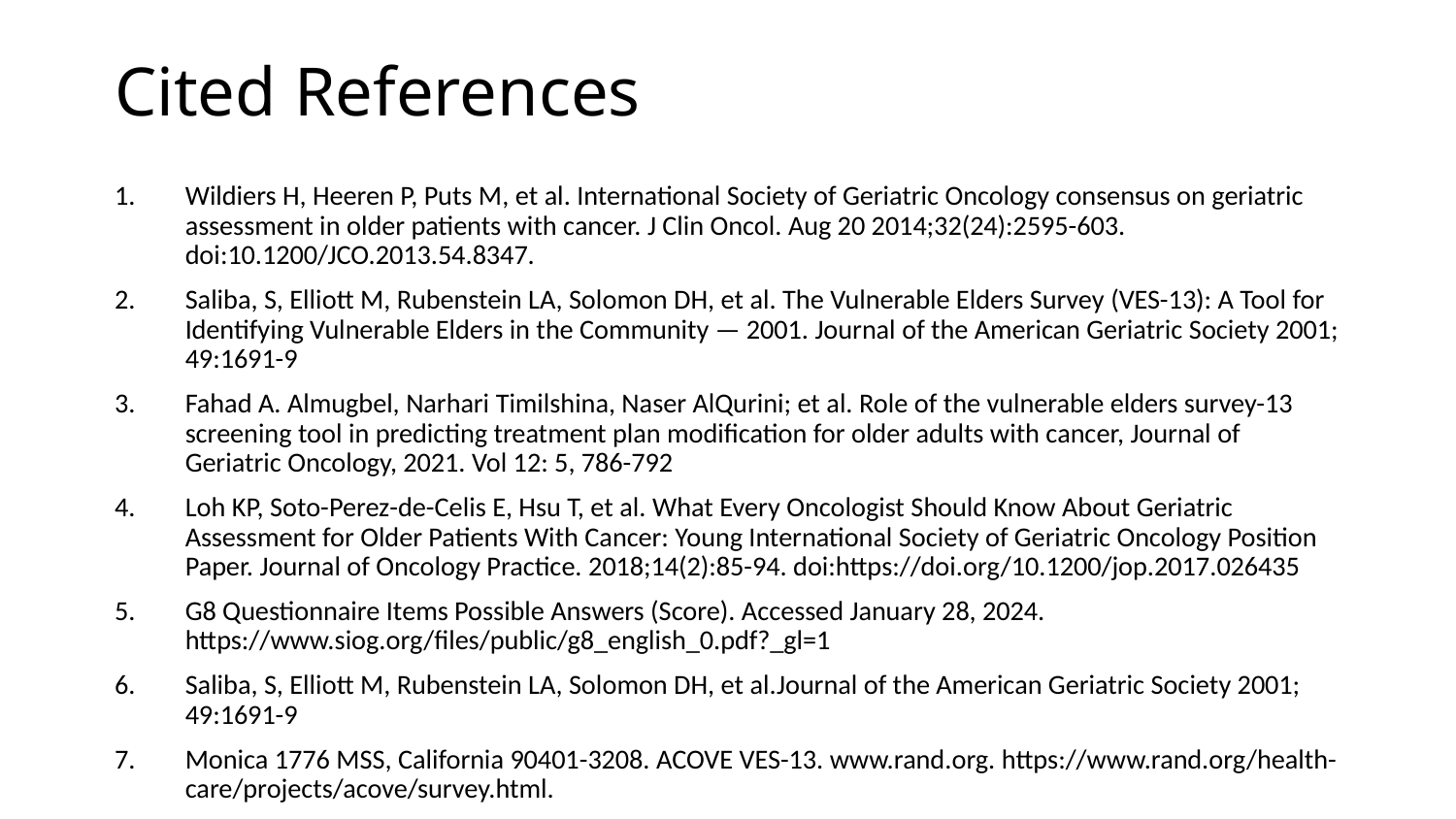

# Cited References
Wildiers H, Heeren P, Puts M, et al. International Society of Geriatric Oncology consensus on geriatric assessment in older patients with cancer. J Clin Oncol. Aug 20 2014;32(24):2595-603. doi:10.1200/JCO.2013.54.8347.
Saliba, S, Elliott M, Rubenstein LA, Solomon DH, et al. The Vulnerable Elders Survey (VES-13): A Tool for Identifying Vulnerable Elders in the Community — 2001. Journal of the American Geriatric Society 2001; 49:1691-9
Fahad A. Almugbel, Narhari Timilshina, Naser AlQurini; et al. Role of the vulnerable elders survey-13 screening tool in predicting treatment plan modification for older adults with cancer, Journal of Geriatric Oncology, 2021. Vol 12: 5, 786-792
Loh KP, Soto-Perez-de-Celis E, Hsu T, et al. What Every Oncologist Should Know About Geriatric Assessment for Older Patients With Cancer: Young International Society of Geriatric Oncology Position Paper. Journal of Oncology Practice. 2018;14(2):85-94. doi:https://doi.org/10.1200/jop.2017.026435
G8 Questionnaire Items Possible Answers (Score). Accessed January 28, 2024. https://www.siog.org/files/public/g8_english_0.pdf?_gl=1
Saliba, S, Elliott M, Rubenstein LA, Solomon DH, et al.Journal of the American Geriatric Society 2001; 49:1691-9
Monica 1776 MSS, California 90401-3208. ACOVE VES-13. www.rand.org. https://www.rand.org/health-care/projects/acove/survey.html.

## Slide 30
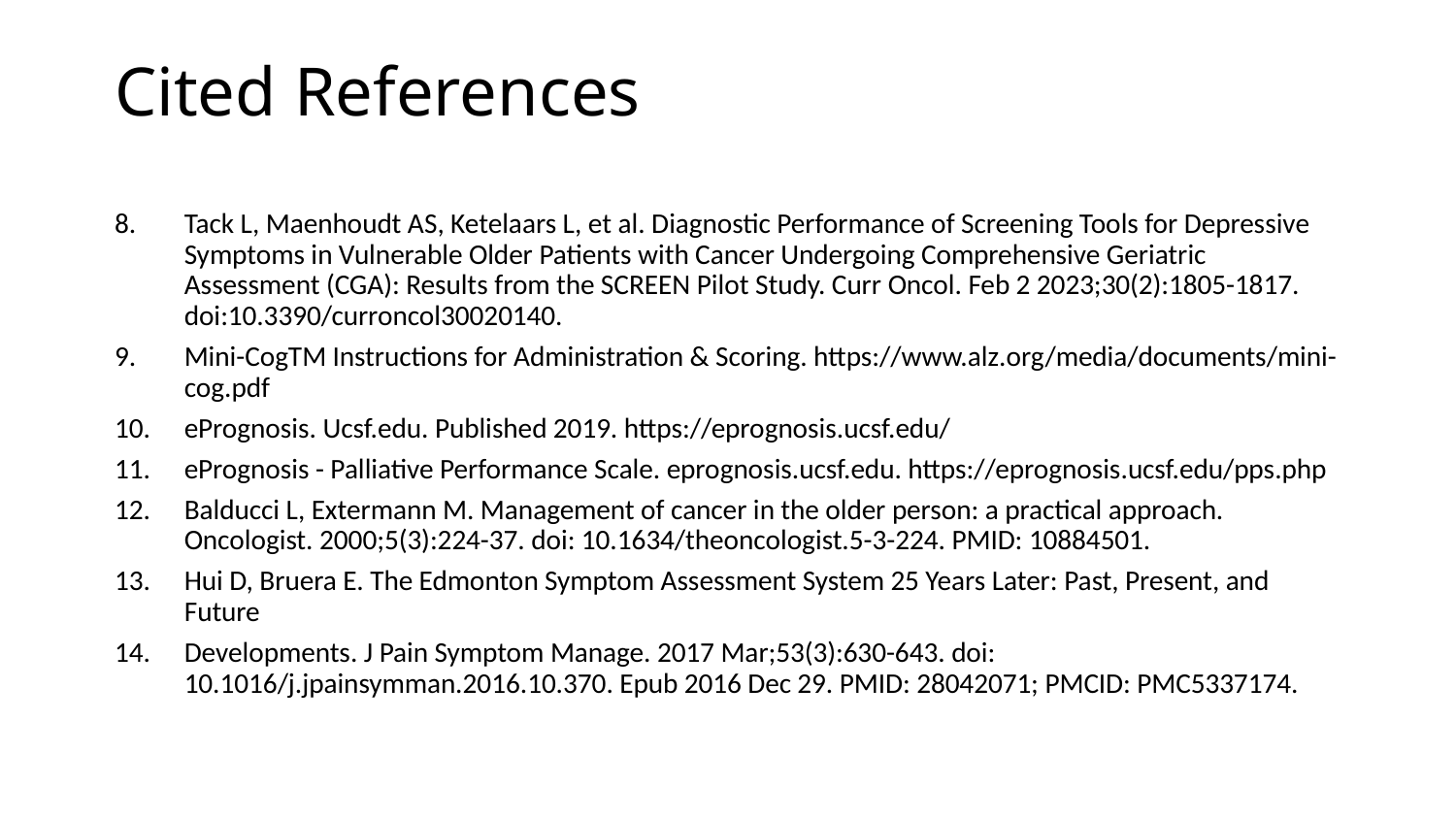

# Cited References
Tack L, Maenhoudt AS, Ketelaars L, et al. Diagnostic Performance of Screening Tools for Depressive Symptoms in Vulnerable Older Patients with Cancer Undergoing Comprehensive Geriatric Assessment (CGA): Results from the SCREEN Pilot Study. Curr Oncol. Feb 2 2023;30(2):1805-1817. doi:10.3390/curroncol30020140.
Mini-CogTM Instructions for Administration & Scoring. https://www.alz.org/media/documents/mini-cog.pdf
ePrognosis. Ucsf.edu. Published 2019. https://eprognosis.ucsf.edu/
‌ePrognosis - Palliative Performance Scale. eprognosis.ucsf.edu. https://eprognosis.ucsf.edu/pps.php
Balducci L, Extermann M. Management of cancer in the older person: a practical approach. Oncologist. 2000;5(3):224-37. doi: 10.1634/theoncologist.5-3-224. PMID: 10884501.
Hui D, Bruera E. The Edmonton Symptom Assessment System 25 Years Later: Past, Present, and Future
Developments. J Pain Symptom Manage. 2017 Mar;53(3):630-643. doi: 10.1016/j.jpainsymman.2016.10.370. Epub 2016 Dec 29. PMID: 28042071; PMCID: PMC5337174.
