# Supplementary material for: An Introductory Course on Geriatric Oncology
Source: MedEdPORTAL. 2024 Nov 14;20:11471. doi: 10.15766/mep_2374-8265.11471 (PMC11561070; doi:10.15766/mep_2374-8265.11471)
Supplement: Supplementary file 1 — Introduction to Geriatric Oncology.pptxThe Comprehensive Geriatric Assessment.pptxGeriatric Screening Tools.pptxBiology of Aging.pptxCancer Therapy in the Older Adult.pptxSummary of Interactive Sessions.docxSession 5 Patient Case 1.docxSession 5 Patient Case 2.docxSession 5 Patient Case 3.docxGeriatric Oncology Knowledge Assessment.docxKnowledge Assessment Answer Key.docxSelf-Perceived Competency Assessment.docxCurriculum Session Assessment.docx [file mep_2374-8265.11471-s001.zip › D. Biology of Aging.pptx]

## Slide 1
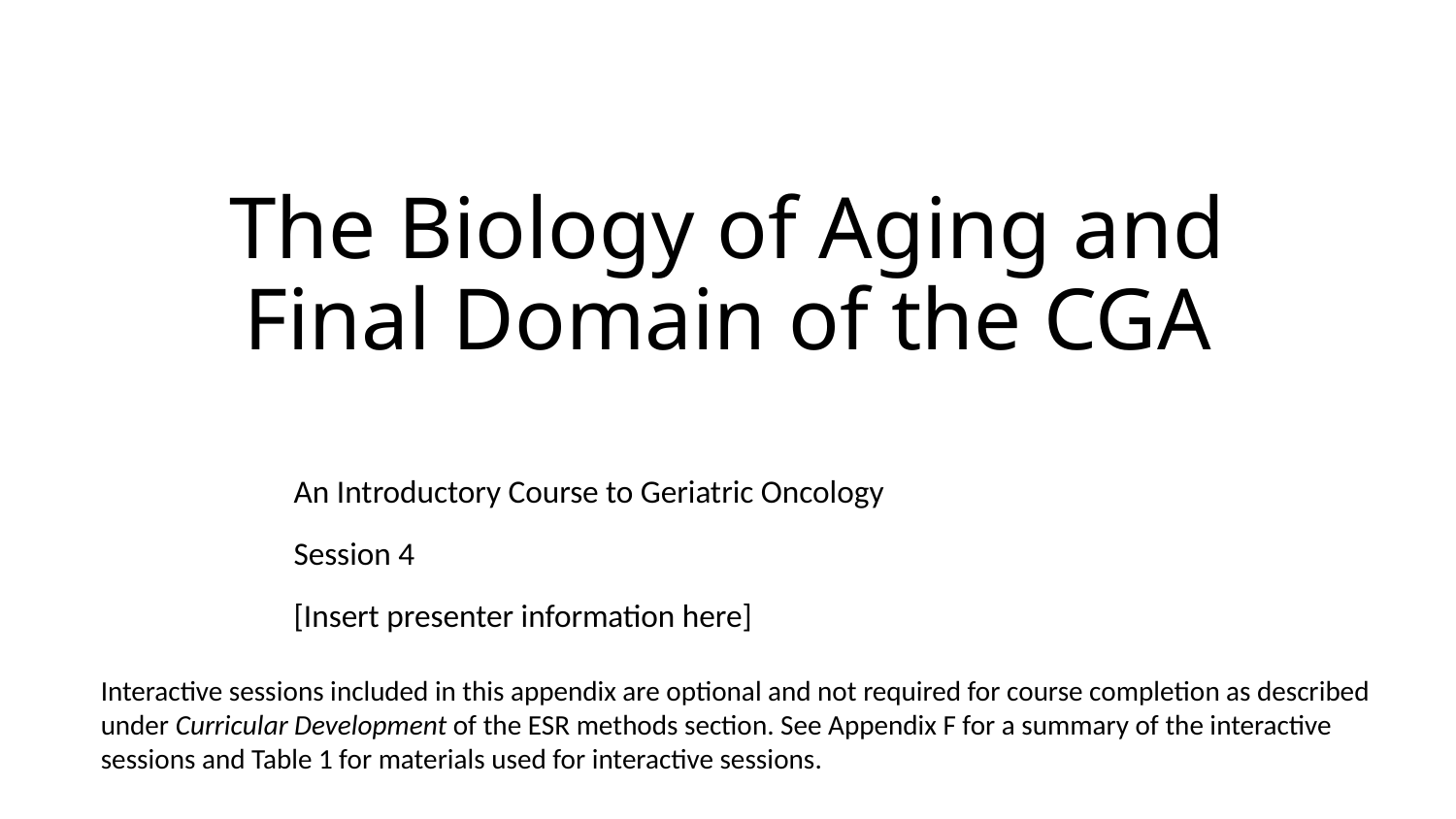

# The Biology of Aging and Final Domain of the CGA
An Introductory Course to Geriatric Oncology
Session 4
[Insert presenter information here]
Interactive sessions included in this appendix are optional and not required for course completion as described under Curricular Development of the ESR methods section. See Appendix F for a summary of the interactive sessions and Table 1 for materials used for interactive sessions.

## Slide 2
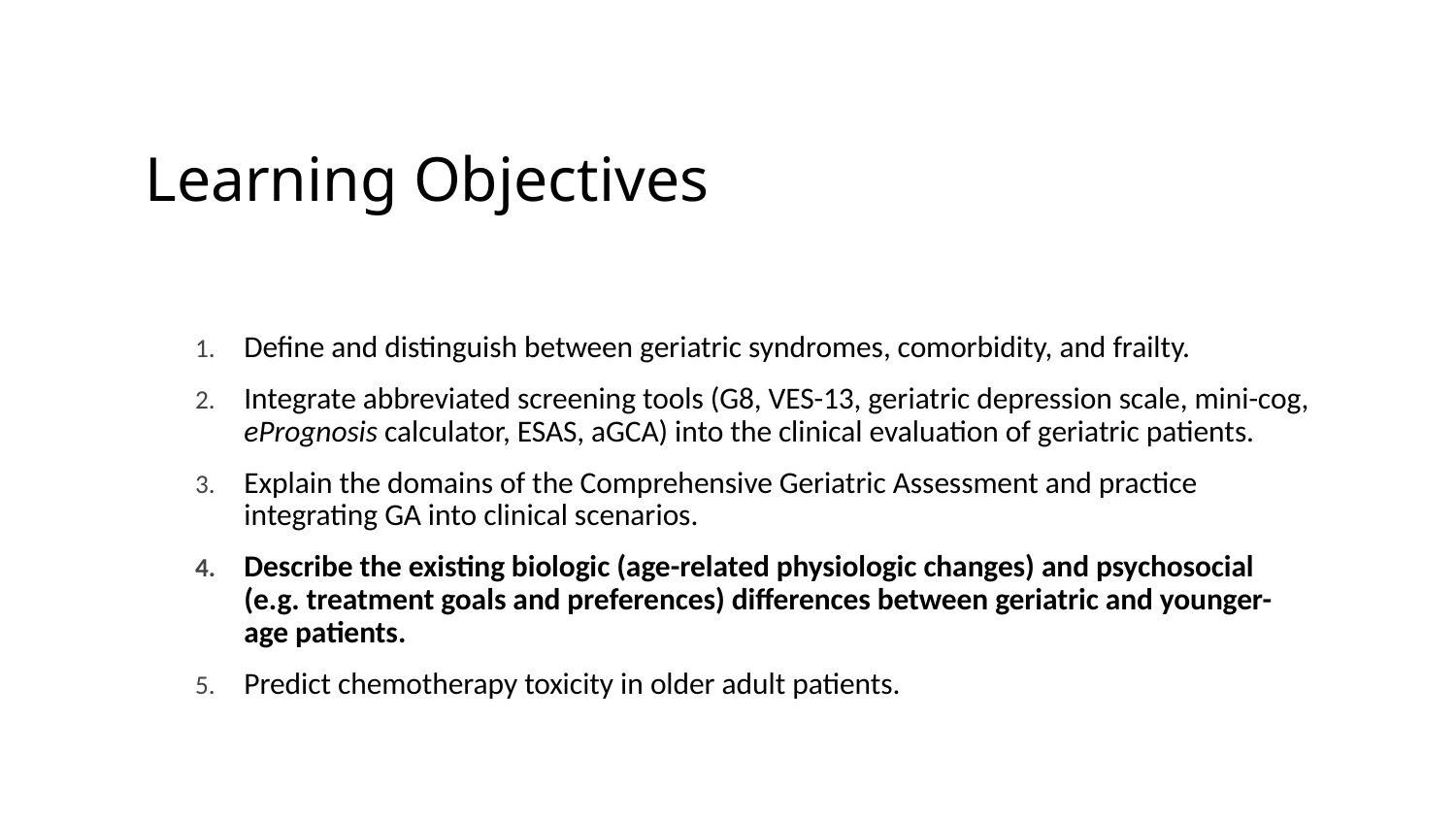

# Learning Objectives
Define and distinguish between geriatric syndromes, comorbidity, and frailty. ​
Integrate abbreviated screening tools (G8, VES-13, geriatric depression scale, mini-cog, ePrognosis calculator, ESAS, aGCA) into the clinical evaluation of geriatric patients. ​
Explain the domains of the Comprehensive Geriatric Assessment and practice integrating GA into clinical scenarios. ​
Describe the existing biologic (age-related physiologic changes) and psychosocial (e.g. treatment goals and preferences) differences between geriatric and younger-age patients. ​​
Predict chemotherapy toxicity in older adult patients.​

## Slide 3
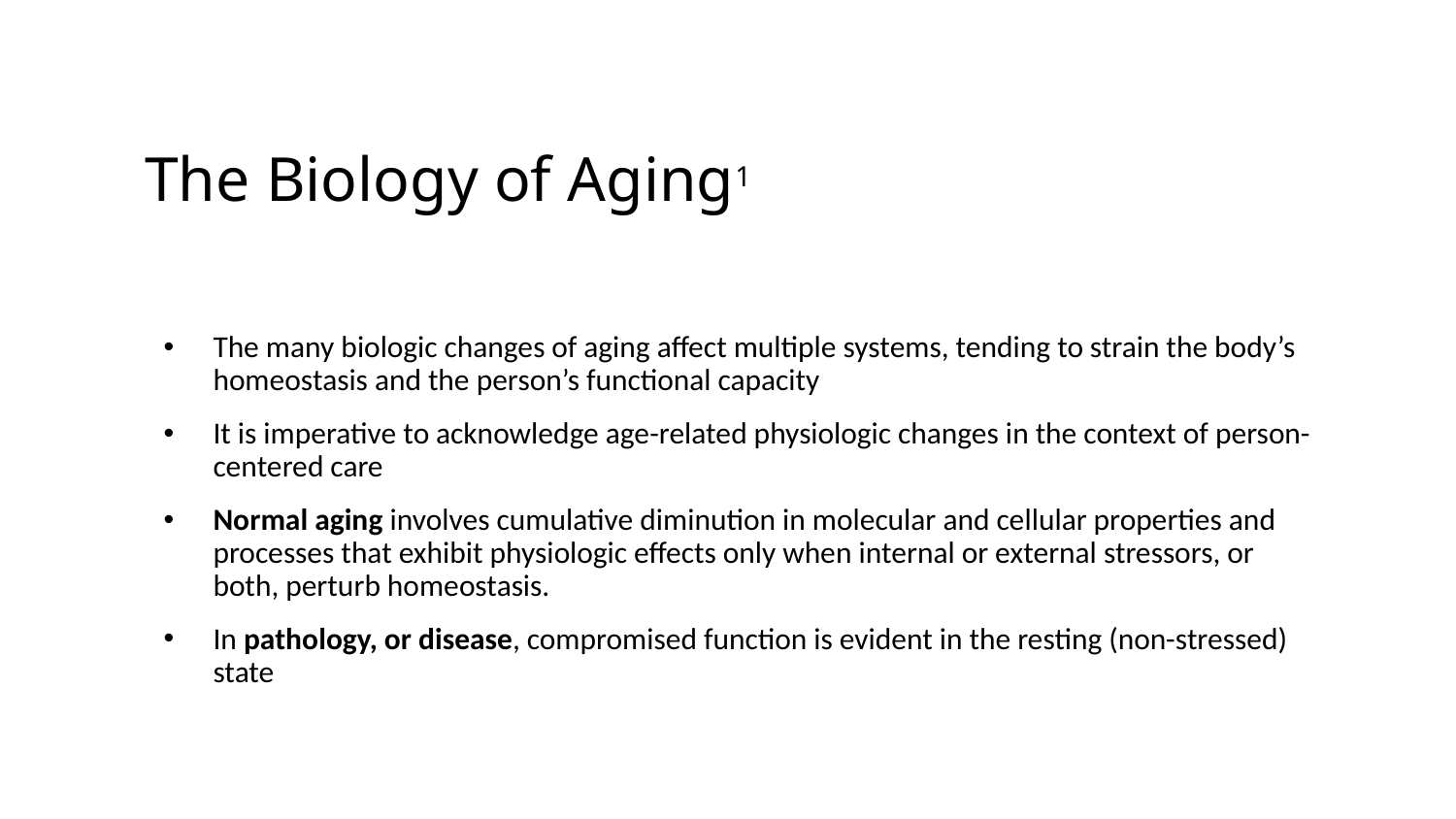

# The Biology of Aging1
The many biologic changes of aging affect multiple systems, tending to strain the body’s homeostasis and the person’s functional capacity​
It is imperative to acknowledge age-related physiologic changes in the context of person-centered care
Normal aging involves cumulative diminution in molecular and cellular properties and processes that exhibit physiologic effects only when internal or external stressors, or both, perturb homeostasis.​​
In pathology, or disease, compromised function is evident in the resting (non-stressed) state

## Slide 4
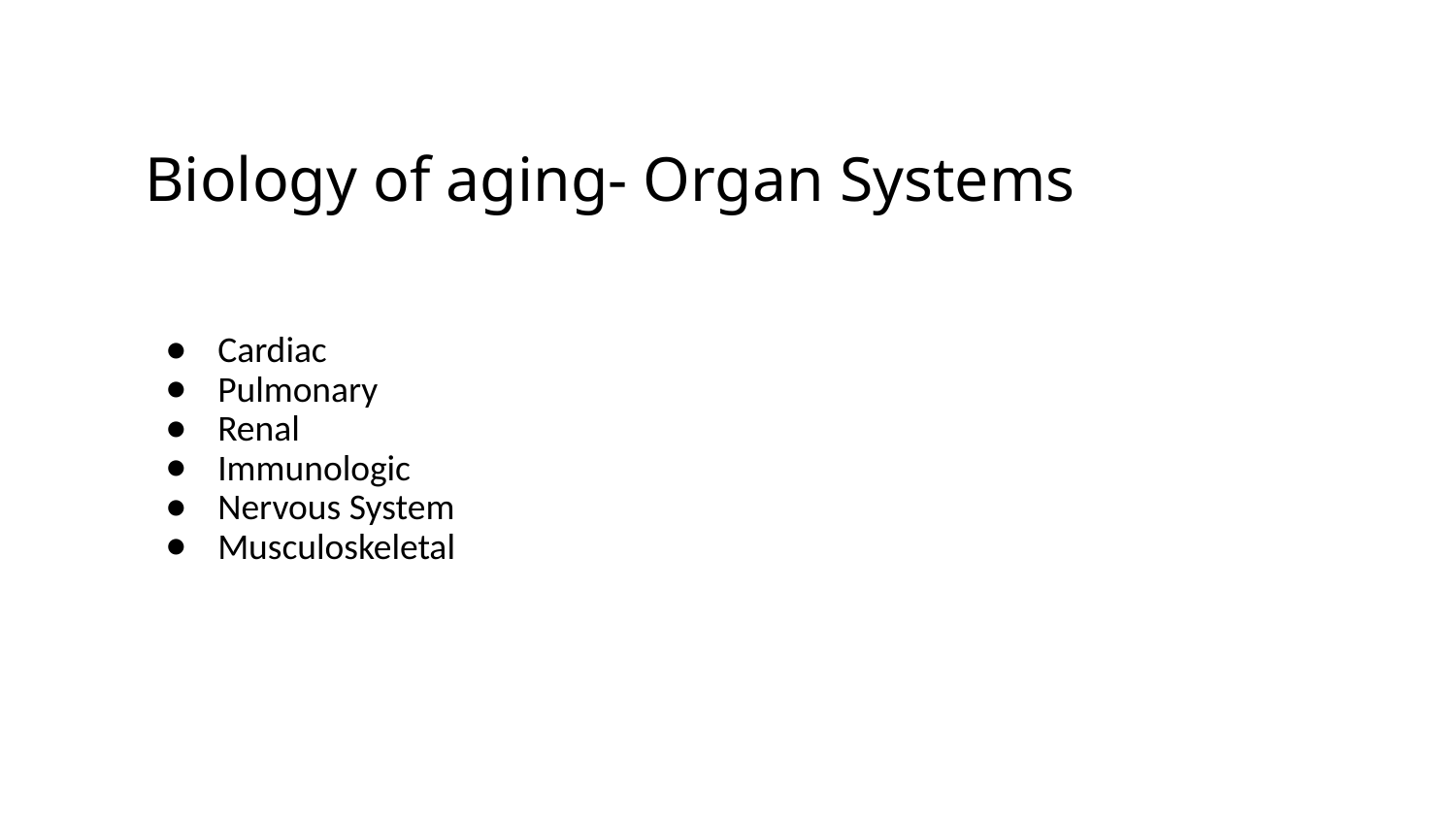

# Biology of aging- Organ Systems
Cardiac
Pulmonary
Renal
Immunologic
Nervous System
Musculoskeletal

## Slide 5
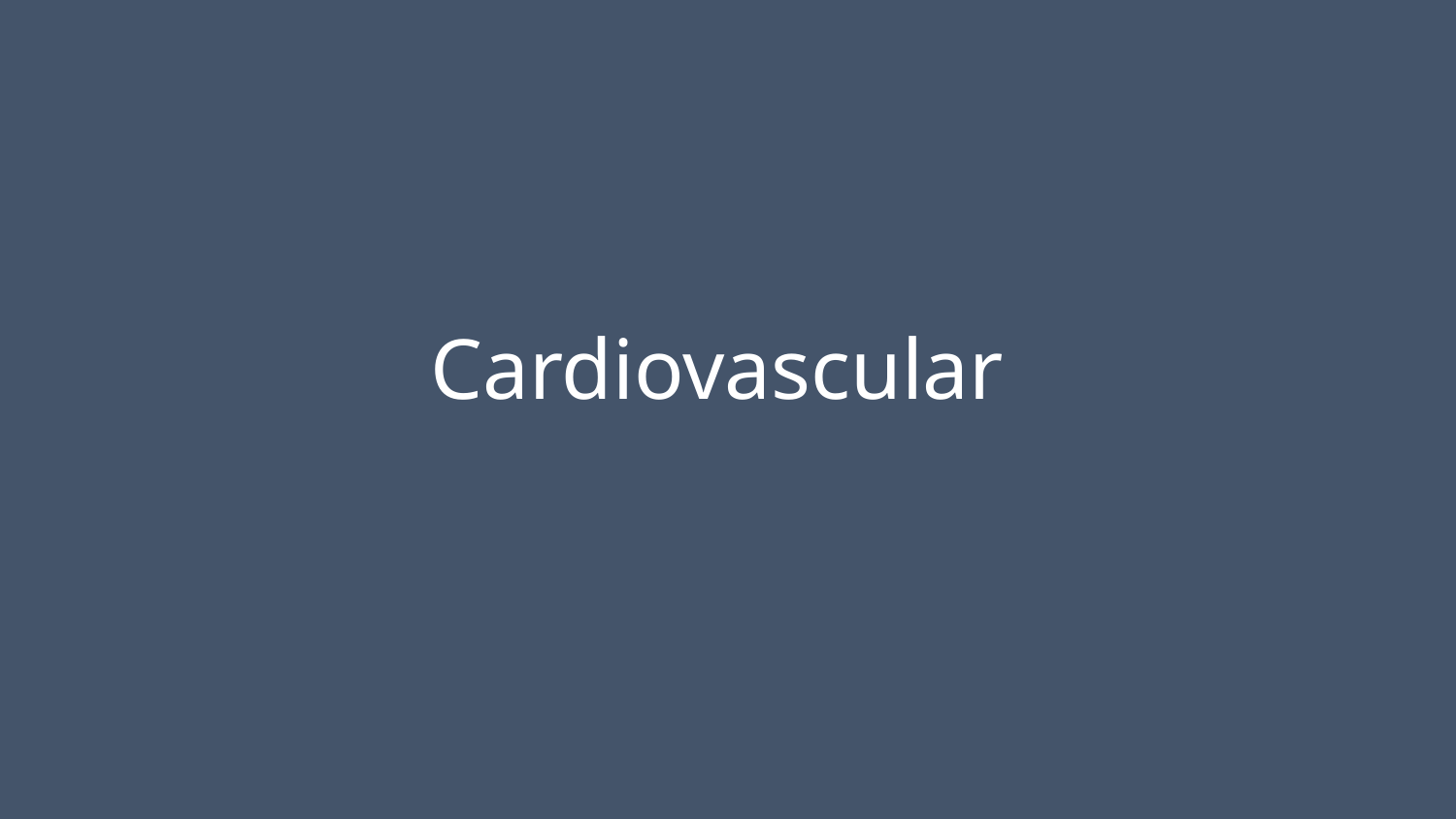

# Cardiovascular

## Slide 6
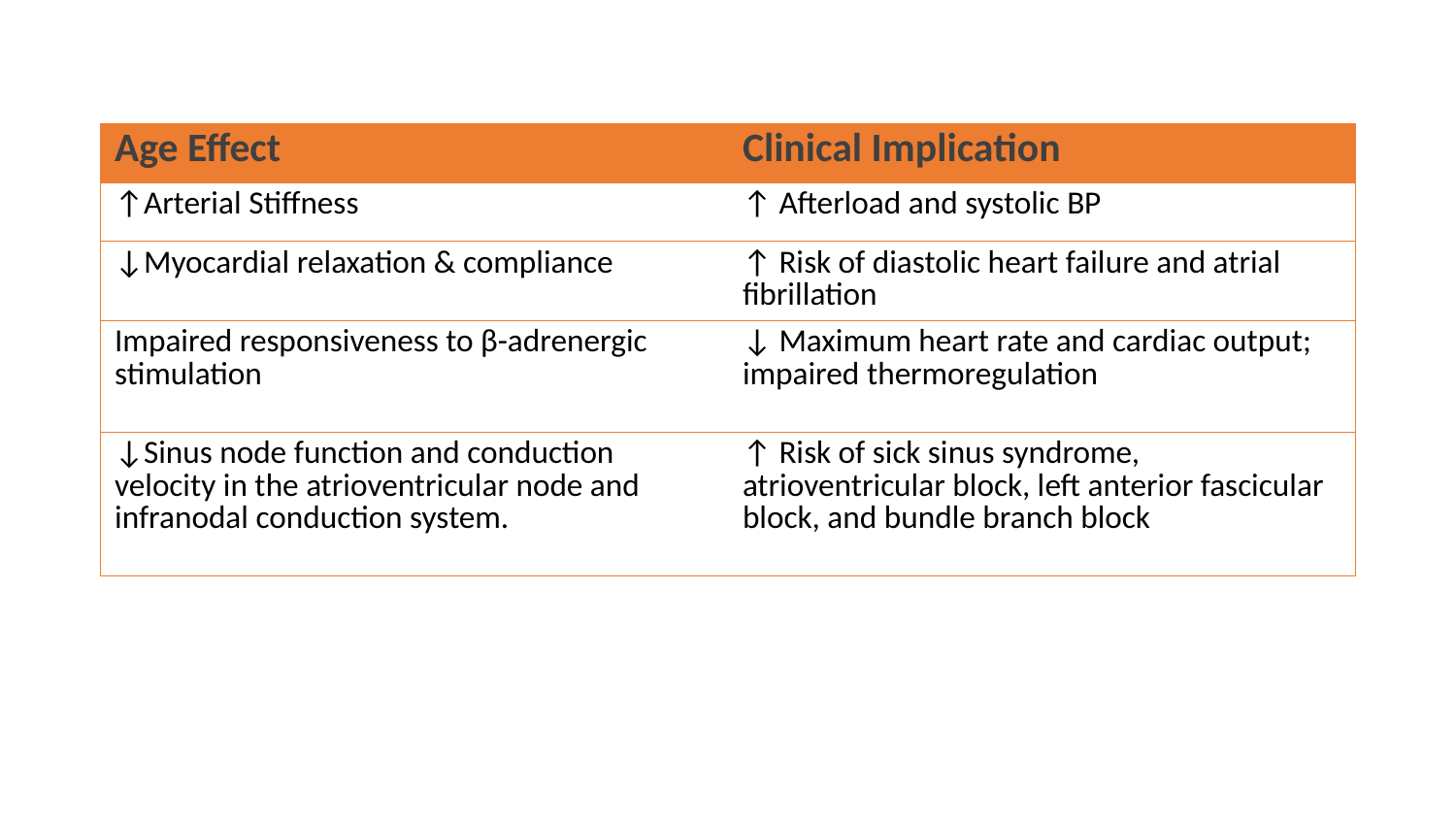

| Age Effect | Clinical Implication |
| --- | --- |
| ↑Arterial Stiffness | ↑ Afterload and systolic BP |
| ↓Myocardial relaxation & compliance | ↑ Risk of diastolic heart failure and atrial fibrillation |
| Impaired responsiveness to β-adrenergic stimulation | ↓ Maximum heart rate and cardiac output; impaired thermoregulation |
| ↓Sinus node function and conduction velocity in the atrioventricular node and infranodal conduction system. | ↑ Risk of sick sinus syndrome, atrioventricular block, left anterior fascicular block, and bundle branch block |

## Slide 7
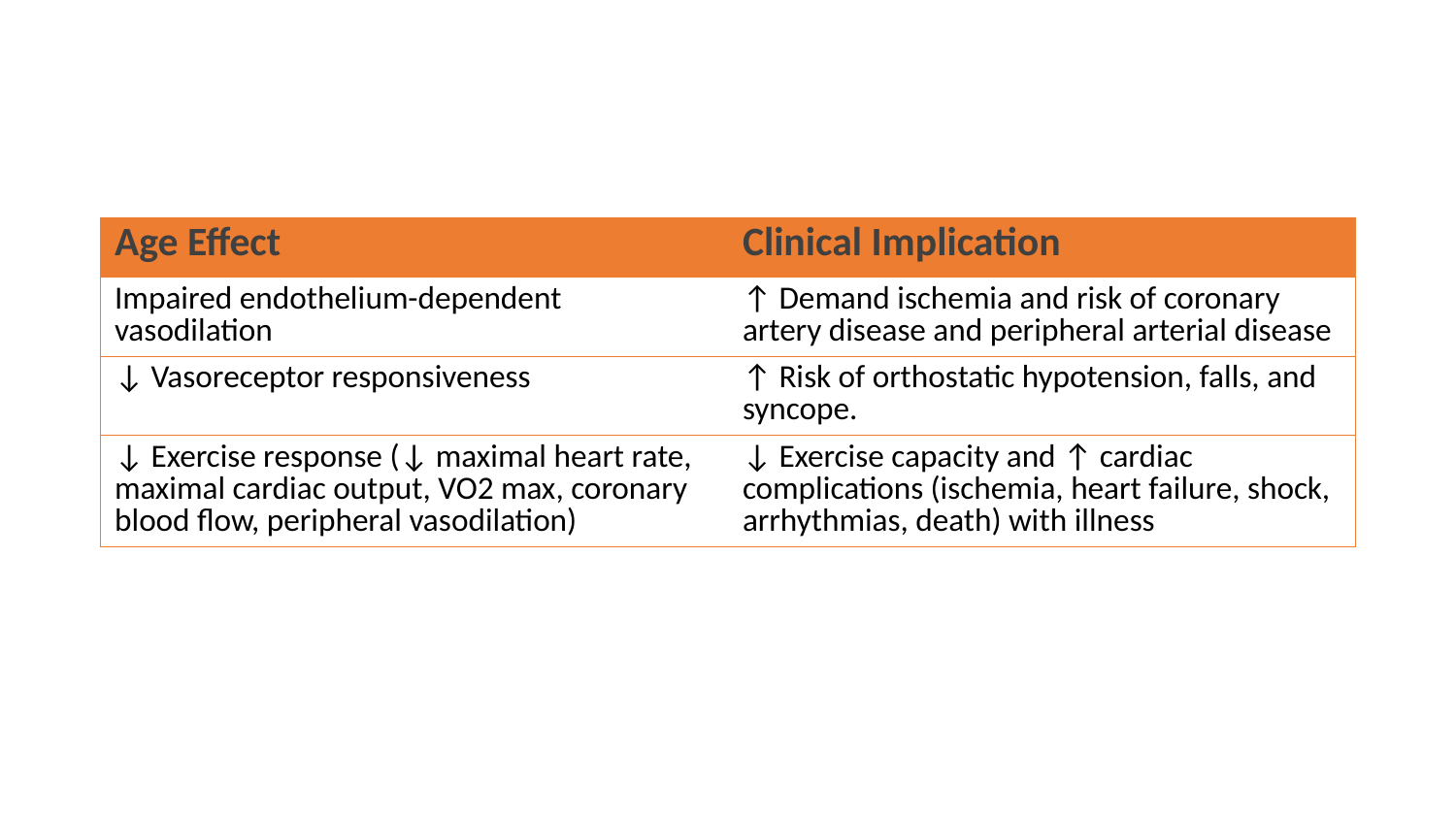

| Age Effect | Clinical Implication |
| --- | --- |
| Impaired endothelium-dependent vasodilation | ↑ Demand ischemia and risk of coronary artery disease and peripheral arterial disease |
| ↓ Vasoreceptor responsiveness | ↑ Risk of orthostatic hypotension, falls, and syncope. |
| ↓ Exercise response (↓ maximal heart rate, maximal cardiac output, VO2 max, coronary blood flow, peripheral vasodilation) | ↓ Exercise capacity and ↑ cardiac complications (ischemia, heart failure, shock, arrhythmias, death) with illness |

## Slide 8
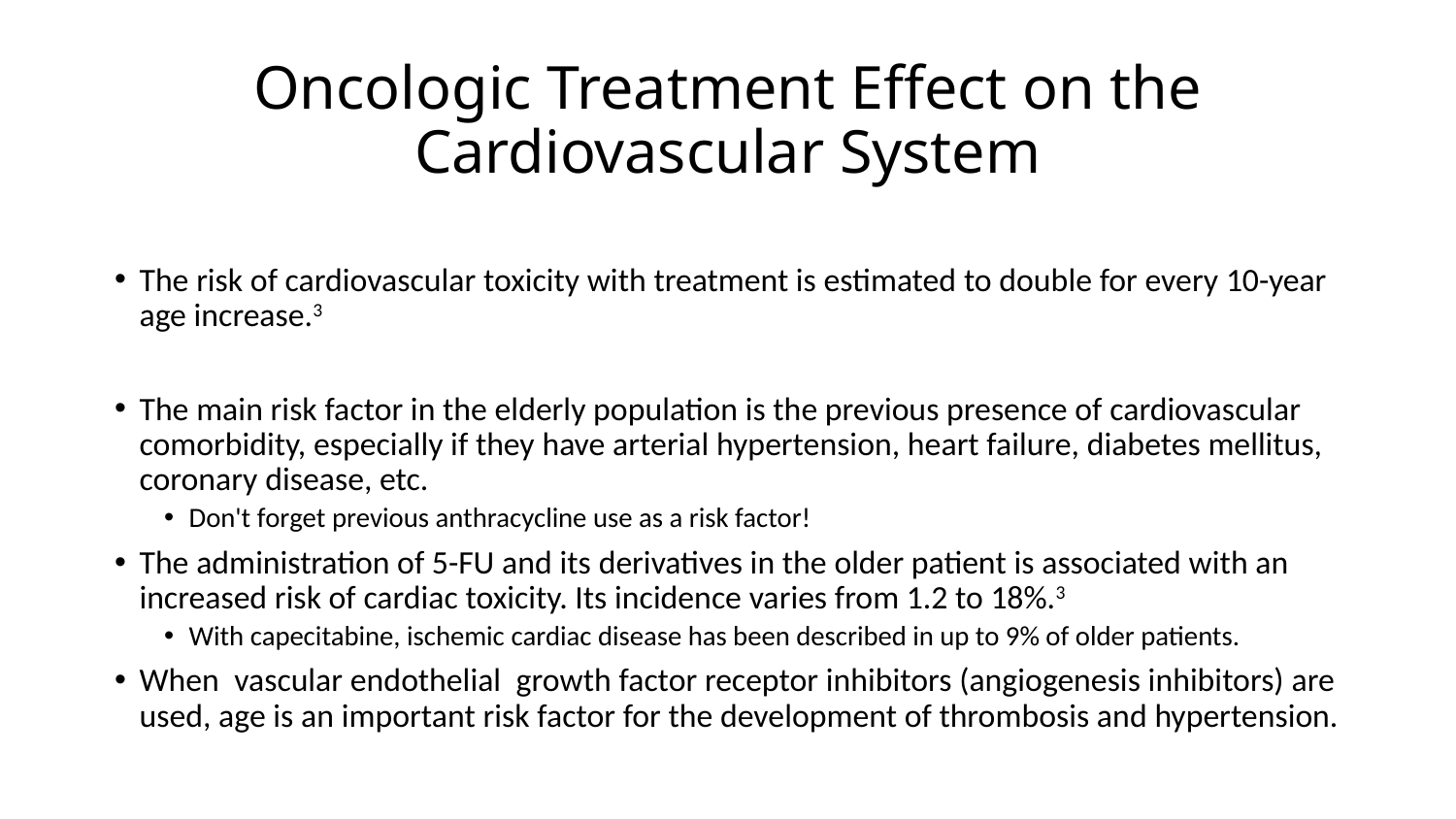

# Oncologic Treatment Effect on the Cardiovascular System
The risk of cardiovascular toxicity with treatment is estimated to double for every 10-year age increase.3
The main risk factor in the elderly population is the previous presence of cardiovascular comorbidity, especially if they have arterial hypertension, heart failure, diabetes mellitus, coronary disease, etc.
Don't forget previous anthracycline use as a risk factor!
The administration of 5-FU and its derivatives in the older patient is associated with an increased risk of cardiac toxicity. Its incidence varies from 1.2 to 18%.3
With capecitabine, ischemic cardiac disease has been described in up to 9% of older patients.
When vascular endothelial growth factor receptor inhibitors (angiogenesis inhibitors) are used, age is an important risk factor for the development of thrombosis and hypertension.

## Slide 9
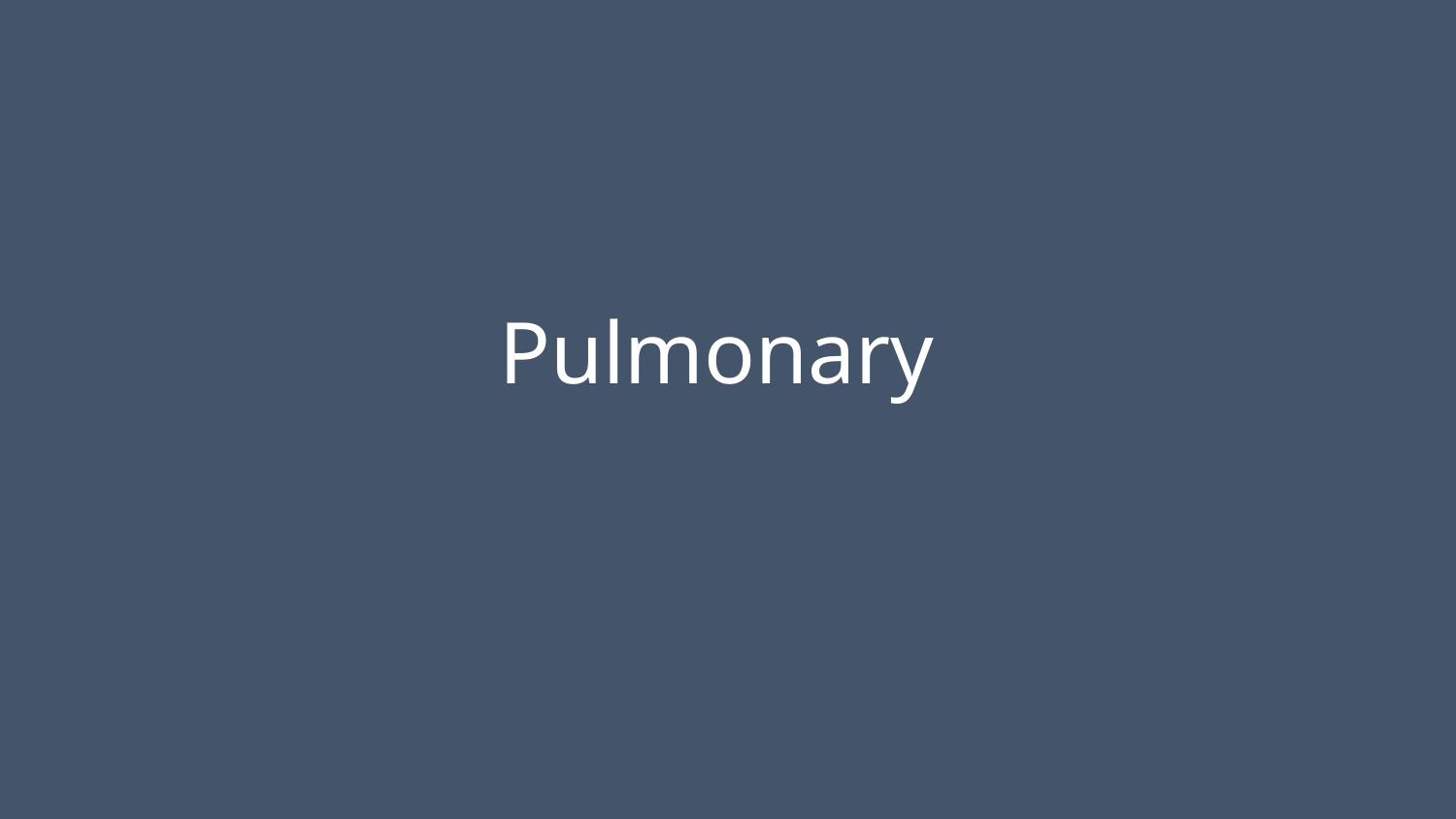

Pulmonary

## Slide 10
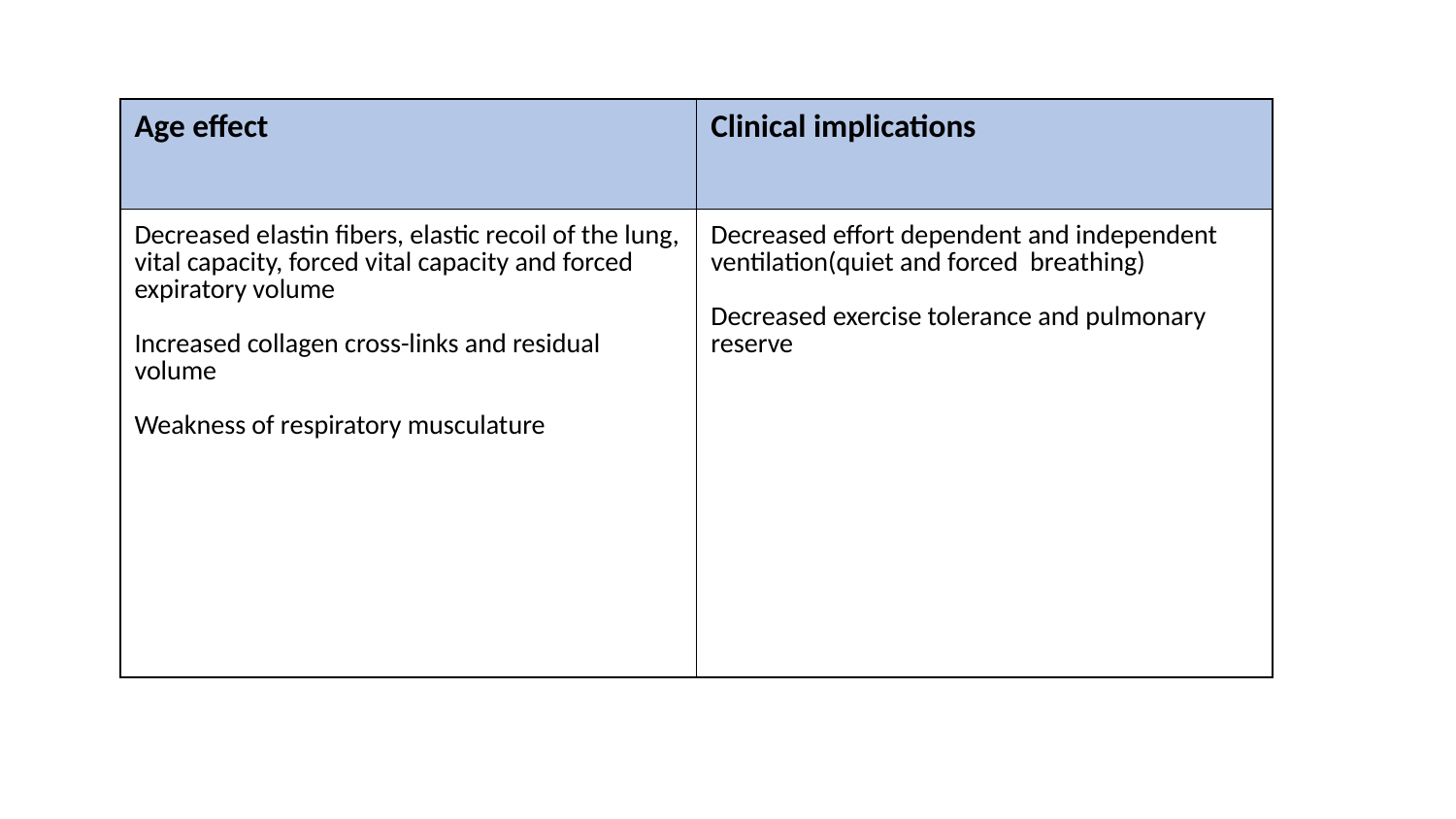

| Age effect | Clinical implications |
| --- | --- |
| Decreased elastin fibers, elastic recoil of the lung, vital capacity, forced vital capacity and forced expiratory volume Increased collagen cross-links and residual volume Weakness of respiratory musculature | Decreased effort dependent and independent ventilation(quiet and forced breathing) Decreased exercise tolerance and pulmonary reserve |

## Slide 11
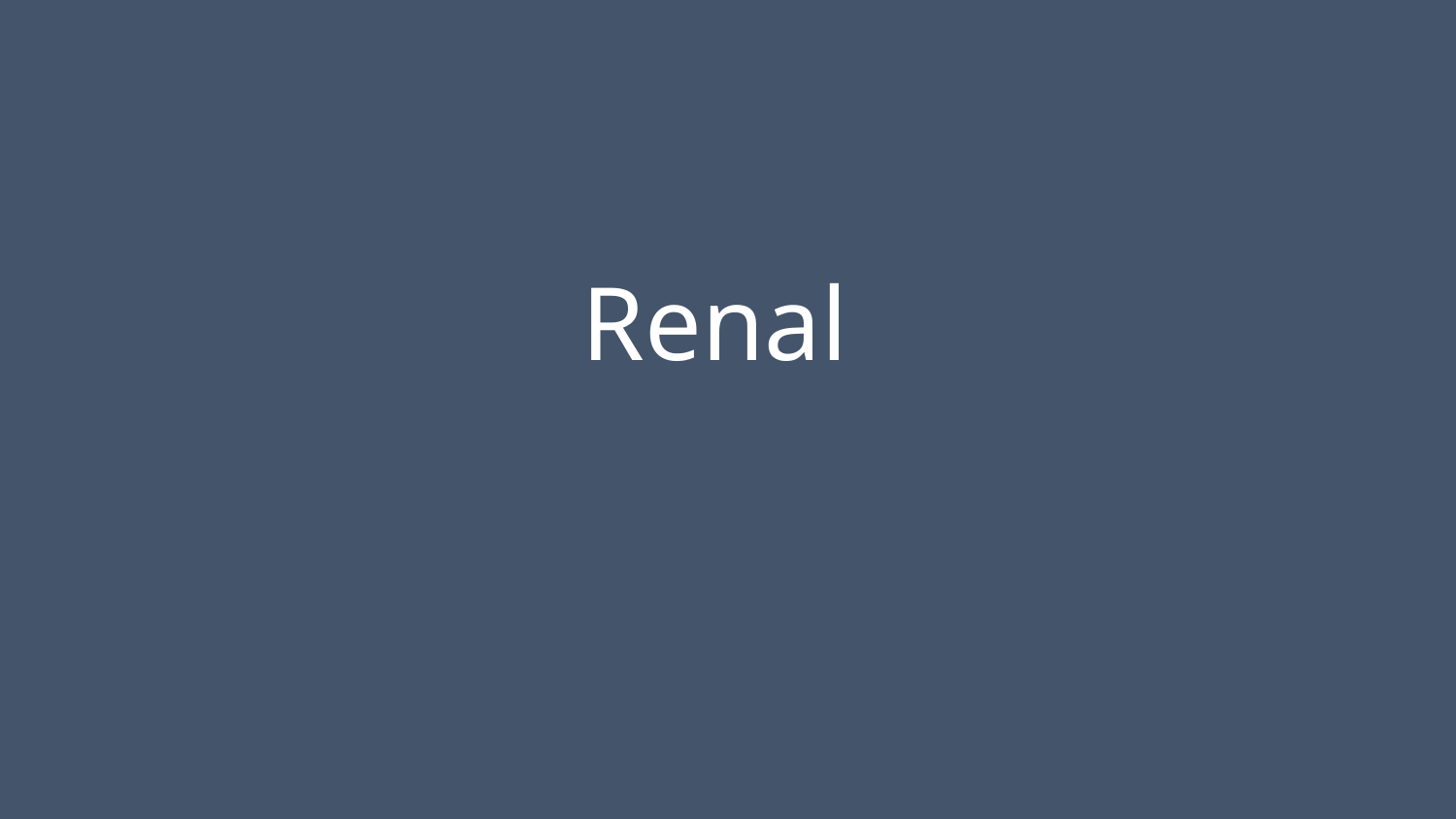

Renal

## Slide 12
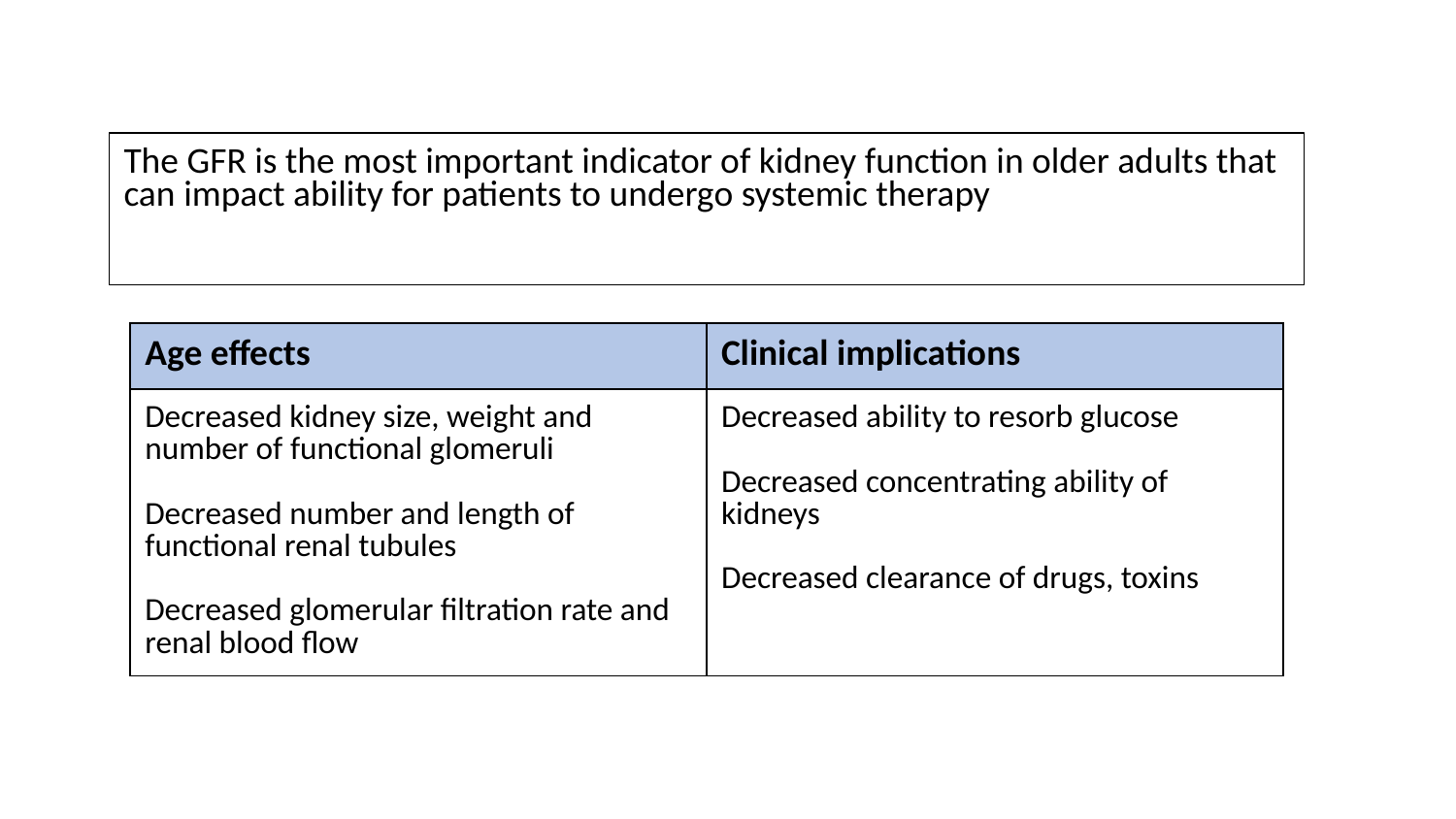

# The GFR is the most important indicator of kidney function in older adults that can impact ability for patients to undergo systemic therapy
| Age effects | Clinical implications |
| --- | --- |
| Decreased kidney size, weight and number of functional glomeruli Decreased number and length of functional renal tubules Decreased glomerular filtration rate and renal blood flow | Decreased ability to resorb glucose Decreased concentrating ability of kidneys Decreased clearance of drugs, toxins |

## Slide 13
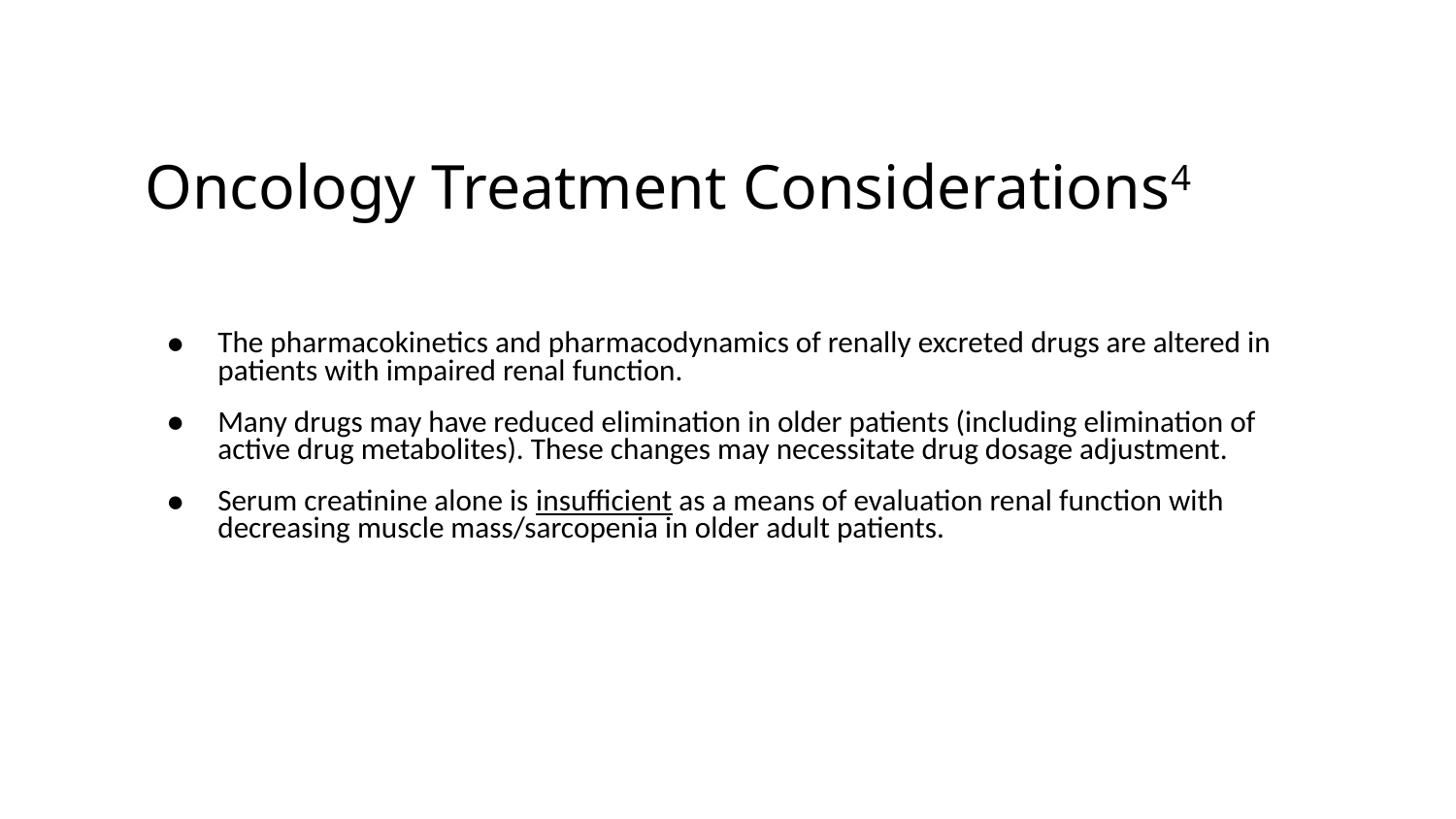

# Oncology Treatment Considerations4
The pharmacokinetics and pharmacodynamics of renally excreted drugs are altered in patients with impaired renal function.
Many drugs may have reduced elimination in older patients (including elimination of active drug metabolites). These changes may necessitate drug dosage adjustment.
Serum creatinine alone is insufficient as a means of evaluation renal function with decreasing muscle mass/sarcopenia in older adult patients.

## Slide 14
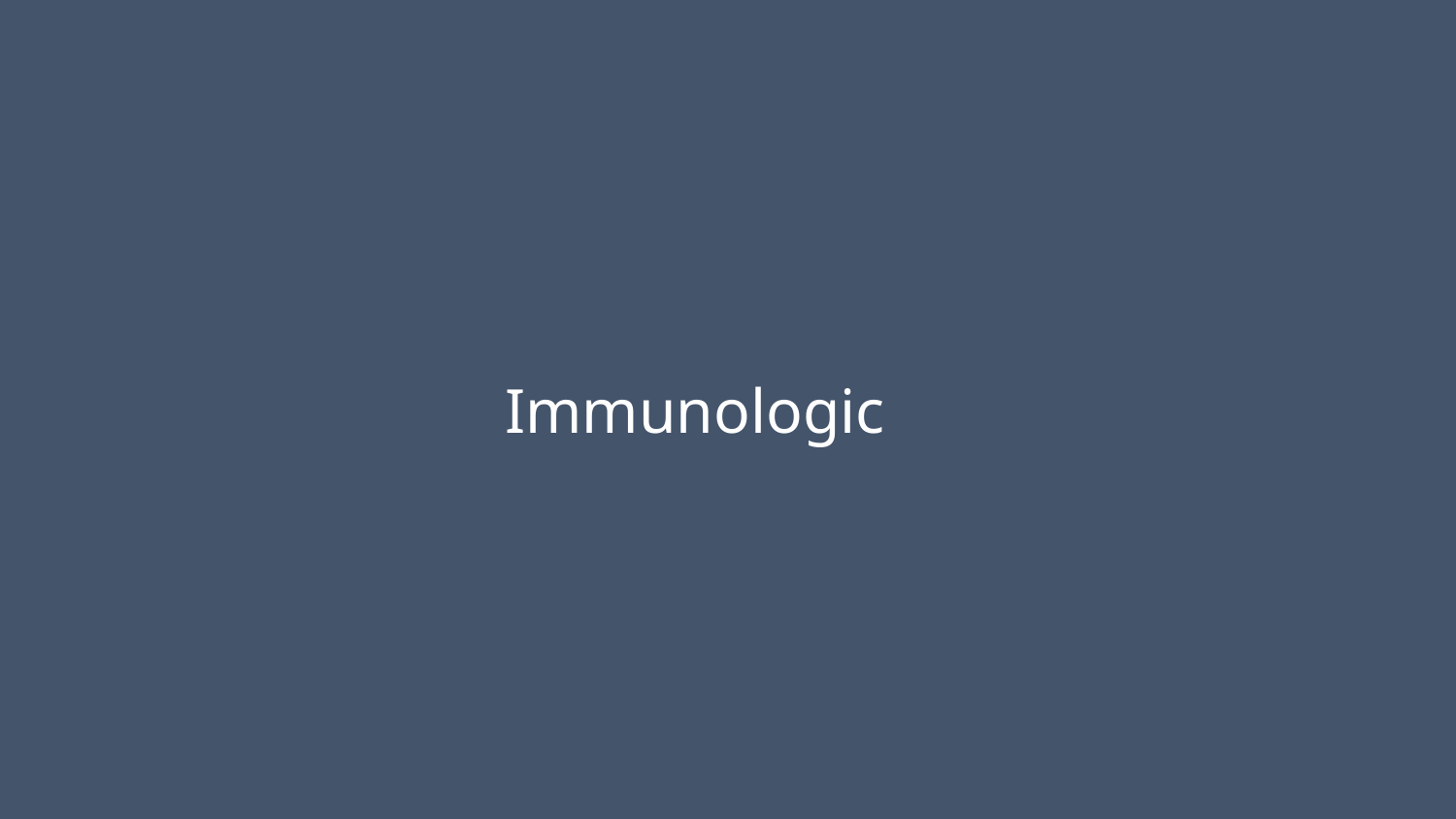

Immunologic

## Slide 15
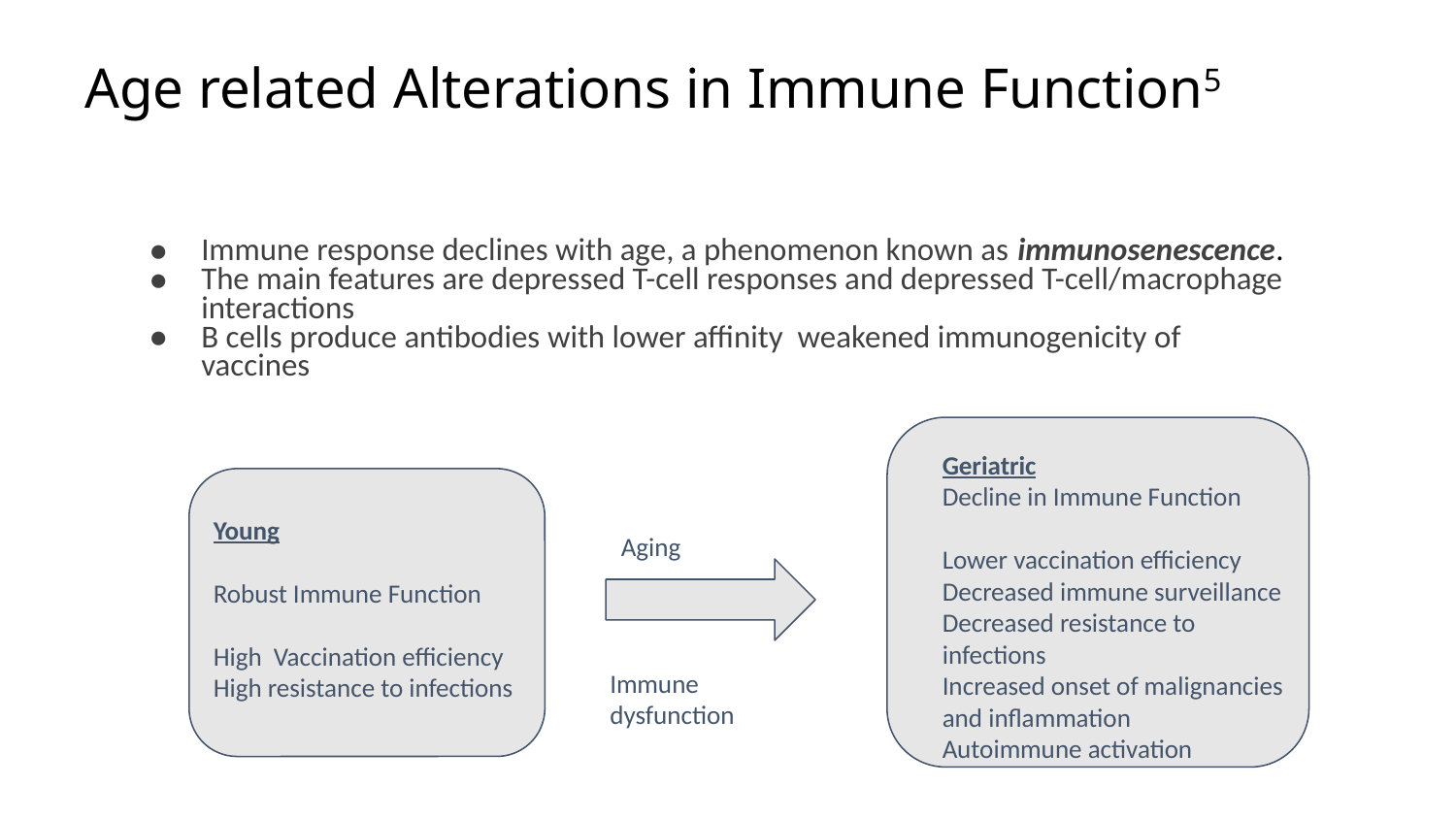

# Age related Alterations in Immune Function5
Immune response declines with age, a phenomenon known as immunosenescence​.
The main features are depressed T-cell responses and depressed T-cell/macrophage interactions​
B cells produce antibodies with lower affinity weakened immunogenicity of vaccines
Geriatric
Decline in Immune Function
Lower vaccination efficiency
Decreased immune surveillance
Decreased resistance to infections
Increased onset of malignancies and inflammation
Autoimmune activation
Young
Robust Immune Function
High Vaccination efficiency
High resistance to infections
Aging
Immune dysfunction

## Slide 16
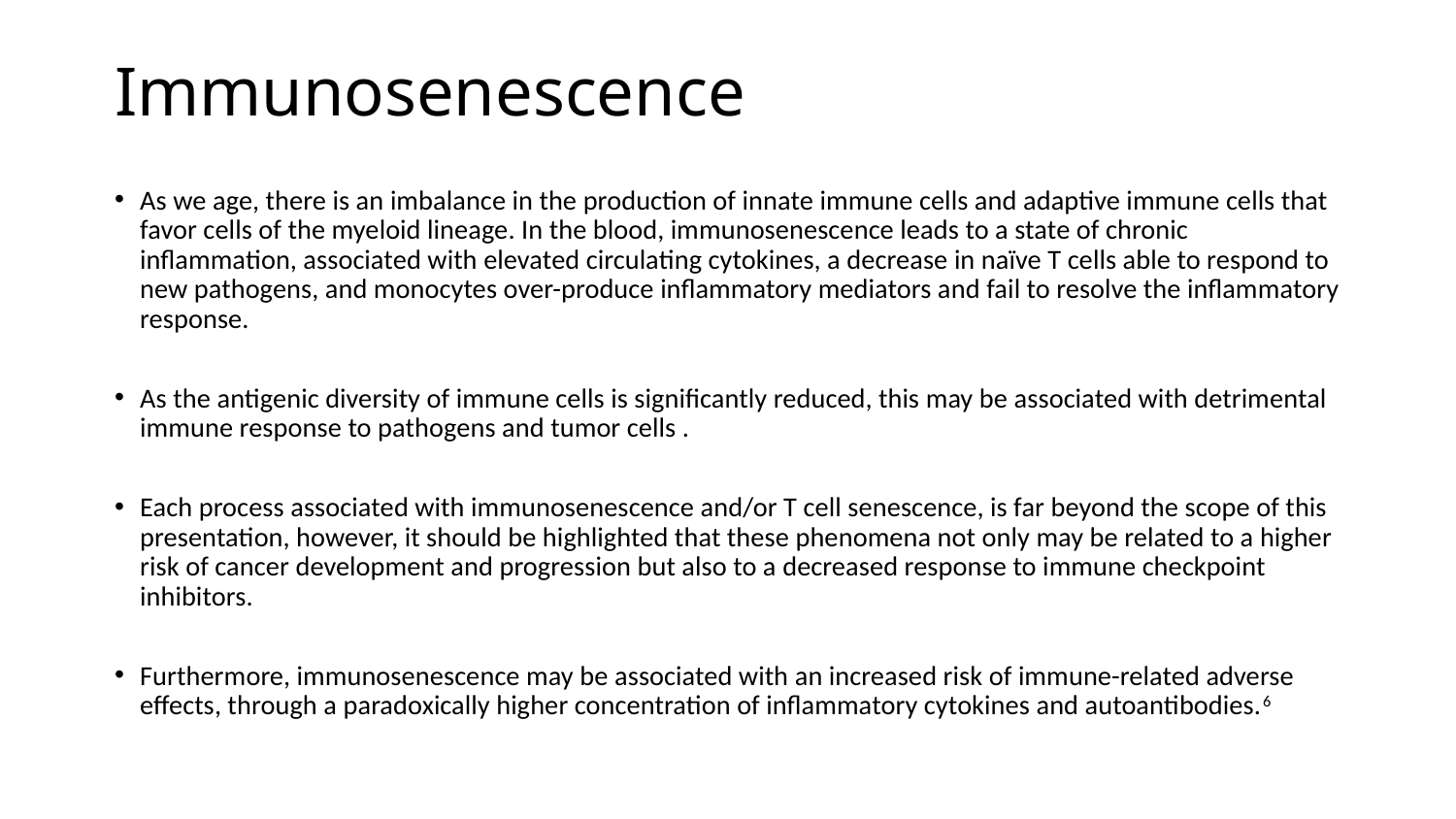

# Immunosenescence
As we age, there is an imbalance in the production of innate immune cells and adaptive immune cells that favor cells of the myeloid lineage. In the blood, immunosenescence leads to a state of chronic inflammation, associated with elevated circulating cytokines, a decrease in naïve T cells able to respond to new pathogens, and monocytes over-produce inflammatory mediators and fail to resolve the inflammatory response.
As the antigenic diversity of immune cells is significantly reduced, this may be associated with detrimental immune response to pathogens and tumor cells .
Each process associated with immunosenescence and/or T cell senescence, is far beyond the scope of this presentation, however, it should be highlighted that these phenomena not only may be related to a higher risk of cancer development and progression but also to a decreased response to immune checkpoint inhibitors.
Furthermore, immunosenescence may be associated with an increased risk of immune-related adverse effects, through a paradoxically higher concentration of inflammatory cytokines and autoantibodies.6

## Slide 17
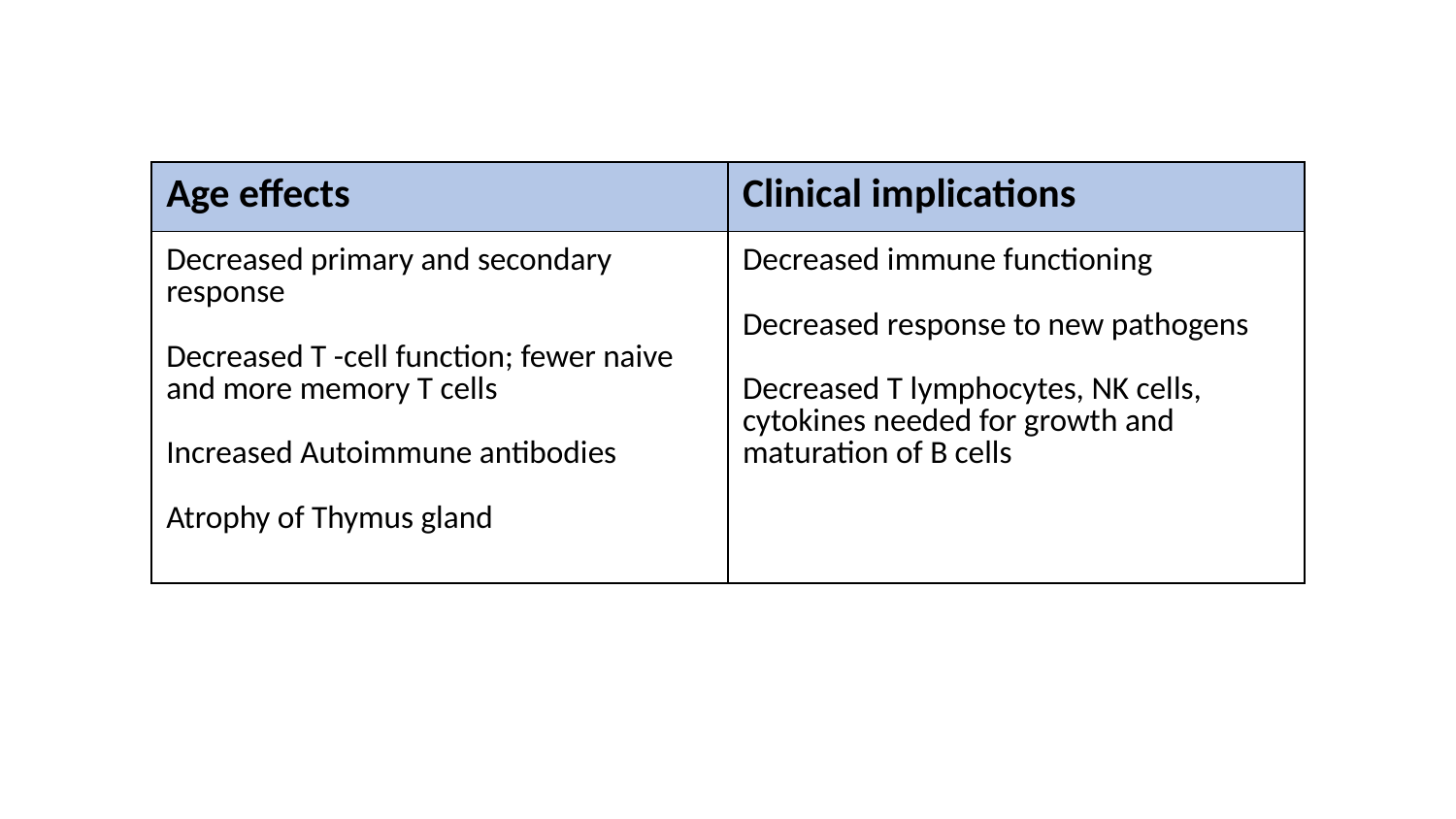

| Age effects | Clinical implications |
| --- | --- |
| Decreased primary and secondary response Decreased T -cell function; fewer naive and more memory T cells Increased Autoimmune antibodies Atrophy of Thymus gland | Decreased immune functioning Decreased response to new pathogens Decreased T lymphocytes, NK cells, cytokines needed for growth and maturation of B cells |

## Slide 18
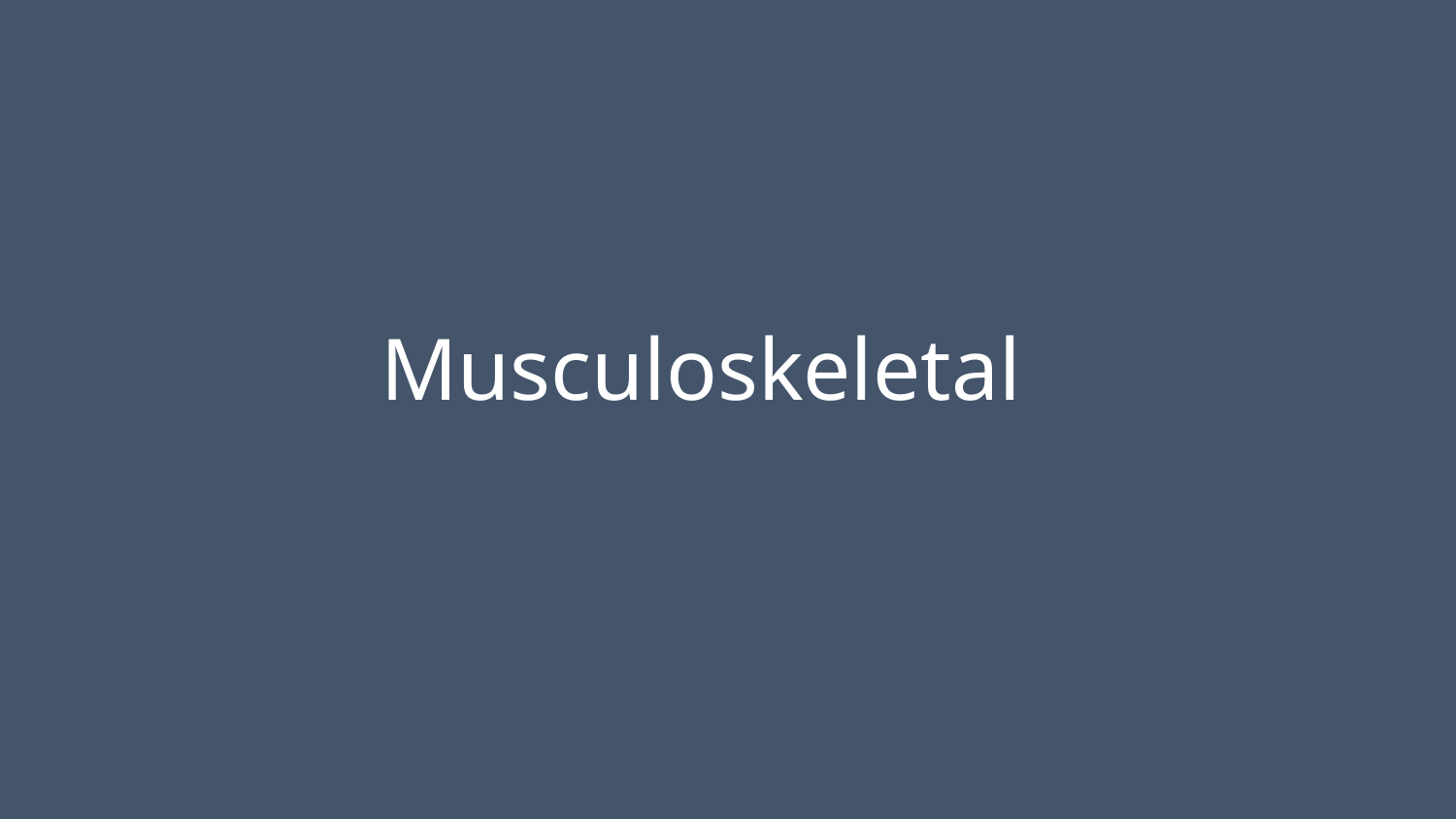

# Musculoskeletal

## Slide 19
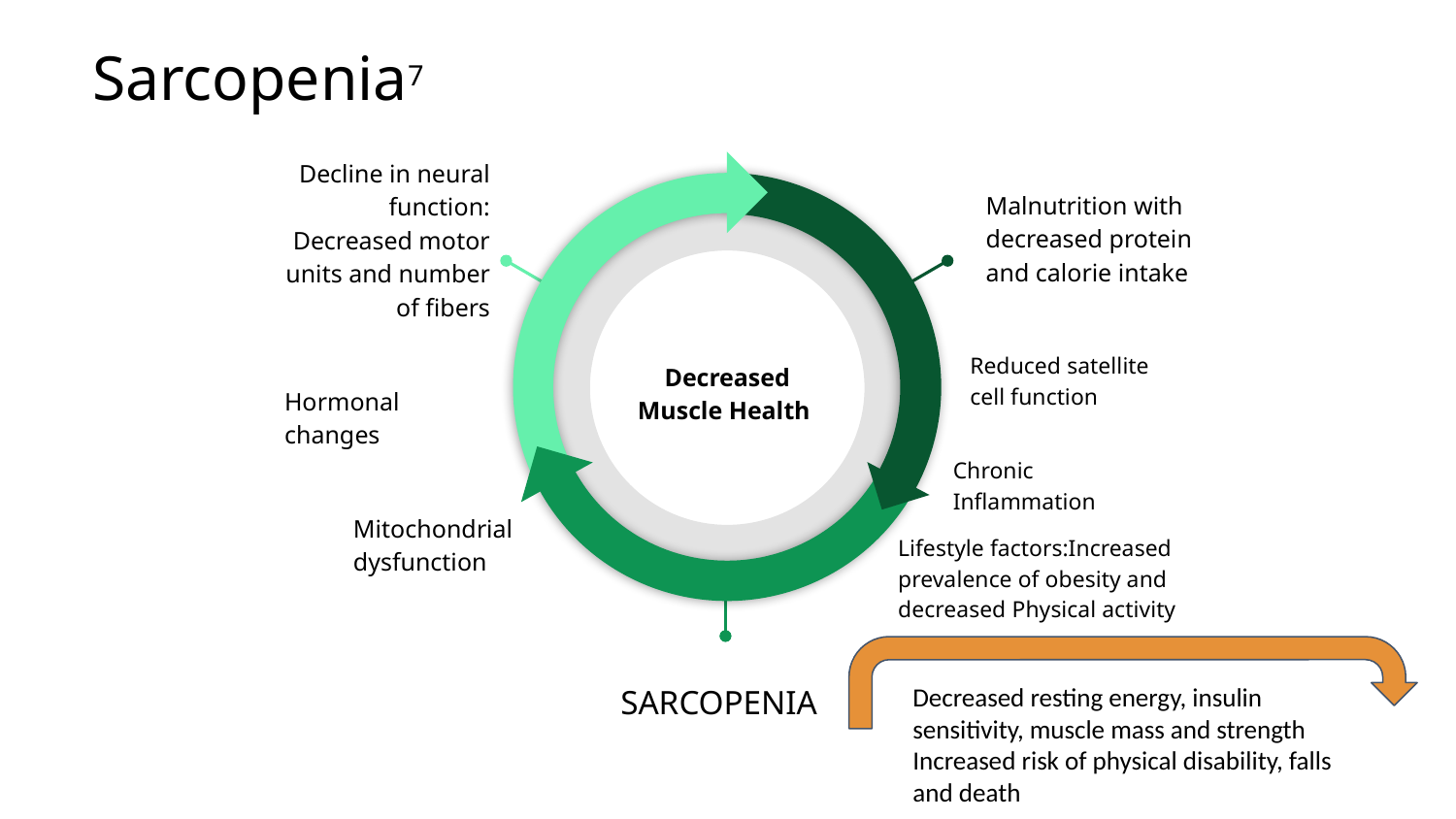

# Sarcopenia7
Decline in neural function: Decreased motor units and number of fibers
Malnutrition with decreased protein and calorie intake
Decreased Muscle Health
Reduced satellite cell function
Hormonal changes
Chronic Inflammation
Mitochondrial dysfunction
Lifestyle factors:Increased prevalence of obesity and decreased Physical activity
SARCOPENIA
Decreased resting energy, insulin sensitivity, muscle mass and strength
Increased risk of physical disability, falls and death

## Slide 20
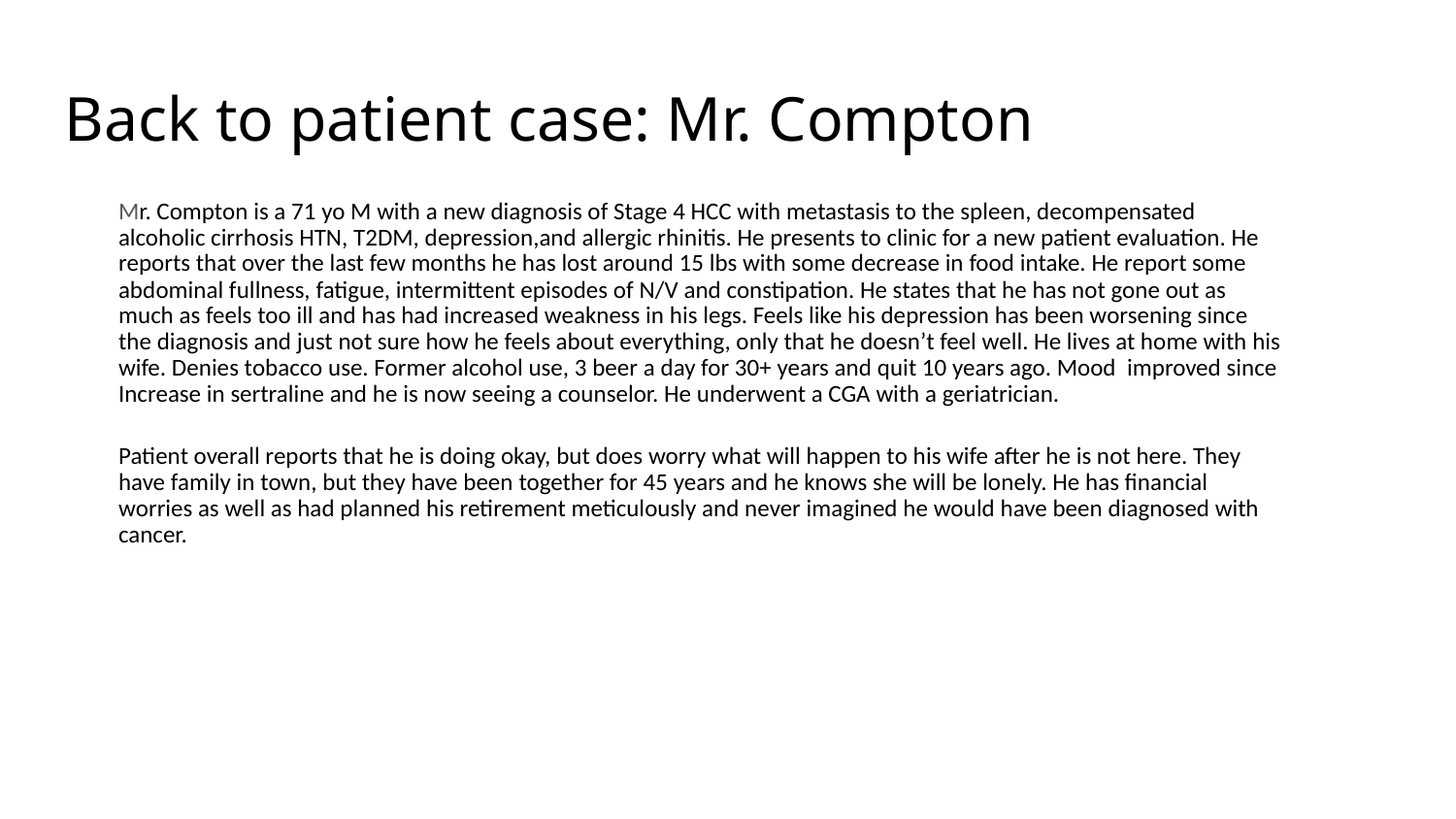

# Back to patient case: Mr. Compton
Mr. Compton is a 71 yo M with a new diagnosis of Stage 4 HCC with metastasis to the spleen, decompensated alcoholic cirrhosis HTN, T2DM, depression,and allergic rhinitis. He presents to clinic for a new patient evaluation. He reports that over the last few months he has lost around 15 lbs with some decrease in food intake. He report some abdominal fullness, fatigue, intermittent episodes of N/V and constipation. He states that he has not gone out as much as feels too ill and has had increased weakness in his legs. Feels like his depression has been worsening since the diagnosis and just not sure how he feels about everything, only that he doesn’t feel well. He lives at home with his wife. Denies tobacco use. Former alcohol use, 3 beer a day for 30+ years and quit 10 years ago. Mood improved since Increase in sertraline and he is now seeing a counselor. He underwent a CGA with a geriatrician.
Patient overall reports that he is doing okay, but does worry what will happen to his wife after he is not here. They have family in town, but they have been together for 45 years and he knows she will be lonely. He has financial worries as well as had planned his retirement meticulously and never imagined he would have been diagnosed with cancer.

## Slide 21
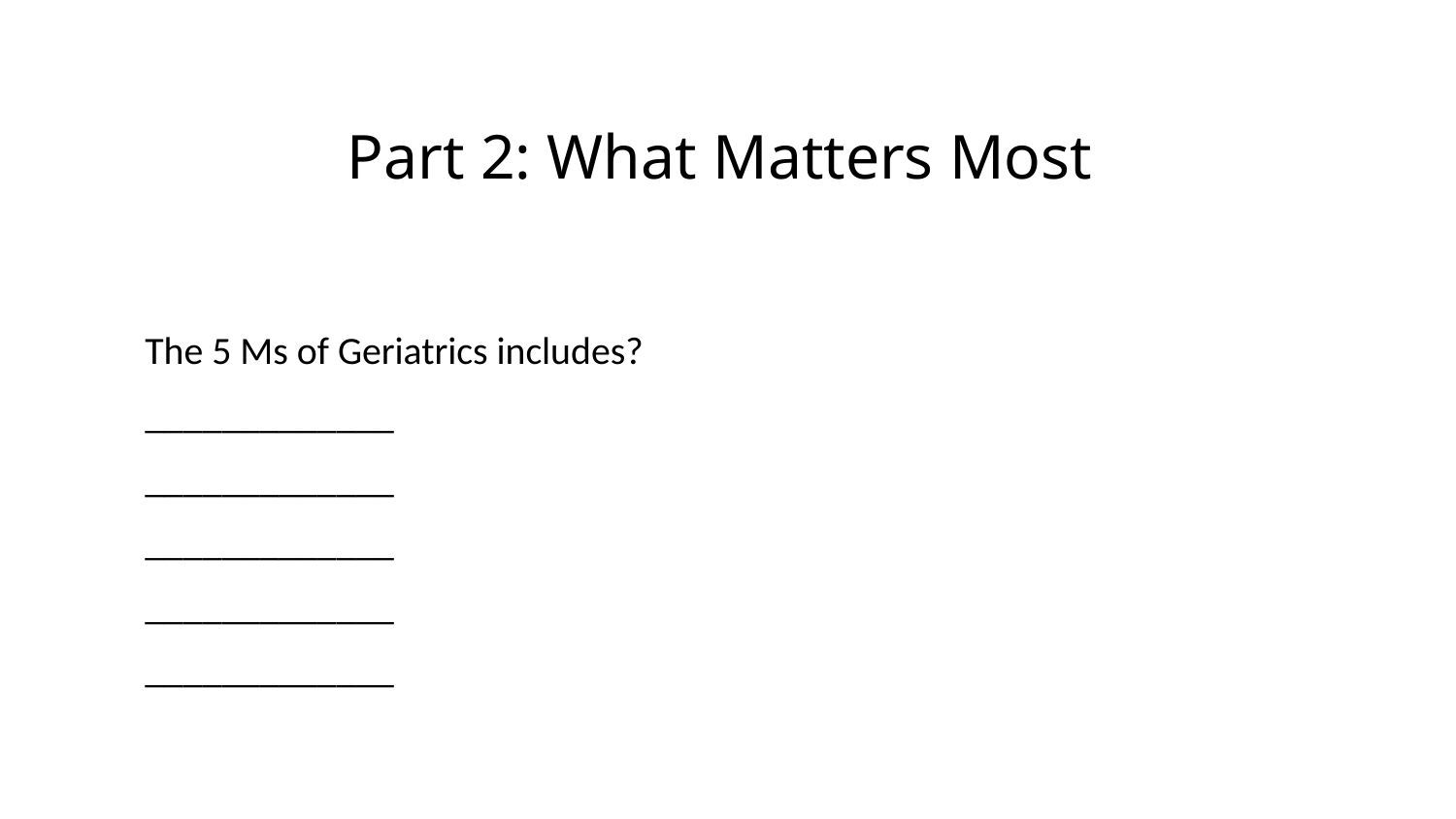

# Part 2: What Matters Most
The 5 Ms of Geriatrics includes?
_____________
_____________
_____________
_____________
_____________

## Slide 22
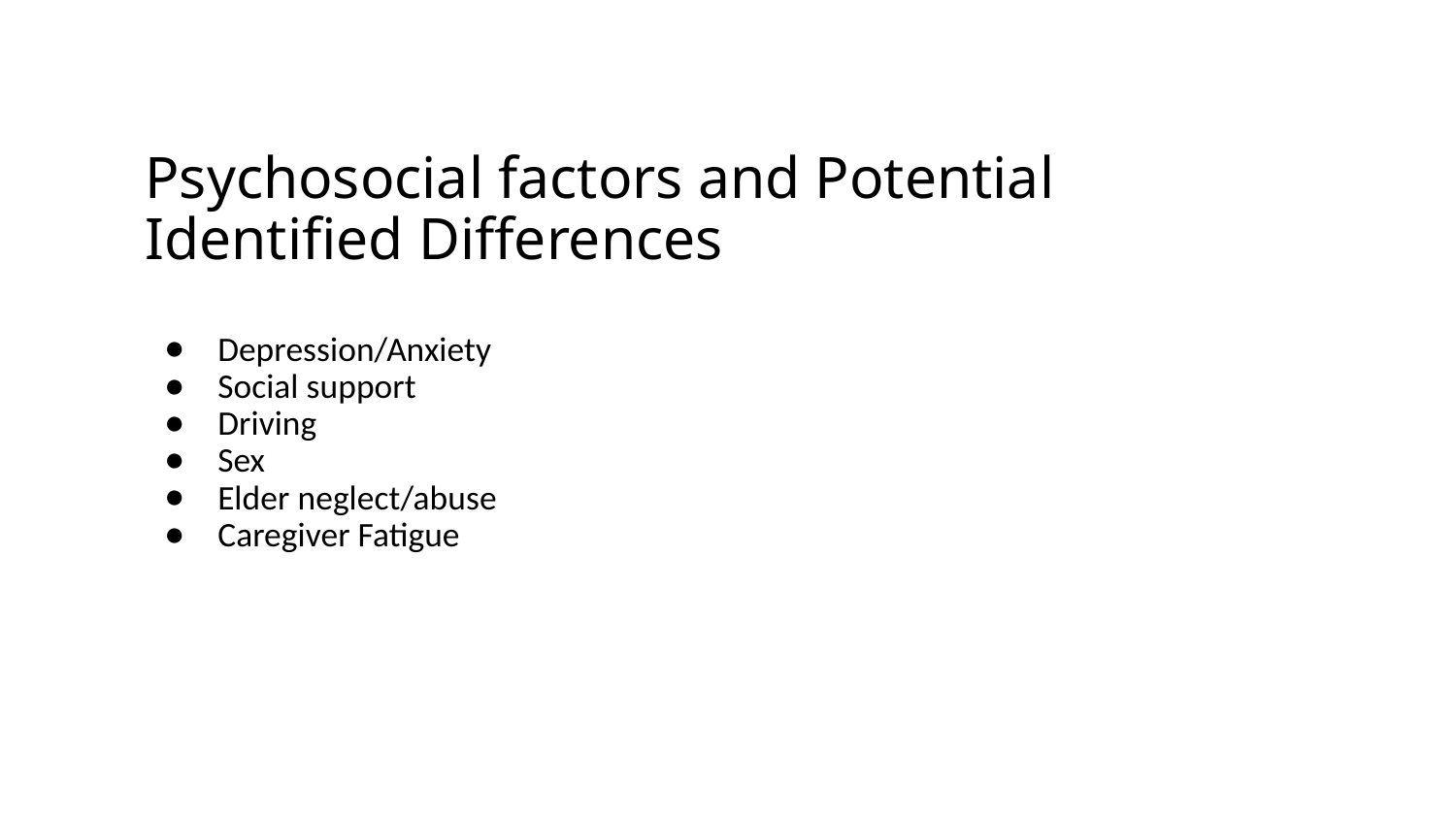

# Psychosocial factors and Potential Identified Differences
Depression/Anxiety
Social support
Driving
Sex
Elder neglect/abuse
Caregiver Fatigue

## Slide 23
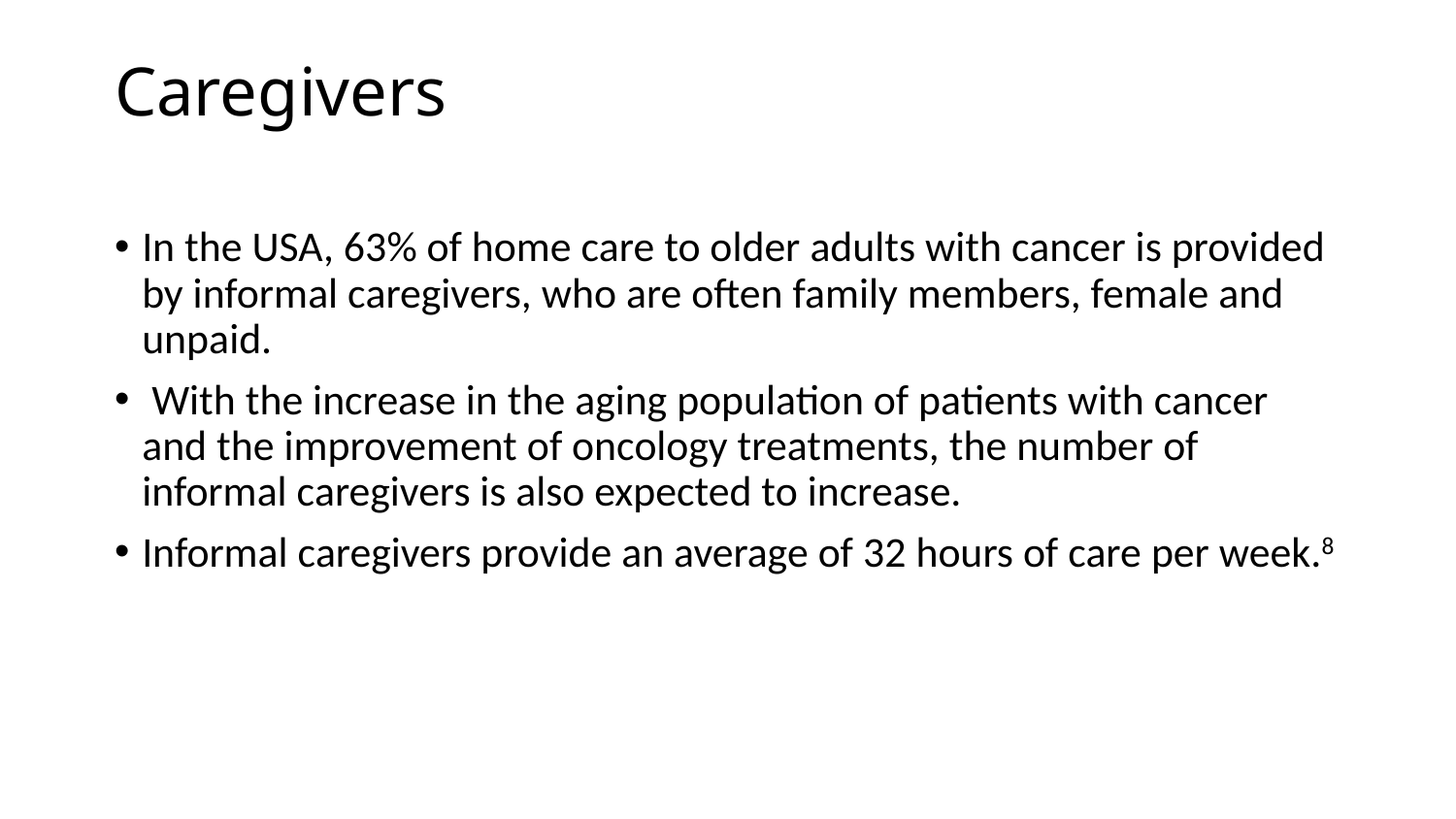

# Caregivers
In the USA, 63% of home care to older adults with cancer is provided by informal caregivers, who are often family members, female and unpaid.
 With the increase in the aging population of patients with cancer and the improvement of oncology treatments, the number of informal caregivers is also expected to increase.
Informal caregivers provide an average of 32 hours of care per week.8

## Slide 24
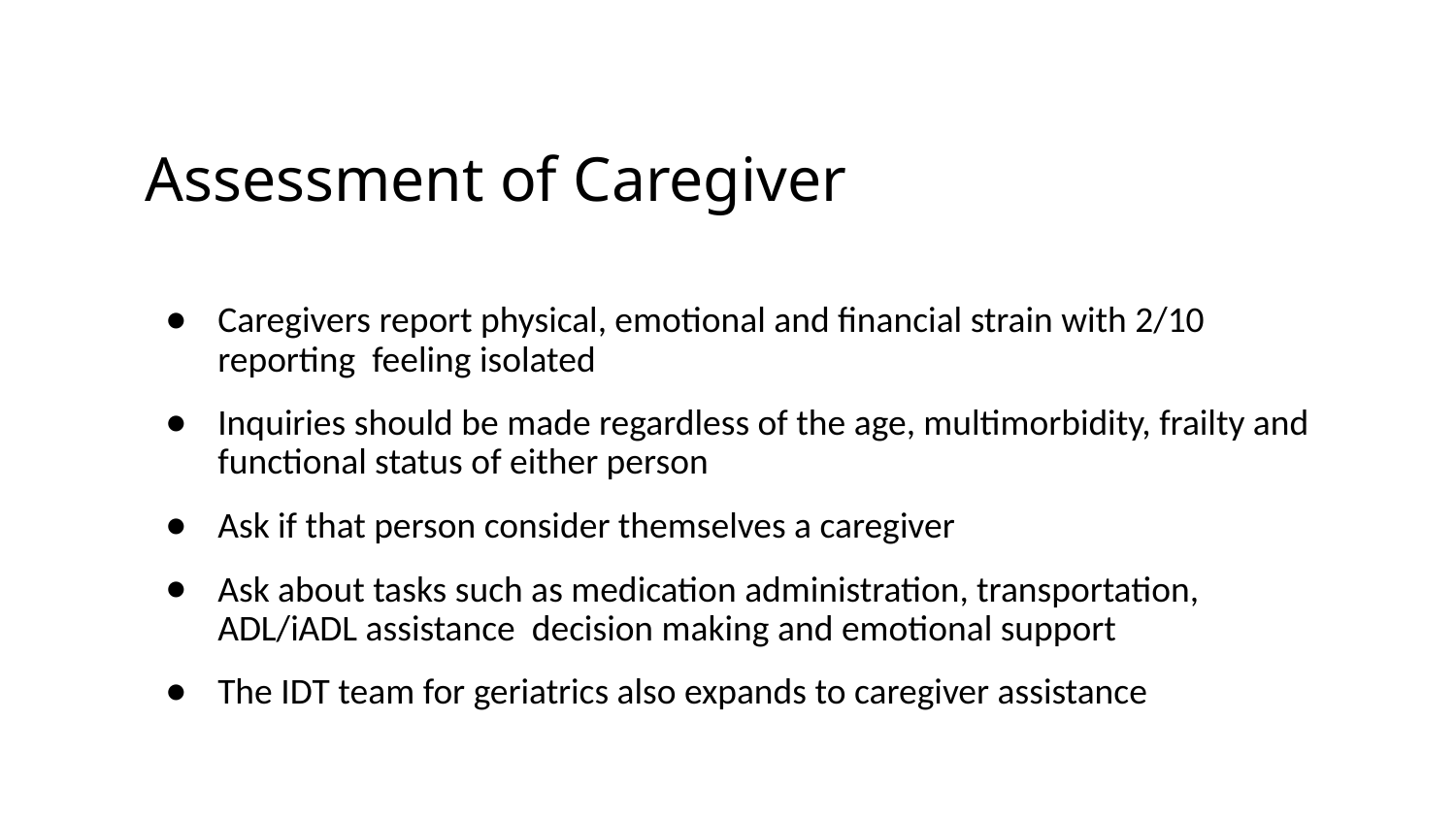

# Assessment of Caregiver
Caregivers report physical, emotional and financial strain with 2/10 reporting feeling isolated
Inquiries should be made regardless of the age, multimorbidity, frailty and functional status of either person
Ask if that person consider themselves a caregiver
Ask about tasks such as medication administration, transportation, ADL/iADL assistance decision making and emotional support
The IDT team for geriatrics also expands to caregiver assistance

## Slide 25
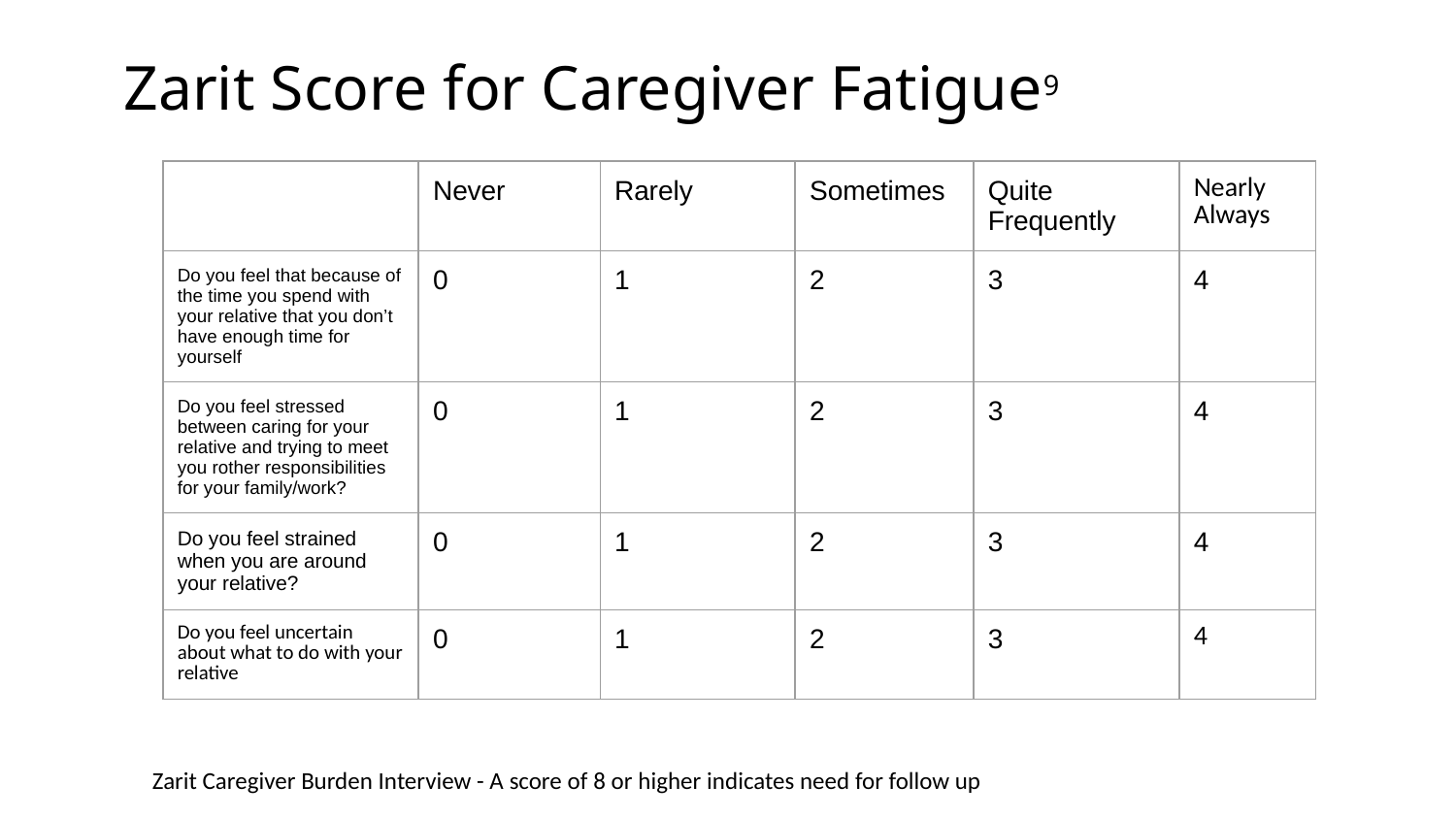

# Zarit Score for Caregiver Fatigue9
| | Never | Rarely | Sometimes | Quite Frequently | Nearly Always |
| --- | --- | --- | --- | --- | --- |
| Do you feel that because of the time you spend with your relative that you don’t have enough time for yourself | 0 | 1 | 2 | 3 | 4 |
| Do you feel stressed between caring for your relative and trying to meet you rother responsibilities for your family/work? | 0 | 1 | 2 | 3 | 4 |
| Do you feel strained when you are around your relative? | 0 | 1 | 2 | 3 | 4 |
| Do you feel uncertain about what to do with your relative | 0 | 1 | 2 | 3 | 4 |
Zarit Caregiver Burden Interview - A score of 8 or higher indicates need for follow up

## Slide 26
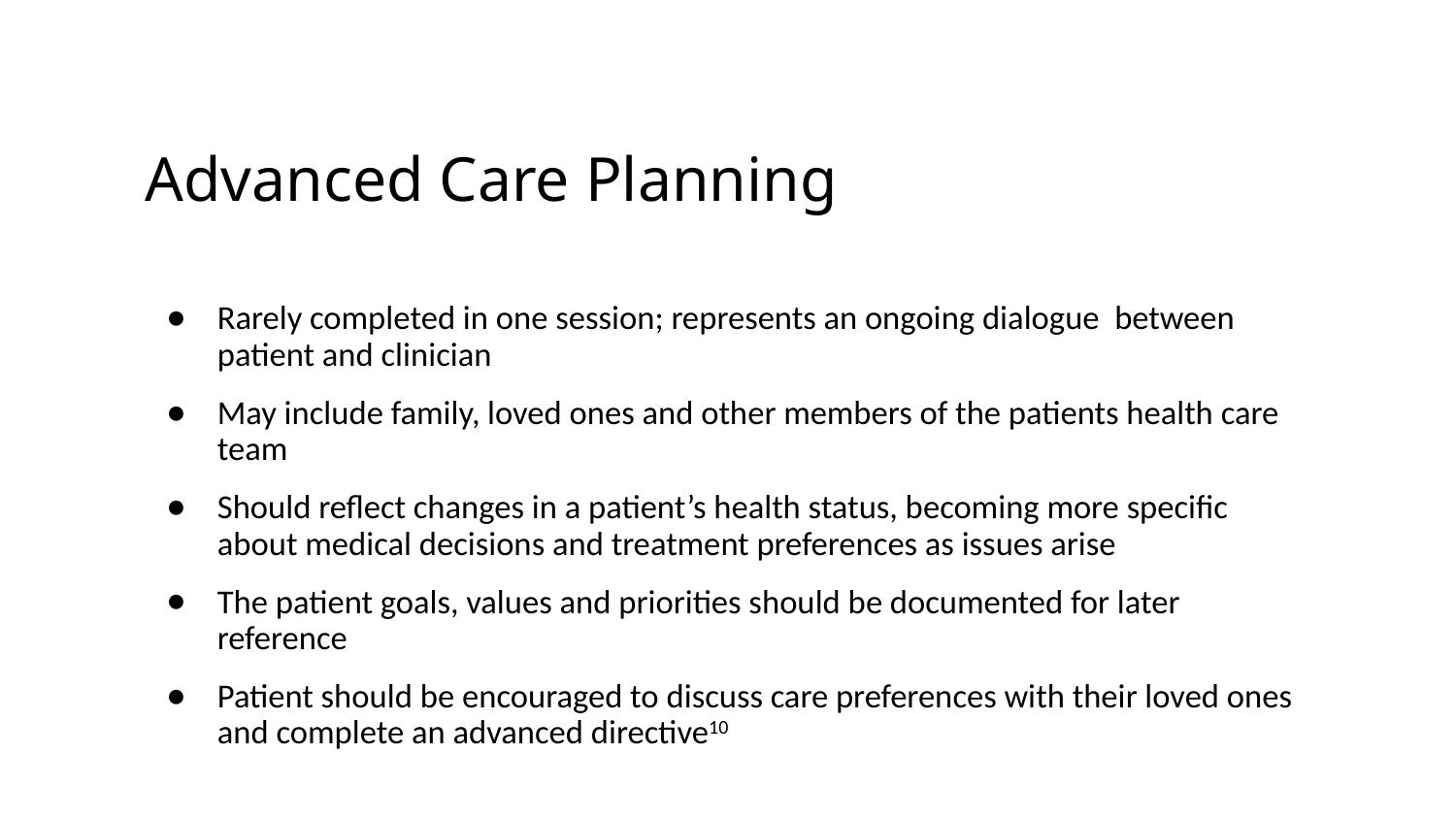

# Advanced Care Planning
Rarely completed in one session; represents an ongoing dialogue between patient and clinician
May include family, loved ones and other members of the patients health care team
Should reflect changes in a patient’s health status, becoming more specific about medical decisions and treatment preferences as issues arise
The patient goals, values and priorities should be documented for later reference
Patient should be encouraged to discuss care preferences with their loved ones and complete an advanced directive10

## Slide 27
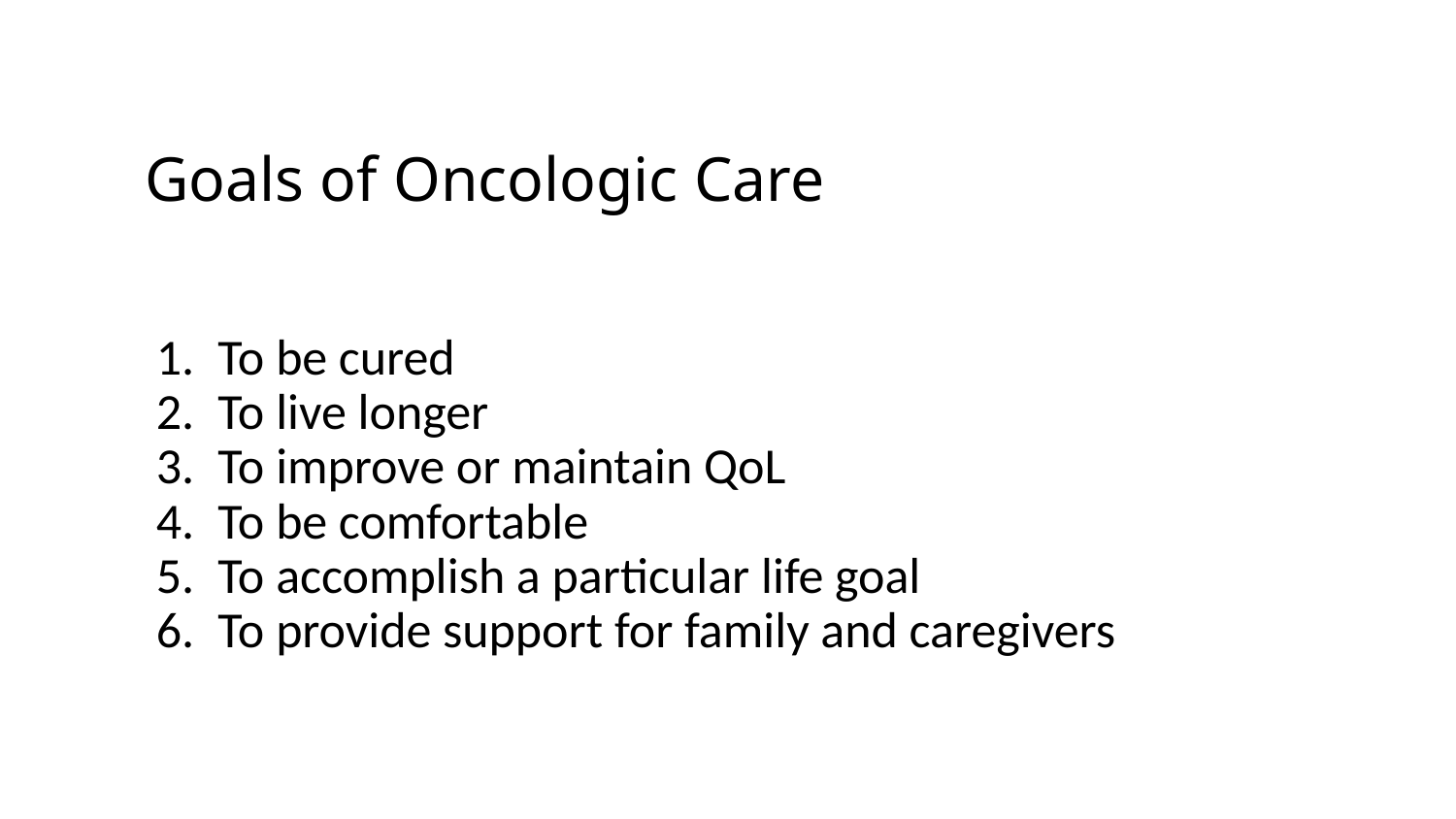

# Goals of Oncologic Care
To be cured
To live longer
To improve or maintain QoL
To be comfortable
To accomplish a particular life goal
To provide support for family and caregivers

## Slide 28
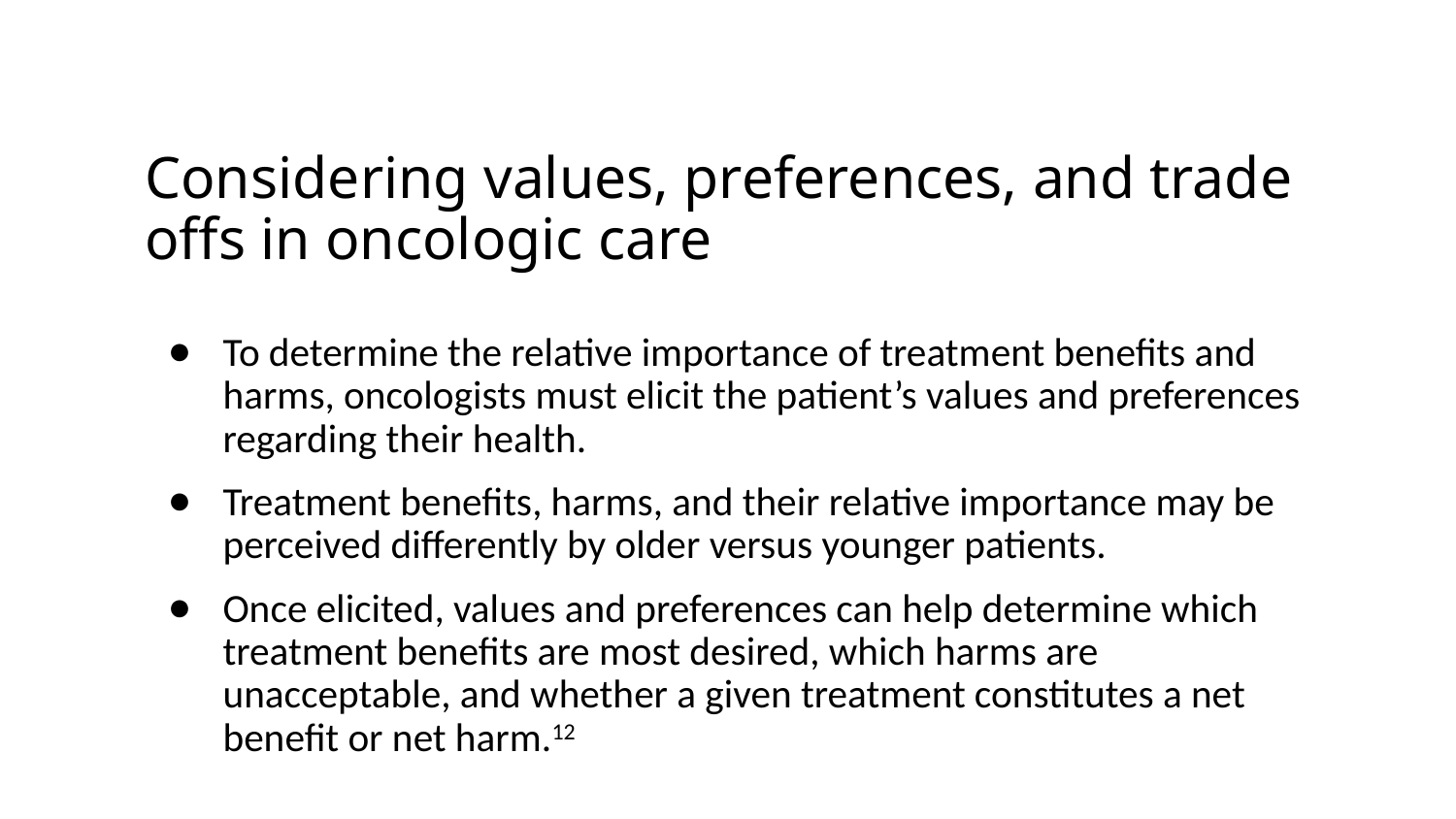

# Considering values, preferences, and trade offs in oncologic care
To determine the relative importance of treatment benefits and harms, oncologists must elicit the patient’s values and preferences regarding their health.
Treatment benefits, harms, and their relative importance may be perceived differently by older versus younger patients.
Once elicited, values and preferences can help determine which treatment benefits are most desired, which harms are unacceptable, and whether a given treatment constitutes a net benefit or net harm.12

## Slide 29
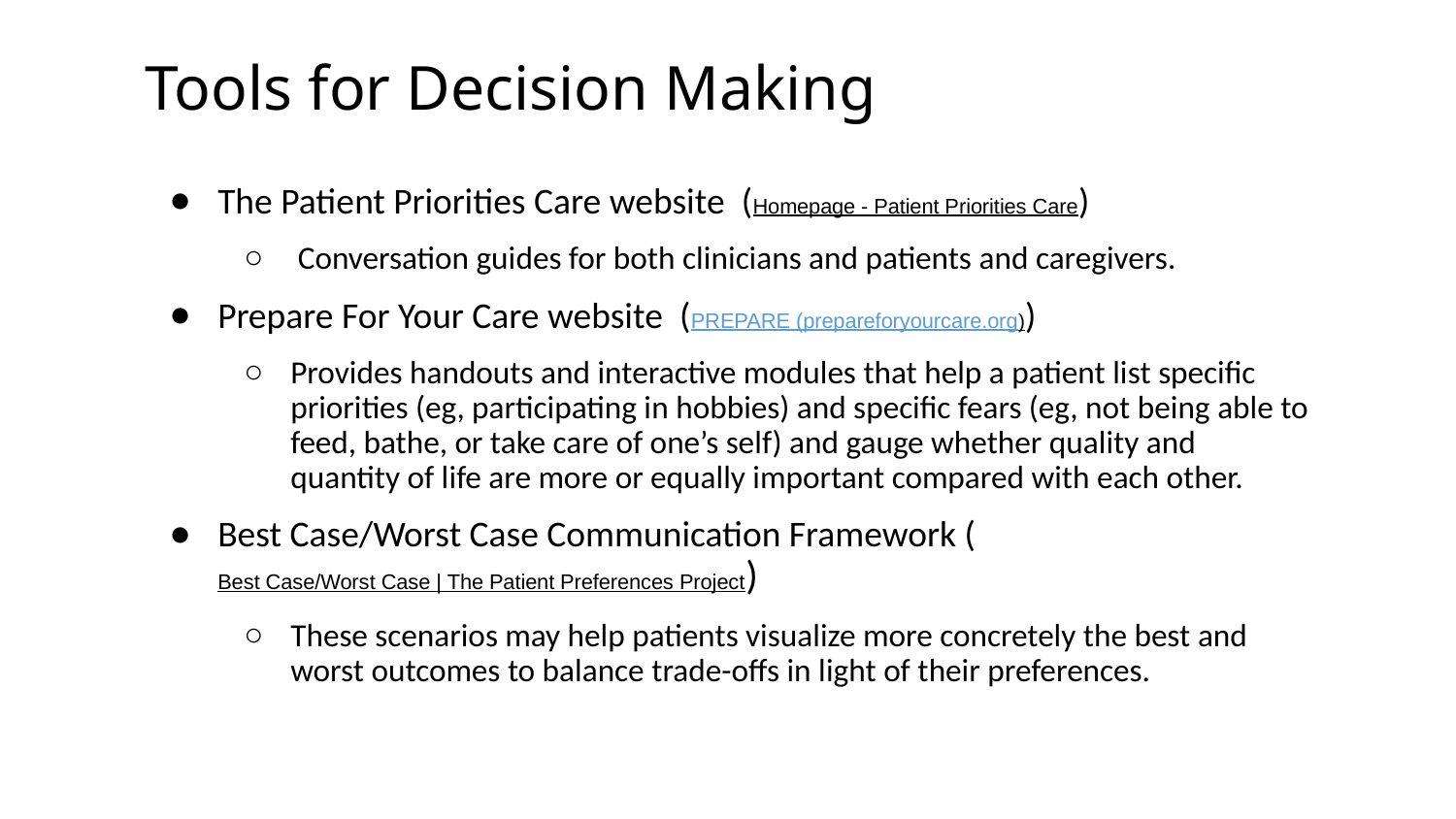

# Tools for Decision Making
The Patient Priorities Care website (Homepage - Patient Priorities Care)
 Conversation guides for both clinicians and patients and caregivers.
Prepare For Your Care website (PREPARE (prepareforyourcare.org))
Provides handouts and interactive modules that help a patient list specific priorities (eg, participating in hobbies) and specific fears (eg, not being able to feed, bathe, or take care of one’s self) and gauge whether quality and quantity of life are more or equally important compared with each other.
Best Case/Worst Case Communication Framework (Best Case/Worst Case | The Patient Preferences Project)
These scenarios may help patients visualize more concretely the best and worst outcomes to balance trade-offs in light of their preferences.

## Slide 30
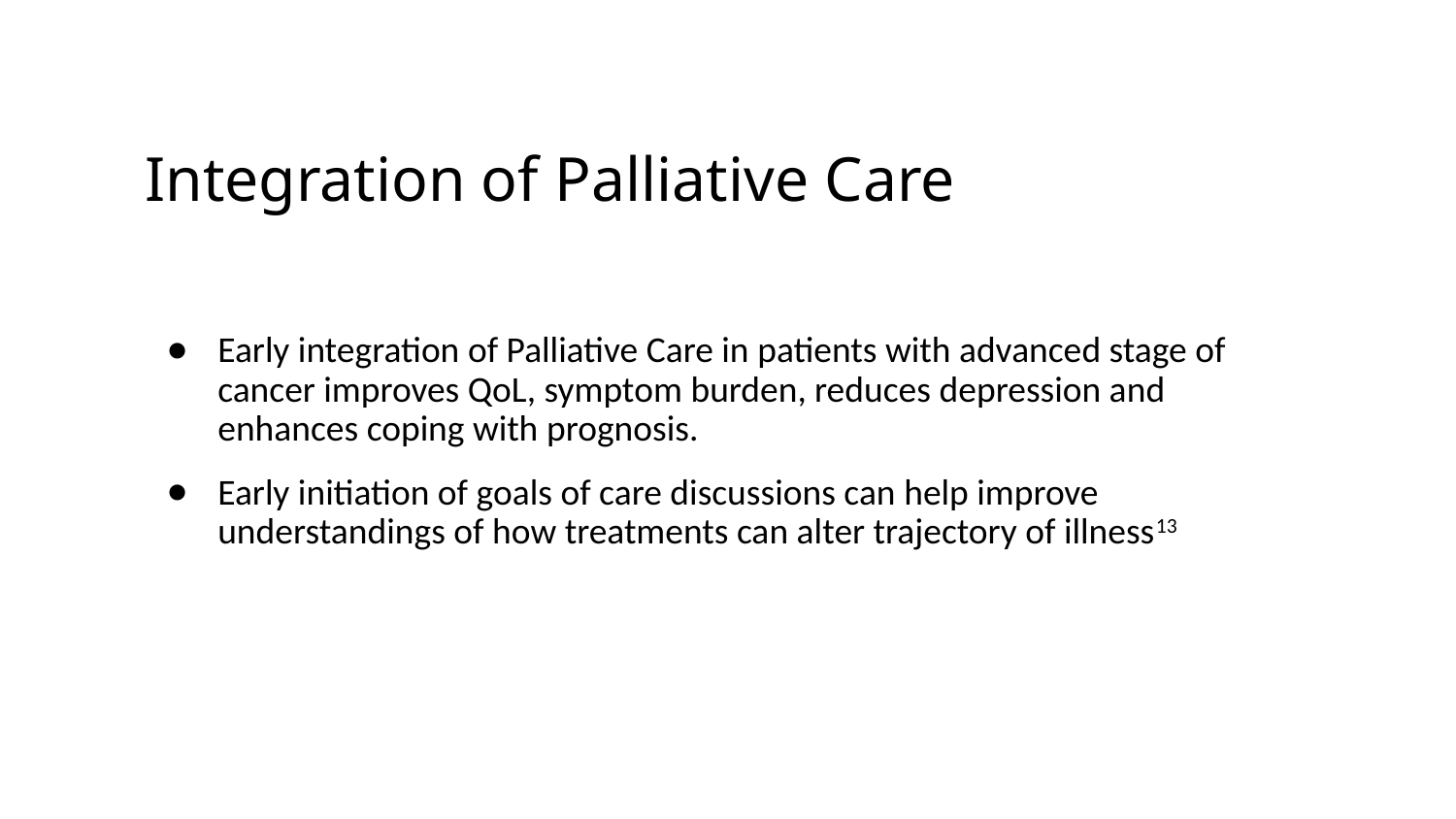

# Integration of Palliative Care
Early integration of Palliative Care in patients with advanced stage of cancer improves QoL, symptom burden, reduces depression and enhances coping with prognosis.
Early initiation of goals of care discussions can help improve understandings of how treatments can alter trajectory of illness13

## Slide 31
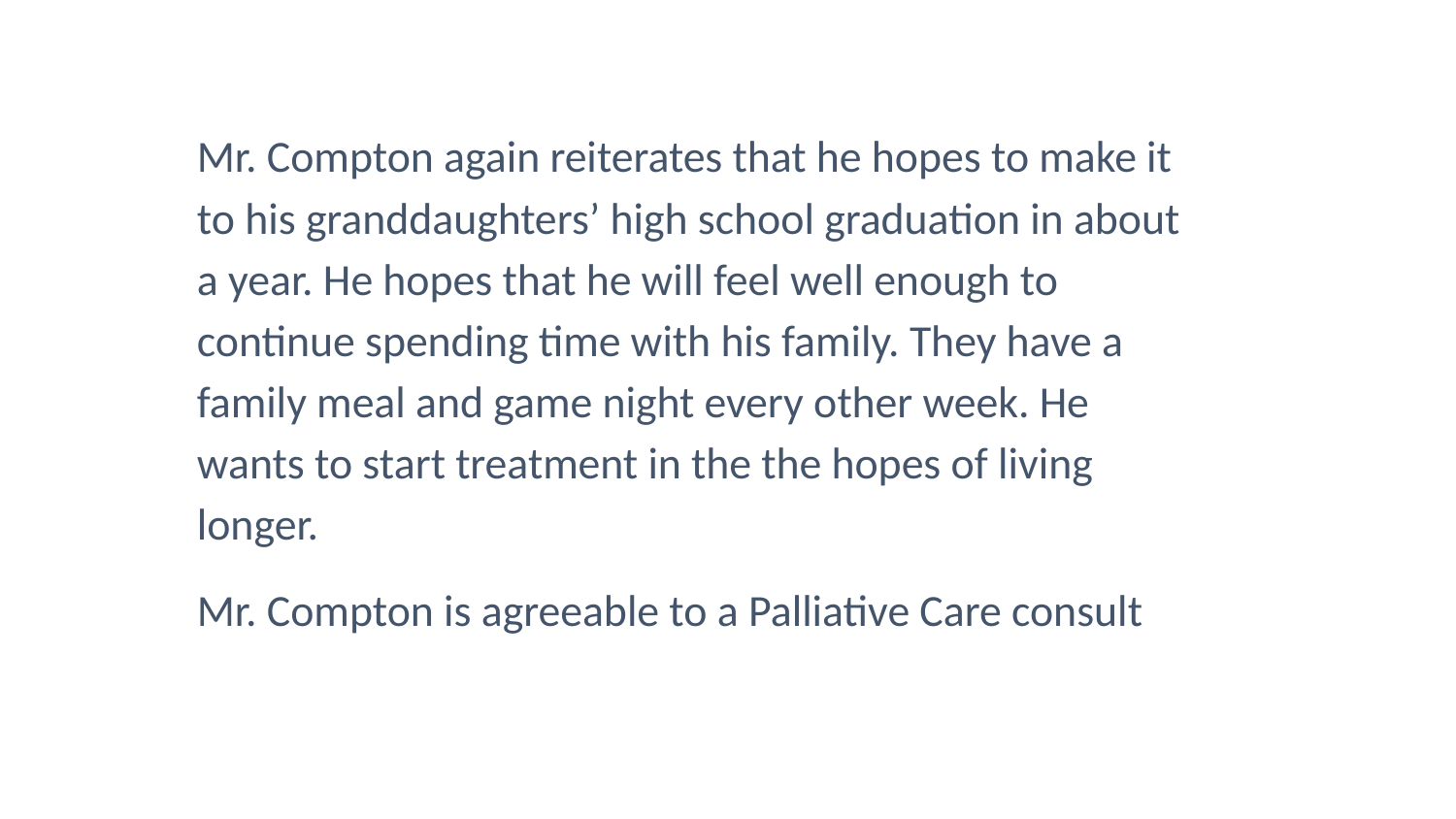

Mr. Compton again reiterates that he hopes to make it to his granddaughters’ high school graduation in about a year. He hopes that he will feel well enough to continue spending time with his family. They have a family meal and game night every other week. He wants to start treatment in the the hopes of living longer.
Mr. Compton is agreeable to a Palliative Care consult

## Slide 32
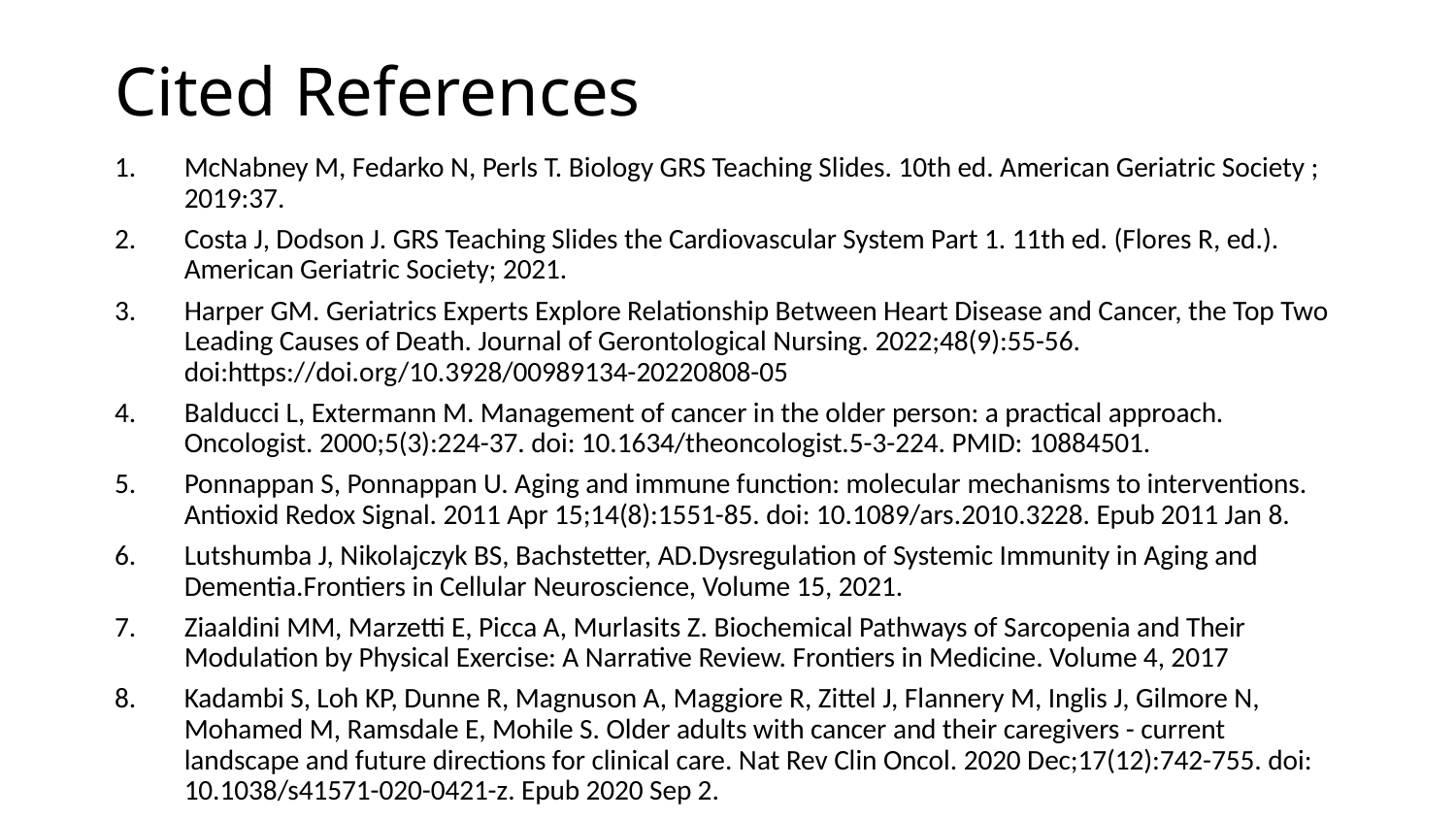

# Cited References
McNabney M, Fedarko N, Perls T. Biology GRS Teaching Slides. 10th ed. American Geriatric Society ; 2019:37.
Costa J, Dodson J. GRS Teaching Slides the Cardiovascular System Part 1. 11th ed. (Flores R, ed.). American Geriatric Society; 2021.
Harper GM. Geriatrics Experts Explore Relationship Between Heart Disease and Cancer, the Top Two Leading Causes of Death. Journal of Gerontological Nursing. 2022;48(9):55-56. doi:https://doi.org/10.3928/00989134-20220808-05
Balducci L, Extermann M. Management of cancer in the older person: a practical approach. Oncologist. 2000;5(3):224-37. doi: 10.1634/theoncologist.5-3-224. PMID: 10884501.
Ponnappan S, Ponnappan U. Aging and immune function: molecular mechanisms to interventions. Antioxid Redox Signal. 2011 Apr 15;14(8):1551-85. doi: 10.1089/ars.2010.3228. Epub 2011 Jan 8.
Lutshumba J, Nikolajczyk BS, Bachstetter, AD.Dysregulation of Systemic Immunity in Aging and Dementia.Frontiers in Cellular Neuroscience, Volume 15, 2021.
Ziaaldini MM, Marzetti E, Picca A, Murlasits Z. Biochemical Pathways of Sarcopenia and Their Modulation by Physical Exercise: A Narrative Review. Frontiers in Medicine. Volume 4, 2017
Kadambi S, Loh KP, Dunne R, Magnuson A, Maggiore R, Zittel J, Flannery M, Inglis J, Gilmore N, Mohamed M, Ramsdale E, Mohile S. Older adults with cancer and their caregivers - current landscape and future directions for clinical care. Nat Rev Clin Oncol. 2020 Dec;17(12):742-755. doi: 10.1038/s41571-020-0421-z. Epub 2020 Sep 2.

## Slide 33
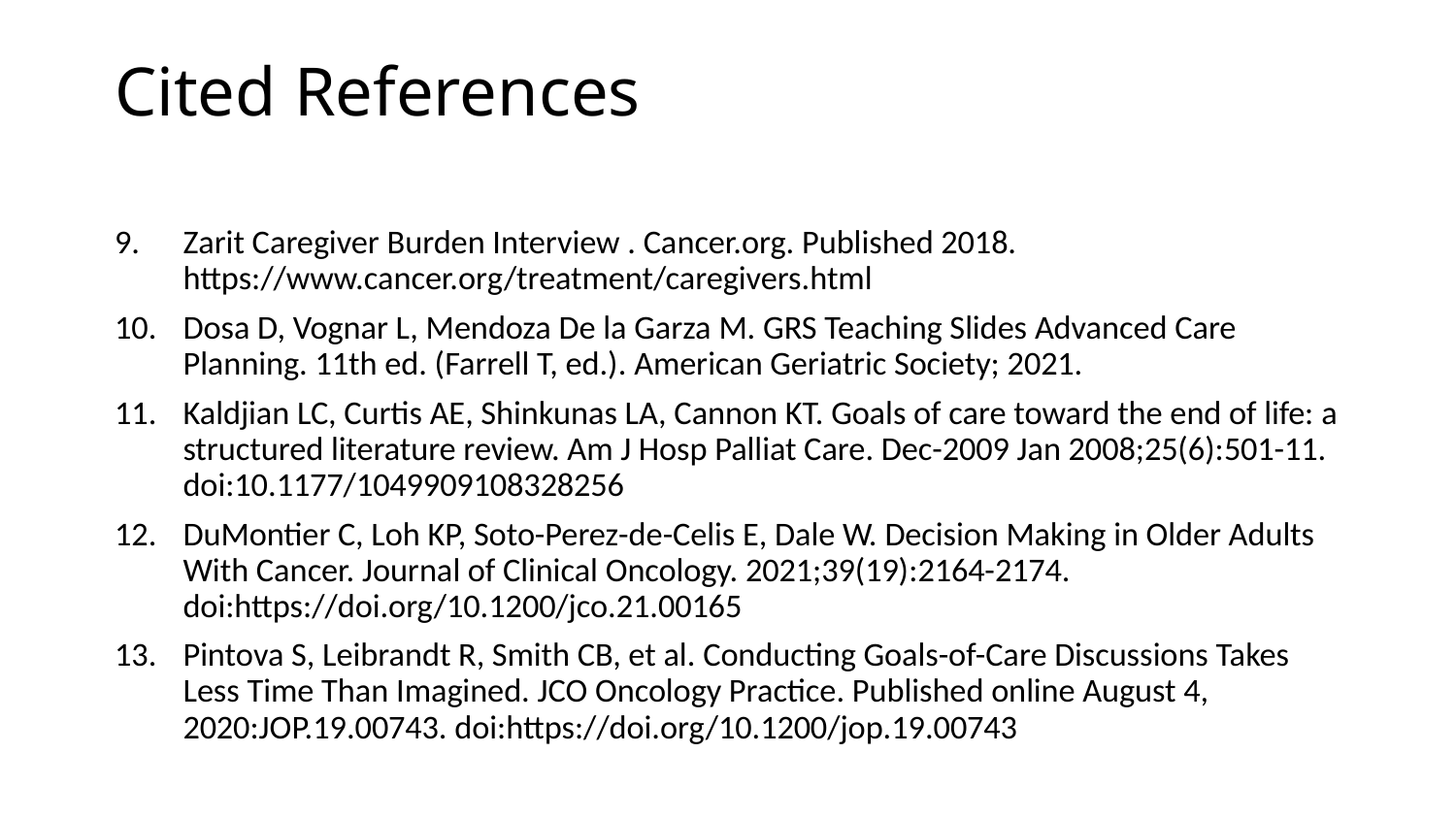

# Cited References
Zarit Caregiver Burden Interview . Cancer.org. Published 2018. https://www.cancer.org/treatment/caregivers.html
Dosa D, Vognar L, Mendoza De la Garza M. GRS Teaching Slides Advanced Care Planning. 11th ed. (Farrell T, ed.). American Geriatric Society; 2021.
Kaldjian LC, Curtis AE, Shinkunas LA, Cannon KT. Goals of care toward the end of life: a structured literature review. Am J Hosp Palliat Care. Dec-2009 Jan 2008;25(6):501-11. doi:10.1177/1049909108328256
DuMontier C, Loh KP, Soto-Perez-de-Celis E, Dale W. Decision Making in Older Adults With Cancer. Journal of Clinical Oncology. 2021;39(19):2164-2174. doi:https://doi.org/10.1200/jco.21.00165
Pintova S, Leibrandt R, Smith CB, et al. Conducting Goals-of-Care Discussions Takes Less Time Than Imagined. JCO Oncology Practice. Published online August 4, 2020:JOP.19.00743. doi:https://doi.org/10.1200/jop.19.00743
