# Supplementary material for: An Introductory Course on Geriatric Oncology
Source: MedEdPORTAL. 2024 Nov 14;20:11471. doi: 10.15766/mep_2374-8265.11471 (PMC11561070; doi:10.15766/mep_2374-8265.11471)
Supplement: Supplementary file 1 — Introduction to Geriatric Oncology.pptxThe Comprehensive Geriatric Assessment.pptxGeriatric Screening Tools.pptxBiology of Aging.pptxCancer Therapy in the Older Adult.pptxSummary of Interactive Sessions.docxSession 5 Patient Case 1.docxSession 5 Patient Case 2.docxSession 5 Patient Case 3.docxGeriatric Oncology Knowledge Assessment.docxKnowledge Assessment Answer Key.docxSelf-Perceived Competency Assessment.docxCurriculum Session Assessment.docx [file mep_2374-8265.11471-s001.zip › E. Cancer Therapy in the Older Adult.pptx]

## Slide 1
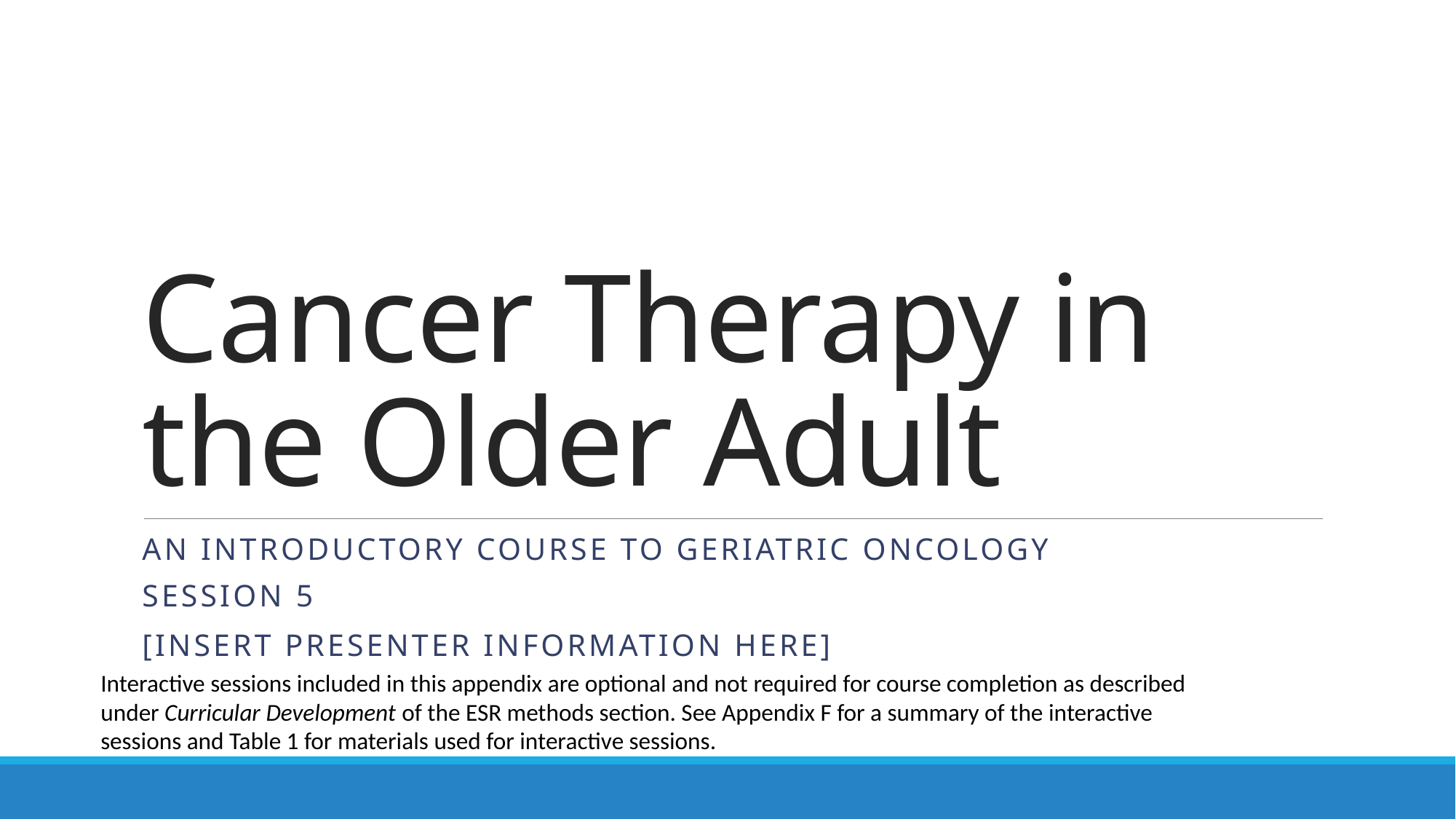

# Cancer Therapy in the Older Adult
An Introductory Course to Geriatric Oncology
Session 5
[Insert presenter information here]
Interactive sessions included in this appendix are optional and not required for course completion as described under Curricular Development of the ESR methods section. See Appendix F for a summary of the interactive sessions and Table 1 for materials used for interactive sessions.

## Slide 2
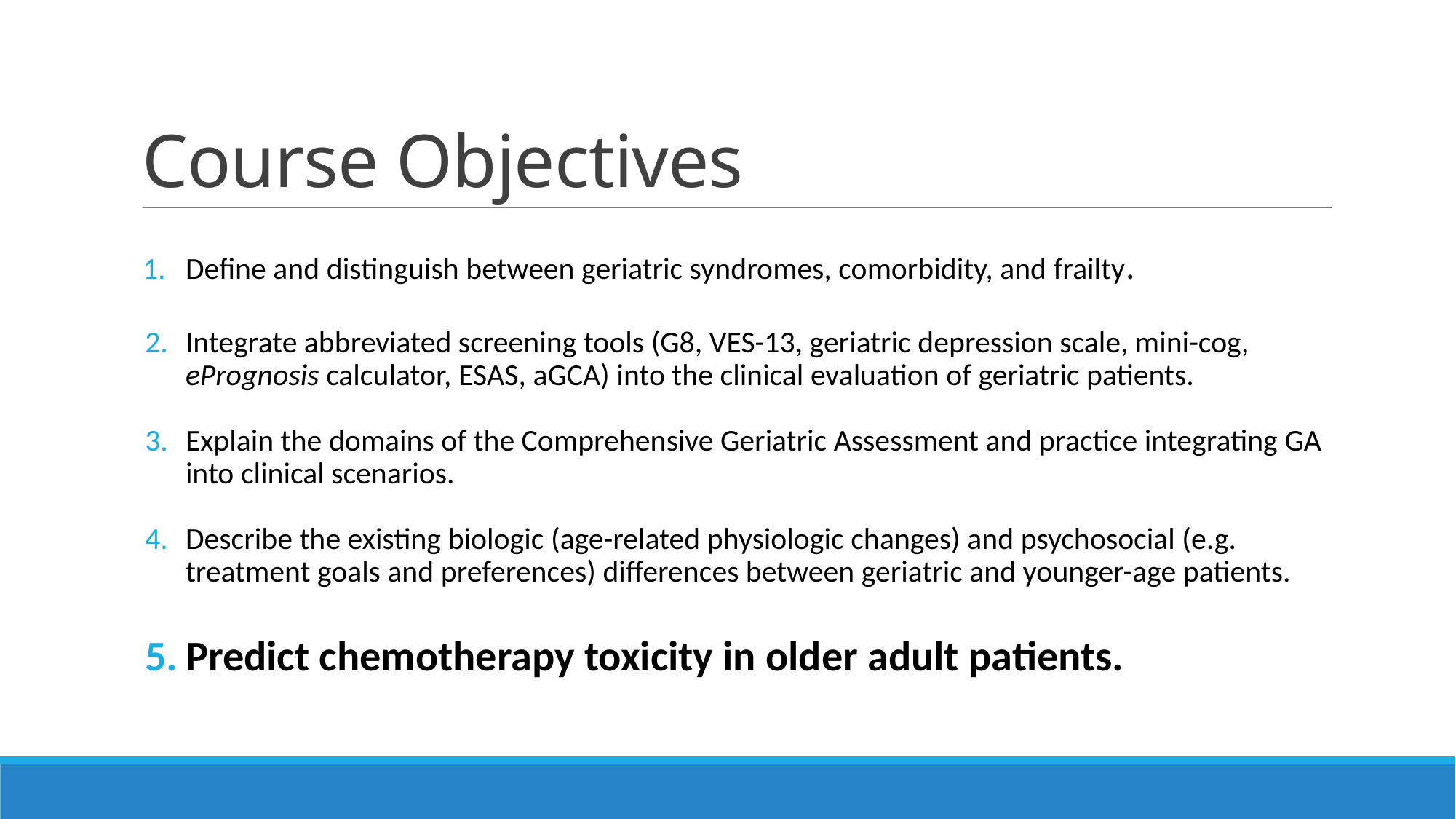

# Course Objectives
Define and distinguish between geriatric syndromes, comorbidity, and frailty. ​
Integrate abbreviated screening tools (G8, VES-13, geriatric depression scale, mini-cog, ePrognosis calculator, ESAS, aGCA) into the clinical evaluation of geriatric patients.
Explain the domains of the Comprehensive Geriatric Assessment and practice integrating GA into clinical scenarios. ​
Describe the existing biologic (age-related physiologic changes) and psychosocial (e.g. treatment goals and preferences) differences between geriatric and younger-age patients. ​​
Predict chemotherapy toxicity in older adult patients.​

## Slide 3
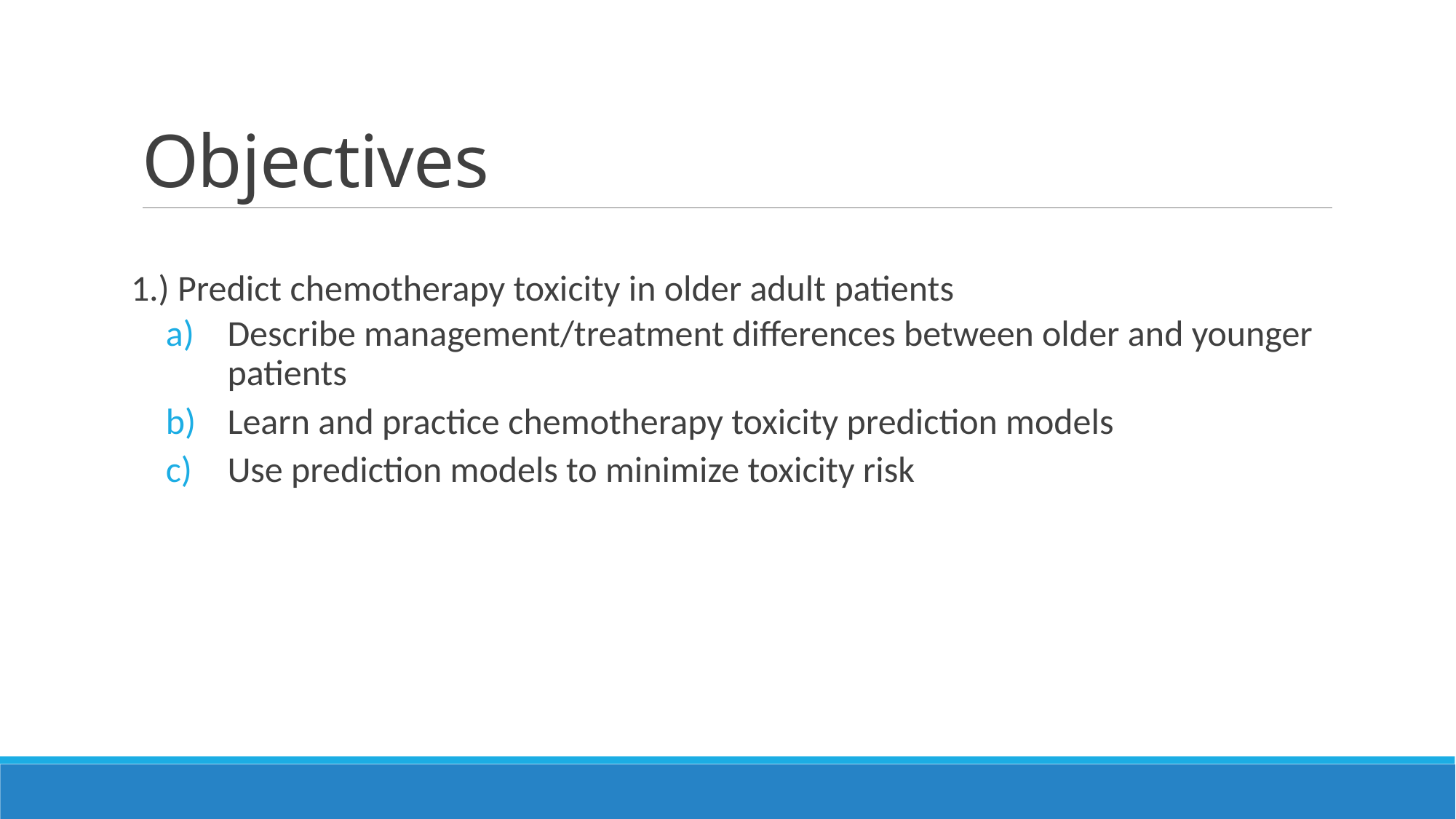

# Objectives
1.) Predict chemotherapy toxicity in older adult patients
Describe management/treatment differences between older and younger patients
Learn and practice chemotherapy toxicity prediction models
Use prediction models to minimize toxicity risk

## Slide 4
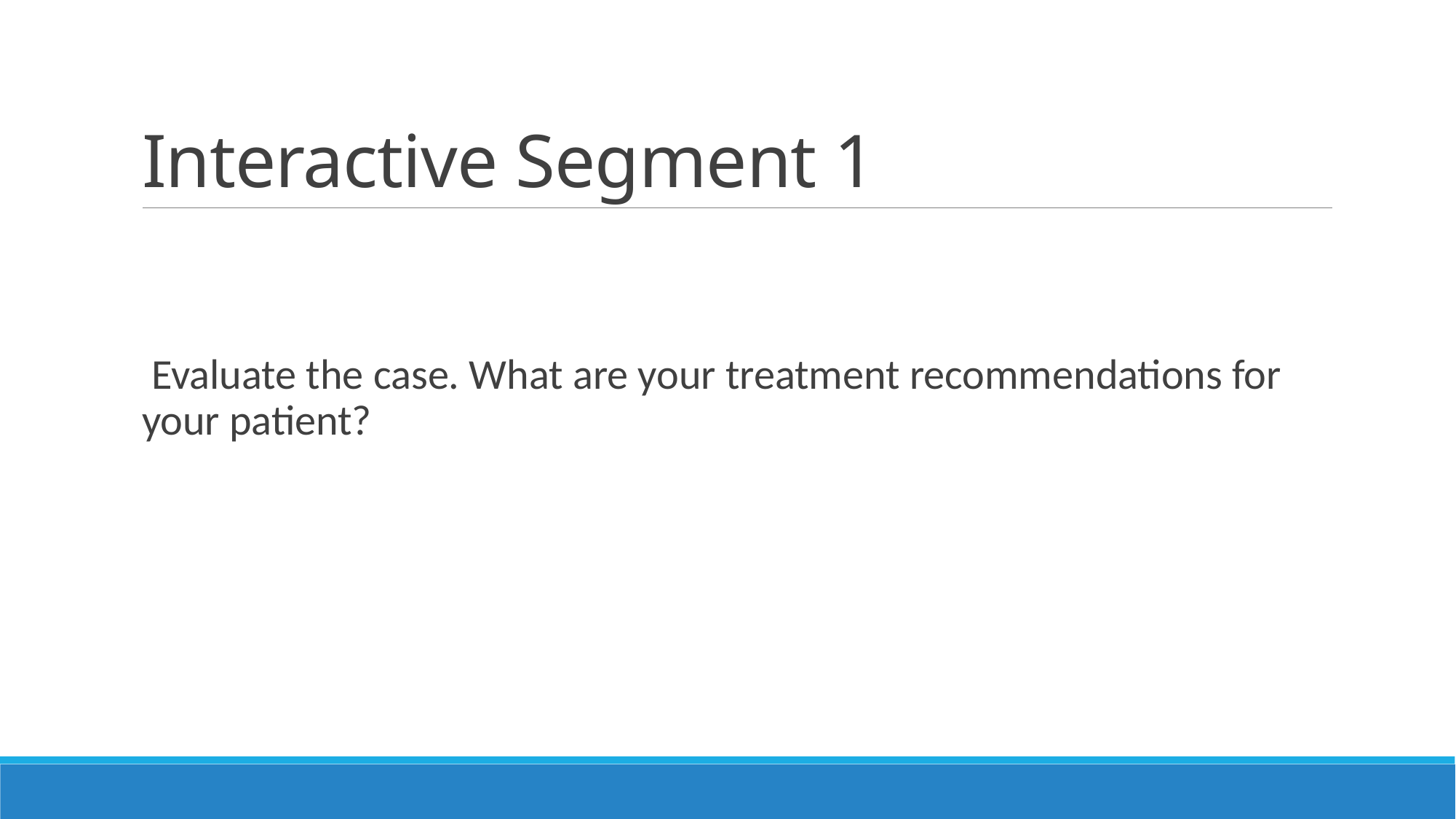

# Interactive Segment 1
 Evaluate the case. What are your treatment recommendations for your patient?

## Slide 5
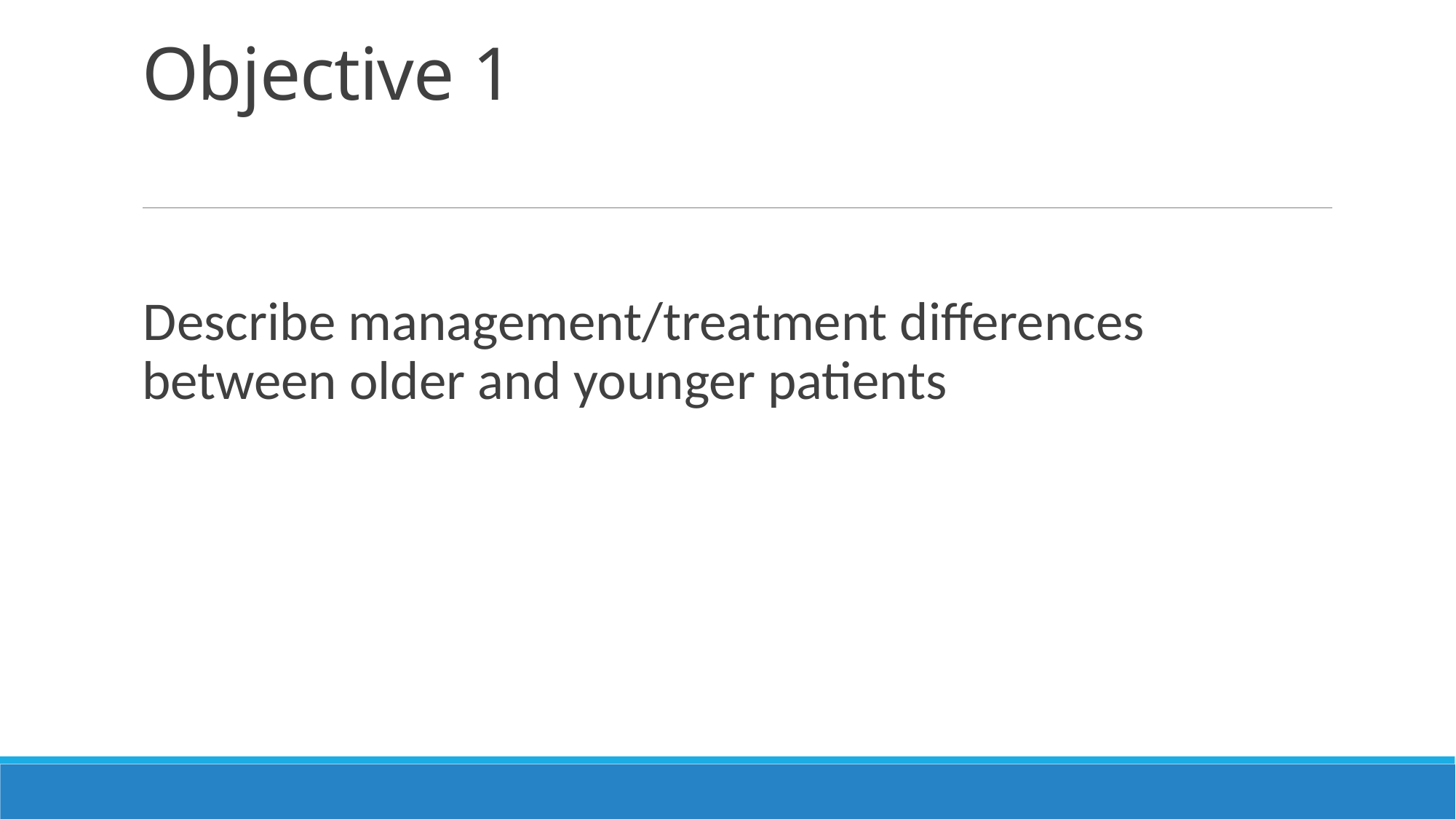

# Objective 1
Describe management/treatment differences between older and younger patients

## Slide 6
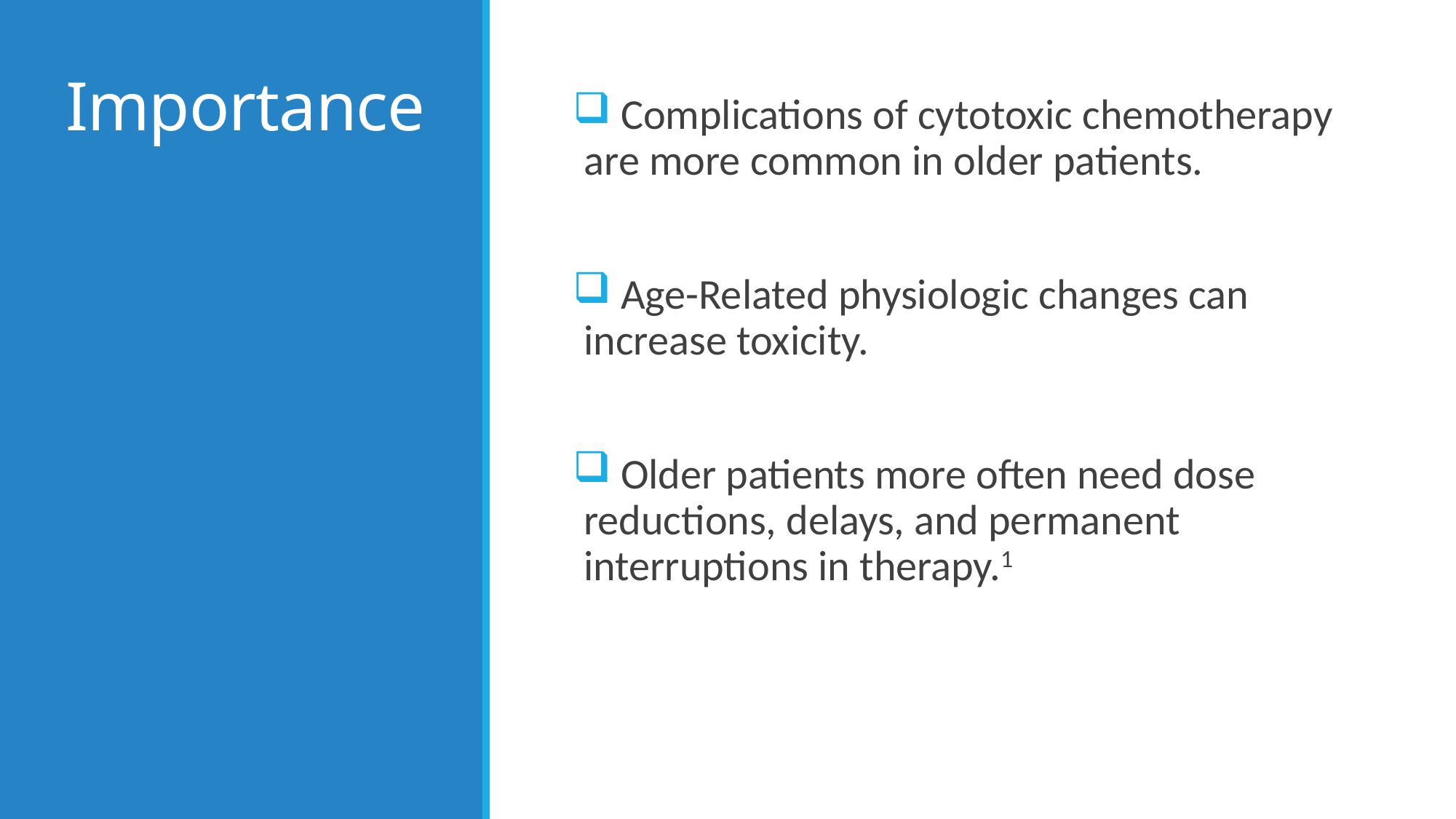

# Importance
 Complications of cytotoxic chemotherapy are more common in older patients.
 Age-Related physiologic changes can increase toxicity.
 Older patients more often need dose reductions, delays, and permanent interruptions in therapy.1

## Slide 7
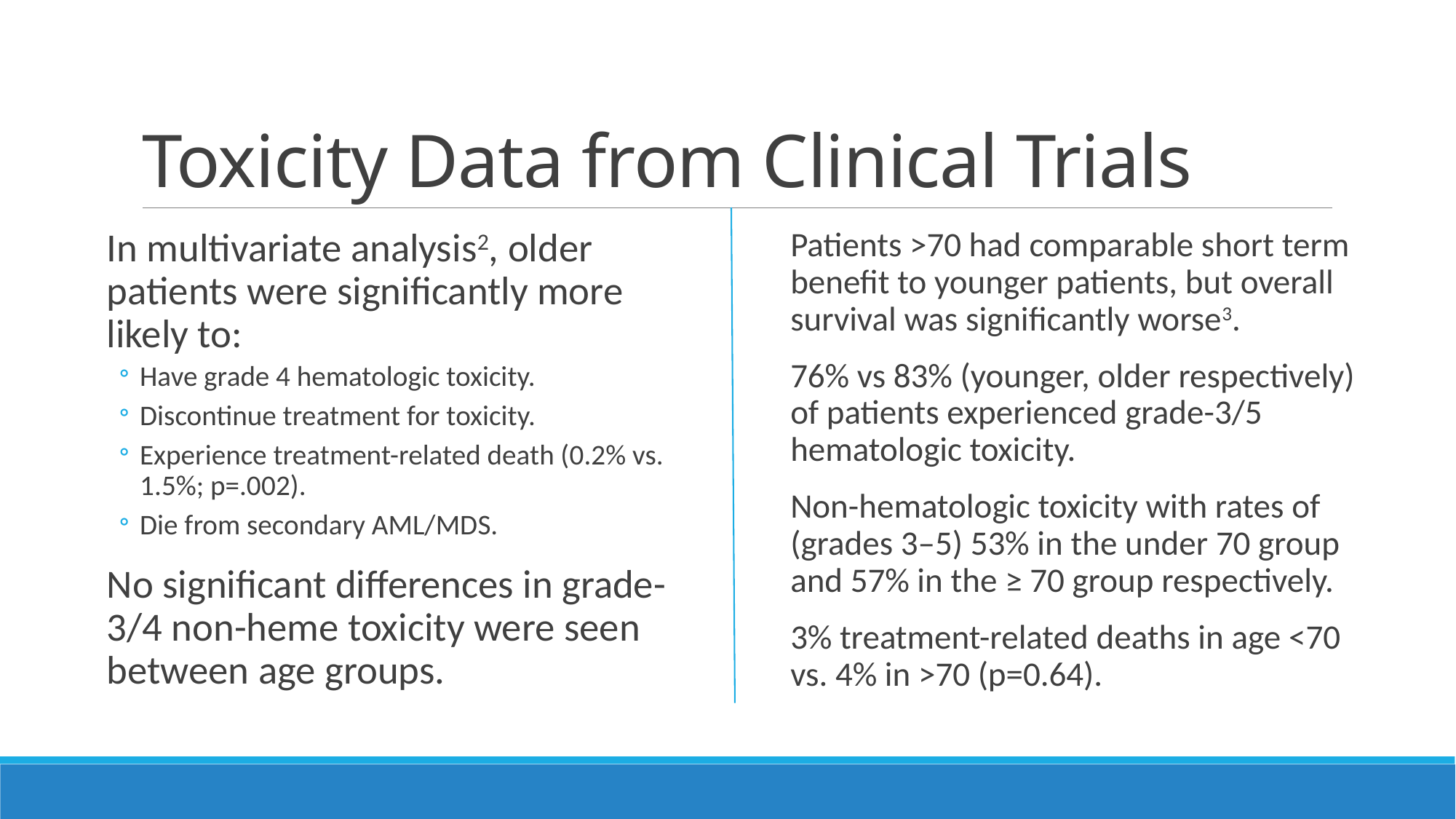

# Toxicity Data from Clinical Trials
Patients >70 had comparable short term benefit to younger patients, but overall survival was significantly worse3.
76% vs 83% (younger, older respectively) of patients experienced grade-3/5 hematologic toxicity.
Non-hematologic toxicity with rates of (grades 3–5) 53% in the under 70 group and 57% in the ≥ 70 group respectively.
3% treatment-related deaths in age <70 vs. 4% in >70 (p=0.64).
In multivariate analysis2, older patients were significantly more likely to:
Have grade 4 hematologic toxicity.
Discontinue treatment for toxicity.
Experience treatment-related death (0.2% vs. 1.5%; p=.002).
Die from secondary AML/MDS.
No significant differences in grade-3/4 non-heme toxicity were seen between age groups.

## Slide 8
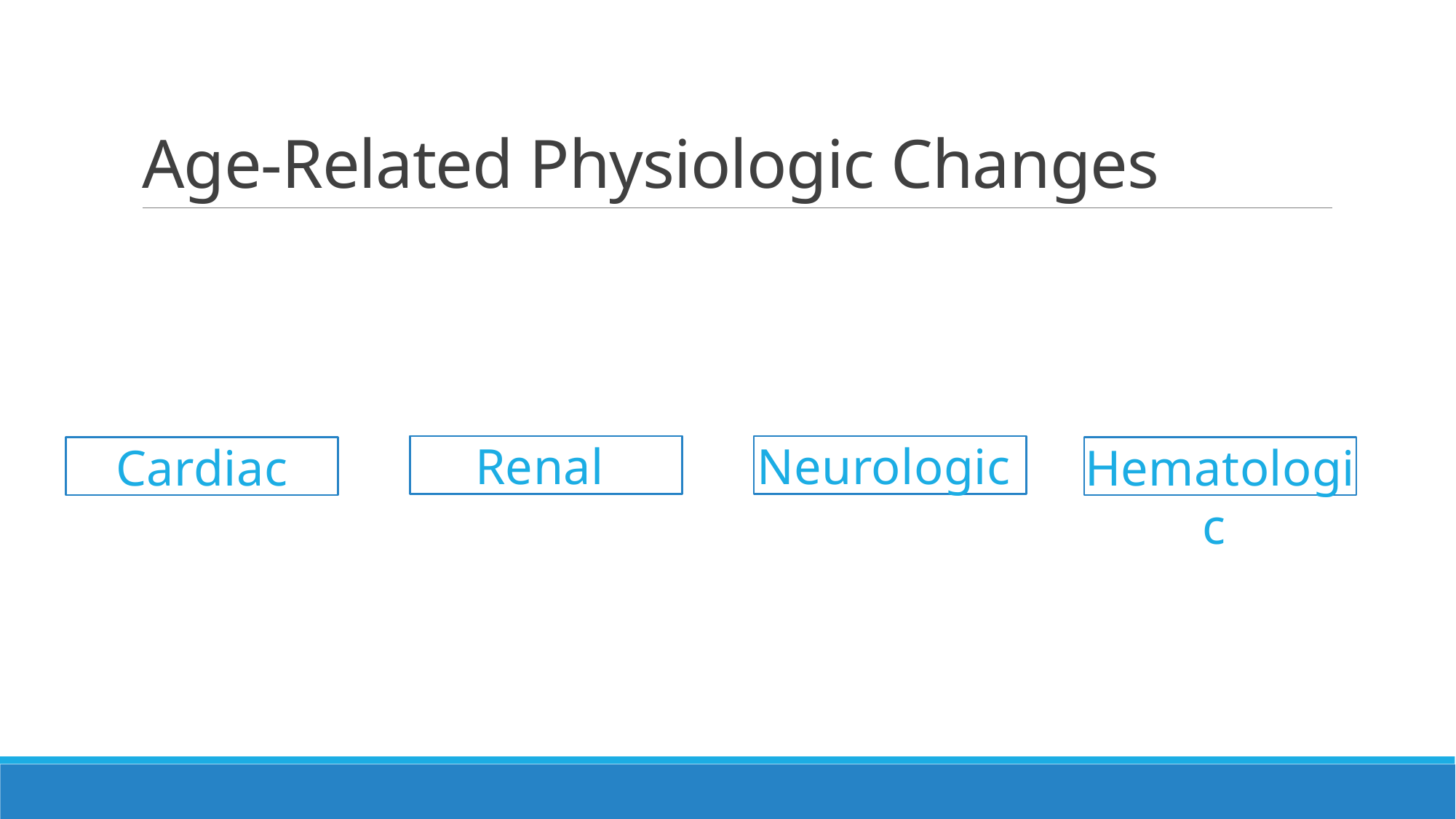

# Age-Related Physiologic Changes
Renal
Neurologic
Cardiac
Hematologic

## Slide 9
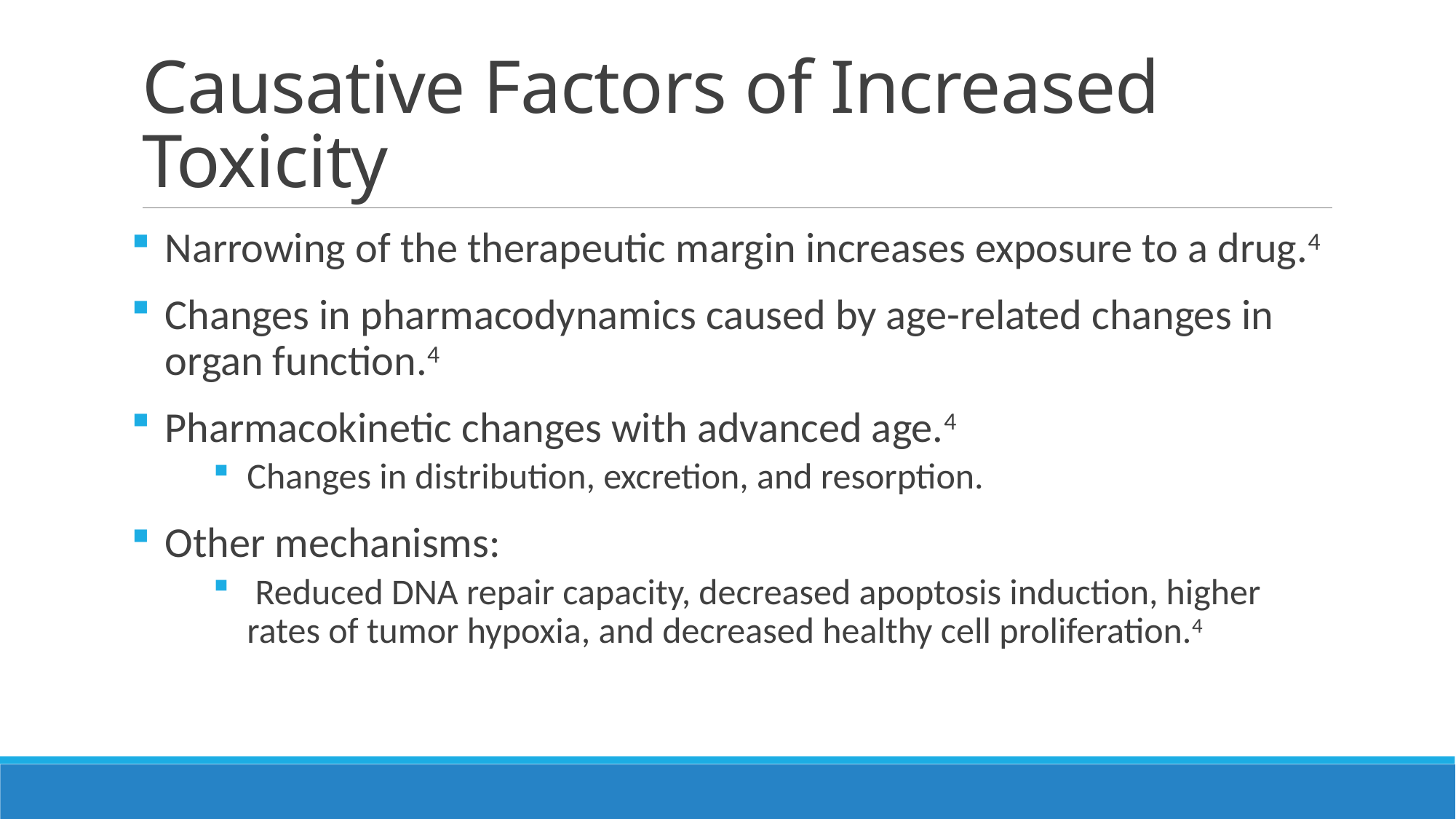

# Causative Factors of Increased Toxicity
Narrowing of the therapeutic margin increases exposure to a drug.4
Changes in pharmacodynamics caused by age-related changes in organ function.4
Pharmacokinetic changes with advanced age.4
Changes in distribution, excretion, and resorption.
Other mechanisms:
 Reduced DNA repair capacity, decreased apoptosis induction, higher rates of tumor hypoxia, and decreased healthy cell proliferation.4

## Slide 10
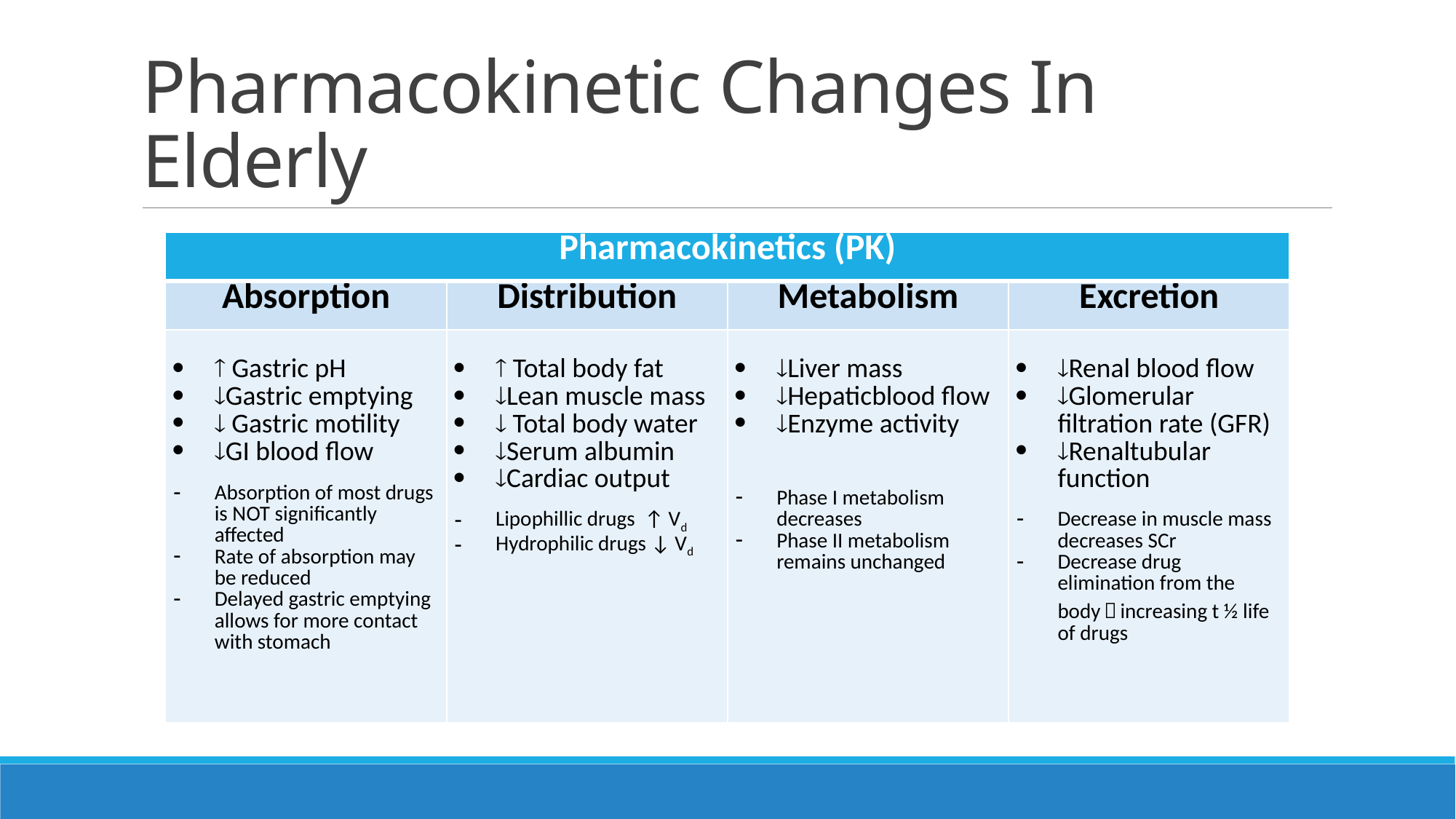

# Pharmacokinetic Changes In Elderly
| Pharmacokinetics (PK) | | | |
| --- | --- | --- | --- |
| Absorption | Distribution | Metabolism | Excretion |
|  Gastric pH Gastric emptying  Gastric motility GI blood flow   Absorption of most drugs is NOT significantly affected Rate of absorption may be reduced Delayed gastric emptying allows for more contact with stomach |  Total body fat Lean muscle mass  Total body water Serum albumin Cardiac output   Lipophillic drugs ↑ Vd Hydrophilic drugs ↓ Vd | Liver mass Hepaticblood flow Enzyme activity       Phase I metabolism decreases Phase II metabolism remains unchanged | Renal blood flow Glomerular filtration rate (GFR) Renaltubular function   Decrease in muscle mass decreases SCr Decrease drug elimination from the body  increasing t ½ life of drugs |

## Slide 11
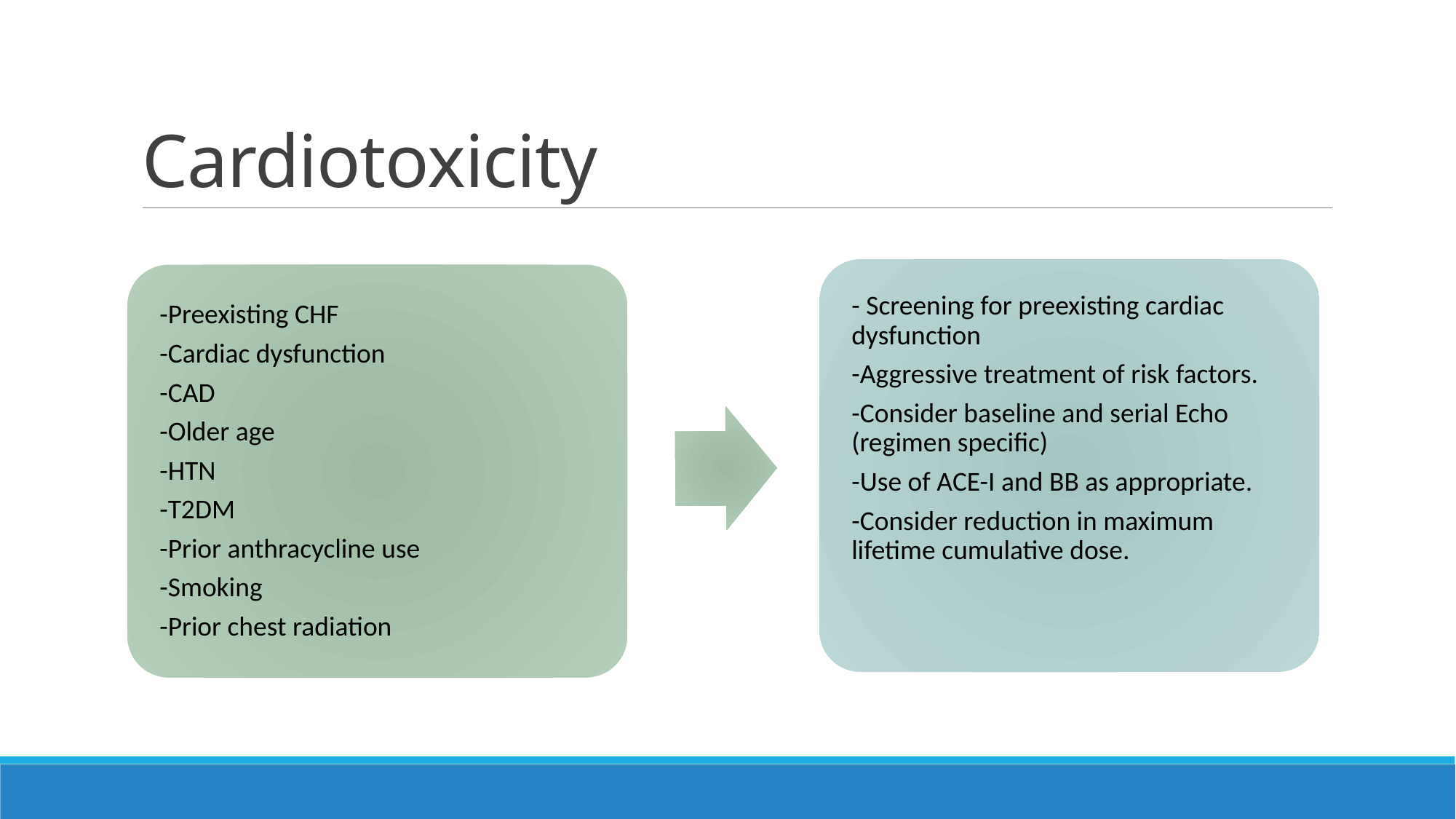

# Cardiotoxicity

## Slide 12
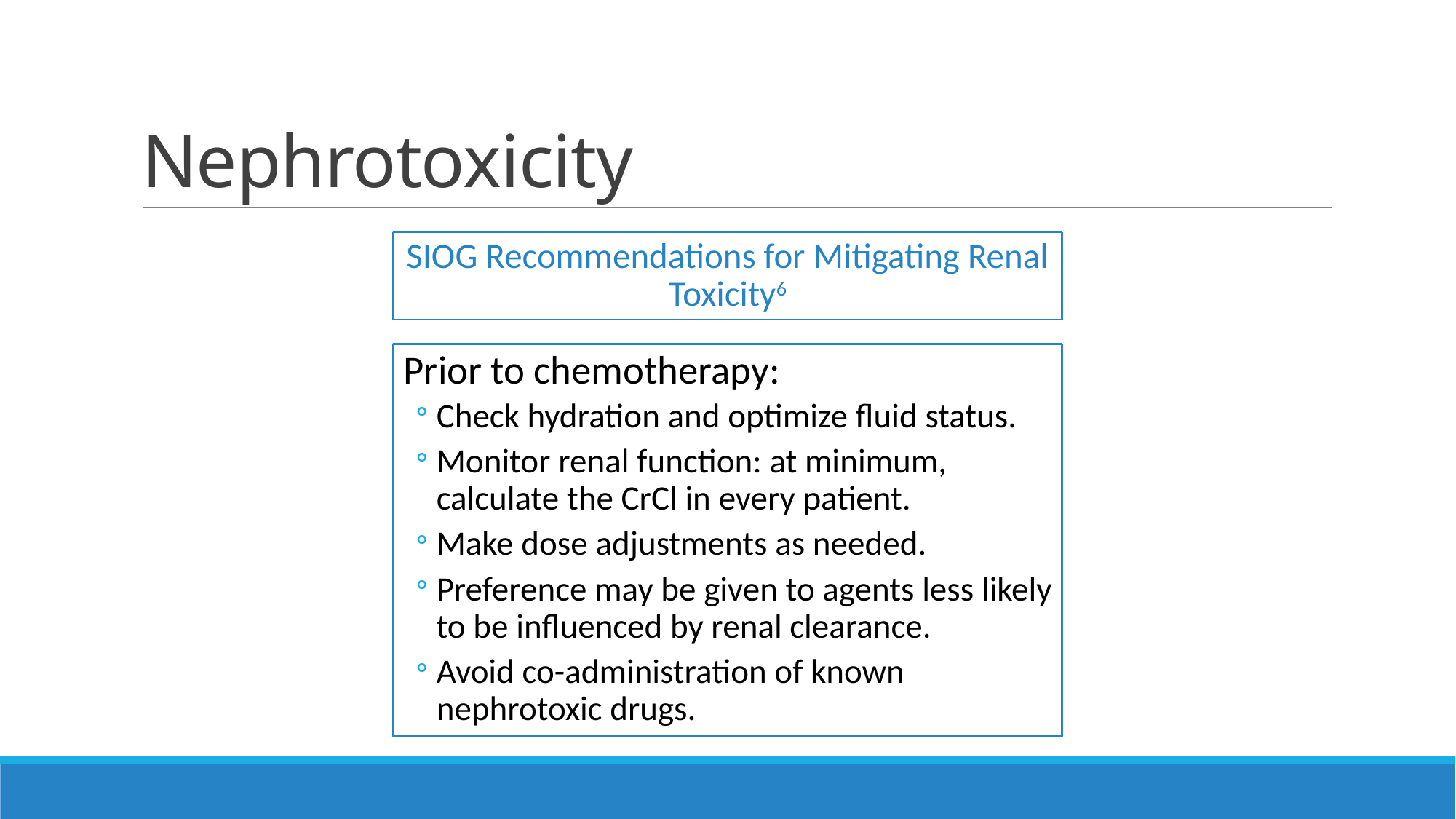

# Nephrotoxicity
SIOG Recommendations for Mitigating Renal Toxicity6
Prior to chemotherapy:
Check hydration and optimize fluid status.
Monitor renal function: at minimum, calculate the CrCl in every patient.
Make dose adjustments as needed.
Preference may be given to agents less likely to be influenced by renal clearance.
Avoid co-administration of known nephrotoxic drugs.

## Slide 13
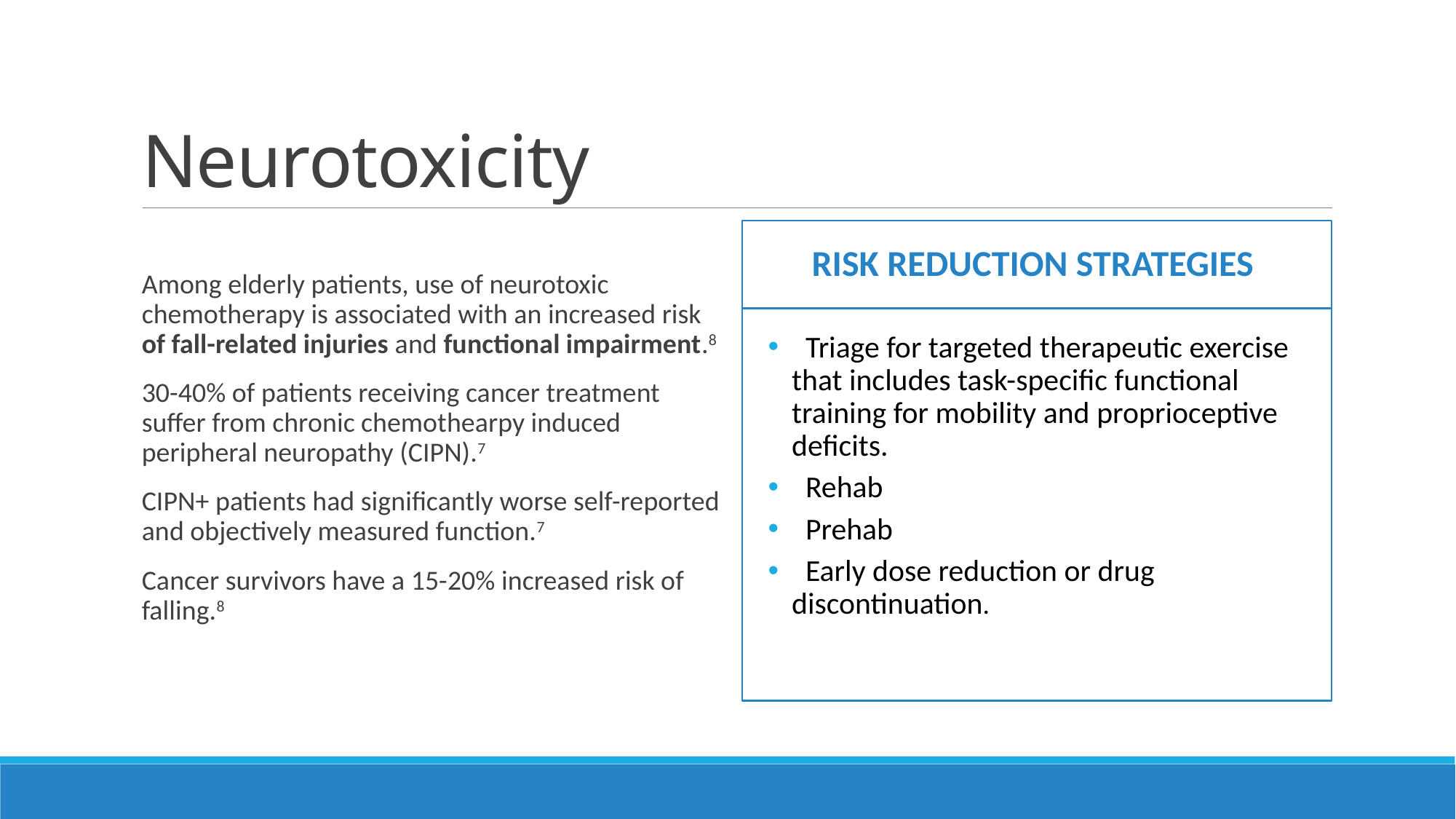

# Neurotoxicity
Risk reduction strategies
Among elderly patients, use of neurotoxic chemotherapy is associated with an increased risk of fall-related injuries and functional impairment.8
30-40% of patients receiving cancer treatment suffer from chronic chemothearpy induced peripheral neuropathy (CIPN).7
CIPN+ patients had significantly worse self-reported and objectively measured function.7
Cancer survivors have a 15-20% increased risk of falling.8
 Triage for targeted therapeutic exercise that includes task-specific functional training for mobility and proprioceptive deficits.
 Rehab
 Prehab
 Early dose reduction or drug discontinuation.

## Slide 14
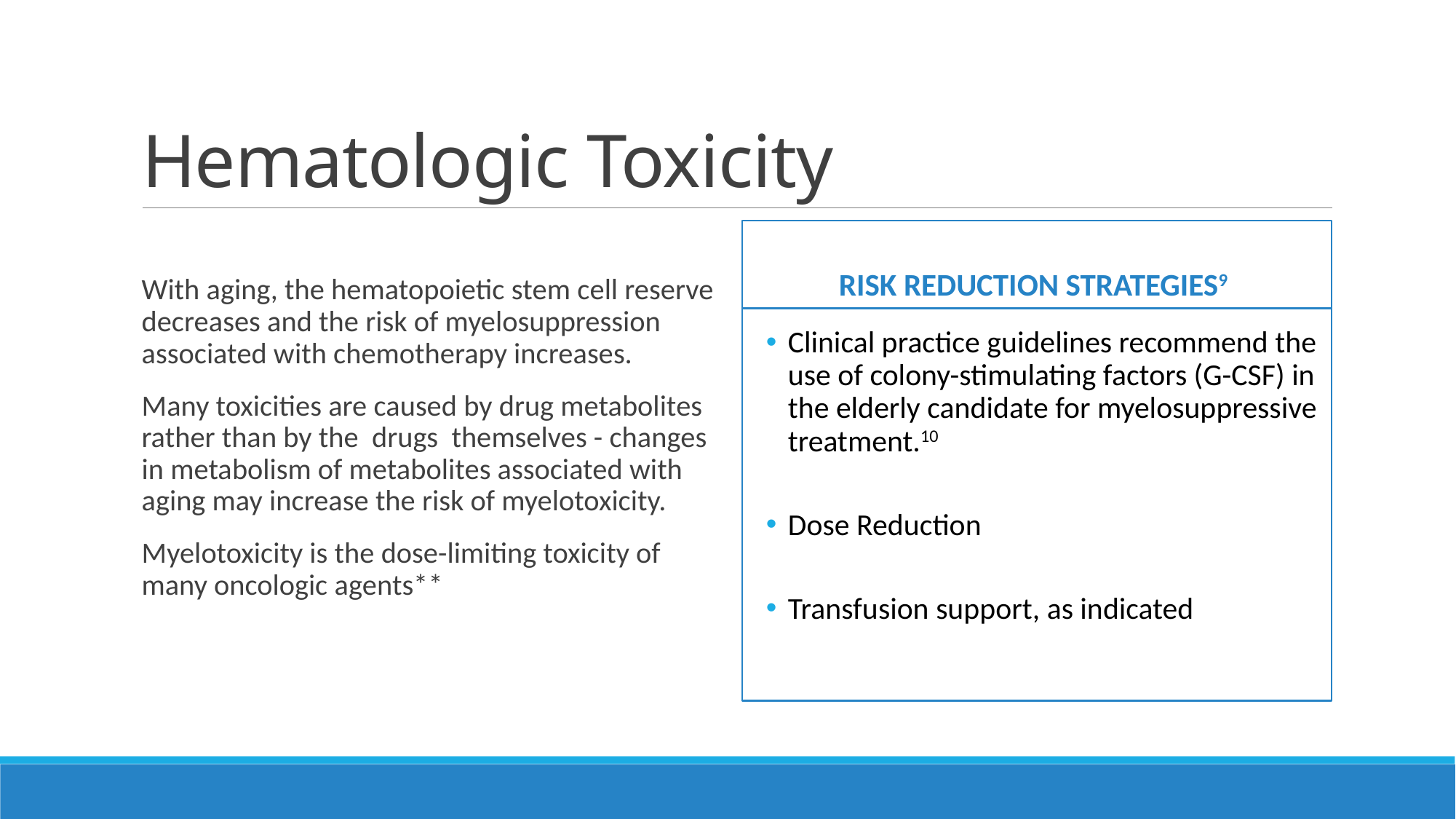

# Hematologic Toxicity
Risk reduction strategies9
With aging, the hematopoietic stem cell reserve decreases and the risk of myelosuppression associated with chemotherapy increases.
Many toxicities are caused by drug metabolites rather than by the drugs themselves - changes in metabolism of metabolites associated with aging may increase the risk of myelotoxicity.
Myelotoxicity is the dose-limiting toxicity of many oncologic agents**
Clinical practice guidelines recommend the use of colony-stimulating factors (G-CSF) in the elderly candidate for myelosuppressive treatment.10
Dose Reduction
Transfusion support, as indicated

## Slide 15
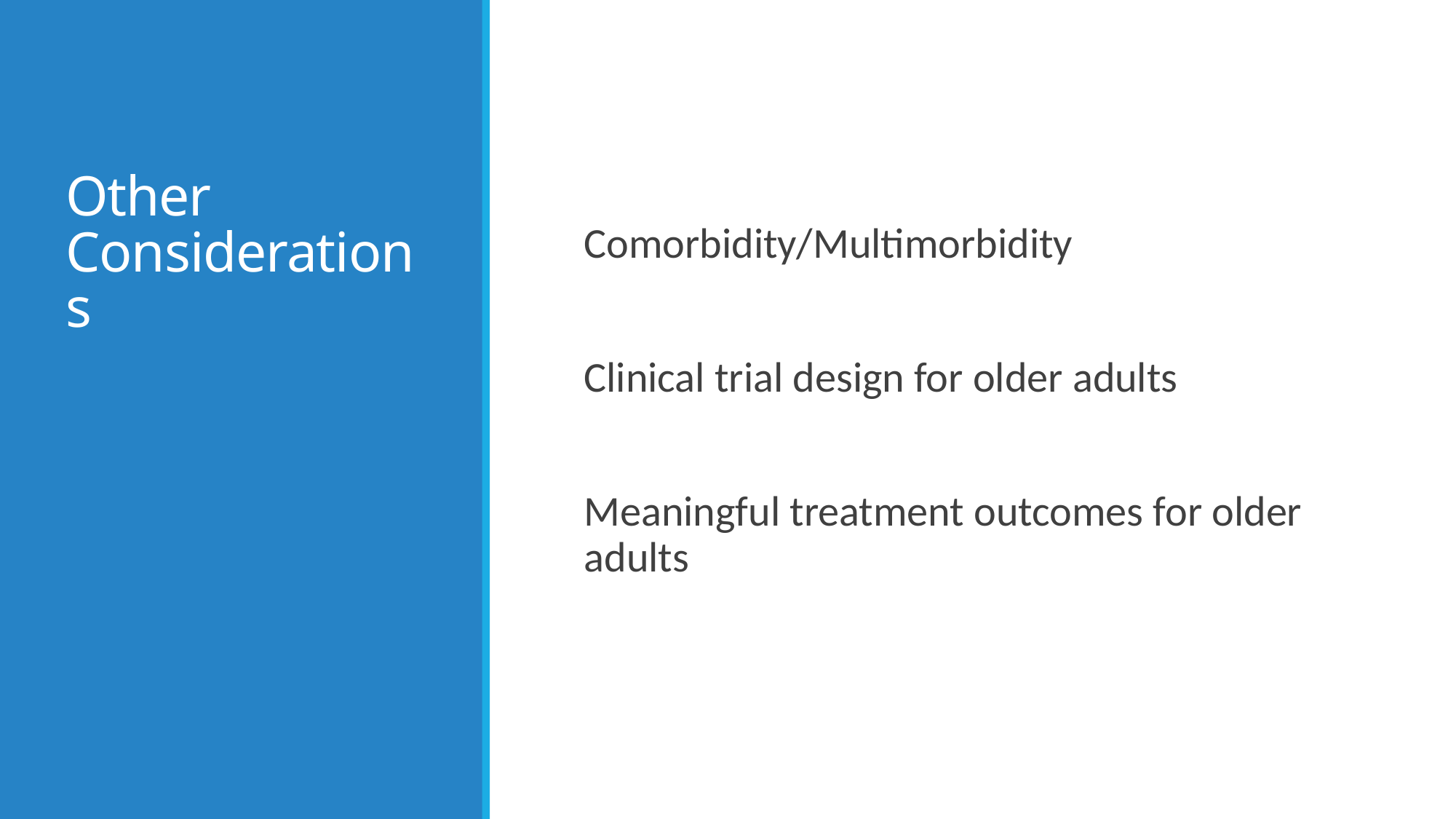

# Other Considerations
Comorbidity/Multimorbidity
Clinical trial design for older adults
Meaningful treatment outcomes for older adults

## Slide 16
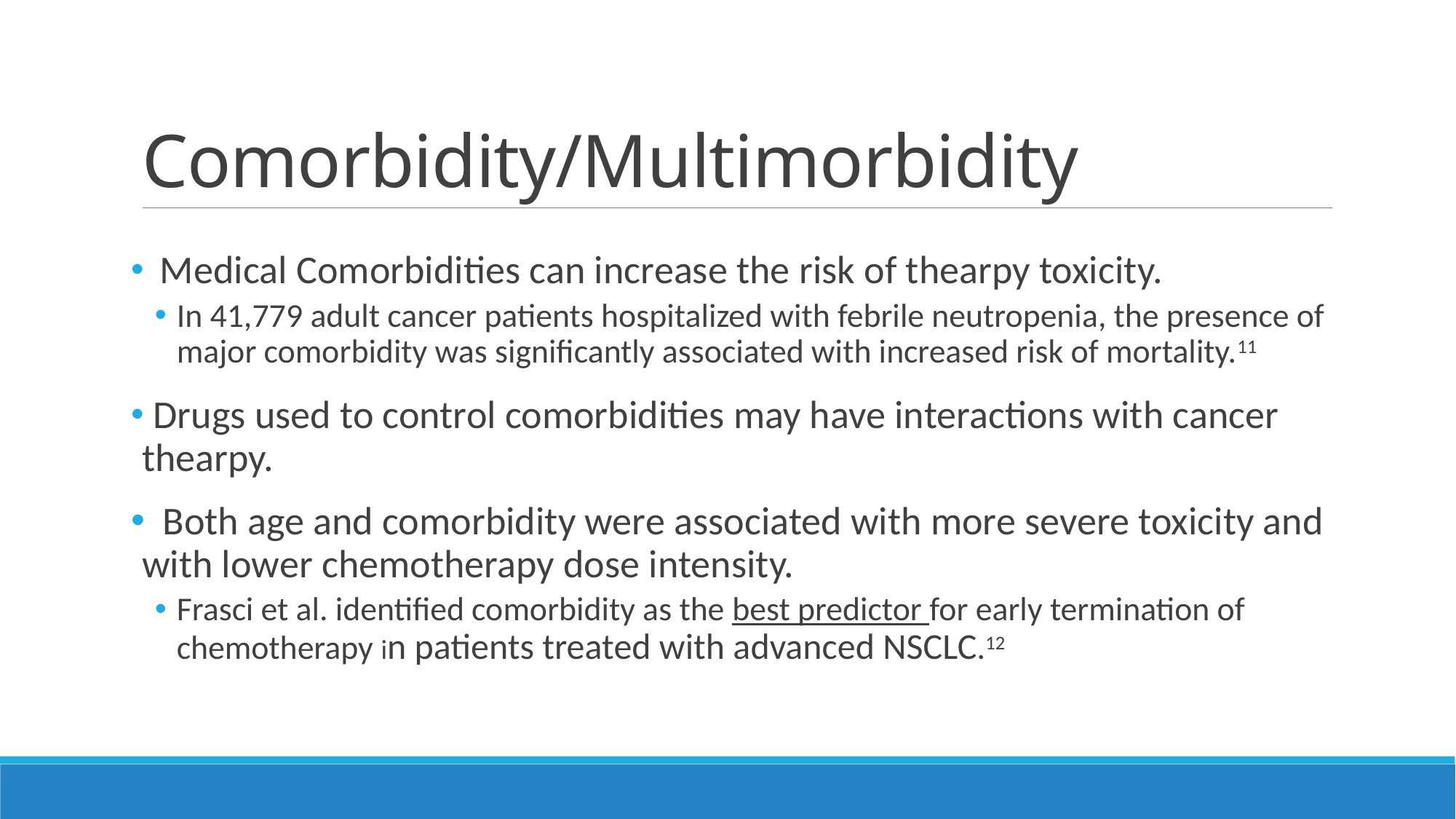

# Comorbidity/Multimorbidity
 Medical Comorbidities can increase the risk of thearpy toxicity.
In 41,779 adult cancer patients hospitalized with febrile neutropenia, the presence of major comorbidity was significantly associated with increased risk of mortality.11
 Drugs used to control comorbidities may have interactions with cancer thearpy.
 Both age and comorbidity were associated with more severe toxicity and with lower chemotherapy dose intensity.
Frasci et al. identified comorbidity as the best predictor for early termination of chemotherapy in patients treated with advanced NSCLC.12

## Slide 17
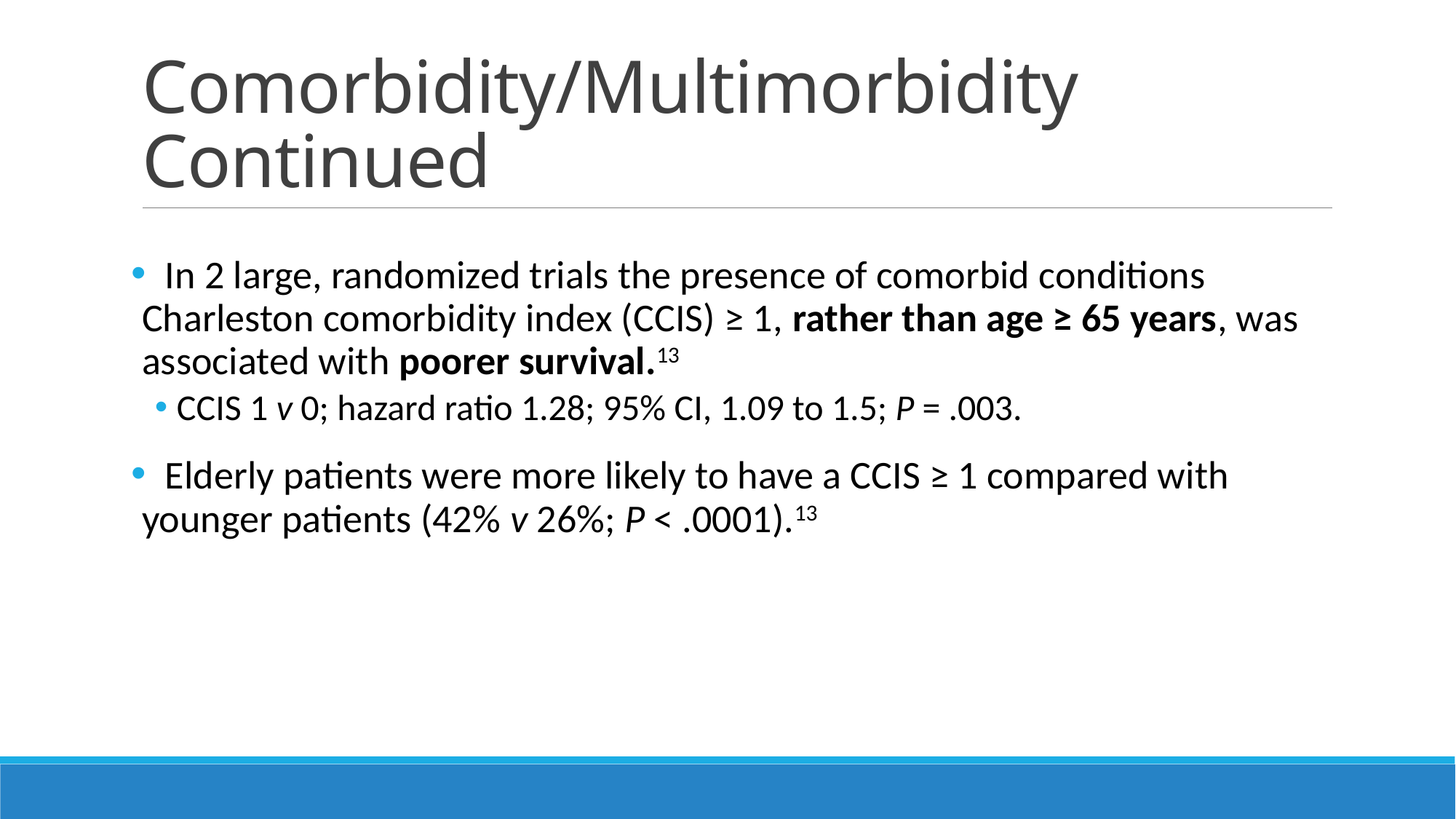

# Comorbidity/Multimorbidity Continued
 In 2 large, randomized trials the presence of comorbid conditions Charleston comorbidity index (CCIS) ≥ 1, rather than age ≥ 65 years, was associated with poorer survival.13
CCIS 1 v 0; hazard ratio 1.28; 95% CI, 1.09 to 1.5; P = .003.
 Elderly patients were more likely to have a CCIS ≥ 1 compared with younger patients (42% v 26%; P < .0001).13

## Slide 18
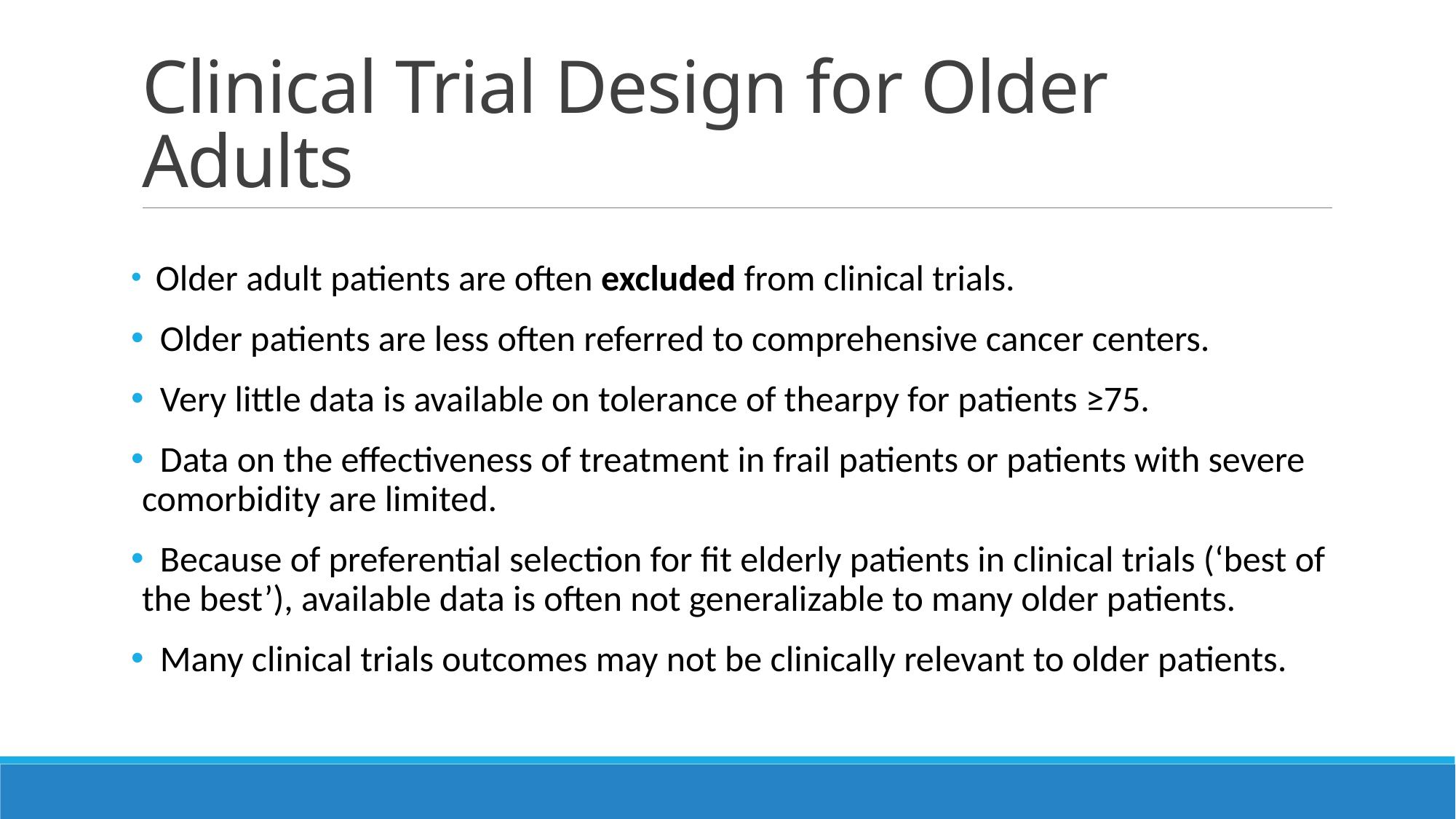

# Clinical Trial Design for Older Adults
 Older adult patients are often excluded from clinical trials.
 Older patients are less often referred to comprehensive cancer centers.
 Very little data is available on tolerance of thearpy for patients ≥75.
 Data on the effectiveness of treatment in frail patients or patients with severe comorbidity are limited.
 Because of preferential selection for fit elderly patients in clinical trials (‘best of the best’), available data is often not generalizable to many older patients.
 Many clinical trials outcomes may not be clinically relevant to older patients.

## Slide 19
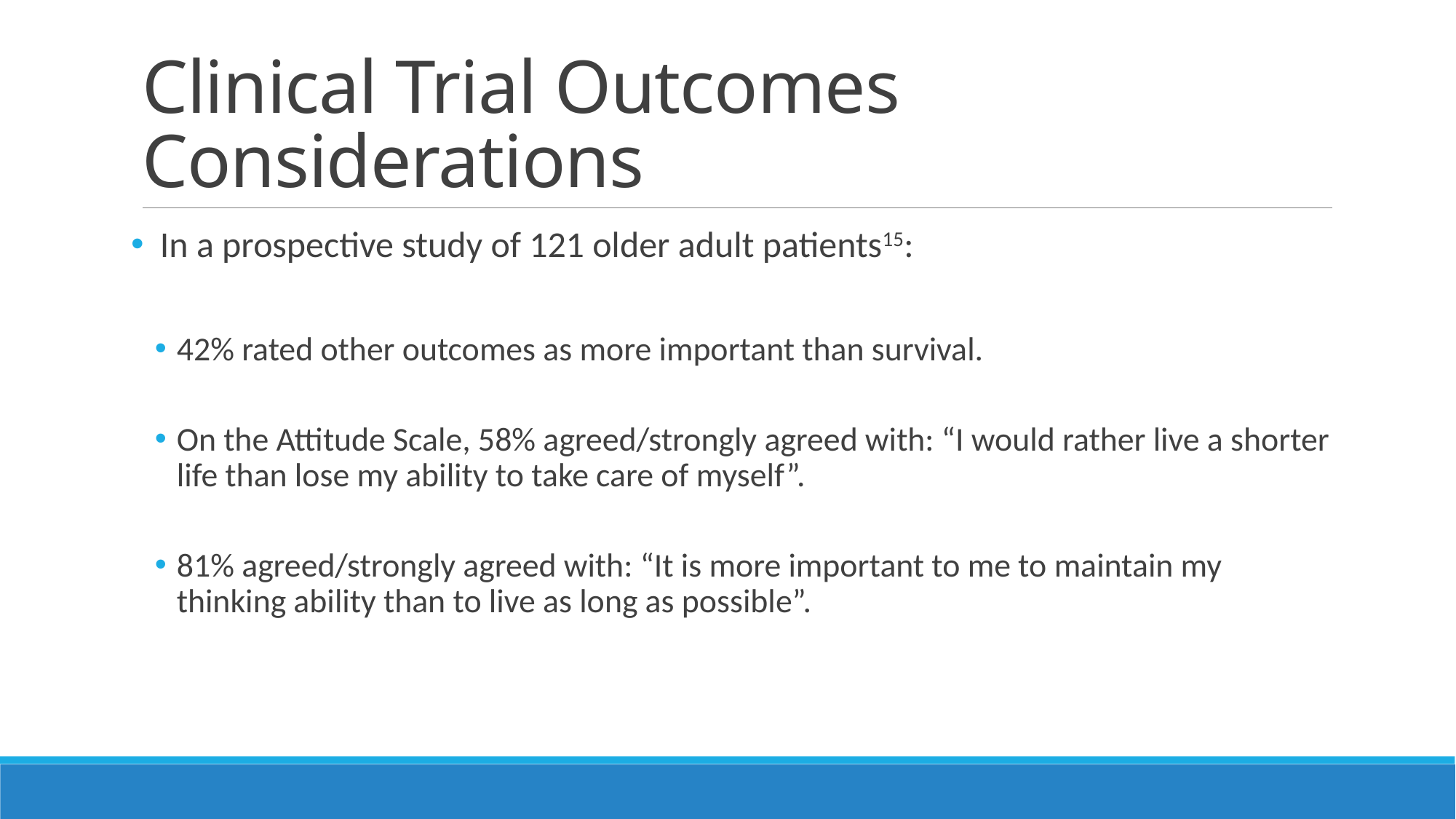

# Clinical Trial Outcomes Considerations
 In a prospective study of 121 older adult patients15:
42% rated other outcomes as more important than survival.
On the Attitude Scale, 58% agreed/strongly agreed with: “I would rather live a shorter life than lose my ability to take care of myself”.
81% agreed/strongly agreed with: “It is more important to me to maintain my thinking ability than to live as long as possible”.

## Slide 20
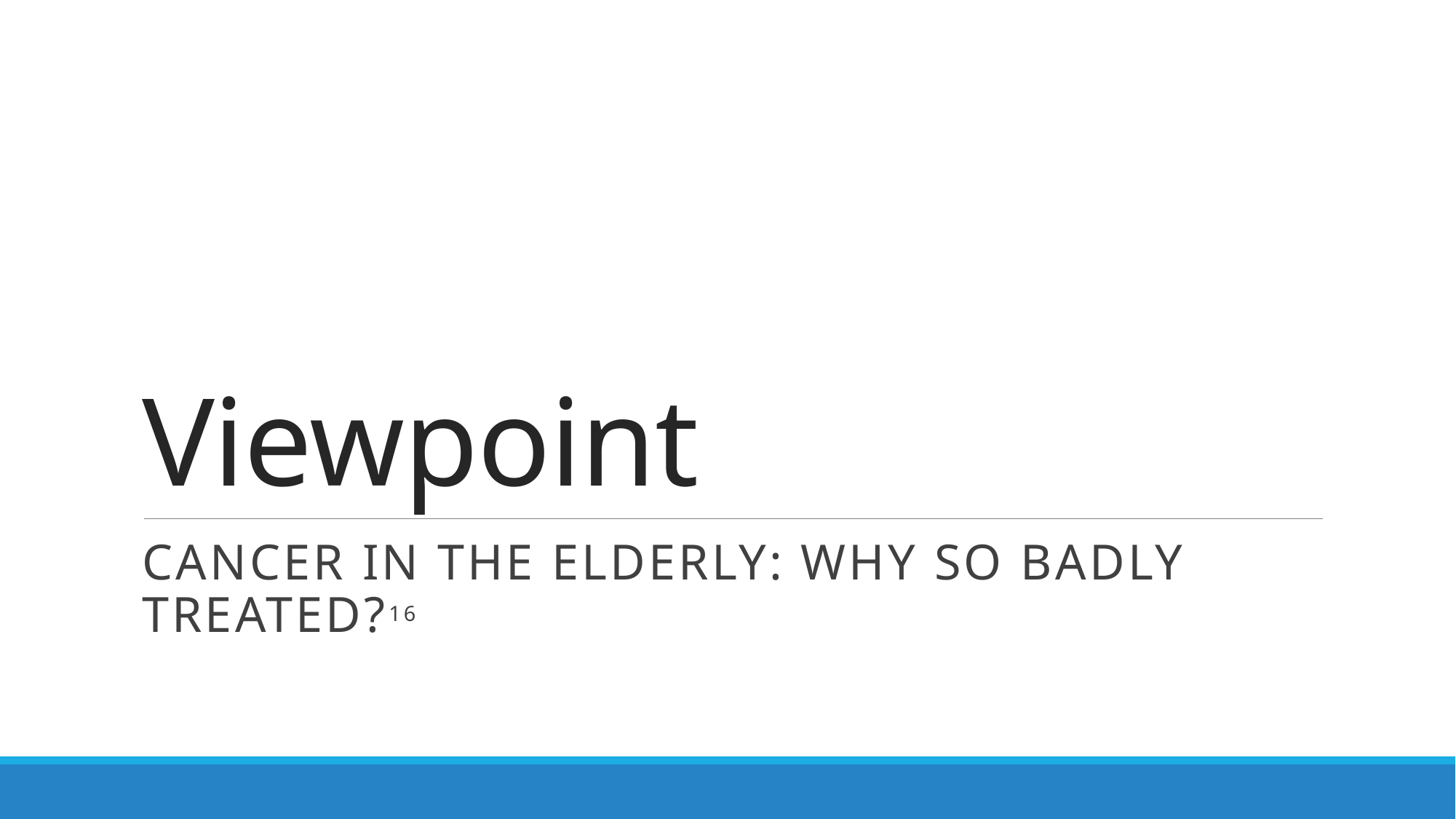

# Viewpoint
Cancer in the elderly: why so badly treated?16

## Slide 21
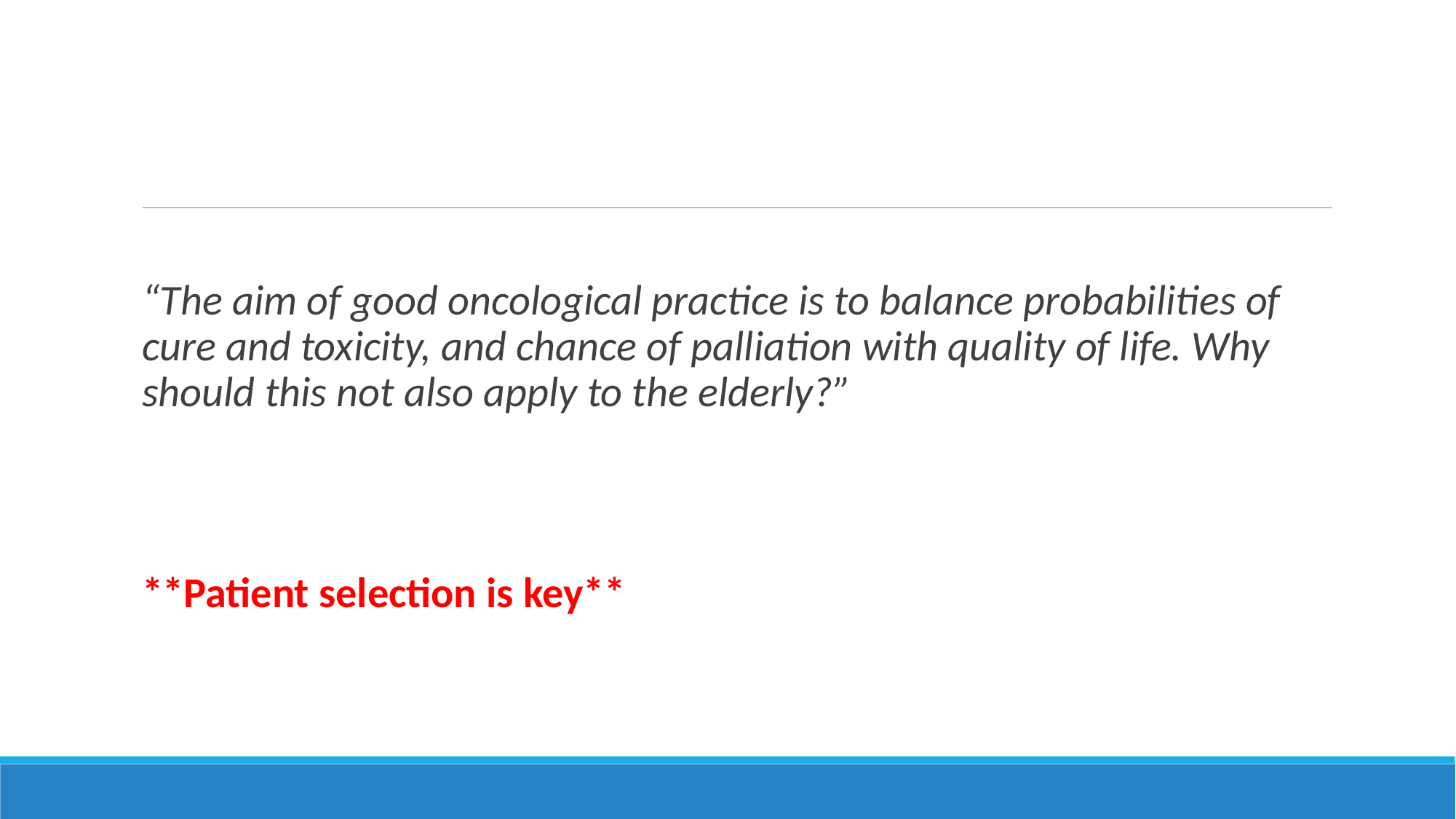

“The aim of good oncological practice is to balance probabilities of cure and toxicity, and chance of palliation with quality of life. Why should this not also apply to the elderly?”
**Patient selection is key**

## Slide 22
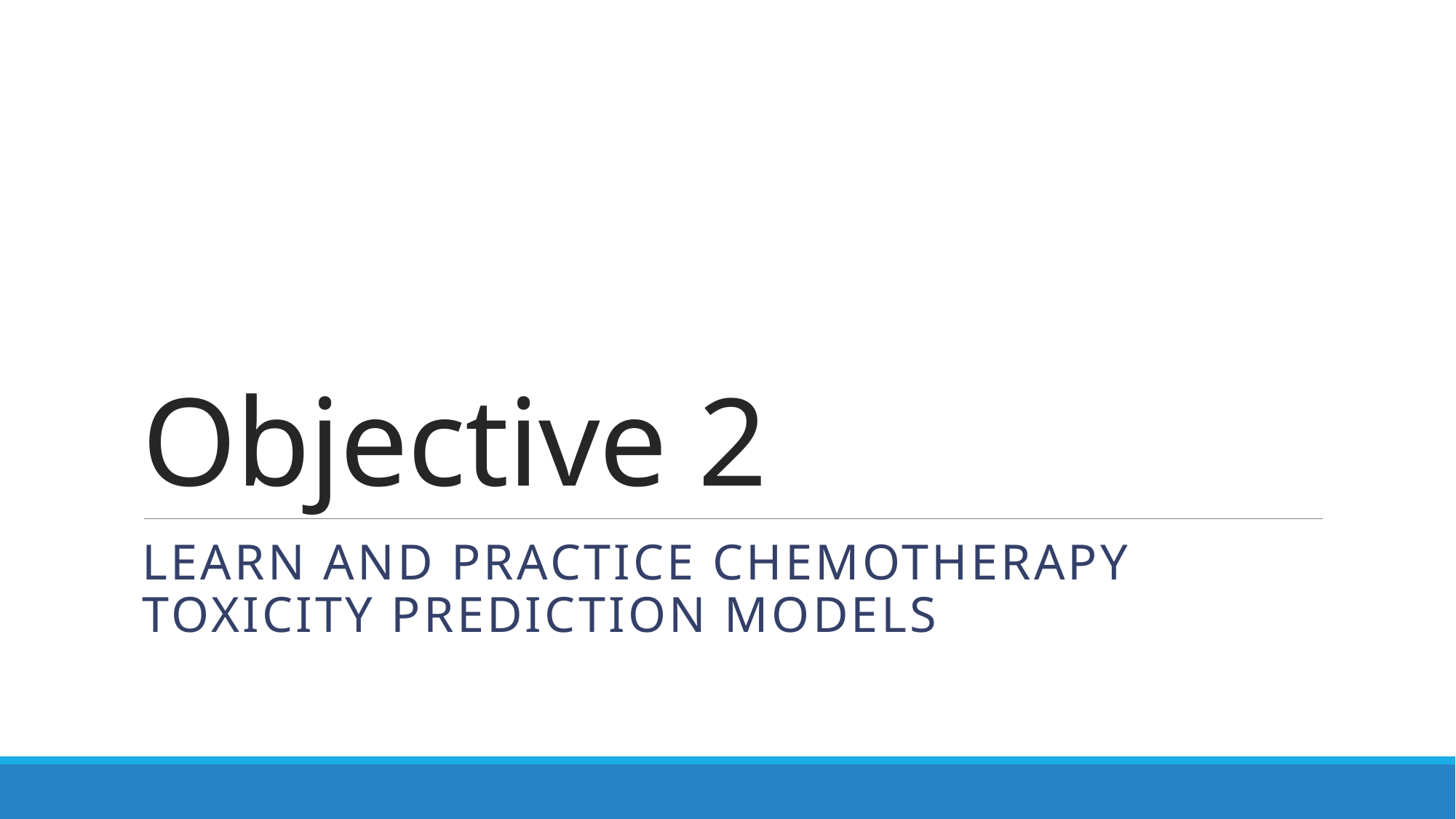

# Objective 2
Learn and practice chemotherapy toxicity prediction models

## Slide 23
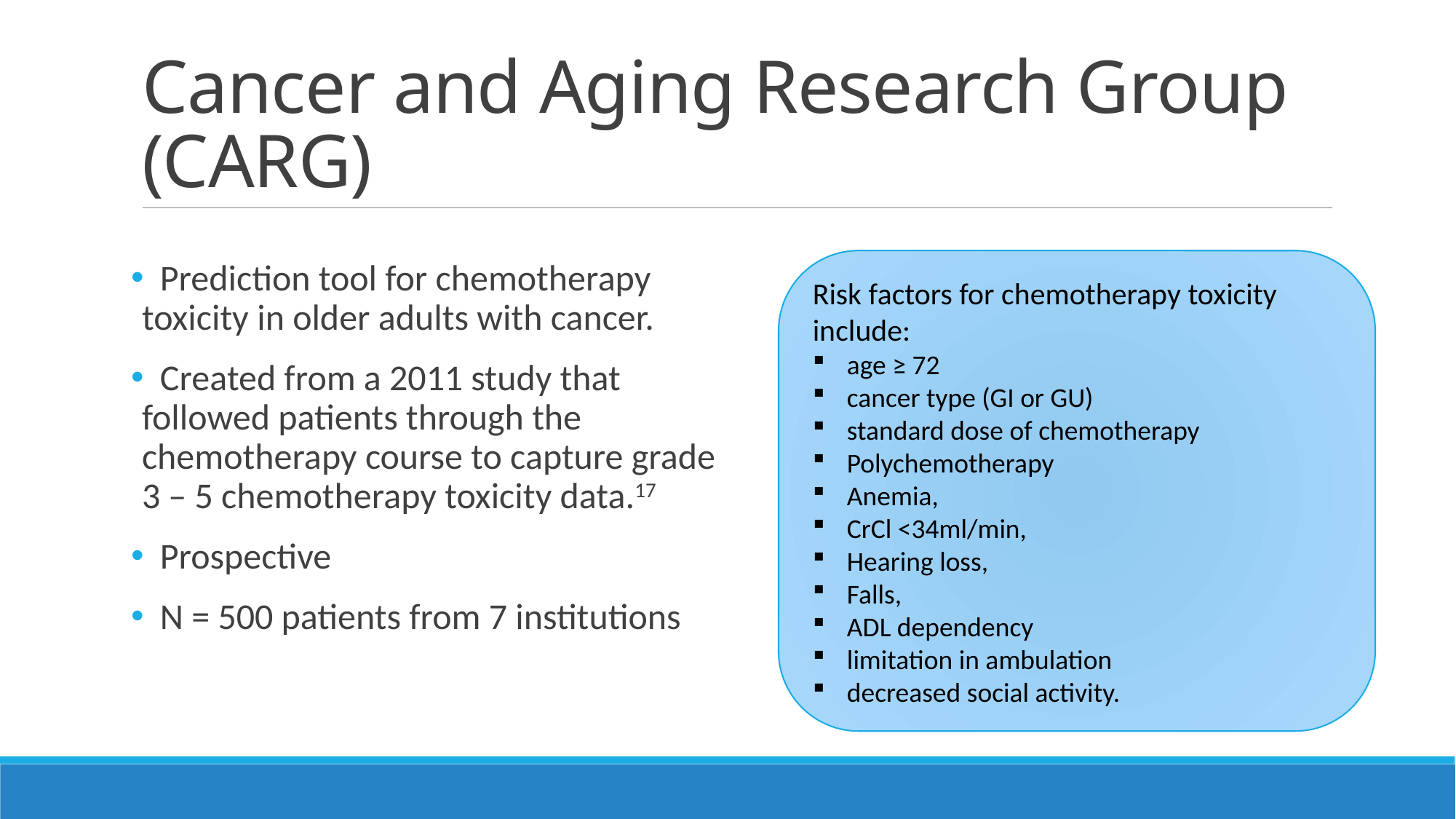

# Cancer and Aging Research Group (CARG)
Risk factors for chemotherapy toxicity include:
age ≥ 72
cancer type (GI or GU)
standard dose of chemotherapy
Polychemotherapy
Anemia,
CrCl <34ml/min,
Hearing loss,
Falls,
ADL dependency
limitation in ambulation
decreased social activity.
 Prediction tool for chemotherapy toxicity in older adults with cancer.
 Created from a 2011 study that followed patients through the chemotherapy course to capture grade 3 – 5 chemotherapy toxicity data.17
 Prospective
 N = 500 patients from 7 institutions

## Slide 24
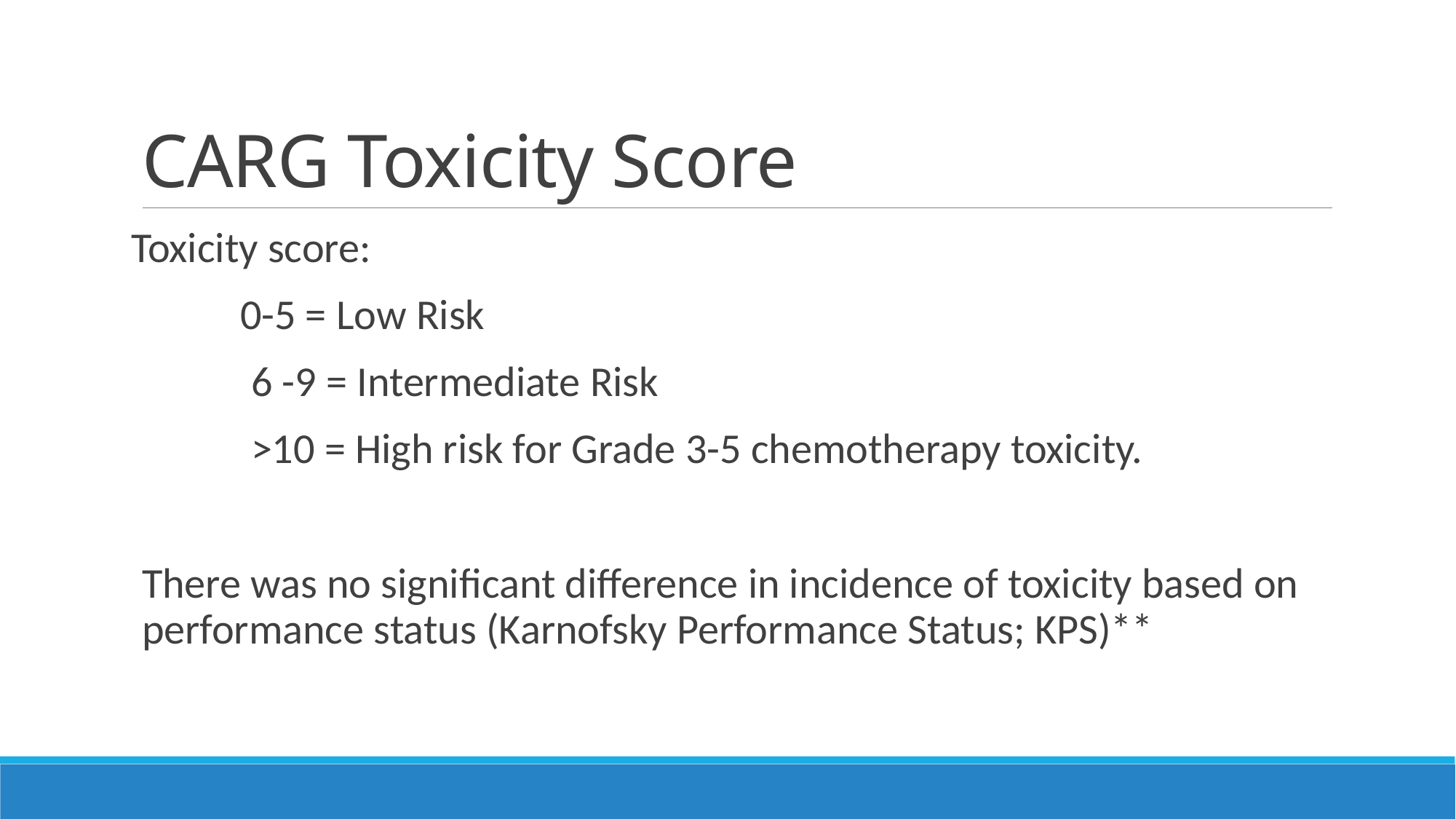

# CARG Toxicity Score
Toxicity score:
	0-5 = Low Risk
 	6 -9 = Intermediate Risk
 	>10 = High risk for Grade 3-5 chemotherapy toxicity.
There was no significant difference in incidence of toxicity based on performance status (Karnofsky Performance Status; KPS)**

## Slide 25
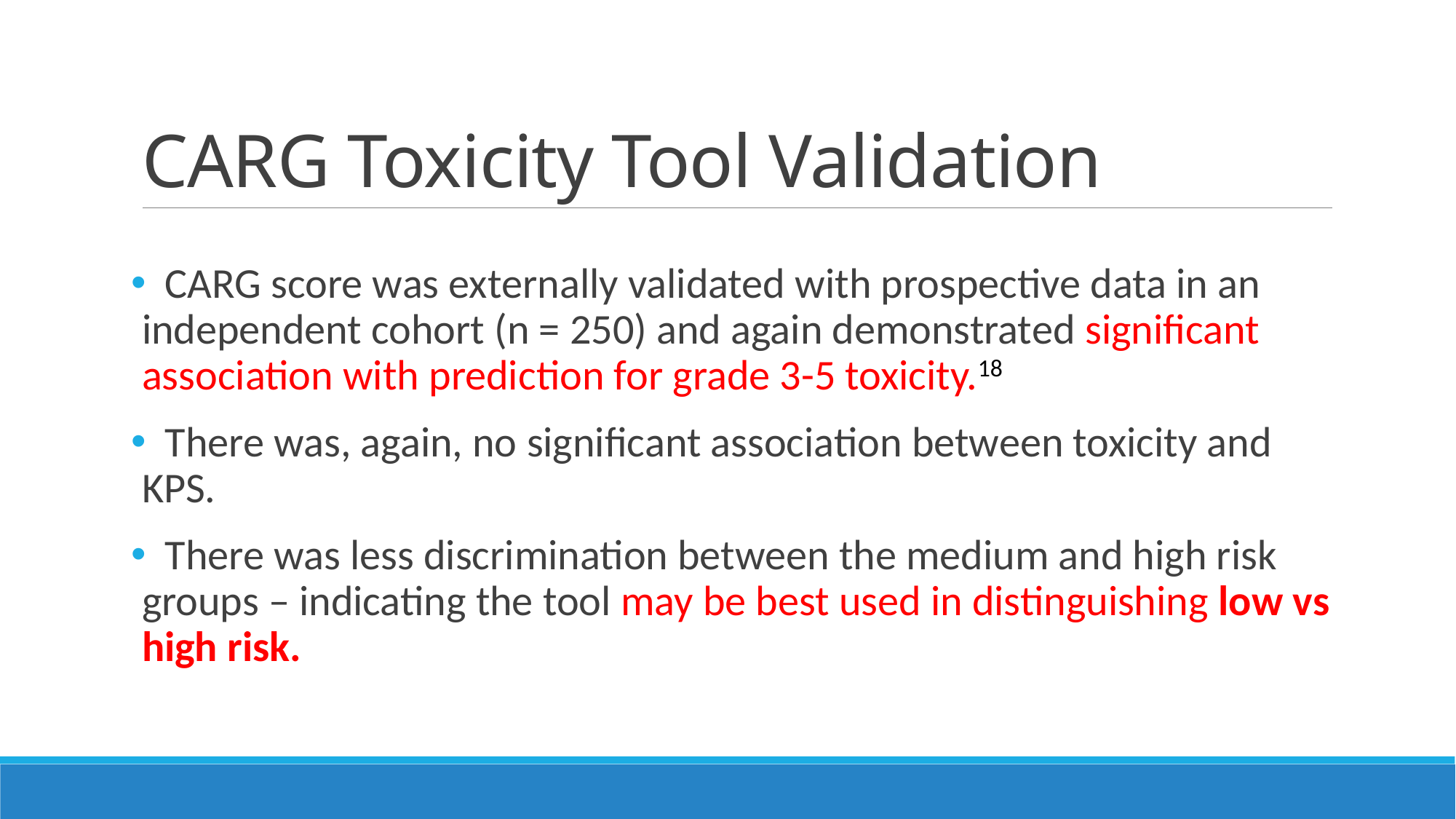

# CARG Toxicity Tool Validation
 CARG score was externally validated with prospective data in an independent cohort (n = 250) and again demonstrated significant association with prediction for grade 3-5 toxicity.18
 There was, again, no significant association between toxicity and KPS.
 There was less discrimination between the medium and high risk groups – indicating the tool may be best used in distinguishing low vs high risk.

## Slide 26
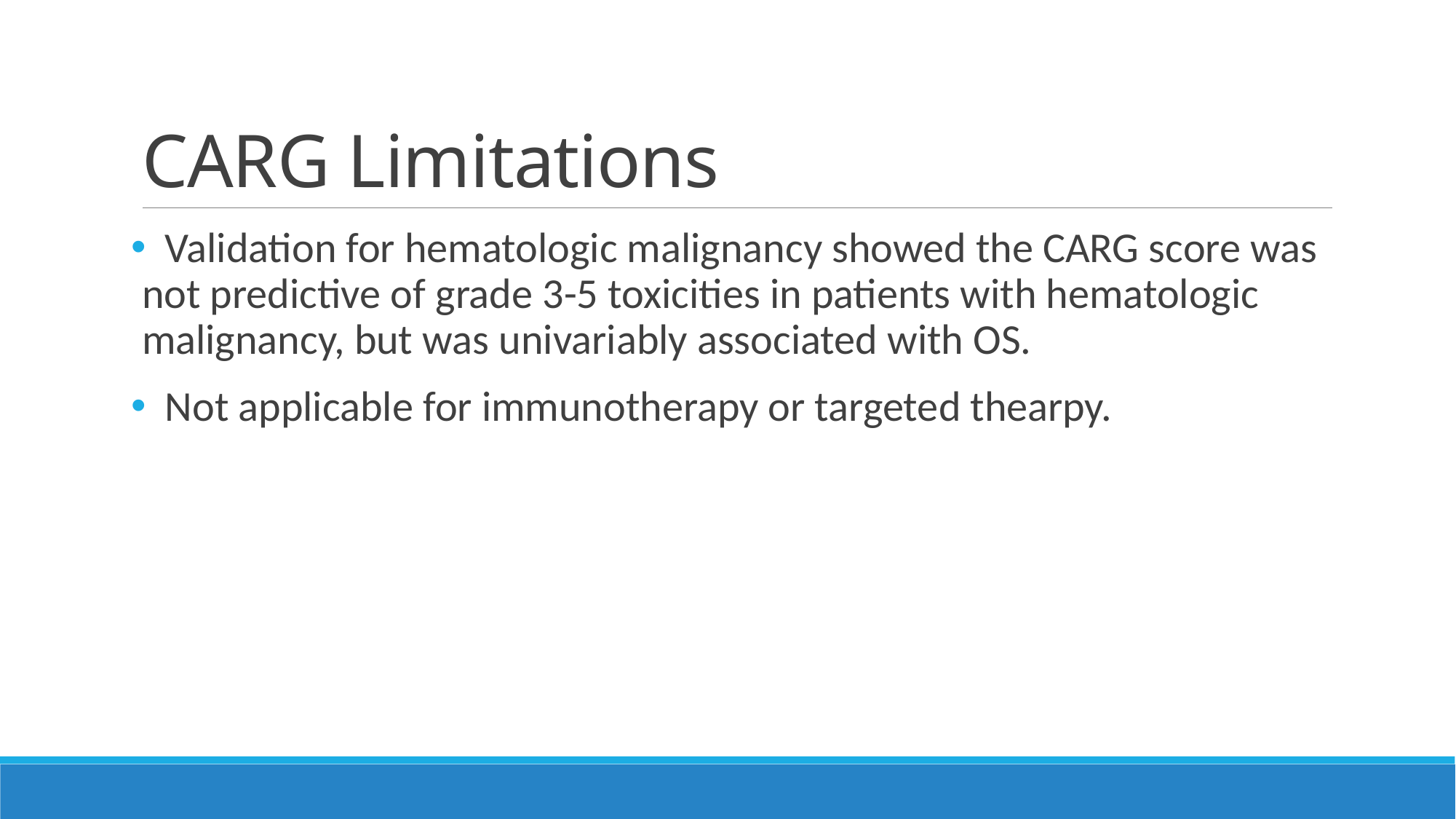

# CARG Limitations
 Validation for hematologic malignancy showed the CARG score was not predictive of grade 3-5 toxicities in patients with hematologic malignancy, but was univariably associated with OS.
 Not applicable for immunotherapy or targeted thearpy.

## Slide 27
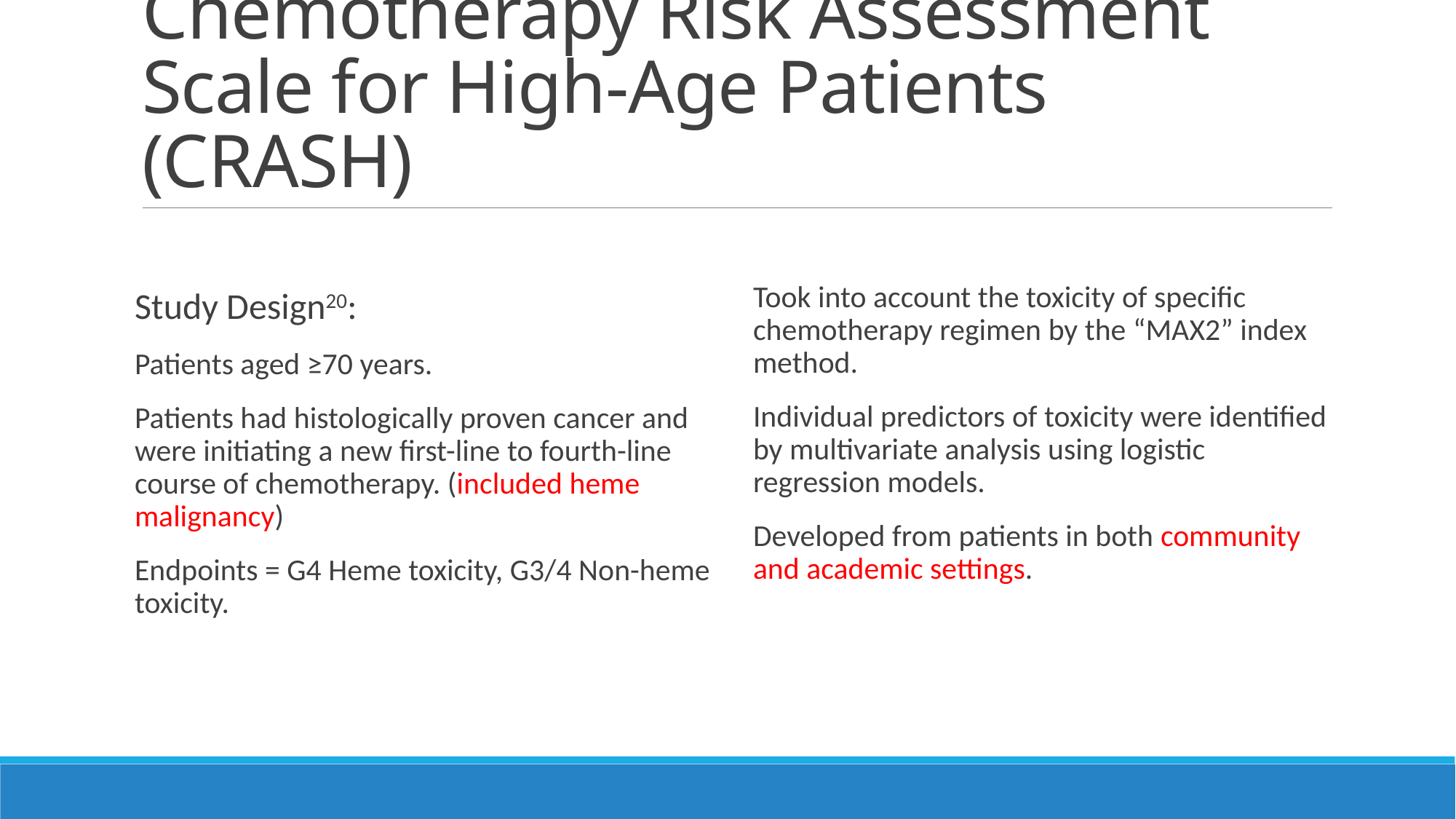

# Chemotherapy Risk Assessment Scale for High-Age Patients (CRASH)
Took into account the toxicity of specific chemotherapy regimen by the “MAX2” index method.
Individual predictors of toxicity were identified by multivariate analysis using logistic regression models.
Developed from patients in both community and academic settings.
Study Design20:
Patients aged ≥70 years.
Patients had histologically proven cancer and were initiating a new first-line to fourth-line course of chemotherapy. (included heme malignancy)
Endpoints = G4 Heme toxicity, G3/4 Non-heme toxicity.

## Slide 28
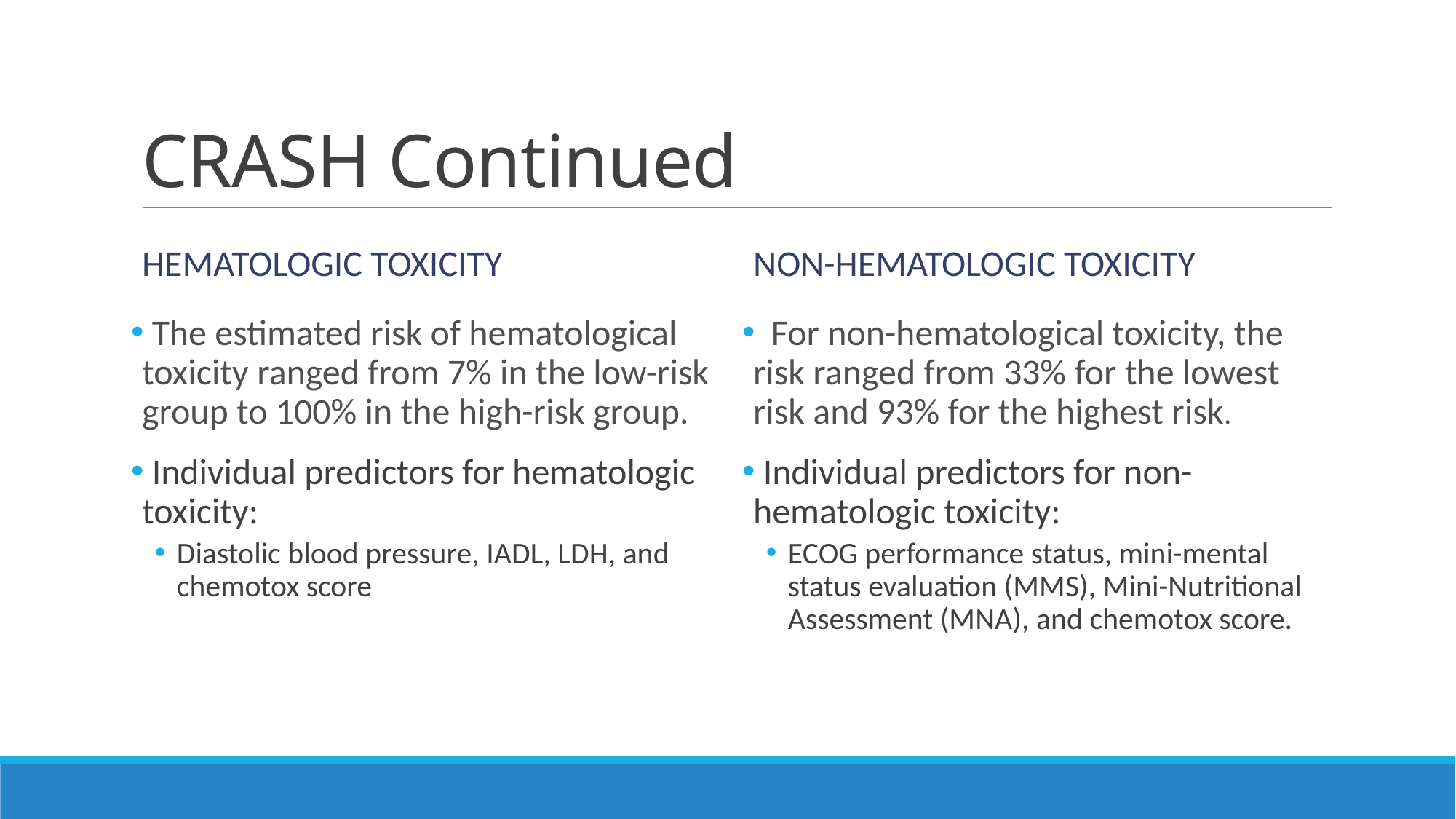

# CRASH Continued
Hematologic Toxicity
Non-Hematologic toxicity
 For non-hematological toxicity, the risk ranged from 33% for the lowest risk and 93% for the highest risk.
 Individual predictors for non-hematologic toxicity:
ECOG performance status, mini-mental status evaluation (MMS), Mini-Nutritional Assessment (MNA), and chemotox score.
 The estimated risk of hematological toxicity ranged from 7% in the low-risk group to 100% in the high-risk group.
 Individual predictors for hematologic toxicity:
Diastolic blood pressure, IADL, LDH, and chemotox score

## Slide 29
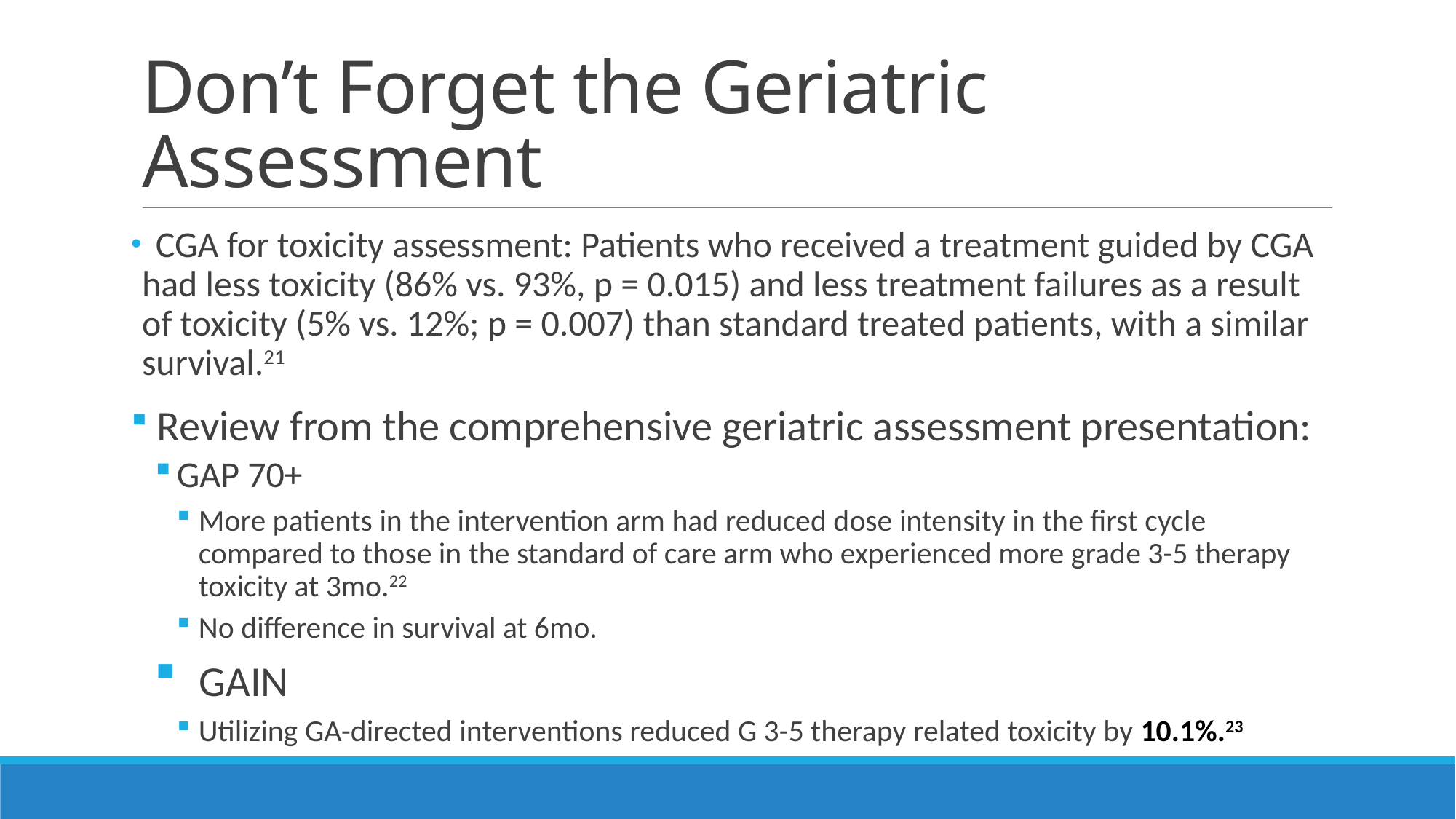

# Don’t Forget the Geriatric Assessment
 CGA for toxicity assessment: Patients who received a treatment guided by CGA had less toxicity (86% vs. 93%, p = 0.015) and less treatment failures as a result of toxicity (5% vs. 12%; p = 0.007) than standard treated patients, with a similar survival.21
 Review from the comprehensive geriatric assessment presentation:
GAP 70+
More patients in the intervention arm had reduced dose intensity in the first cycle compared to those in the standard of care arm who experienced more grade 3-5 therapy toxicity at 3mo.22
No difference in survival at 6mo.
 GAIN
Utilizing GA-directed interventions reduced G 3-5 therapy related toxicity by 10.1%.23

## Slide 30
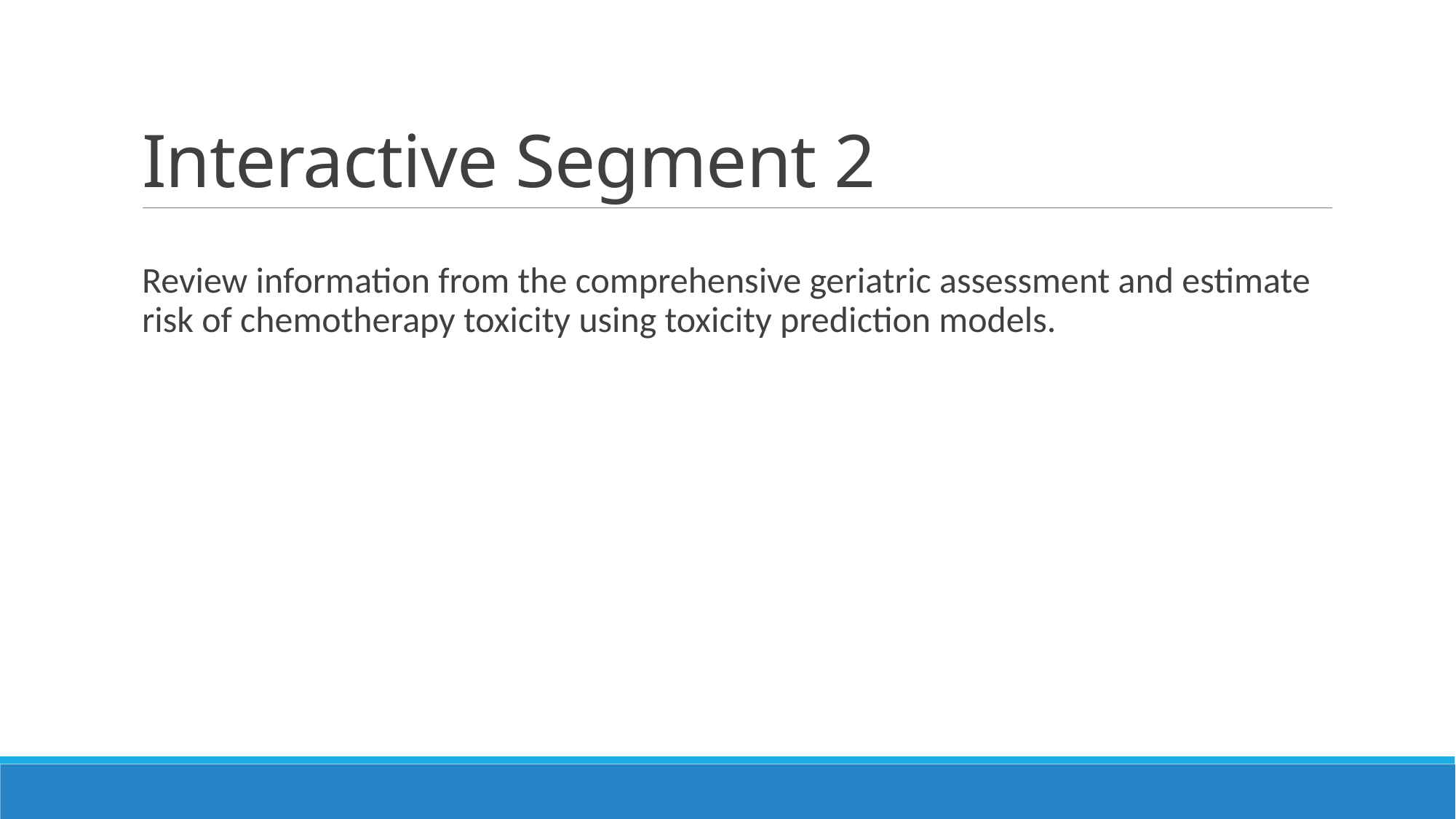

# Interactive Segment 2
Review information from the comprehensive geriatric assessment and estimate risk of chemotherapy toxicity using toxicity prediction models.

## Slide 31
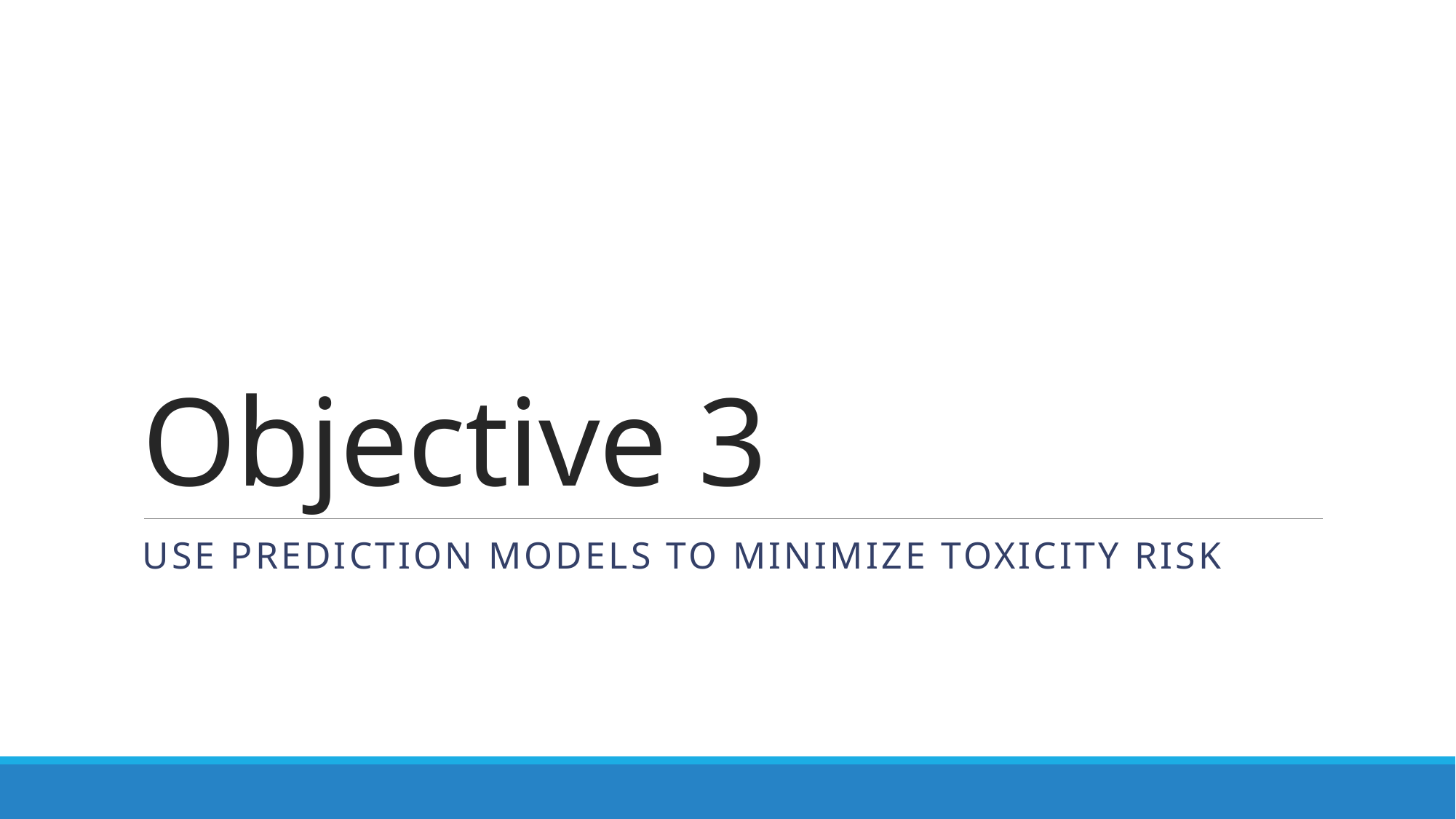

# Objective 3
Use prediction models to minimize toxicity risk

## Slide 32
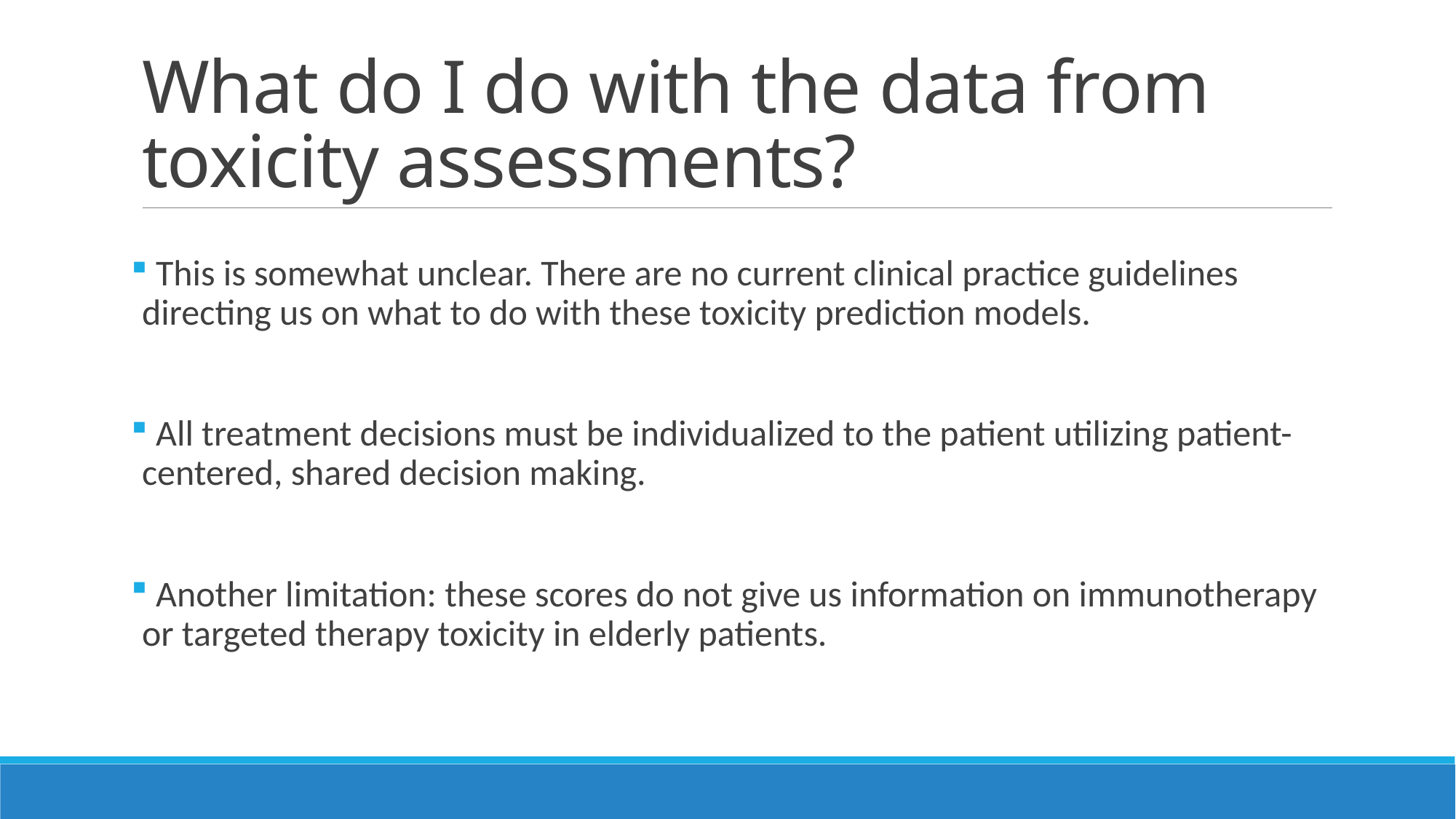

# What do I do with the data from toxicity assessments?
 This is somewhat unclear. There are no current clinical practice guidelines directing us on what to do with these toxicity prediction models.
 All treatment decisions must be individualized to the patient utilizing patient-centered, shared decision making.
 Another limitation: these scores do not give us information on immunotherapy or targeted therapy toxicity in elderly patients.

## Slide 33
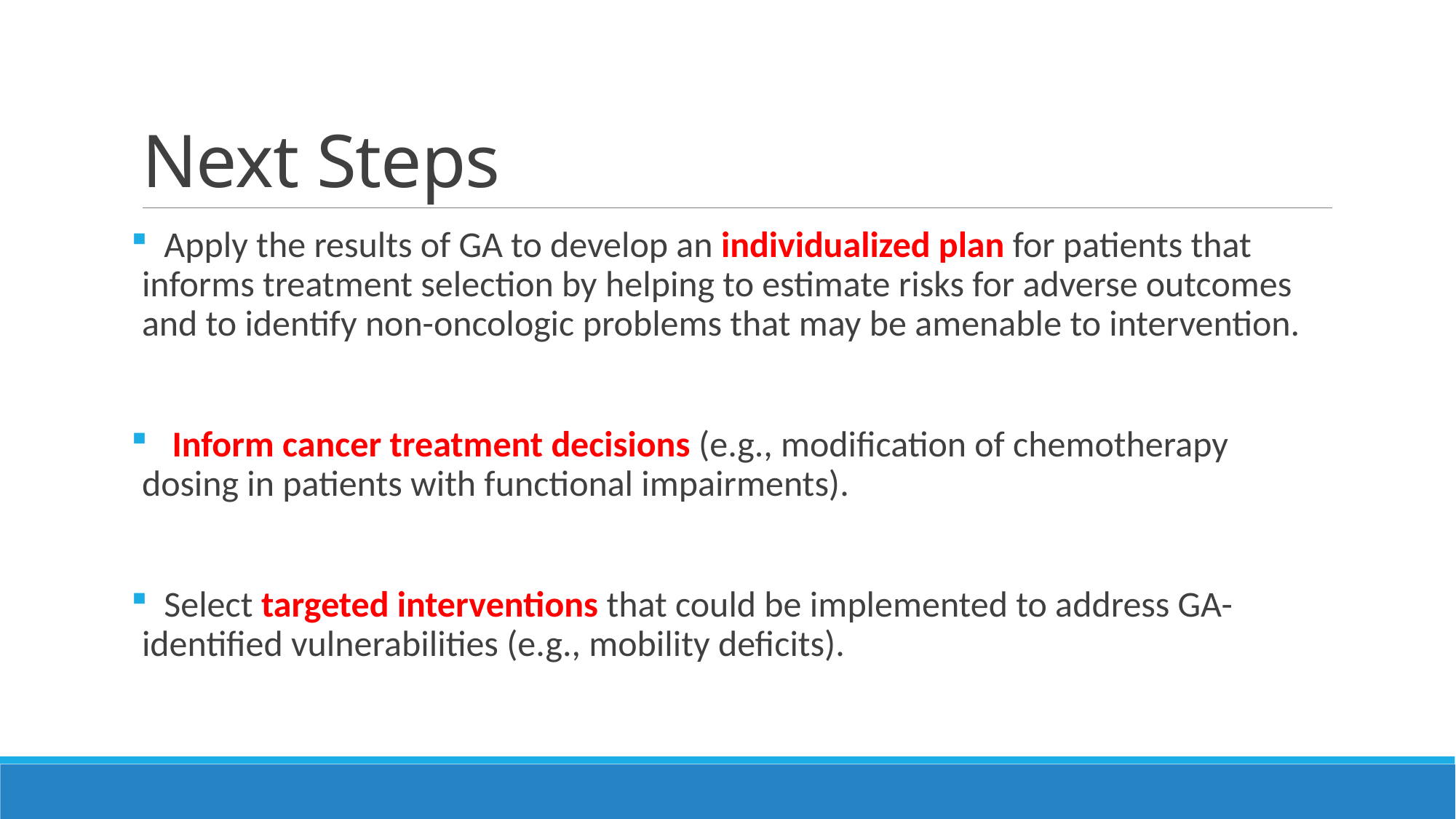

# Next Steps
 Apply the results of GA to develop an individualized plan for patients that informs treatment selection by helping to estimate risks for adverse outcomes and to identify non-oncologic problems that may be amenable to intervention.
 Inform cancer treatment decisions (e.g., modification of chemotherapy dosing in patients with functional impairments).
 Select targeted interventions that could be implemented to address GA-identified vulnerabilities (e.g., mobility deficits).

## Slide 34
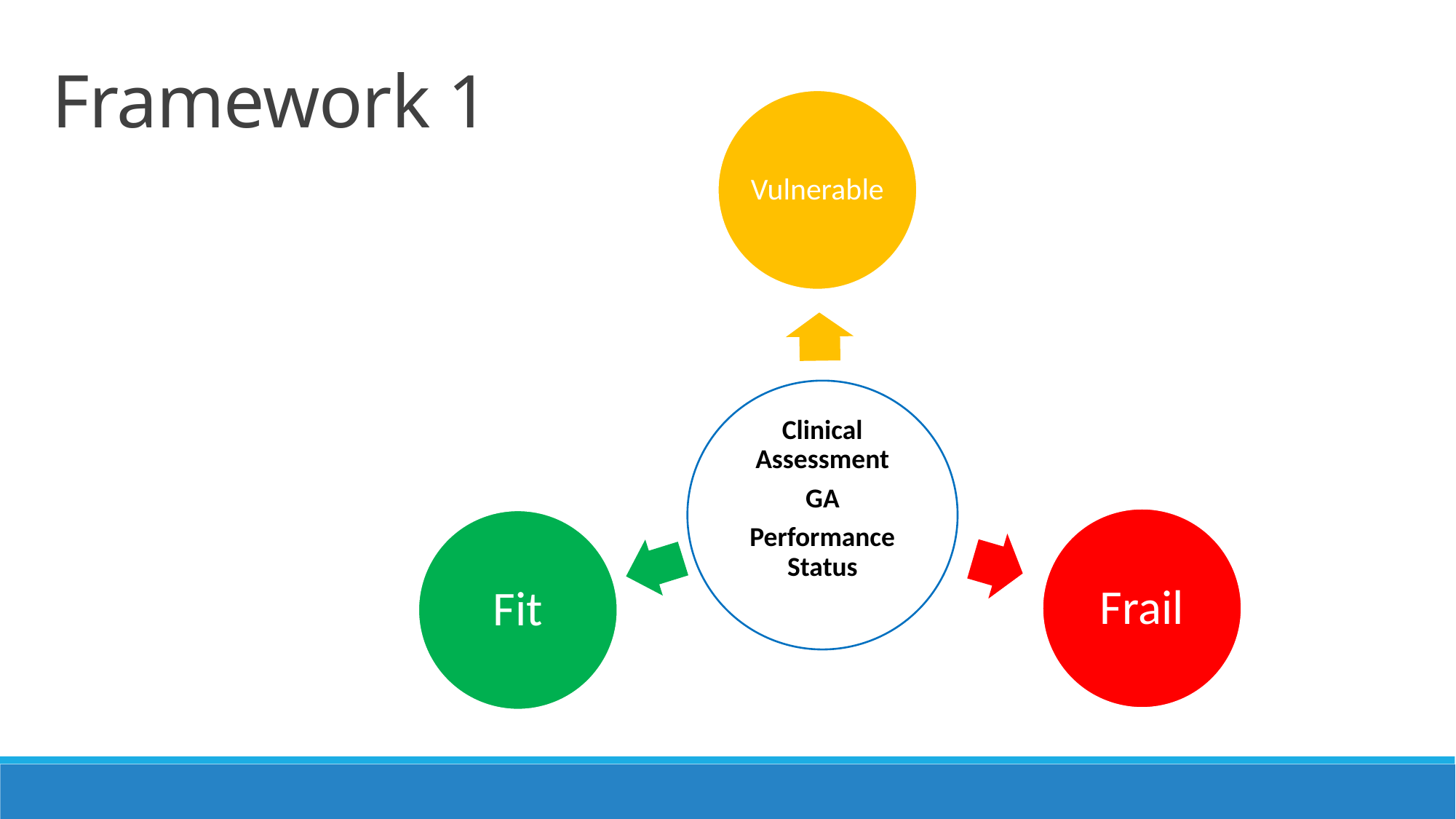

Framework 1

## Slide 35
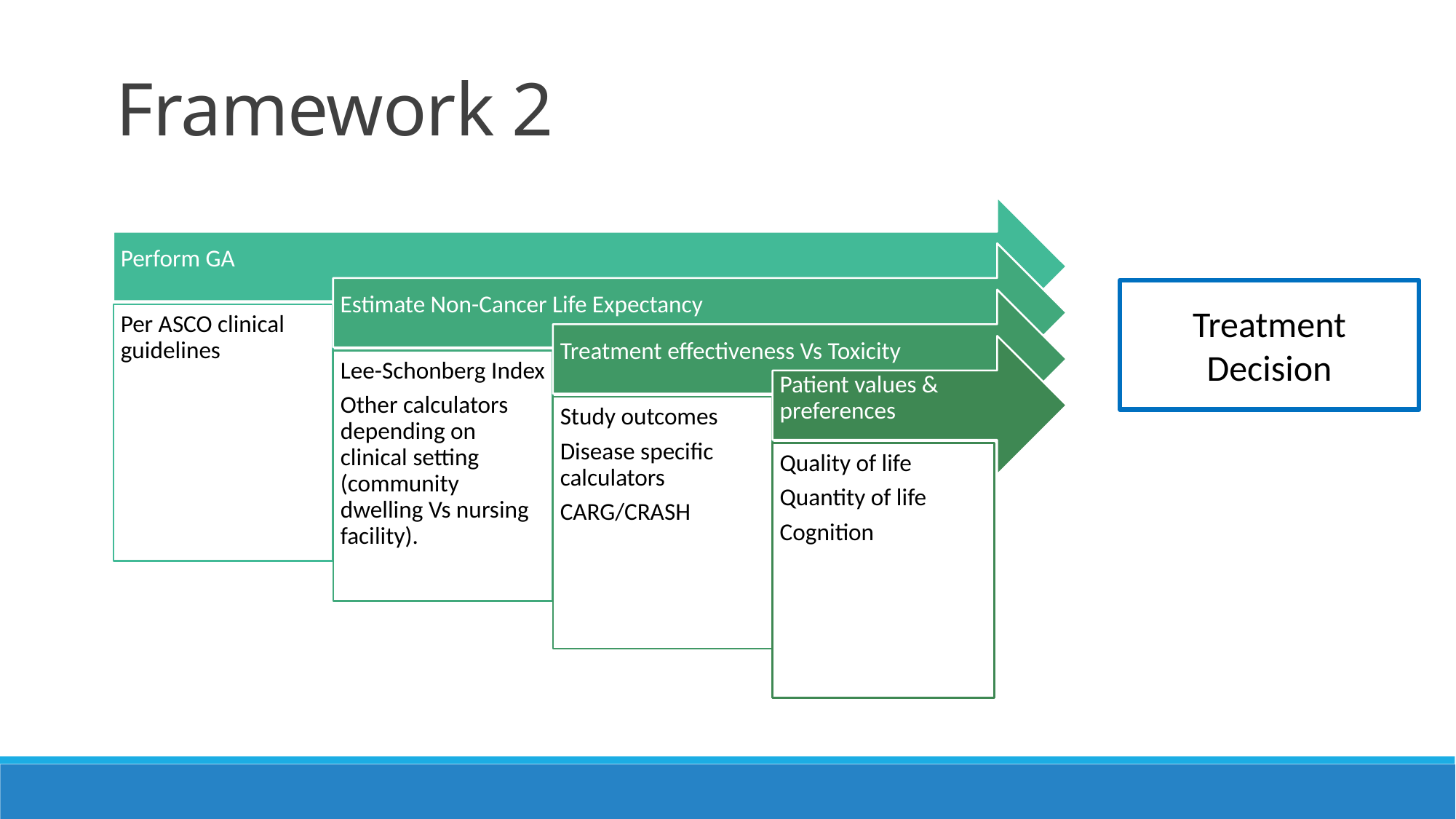

Framework 2
Treatment Decision

## Slide 36
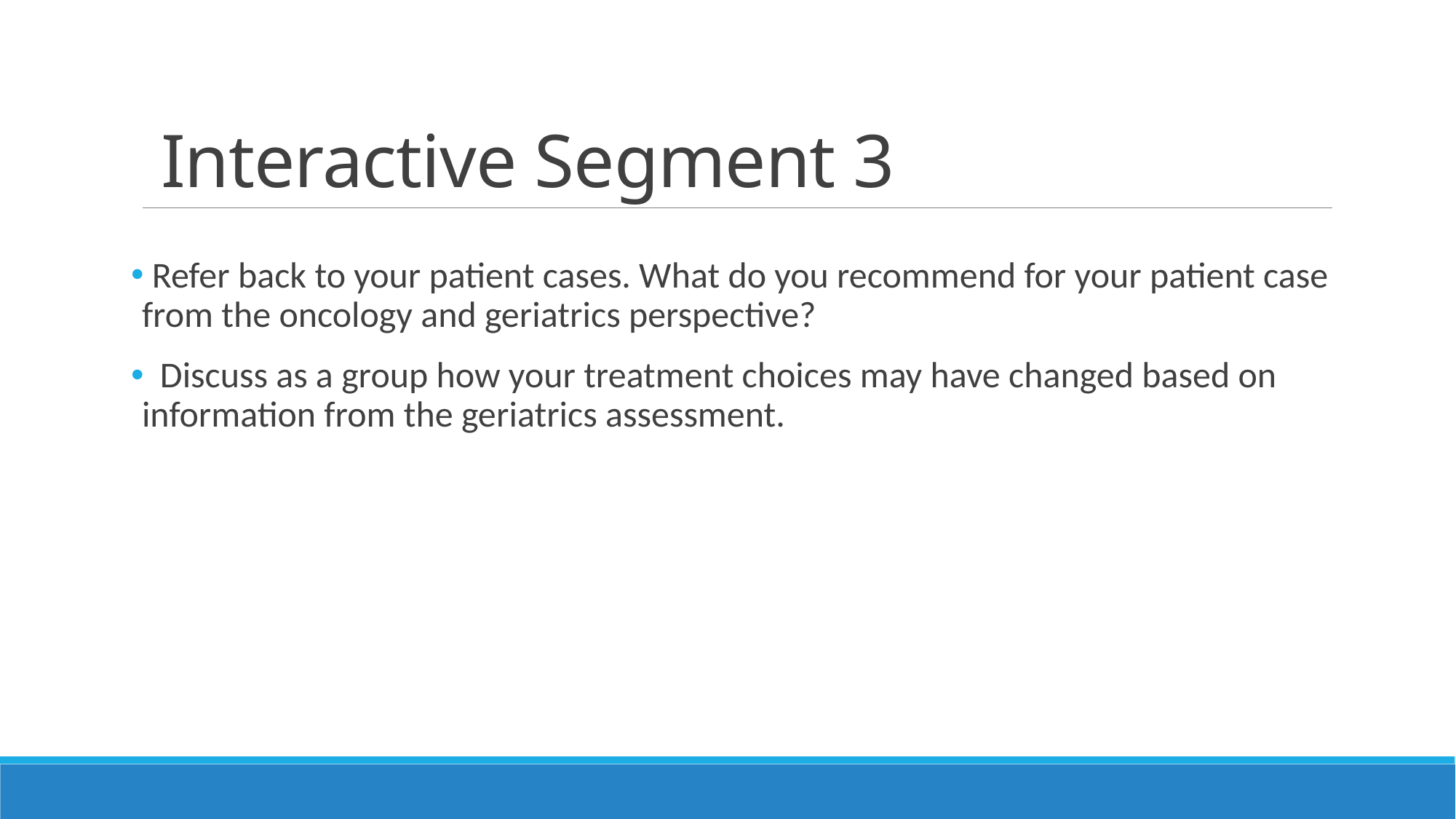

# Interactive Segment 3
 Refer back to your patient cases. What do you recommend for your patient case from the oncology and geriatrics perspective?
 Discuss as a group how your treatment choices may have changed based on information from the geriatrics assessment.

## Slide 37
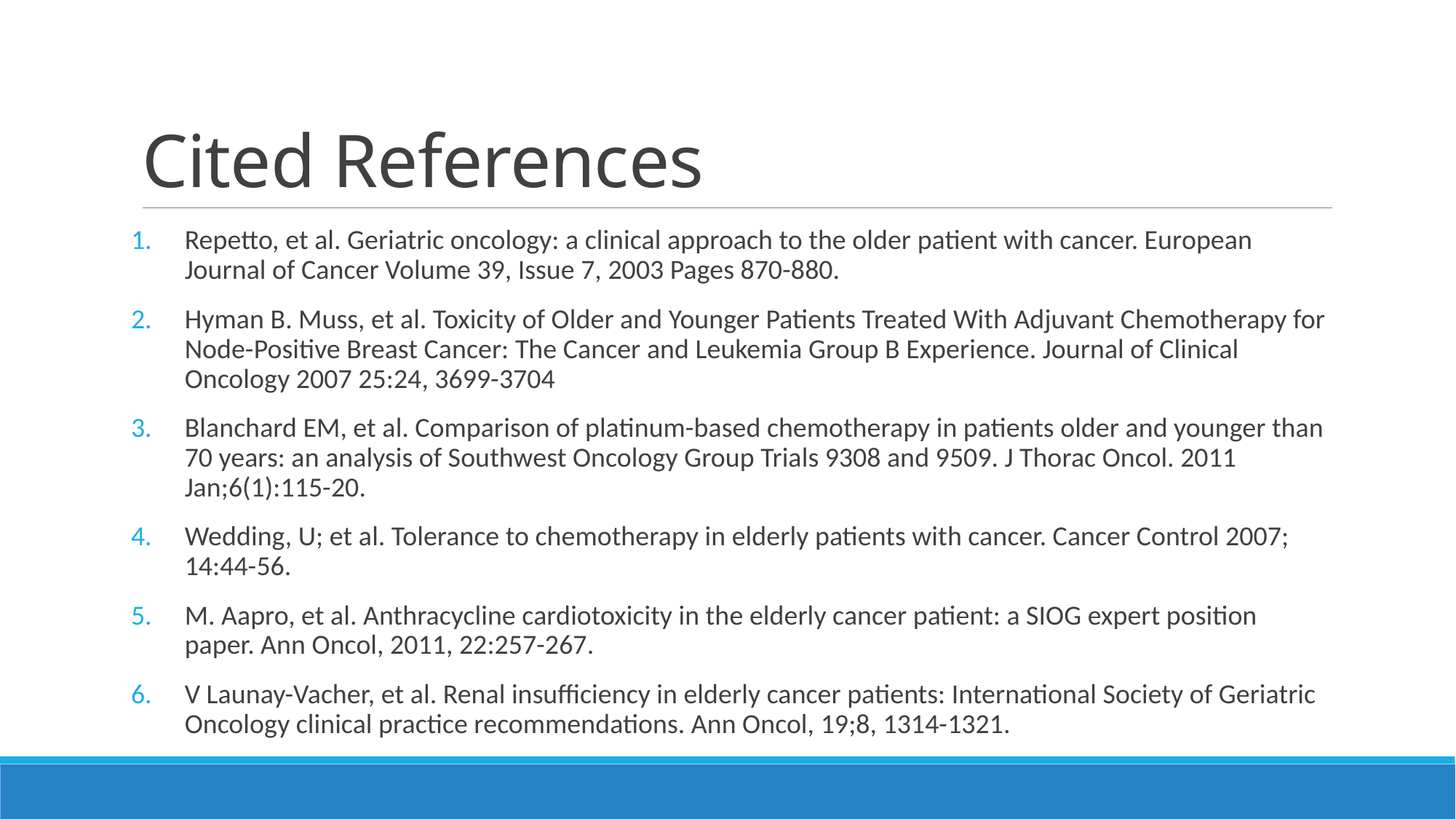

# Cited References
Repetto, et al. Geriatric oncology: a clinical approach to the older patient with cancer. European Journal of Cancer Volume 39, Issue 7, 2003 Pages 870-880.
Hyman B. Muss, et al. Toxicity of Older and Younger Patients Treated With Adjuvant Chemotherapy for Node-Positive Breast Cancer: The Cancer and Leukemia Group B Experience. Journal of Clinical Oncology 2007 25:24, 3699-3704
Blanchard EM, et al. Comparison of platinum-based chemotherapy in patients older and younger than 70 years: an analysis of Southwest Oncology Group Trials 9308 and 9509. J Thorac Oncol. 2011 Jan;6(1):115-20.
Wedding, U; et al. Tolerance to chemotherapy in elderly patients with cancer. Cancer Control 2007; 14:44-56.
M. Aapro, et al. Anthracycline cardiotoxicity in the elderly cancer patient: a SIOG expert position paper. Ann Oncol, 2011, 22:257-267.
V Launay-Vacher, et al. Renal insufficiency in elderly cancer patients: International Society of Geriatric Oncology clinical practice recommendations. Ann Oncol, 19;8, 1314-1321.

## Slide 38
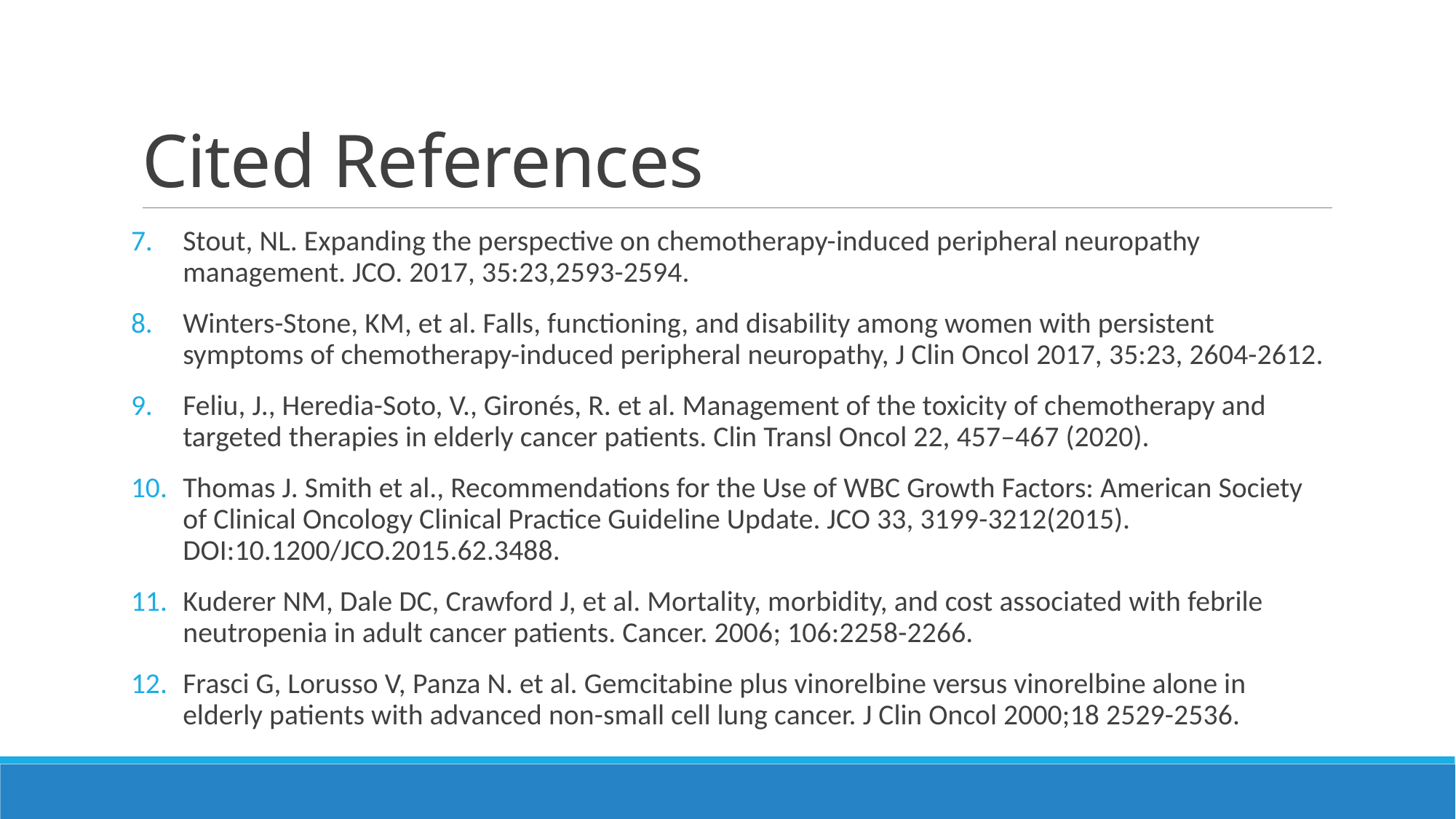

# Cited References
Stout, NL. Expanding the perspective on chemotherapy-induced peripheral neuropathy management. JCO. 2017, 35:23,2593-2594.
Winters-Stone, KM, et al. Falls, functioning, and disability among women with persistent symptoms of chemotherapy-induced peripheral neuropathy, J Clin Oncol 2017, 35:23, 2604-2612.
Feliu, J., Heredia-Soto, V., Gironés, R. et al. Management of the toxicity of chemotherapy and targeted therapies in elderly cancer patients. Clin Transl Oncol 22, 457–467 (2020).
Thomas J. Smith et al., Recommendations for the Use of WBC Growth Factors: American Society of Clinical Oncology Clinical Practice Guideline Update. JCO 33, 3199-3212(2015). DOI:10.1200/JCO.2015.62.3488.
Kuderer NM, Dale DC, Crawford J, et al. Mortality, morbidity, and cost associated with febrile neutropenia in adult cancer patients. Cancer. 2006; 106:2258-2266.
Frasci G, Lorusso V, Panza N. et al. Gemcitabine plus vinorelbine versus vinorelbine alone in elderly patients with advanced non-small cell lung cancer. J Clin Oncol 2000;18 2529-2536.

## Slide 39
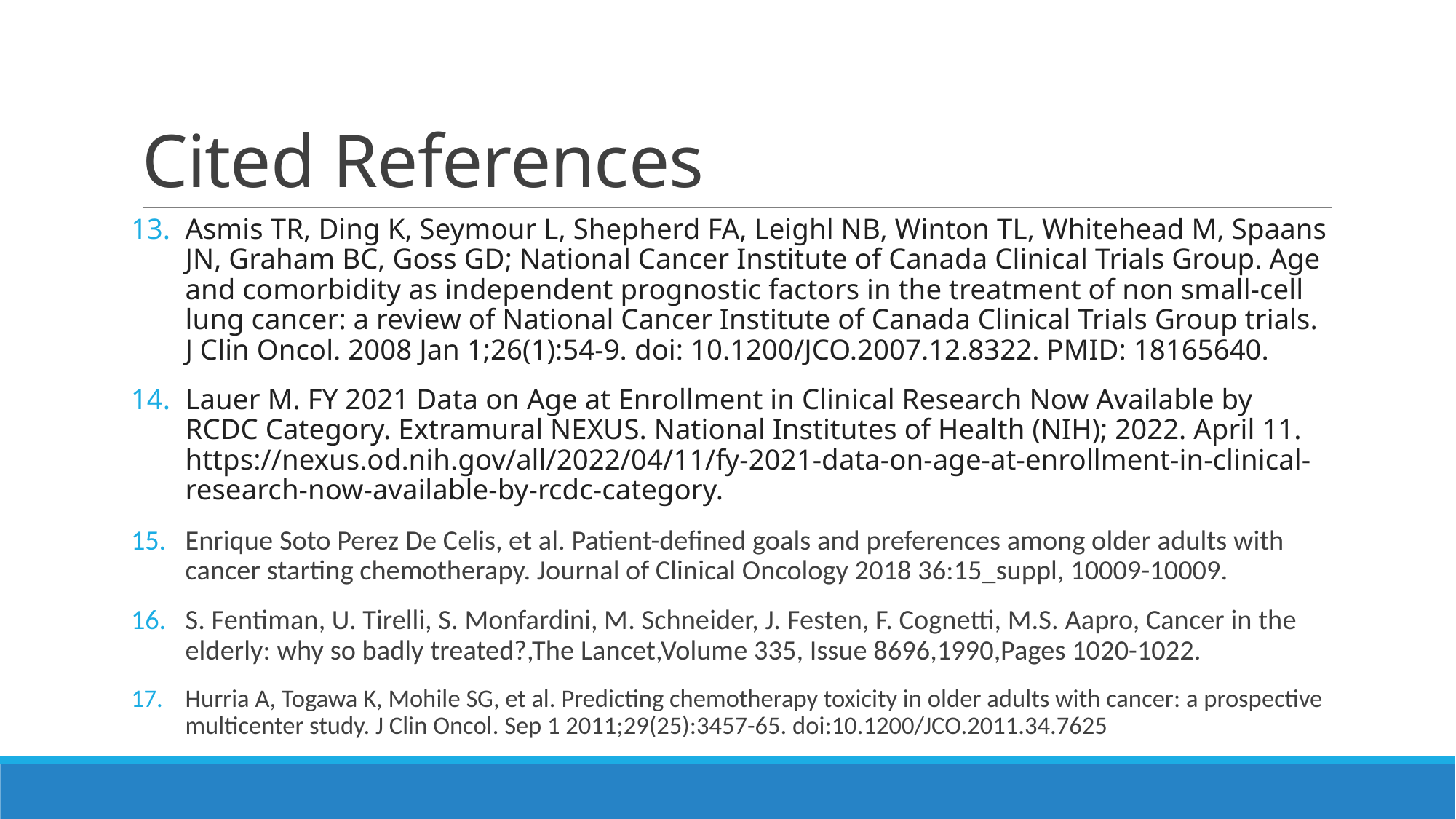

# Cited References
Asmis TR, Ding K, Seymour L, Shepherd FA, Leighl NB, Winton TL, Whitehead M, Spaans JN, Graham BC, Goss GD; National Cancer Institute of Canada Clinical Trials Group. Age and comorbidity as independent prognostic factors in the treatment of non small-cell lung cancer: a review of National Cancer Institute of Canada Clinical Trials Group trials. J Clin Oncol. 2008 Jan 1;26(1):54-9. doi: 10.1200/JCO.2007.12.8322. PMID: 18165640.
Lauer M. FY 2021 Data on Age at Enrollment in Clinical Research Now Available by RCDC Category. Extramural NEXUS. National Institutes of Health (NIH); 2022. April 11. https://nexus.od.nih.gov/all/2022/04/11/fy-2021-data-on-age-at-enrollment-in-clinical-research-now-available-by-rcdc-category.
Enrique Soto Perez De Celis, et al. Patient-defined goals and preferences among older adults with cancer starting chemotherapy. Journal of Clinical Oncology 2018 36:15_suppl, 10009-10009.
S. Fentiman, U. Tirelli, S. Monfardini, M. Schneider, J. Festen, F. Cognetti, M.S. Aapro, Cancer in the elderly: why so badly treated?,The Lancet,Volume 335, Issue 8696,1990,Pages 1020-1022.
Hurria A, Togawa K, Mohile SG, et al. Predicting chemotherapy toxicity in older adults with cancer: a prospective multicenter study. J Clin Oncol. Sep 1 2011;29(25):3457-65. doi:10.1200/JCO.2011.34.7625

## Slide 40
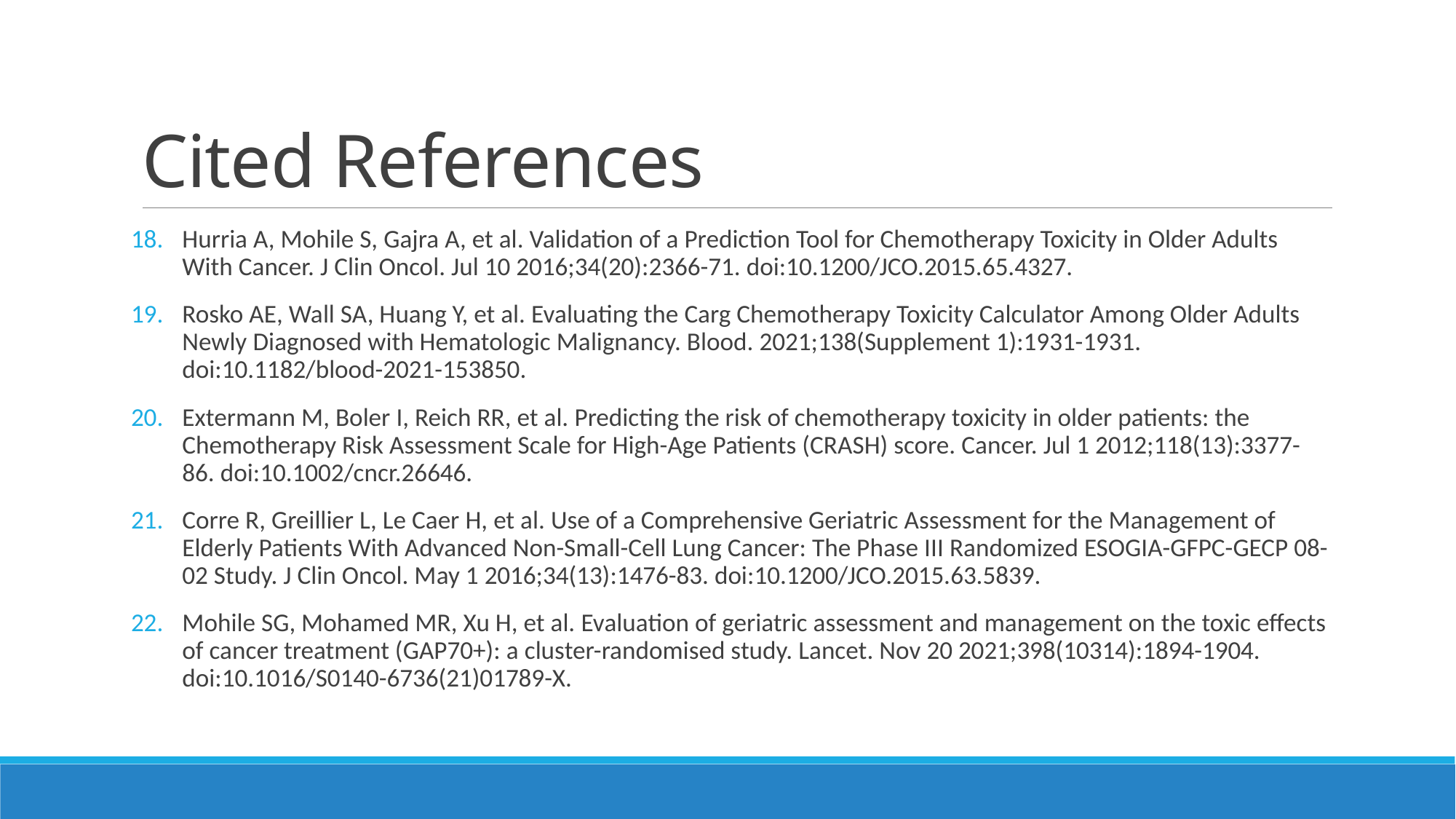

# Cited References
Hurria A, Mohile S, Gajra A, et al. Validation of a Prediction Tool for Chemotherapy Toxicity in Older Adults With Cancer. J Clin Oncol. Jul 10 2016;34(20):2366-71. doi:10.1200/JCO.2015.65.4327.
Rosko AE, Wall SA, Huang Y, et al. Evaluating the Carg Chemotherapy Toxicity Calculator Among Older Adults Newly Diagnosed with Hematologic Malignancy. Blood. 2021;138(Supplement 1):1931-1931. doi:10.1182/blood-2021-153850.
Extermann M, Boler I, Reich RR, et al. Predicting the risk of chemotherapy toxicity in older patients: the Chemotherapy Risk Assessment Scale for High-Age Patients (CRASH) score. Cancer. Jul 1 2012;118(13):3377-86. doi:10.1002/cncr.26646.
Corre R, Greillier L, Le Caer H, et al. Use of a Comprehensive Geriatric Assessment for the Management of Elderly Patients With Advanced Non-Small-Cell Lung Cancer: The Phase III Randomized ESOGIA-GFPC-GECP 08-02 Study. J Clin Oncol. May 1 2016;34(13):1476-83. doi:10.1200/JCO.2015.63.5839.
Mohile SG, Mohamed MR, Xu H, et al. Evaluation of geriatric assessment and management on the toxic effects of cancer treatment (GAP70+): a cluster-randomised study. Lancet. Nov 20 2021;398(10314):1894-1904. doi:10.1016/S0140-6736(21)01789-X.

## Slide 41
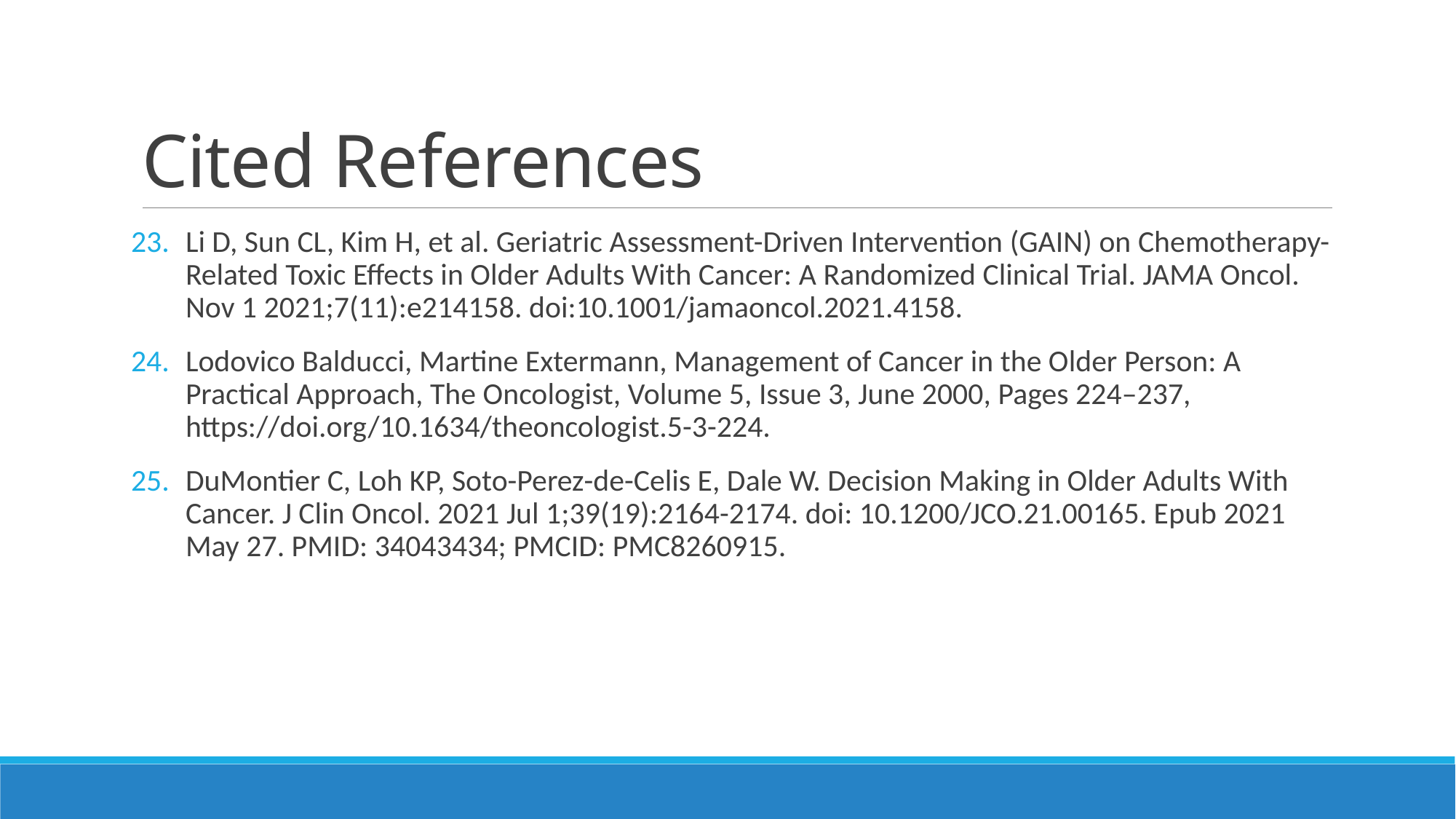

# Cited References
Li D, Sun CL, Kim H, et al. Geriatric Assessment-Driven Intervention (GAIN) on Chemotherapy-Related Toxic Effects in Older Adults With Cancer: A Randomized Clinical Trial. JAMA Oncol. Nov 1 2021;7(11):e214158. doi:10.1001/jamaoncol.2021.4158.
Lodovico Balducci, Martine Extermann, Management of Cancer in the Older Person: A Practical Approach, The Oncologist, Volume 5, Issue 3, June 2000, Pages 224–237, https://doi.org/10.1634/theoncologist.5-3-224.
DuMontier C, Loh KP, Soto-Perez-de-Celis E, Dale W. Decision Making in Older Adults With Cancer. J Clin Oncol. 2021 Jul 1;39(19):2164-2174. doi: 10.1200/JCO.21.00165. Epub 2021 May 27. PMID: 34043434; PMCID: PMC8260915.
