# Supplementary material for: An Introductory Course on Geriatric Oncology
Source: MedEdPORTAL. 2024 Nov 14;20:11471. doi: 10.15766/mep_2374-8265.11471 (PMC11561070; doi:10.15766/mep_2374-8265.11471)
Supplement: Supplementary file 1 — Introduction to Geriatric Oncology.pptxThe Comprehensive Geriatric Assessment.pptxGeriatric Screening Tools.pptxBiology of Aging.pptxCancer Therapy in the Older Adult.pptxSummary of Interactive Sessions.docxSession 5 Patient Case 1.docxSession 5 Patient Case 2.docxSession 5 Patient Case 3.docxGeriatric Oncology Knowledge Assessment.docxKnowledge Assessment Answer Key.docxSelf-Perceived Competency Assessment.docxCurriculum Session Assessment.docx [file mep_2374-8265.11471-s001.zip › F. Summary of Interactive Sessions.docx]

This appendix is used to outline instructions to the interactive activities included within the course presentations. Activities are listed according to the presentation title and the corresponding appendix.

The approximate time required for each individual activity is listed below the activity description.

| Introduction to Geriatric Oncology  (Appendix A) | 1. Geriatric Syndromes and Multimorbidity exercise: Fellows were given 4 plastic bags of geriatric syndromes separately labeled “Osteoporosis, Falls, Cognitive impairment and Polypharmacy”. Each fellow was also given 9 individual pieces of paper labeled “Vitamin D deficiency, Atorvastatin, Osteoarthritis, Cerebrovascular accident, Alcohol use Disorder, Type 2 Diabetes Mellitus with Neuropathy, hypotension, Cholecalciferol and Congestive Heart Failure”. Each fellow places the individual pieces of paper in the plastic bag they thought best contributed to a geriatric syndrome. For example, some fellows placed “Alcohol Use Disorder” in the bag labeled “Falls”, while others placed it in the bag labeled “Cognitive Impairment”. The purpose of this exercise is for learners to visualize how different multimorbidities/medications can contribute to various geriatric syndromes   Approximate activity time: 10-15 minutes   1. Frailty exercise- The purpose of this exercise is for learners to visualize how various medications, hospitalizations, and geriatric syndromes could contribute to frailty. This interactive session involved using the game *Jenga* or a similar wooden tower block game. For example, “hospitalized for a fall with a hip fracture” would have the learners remove a wooden piece that was then set on top. Many different examples can be used for removal of a wooden piece. We gave 5 more examples where pieces were removed and subsequently placed on top of the tower. The wooden block tower was then pushed and compared to an intact tower without any pieces removed. Most of the towers toppled over with light force representing to the group how different factors are additive in contributing to frailty in an individual patient as a whole   Approximate activity time: 5-10 minutes |
| --- | --- |
| The Comprehensive Geriatric Assessment  (Appendix B) | 1. MOCA (*Montreal Cognitive Assessment)* exercise: One learner participated as the patient while their partner was the exam administrator. The learners completed certain parts of the MoCA that included identification of the three animals, drawing of a clock, and sentence repetition. The learners were given cataract simulation goggles made from goggles wrapped with plastic wrap, ear plugs, and gloves with the thumb and index taped together and the other three digits also taped together that simulated neuropathy and arthritis. The purpose of this exercise was for learners to see how certain comorbidities or sensory impairments may influence scores on a cognitive assessment.   Approximate activity time: 10-15 minutes   1. Timed-Up-And-Go and 4 stage balance exercise. The lecture first provided background information on how to implement the timed up-and-go test and the four-stage balance test to assess for fall risk. The learners performed these tasks with popcorn kernels and/or chickpeas placed in their shoes to simulate neuropathy.   Approximate activity time: 5-10 minutes |
| Geriatric Screening tools  (Appendix C) | 1. G8 (Geriatric 8)   Learners were presented with 2 clinical cases. Based on the case, learners developed individual G8 scores. The purpose of this exercise was for learners to visualize what items are included in the G8 screening tool.  Approximate activity time: 5-10 minutes |
| Cancer Therapy in Older Adults  (Appendix E) | 1. Three different clinical cases were given to the learners (see appendix G – I). Test patients within the cases were diagnosed with either non-small cell lung cancer (NSCLC), colorectal cancer (CRC), or acute myeloid leukemia (AML). There was a brief summation of each case history. The learners would then choose their recommended treatment plan for the selected patient based on the available information. Treatment plans described in the cases ranged in perceived intensity from “best supportive care” to “aggressive” treatment regimens and participants were asked to discuss their selection and reasons for selection. Later in the lecture, learners were given the results of a comprehensive geriatric assessment for each case (see appendix G – I). Participants were instructed to list patient information into either the CARG or CRASH model to estimate chemotherapy toxicity. Learners then discussed together if their treatment recommendations changed from their initial assessment based on the findings from these tools.   Approximate activity time: 15-20 minutes |
