# Supplementary material for: An Introductory Course on Geriatric Oncology
Source: MedEdPORTAL. 2024 Nov 14;20:11471. doi: 10.15766/mep_2374-8265.11471 (PMC11561070; doi:10.15766/mep_2374-8265.11471)
Supplement: Supplementary file 1 — Introduction to Geriatric Oncology.pptxThe Comprehensive Geriatric Assessment.pptxGeriatric Screening Tools.pptxBiology of Aging.pptxCancer Therapy in the Older Adult.pptxSummary of Interactive Sessions.docxSession 5 Patient Case 1.docxSession 5 Patient Case 2.docxSession 5 Patient Case 3.docxGeriatric Oncology Knowledge Assessment.docxKnowledge Assessment Answer Key.docxSelf-Perceived Competency Assessment.docxCurriculum Session Assessment.docx [file mep_2374-8265.11471-s001.zip › G. Session 5 Patient Case 1.docx]

Session 5, Cancer Therapy in the Older Adult

Patient Case – Colorectal Cancer:

Mrs Aguilar is a 68-year-old woman with stage III colon adenocarcinoma near the splenic flexure who comes to see you post-operatively for your recommendation on treatment. She recently underwent left partial colectomy with end-to-end anastomosis 3 weeks ago. This was an R0 resection. CT imaging shows no evidence of distant disease.

Post operatively, she has had a slow recovery with delayed wound healing and continued weakness/fatigue. She currently finds going out of her home difficult without assistance and she had 1 fall 2 weeks ago because she ‘lost her footing’. Prior to surgery, the patient was more active.

Pathology:

3/17 lymphnodes were positive for metastasis and surgical margins were negative. IHC shows her tumor is MMR proficient.

Past Medical History:

Hypertension, Type 2 Diabetes, diabetic neuropathy, vit D deficiency, hypothyroidism, obesity, hyperlipidemia, anxiety

Medications:

lisinopril, insulin lispro, insulin glargine, gabapentin, levothyroxine, atorvastatin, diazepam,

Performance Status: ECOG 2

Vital Signs:

Temp 97.5* HR 78 BP 154/85 Pain 0

Lab:

WBC 5,000 SCr 1.3

Hgb 9.8 LDH 150

Hct 29.4 A1C 11%

Plt 245,000

What treatment strategy would you recommend?

1. Full dose adjuvant chemotherapy such as FOLFOX for 6 months
2. Full dose adjuvant chemotherapy such as CAPEOX for 6 months
3. Singe agent F-FU or Capecitabine based treatment
4. Reassess the patient for therapy fitness in 3-4 weeks
5. Observation

Comprehensive Geriatric Assessment: Case 1

Basic ADL Score __6/6____ IADL Score __6/8____

Montreal Cognitive Assessment (MoCA) __27/30___

MMS ___30/30____

MNA ___28/30___

Patient’s Zarit Screening: __n/a___

Falls in the past year __1___

Timed-Up-and-Go __15 sec___

Matters Most/Treatment preferences: Prefers to remain at home to care for her dogs. She favors quality of life and is looking forward to recovering further from her surgery. She is willing to receive thearpy that may prolong her life for a limited time tradeoff in quality of life.

More info for toxicity assessment:

Height 61 in

Weight 195lbs

Hearing: Excellent

Take medications: Without help

Walking 1 block: Limited a lot

Interference with social activities: Most of the time

Chemotherapy Toxicity Tools

Cancer and Aging Research Group (CARG) Chemo-Toxicity Score*

Instructions: Please circle applicable risk factors and total score below.

| **Risk Factor** | **Score** |
| --- | --- |
| Age ≥ 72 | 2 |
| Gastrointestinal or Genitourinary Cancer | 2 |
| Standard dose chemotherapy | 2 |
| >1 chemotherapy drug | 2 |
| Hemoglobin <11 (male) or < 10 (female) | 3 |
| Creatinine Clearance <34mL/min | 3 |
| Hearing, fair or worse | 2 |
| 1 or more falls in the past 6 months | 3 |
| Needs help with taking medications | 1 |
| Walking 1 block somewhat limited | 2 |
| Decreased social activity due to health | 1 |
| **Total** |  |

| **Risk Category** | **Low** | **Intermediate** | **High** |
| --- | --- | --- | --- |
| Score | 0-5 | 6-9 | 10-19 |

*Citation included in ESR summary document.

CRASH (Chemotherapy Risk Age Scale for High-Risk Patients) Score*

Chemotherapy Risk

Score ______________

| **Points (Circle one)** | | |
| --- | --- | --- |
| **0** | **1** | **2** |
| Ado-trastuzumab emtansine | Bendamustine (90mg/m2) + rituximab | 5-FU/LV |
| Capecitabine 2g/m2 | Capecitabine 2.5g/m2 +/- trastuzumab |  |
| Chlorambucil daily + rituximab | Carboplatin/gemcitabine AUC 4-6/1g d1, d8 | 5-FU/LV + bevacizumab |
| Cisplatin 75/gemcitabine d1,8 | Carboplatin/pemetrexed | AC |
| Cisplatin/pemetrexed | Carboplatin/paclitaxel q3w | CAF |
| Dacarbazine | Cisplatin 100/gemcitabine d1,8 | Carboplatin/docetaxel q3w |
| Docetaxel weekly | ECF | CHOP |
| FOLFIRI | Fludarabine | Cisplatin/docetaxel 75/75 |
| Gemcitabine 1g 3/4 weeks | FOLFOX 85mg/m2 | Cisplatin/etoposide |
| Gemcitabine 1.25g 3/4 weeks | Gemcitabine 7/8 weeks then 3/4 | Cisplatin/gemcitabine d1,8,15 |
| Paclitaxel weekly or 3/4 weeks | Gemcitabine/irinotecan | Cisplatin/irinotecan |
| Pemetrexed | PEG doxorubicin 50q4w | Cisplatin/paclitaxel 135-24h q3w |
|  |  | Doxorubicin q3w |
|  |  | FOLFOX 100-130 mg/m2 |
|  |  | Gemcitabine/docetaxel |
|  |  | Gemcitabine/nab-paclitaxel |
|  |  | Gemcitabine/pemetrexed d8 |
|  |  | Irinotecan q3w |
|  |  | Paclitaxel q3w |
|  |  | Docetaxel q3w |
|  |  | Topotecan monthly |

Regimens not listed should be scored by analogy.

Hematologic Risk Factors

Score ________

| **Diastolic Blood Pressure** | |
| --- | --- |
| >72 | 1 |
| Otherwise | 0 |
| **IADL** | |
| <26 | 1 |
| Otherwise | 0 |
| **LDH** | |
| >459 | 1 |
| Otherwise | 0 |

Non-Hematologic Risk Factors

Score _______

| **ECOG Performance Status** | |
| --- | --- |
| 0 | 0 |
| 1-2 | 1 |
| 3-2 | 2 |
| **MMS (Mini Mental State Exam)** | |
| <30 | 2 |
| 30 | 0 |
| **MNA (Mini Nutritional Assessment)** | |
| <28 | 2 |
| Otherwise | 0 |

MMS and MNA assessments are not outlined in this activity.

Combined Score __________

| Risk Category | Low | Int-Low | Int-High | High |
| --- | --- | --- | --- | --- |
| Combined Score | 0-3 | 4-6 | 7-9 | >9 |
| % with severe toxicity based on derivation sample | 50% | 58% | 77% | 79% |

*Citation included in ESR summary document.

The following URL/QR Codes are provider for learner convenience to access e-calculator tools online for the CARG and CRASH toxicity scores. These scoring systems are website-based versions of the tables listed above. They are OPTIONAL and their use is NOT required for this learning activity. Also provided below is a URL/QR Code for the ePrognosis calculation tool. This resource is OPTIONAL and NOT required for this learning activity.


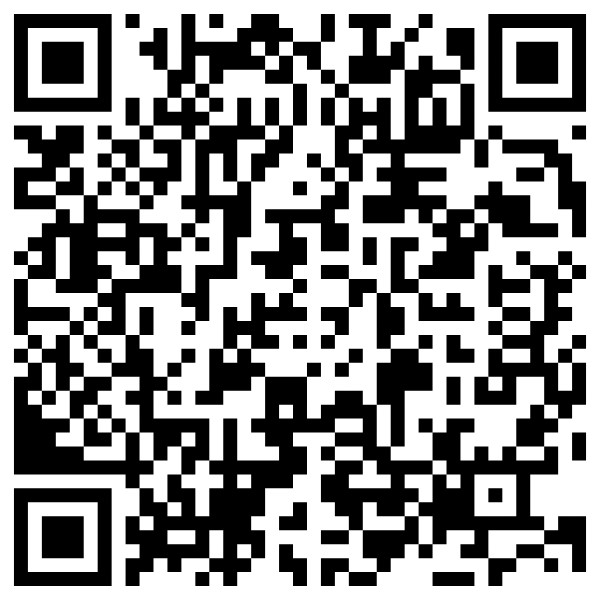

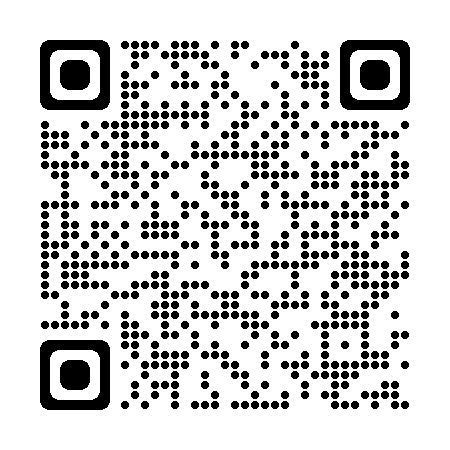

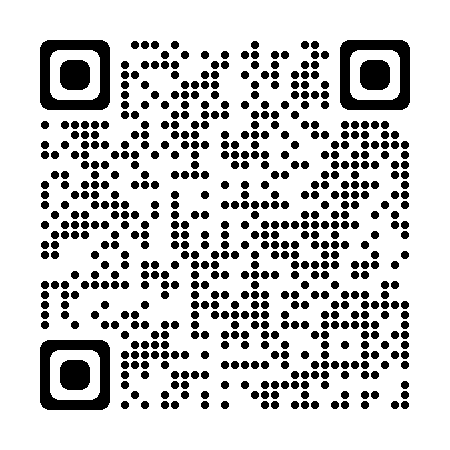


ePrognosis tool

CRASH Score

CARG Toxicity Score
