# Supplementary material for: An Introductory Course on Geriatric Oncology
Source: MedEdPORTAL. 2024 Nov 14;20:11471. doi: 10.15766/mep_2374-8265.11471 (PMC11561070; doi:10.15766/mep_2374-8265.11471)
Supplement: Supplementary file 1 — Introduction to Geriatric Oncology.pptxThe Comprehensive Geriatric Assessment.pptxGeriatric Screening Tools.pptxBiology of Aging.pptxCancer Therapy in the Older Adult.pptxSummary of Interactive Sessions.docxSession 5 Patient Case 1.docxSession 5 Patient Case 2.docxSession 5 Patient Case 3.docxGeriatric Oncology Knowledge Assessment.docxKnowledge Assessment Answer Key.docxSelf-Perceived Competency Assessment.docxCurriculum Session Assessment.docx [file mep_2374-8265.11471-s001.zip › H. Session 5 Patient Case 2.docx]

Session 5, Cancer Therapy in the Older Adult

Patient Case – Non-small cell lung cancer:

Mr. Hodges is a 73-year-old man with a 45 pack year smoking history and recently diagnosed, metastatic squamous cell carcinoma of the lung based on tissue biopsy of an enlarging right middle lobe spiculated pulmonary nodule and bone biopsy of a vertebral lesion. He displays significant comorbidity in the form of advanced COPD with several hospitalizations in the past 2 years for COPD exacerbations, but remains oxygen independent. His pulmonologist is concerned for possible early development of ILD based on radiographic findings. He continues to smoke 1/2 PPD.

The patient reports he has similar, chronic dyspnea on exertion, but no recent increase in cough or sputum production and no SOB at rest. He does complain of worsening bony pain located at multiple sites along the thoracic spine and rib cage. He uses a cane for ambulation.

Pathology:

NGS of tumor tissue is negative for actionable mutations in EGFR, ALK, ROS1, MET, RET, or KRAS.

PDL1 TPS 5%

Past Medical History:

Heavy tobacco use, severe COPD (GOLD 3), chronic low back pain s/p spinal stimulator, osteoarthritis, peripheral artery disease

Medications:

aspirin, budesonide-formoterol, tiotropium, albuterol rescue inhaler, tramadol

Performance Status: ECOG 1

Vital Signs:

Temp 97.5* HR 78 BP 154/85 Pain 5 RR 16 pO2 93%

Lab:

WBC 5,000 SCr 1.3

Hgb 12.6 LDH 150

Hct 37.8

Plt 245,000

What treatment strategy would you recommend?

1. Full dose platinum-doublet chemotherapy plus immunotherapy
2. Reduced dose platinum-doublet chemotherapy plus immunotherapy
3. Single agent chemotherapy
4. Immunotherapy alone
5. Best supportive care

Comprehensive Geriatric Assessment: Case 2

Basic ADL Score __6/6____ IADL Score __8/8____

Montreal Cognitive Assessment (MoCA) __27/30___

MMS ___29/30____

MNA ___22/30___

Patient’s Zarit Screening: __n/a___

Falls in the past year __0___

Timed-Up-and-Go __11 sec___

Matters Most/Treatment preferences: Patient is independent and wants to stay that way. He wants to avoid hospitals and nursing facilities at all costs. He prizes functional and independence above survival. He currently lives alone and drives to all appointments. He has no immediate family or friends to rely upon and no surrogate medical decision maker.

More info for toxicity assessment:

Height 75 in

Weight 160lbs

Hearing: Fair

Take medications: Without help

Walking 1 block: Limited a little

Interference with social activities: “None of the time”

Chemotherapy Toxicity Tools

Cancer and Aging Research Group (CARG) Chemo-Toxicity Score*

Instructions: Please circle applicable risk factors and total score below.

| **Risk Factor** | **Score** |
| --- | --- |
| Age ≥ 72 | 2 |
| Gastrointestinal or Genitourinary Cancer | 2 |
| Standard dose chemotherapy | 2 |
| >1 chemotherapy drug | 2 |
| Hemoglobin <11 (male) or < 10 (female) | 3 |
| Creatinine Clearance <34mL/min | 3 |
| Hearing, fair or worse | 2 |
| 1 or more falls in the past 6 months | 3 |
| Needs help with taking medications | 1 |
| Walking 1 block somewhat limited | 2 |
| Decreased social activity due to health | 1 |
| **Total** |  |

| **Risk Category** | **Low** | **Intermediate** | **High** |
| --- | --- | --- | --- |
| Score | 0-5 | 6-9 | 10-19 |

*Citation included in ESR summary document.

CRASH (Chemotherapy Risk Age Scale for High-Risk Patients) Score*

Chemotherapy Risk

Score ______________

| **Points (Circle one)** | | |
| --- | --- | --- |
| **0** | **1** | **2** |
| Ado-trastuzumab emtansine | Bendamustine (90mg/m2) + rituximab | 5-FU/LV |
| Capecitabine 2g/m2 | Capecitabine 2.5g/m2 +/- trastuzumab |  |
| Chlorambucil daily + rituximab | Carboplatin/gemcitabine AUC 4-6/1g d1, d8 | 5-FU/LV + bevacizumab |
| Cisplatin 75/gemcitabine d1,8 | Carboplatin/pemetrexed | AC |
| Cisplatin/pemetrexed | Carboplatin/paclitaxel q3w | CAF |
| Dacarbazine | Cisplatin 100/gemcitabine d1,8 | Carboplatin/docetaxel q3w |
| Docetaxel weekly | ECF | CHOP |
| FOLFIRI | Fludarabine | Cisplatin/docetaxel 75/75 |
| Gemcitabine 1g 3/4 weeks | FOLFOX 85mg/m2 | Cisplatin/etoposide |
| Gemcitabine 1.25g 3/4 weeks | Gemcitabine 7/8 weeks then 3/4 | Cisplatin/gemcitabine d1,8,15 |
| Paclitaxel weekly or 3/4 weeks | Gemcitabine/irinotecan | Cisplatin/irinotecan |
| Pemetrexed | PEG doxorubicin 50q4w | Cisplatin/paclitaxel 135-24h q3w |
|  |  | Doxorubicin q3w |
|  |  | FOLFOX 100-130 mg/m2 |
|  |  | Gemcitabine/docetaxel |
|  |  | Gemcitabine/nab-paclitaxel |
|  |  | Gemcitabine/pemetrexed d8 |
|  |  | Irinotecan q3w |
|  |  | Paclitaxel q3w |
|  |  | Docetaxel q3w |
|  |  | Topotecan monthly |

Regimens not listed should be scored by analogy.

Hematologic Risk Factors

Score ________

| **Diastolic Blood Pressure** | |
| --- | --- |
| >72 | 1 |
| Otherwise | 0 |
| **IADL** | |
| <26 | 1 |
| Otherwise | 0 |
| **LDH** | |
| >459 | 1 |
| Otherwise | 0 |

Non-Hematologic Risk Factors

Score _______

| **ECOG Performance Status** | |
| --- | --- |
| 0 | 0 |
| 1-2 | 1 |
| 3-2 | 2 |
| **MMS (Mini Mental State Exam)** | |
| <30 | 2 |
| 30 | 0 |
| **MNA (Mini Nutritional Assessment)** | |
| <28 | 2 |
| Otherwise | 0 |

MMS and MNA assessments are not outlined in this activity.

Combined Score __________

| Risk Category | Low | Int-Low | Int-High | High |
| --- | --- | --- | --- | --- |
| Combined Score | 0-3 | 4-6 | 7-9 | >9 |
| % with severe toxicity based on derivation sample | 50% | 58% | 77% | 79% |

*Citation included in ESR summary document.

The following URL/QR Codes are provider for learner convenience to access e-calculator tools online for the CARG and CRASH toxicity scores. These scoring systems are website-based versions of the tables listed above. They are OPTIONAL and their use is NOT required for this learning activity. Also provided below is a URL/QR Code for the ePrognosis calculation tool. This resource is OPTIONAL and NOT required for this learning activity.


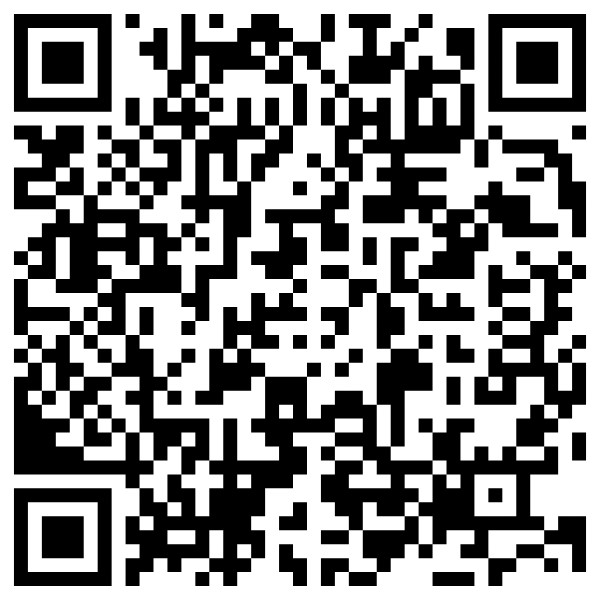

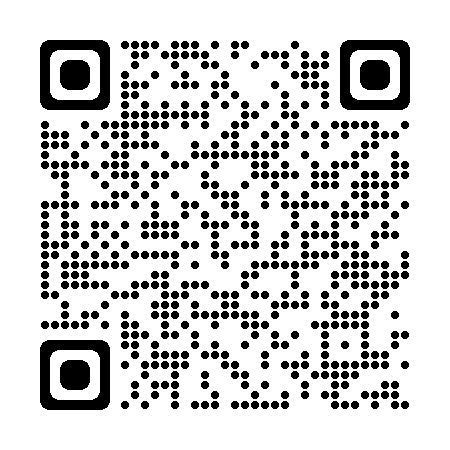

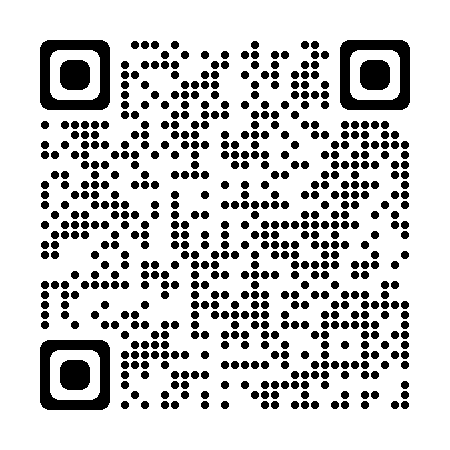


ePrognosis tool

CRASH Score

CARG Toxicity Score
