# Supplementary material for: An Introductory Course on Geriatric Oncology
Source: MedEdPORTAL. 2024 Nov 14;20:11471. doi: 10.15766/mep_2374-8265.11471 (PMC11561070; doi:10.15766/mep_2374-8265.11471)
Supplement: Supplementary file 1 — Introduction to Geriatric Oncology.pptxThe Comprehensive Geriatric Assessment.pptxGeriatric Screening Tools.pptxBiology of Aging.pptxCancer Therapy in the Older Adult.pptxSummary of Interactive Sessions.docxSession 5 Patient Case 1.docxSession 5 Patient Case 2.docxSession 5 Patient Case 3.docxGeriatric Oncology Knowledge Assessment.docxKnowledge Assessment Answer Key.docxSelf-Perceived Competency Assessment.docxCurriculum Session Assessment.docx [file mep_2374-8265.11471-s001.zip › I. Session 5 Patient Case 3.docx]

Appendix I: Session 5, Cancer Therapy in the Older Adult

Patient Case – Acute Myeloid Leukemia:

Mr. Williams is a 79-year-old man you are seeing in the hospital who was recently diagnosed on this admission with AML. He complains of significant fatigue and worsening dyspnea on exertion as chief complaints. Additionally, he has noticed increased bruising and gum bleeding with minor trauma over the past 2-3 weeks. He denies depressed mood, but reports some anxiety about this diagnosis and how he will help care for his wife with moderate dementia. He stays active during the day and volunteers with his church and local VA hospital.

Pathology:

His baseline bone marrow biopsy reveals 45% myeloblasts. FISH for PML-RARA is negative. Cytogenetics are complex with 4 different abnormalities seen. Further molecular profiling is pending.

Past Medical History:

HTN, CAD s/p PCI with DES x2 in 2018, HFpEF, prior tobacco use (35 pack years, quit in 2018).

Medications:

aspirin, metoprolol, losartan, furosemide PRN

Performance Status: ECOG 2

Vital Signs:

Temp 97.5* HR 78 BP 154/85 Pain 0 RR 18 pO2 98%

Lab:

WBC 34,000 SCr 0.9

Hgb 6.0 LDH 652

Hct 18.1

Plt 25,000

What treatment strategy would you recommend?

1. Intensive chemotherapy (e.g. 7+3)
2. Intermediate intensity chemotherapy (e.g. azacytidine +Venetoclax)
3. Low intensity chemotherapy (e.g. azacytidine alone)
4. Wait for molecular testing results
5. Best supportive care

Comprehensive Geriatric Assessment: Case 3

Basic ADL Score __6/6____ IADL Score __5/8____

Montreal Cognitive Assessment (MoCA) __23/30___

MMS ___24/30___

MNA ___28/30___

Patient’s Zarit Screening: __not performed___

Falls in the past year __4___

Timed-Up-and-Go __not performed___

Matters Most/Treatment preferences: Patient would like to live as long as possible, but he most prizes being at home to help take care of his life with moderate dementia.

Chemotherapy Toxicity Tools

Cancer and Aging Research Group (CARG) Chemo-Toxicity Score*

Instructions: Please circle applicable risk factors and total score below.

| **Risk Factor** | **Score** |
| --- | --- |
| Age ≥ 72 | 2 |
| Gastrointestinal or Genitourinary Cancer | 2 |
| Standard dose chemotherapy | 2 |
| >1 chemotherapy drug | 2 |
| Hemoglobin <11 (male) or < 10 (female) | 3 |
| Creatinine Clearance <34mL/min | 3 |
| Hearing, fair or worse | 2 |
| 1 or more falls in the past 6 months | 3 |
| Needs help with taking medications | 1 |
| Walking 1 block somewhat limited | 2 |
| Decreased social activity due to health | 1 |
| **Total** |  |

| **Risk Category** | **Low** | **Intermediate** | **High** |
| --- | --- | --- | --- |
| Score | 0-5 | 6-9 | 10-19 |

*Citation included in ESR summary document.

CRASH (Chemotherapy Risk Age Scale for High-Risk Patients) Score*

Chemotherapy Risk

Score ______________

| **Points (Circle one)** | | |
| --- | --- | --- |
| **0** | **1** | **2** |
| Ado-trastuzumab emtansine | Bendamustine (90mg/m2) + rituximab | 5-FU/LV |
| Capecitabine 2g/m2 | Capecitabine 2.5g/m2 +/- trastuzumab |  |
| Chlorambucil daily + rituximab | Carboplatin/gemcitabine AUC 4-6/1g d1, d8 | 5-FU/LV + bevacizumab |
| Cisplatin 75/gemcitabine d1,8 | Carboplatin/pemetrexed | AC |
| Cisplatin/pemetrexed | Carboplatin/paclitaxel q3w | CAF |
| Dacarbazine | Cisplatin 100/gemcitabine d1,8 | Carboplatin/docetaxel q3w |
| Docetaxel weekly | ECF | CHOP |
| FOLFIRI | Fludarabine | Cisplatin/docetaxel 75/75 |
| Gemcitabine 1g 3/4 weeks | FOLFOX 85mg/m2 | Cisplatin/etoposide |
| Gemcitabine 1.25g 3/4 weeks | Gemcitabine 7/8 weeks then 3/4 | Cisplatin/gemcitabine d1,8,15 |
| Paclitaxel weekly or 3/4 weeks | Gemcitabine/irinotecan | Cisplatin/irinotecan |
| Pemetrexed | PEG doxorubicin 50q4w | Cisplatin/paclitaxel 135-24h q3w |
|  |  | Doxorubicin q3w |
|  |  | FOLFOX 100-130 mg/m2 |
|  |  | Gemcitabine/docetaxel |
|  |  | Gemcitabine/nab-paclitaxel |
|  |  | Gemcitabine/pemetrexed d8 |
|  |  | Irinotecan q3w |
|  |  | Paclitaxel q3w |
|  |  | Docetaxel q3w |
|  |  | Topotecan monthly |

Regimens not listed should be scored by analogy.

Hematologic Risk Factors

Score ________

| **Diastolic Blood Pressure** | |
| --- | --- |
| >72 | 1 |
| Otherwise | 0 |
| **IADL** | |
| <26 | 1 |
| Otherwise | 0 |
| **LDH** | |
| >459 | 1 |
| Otherwise | 0 |

Non-Hematologic Risk Factors

Score _______

| **ECOG Performance Status** | |
| --- | --- |
| 0 | 0 |
| 1-2 | 1 |
| 3-2 | 2 |
| **MMS (Mini Mental State Exam)** | |
| <30 | 2 |
| 30 | 0 |
| **MNA (Mini Nutritional Assessment)** | |
| <28 | 2 |
| Otherwise | 0 |

MMS and MNA assessments are not outlined in this activity.

Combined Score __________

| Risk Category | Low | Int-Low | Int-High | High |
| --- | --- | --- | --- | --- |
| Combined Score | 0-3 | 4-6 | 7-9 | >9 |
| % with severe toxicity based on derivation sample | 50% | 58% | 77% | 79% |

*Citation included in ESR summary document.

The following URL/QR Codes are provider for learner convenience to access e-calculator tools online for the CARG and CRASH toxicity scores. These scoring systems are website-based versions of the tables listed above. They are OPTIONAL and their use is NOT required for this learning activity. Also provided below is a URL/QR Code for the ePrognosis calculation tool. This resource is OPTIONAL and NOT required for this learning activity.


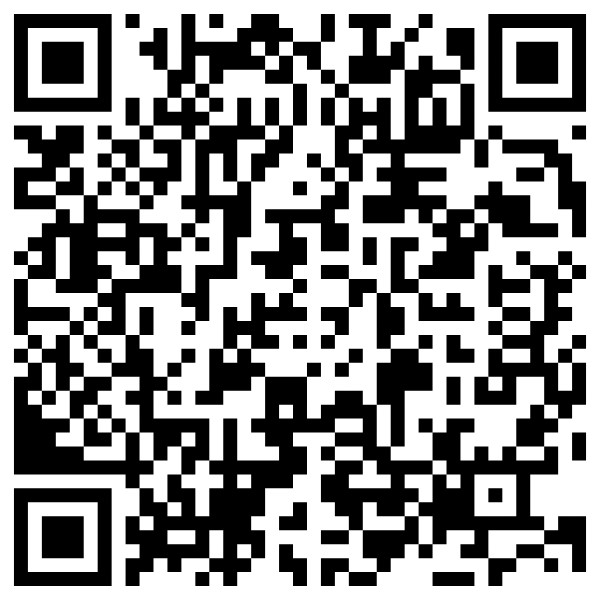

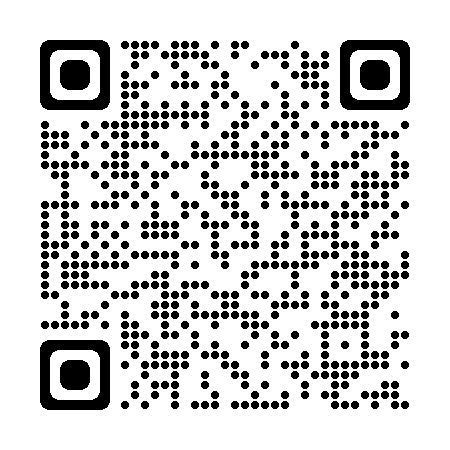

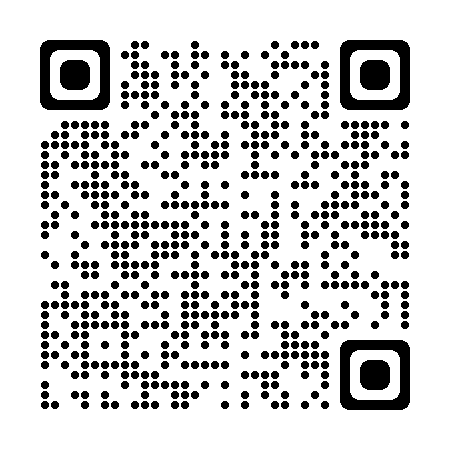


ePrognosis tool

CRASH Score

CARG Toxicity Score
